# Developmental system drift in dorsoventral patterning is linked to transitions to autonomous development in Annelida

**DOI:** 10.1038/s41467-026-71950-7

**Published:** 2026-04-18

**Authors:** Allan M. Carrillo-Baltodano, Emmanuel Haillot, Steffanie Mutiara Meha, Imran Luqman, Artenis Pashaj, Jimena Montagne, Yun-Ju Lee, Tsai-Ming Lu, David E. K. Ferrier, Stephan Q. Schneider, José M. Martín-Durán

**Affiliations:** 1 School of Biological and Behavioural Studies, Queen Mary University of London, London, UK; 2 Institute of Cellular and Organismic Biology, Academia Sinica, Taipei, Taiwan; 3 Department of Neurosciences and Developmental Biology, Faculty of Life Sciences, University of Vienna, Vienna, Austria; 4 Molecular and Biological Agricultural Sciences Program, Taiwan International Graduate Program, Academia Sinica and National Chung Hsing University, Taipei, Taiwan; 5 Graduate Institute of Biotechnology, National Chung Hsing University, Taichung, Taiwan; 6 The Marine Biological Association, Plymouth, UK; 7 Kingston University, London, UK; 8 The Scottish Oceans Institute, Gatty Marine Laboratory, School of Biology, University of St. Andrews, St. Andrews, UK; 9 Biotechnology Center, National Chung Hsing University, Taichung, Taiwan

**Keywords:** Cell signalling, Evolutionary developmental biology, Body patterning

## Abstract

The Bone Morphogenetic Protein (BMP) pathway is the ancestral signalling system patterning the dorsoventral axis in bilaterally symmetrical animals. However, in Spiralia, a large bilaterian clade including molluscs and annelids, BMP’s axial function varies across species, obscuring the ancestral developmental role of this pathway. Here, we study four annelid species to demonstrate that BMP is ancestrally downstream of ERK1/2 and promotes dorsoventral development in Annelida. Importantly, this signalling hierarchy is lost in annelids that secondarily transitioned to a maternally controlled, unequal cleavage, with some using Activin/Nodal and others relying on BMP to establish dorsoventral polarity only in the head. Unexpectedly, this divergence implies extensive rewiring of downstream targets involved in dorsoventral patterning. Together, our data clarify BMP’s ancestral axial role in Spiralia, uncovering a potential causal link between parallel shifts toward unequal spiral cleavage and the emergence of developmental system drift, a pervasive yet poorly understood phenomenon in animal embryogenesis.

## Abstract (web summary)

Here they show that BMP signalling is the ancestral pathway that patterns the dorsoventral (DV) axis in Annelida and Spiralia. The shift to unequal cleavage involved alternative pathways for patterning the DV axis, leading to a unique case of developmental system drift.

## Introduction

**Fig. 1:**
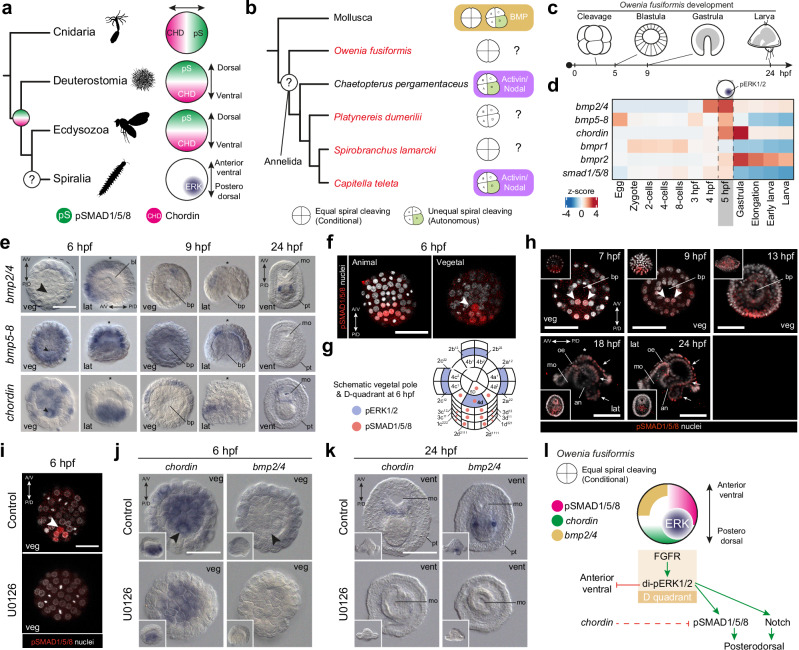
A dorsoventral gradient of pSMAD1/5/8 in the annelid *Owenia fusiformis.* **a** BMP and BMP antagonists (e.g., Chordin) establish a pSMAD1/5/8 gradient that defines the secondary axis in cnidarians and the dorsoventral (DV) axis in bilaterians. In Spiralia, one cell activated by ERK acts as an axial organiser, and BMP’s role in DV patterning is contentious. Phylopic images of *Nematostella*, sea urchin, *Drosophila* and annelid are under the CCO 1.0 public domain license. **b** In the studied annelid species, *C. teleta* and *C. pergamentaceus*, which have unequal spiral cleavage, Activin/Nodal specifies the DV axis. Whether this represents the ancestral annelid state is unclear. To solve this, we have studied four annelids (in red) with different modes of spiral cleavage. **c** Schematic diagram of *O. fusiformis*’ embryogenesis. **d** Heatmap of the relative expression of members of the BMP pathway during the development of *O. fusiformis*. **e** Whole mount in situ hybridisation of a subset (ligands and antagonist) of the BMP pathway genes in blastula (6 h post-fertilisation, hpf), gastrula (9 hpf) and larval stages (24 hpf). Dotted line in *bmp2/4* at 6 hpf indicates the area of expression. **f**, **h**,** i** Z-stack projections of embryos at 6-, 7-, 9-, 13-, 18- and 24 hpf stained against pSMAD1/5/8 (red) and nuclei (DAPI). Arrows in **h** indicate the posterodorsal pSMAD1/5/8 signal in the ectoderm and endoderm. **g** Schematic representation of the vegetal pole during the activation of pSMAD1/5/8. Cell identities follow the standard spiralian nomenclature. **j**, **k** Whole-mount in situ hybridisation of *chordin* and *bmp2/4* demonstrates that inhibition of ERK1/2 activation prevents BMP activation. **l** Schematic representation of the signalling regulatory interactions specifying the bilateral symmetry in *O. fusiformis*. Arrowheads in (**e**, **f**) and (**h**, **i**) point to the embryonic organiser cell (4d) or its subsequent daughter cells 4d^1^ and 4d^2^. An asterisk marks the animal/apical. In **e**–**l**, animal/vegetal views are with the 4d to the bottom, and lateral views are with anteroventral to the left. Scale bars are 50 µm. an anus, bl blastocoel, bp blastopore, lat lateral, mo mouth, oe oesophagus, pt prototroch, veg vegetal, vent ventral. For whole-mount in situ hybridisation and immunostaining, we show representatives of at least two independent experiments. Drawings are not to scale. Source data are provided as a Source Data file.

More than 99% of known animals exhibit bilateral symmetry^1,2^, with their anatomies organised along two orthogonal symmetry planes referred to as the primary anteroposterior (AP) and the secondary dorsoventral (DV) axes. Remarkably, distantly related bilaterian lineages use homologous developmental signalling pathways to form their body axes^3,4^. In Deuterostomia (i.e., chordates, echinoderms, and hemichordates) and Ecdysozoa (e.g., arthropods), the Bone Morphogenetic Protein (BMP) pathway patterns the DV axis through the interplay of extracellular secreted ligands (BMP2/4) and antagonists (Chordin). This creates an intracellular morphogenetic gradient of active phosphorylated SMAD1/5/8 (pSMAD1/5/8) that accumulates in the opposite pole to the source of the antagonist, promoting dorsal structures in non-chordates and ventral structures in chordates^5^ (Fig. 1a). Notably, an opposing gradient along the secondary body axis of BMP antagonists and pSMAD1/5/8 also occurs in Cnidaria^6^, the sister group to Bilateria^2^. Therefore, an upstream role of the BMP pathway in the body symmetry and patterning of the DV axis is likely ancestral to Bilateria^2,5^ (Fig. 1a). In Spiralia (e.g. molluscs and annelids), the third major lineage of bilaterian animals^7^, the initial symmetry-breaking event and specification of the AP and DV axis ancestrally involved the Fibroblast Growth Factor receptor (FGFR) and ERK1/2 pathways^8^ (Fig. 1a). In contrast, the developmental role of the BMP signalling pathway varies dramatically across the studied spiralian species^5,9^.

Spiralia is characterised by an ancestral, stereotypical early-embryonic programme known as spiral cleavage^10,11^. In molluscs and annelids, the ancestral type of spiral cleavage that exhibits two first rounds of equal zygotic cell divisions and relies on FGFR-ERK1/2-mediated inductive interactions to specify the embryonic organiser (the so-called “equal spiral cleavage”) has independently diverged multiple times into a mode of spiral cleavage where the primary blastomere identities are defined autonomously and maternally through early asymmetric cell divisions (the so-called “unequal spiral cleavage”)^8,9^. In some molluscs, regardless of the cleavage type, BMP is downstream of ERK1/2 activity and controls DV development, sometimes promoting and sometimes repressing neurogenesis^12,13^. However, in other molluscs, BMP controls only head and anterior neural development^14^.

Surprisingly, BMP does not seem to play a role in DV patterning in annelids^15–18^. However, only species with the derived unequal mode of spiral cleavage have been studied (Fig. 1b). Moreover, a complex pattern of gene loss characterises the evolutionary history of *chordin* in annelids^19^. Accordingly, related signalling pathways, such as Activin/Nodal, and lineage-specific signalling interactions pattern the annelid DV axis^15–17,20^. To complicate things further, brachiopods, a spiralian lineage that has lost the ancestral spiral cleavage^10^, exhibit a Deuterostomia- and Ecdysozoa-like condition, where BMP signalling controls DV patterning, and FGFR participates in late stages of mesoderm development^21–23^. Therefore, the ancestral role of the BMP signalling pathway remains contentious in Spiralia, preventing a fundamental understanding of how dorsoventral patterning occurs and has evolved in a major bilaterian lineage representing nearly half of the animal kingdom.

Here, we apply a phylum-wide comparative approach to resolve the role of the BMP and Activin/Nodal signalling pathways in Annelida. By studying two distantly related annelids with the ancestral equal spiral cleavage, *Owenia fusiformis* and *Spirobranchus lamarcki*, we demonstrate that ERK1/2 activates BMP signalling on the prospective dorsal side of the embryo, which is required to activate downstream genes involved in the development of dorsoposterior structures. This is similar to what has been described in molluscs with equal and unequal cleavage^12,13,24^ and likely represents the ancestral condition for Annelida and Spiralia. However, the annelid *Platynereis dumerilii*, a species with the derived unequal spiral cleavage, deploys an axial patterning role of the BMP pathway preferentially in the head region. Notably, this is unlike other annelids with unequal development, such as *Capitella teleta* and *Chaetopterus pergamentaceus*, which use Activin/Nodal instead^16,17,20^. In *P. dumerilii*, Activin/Nodal does not affect DV polarity. Still, it contributes to the normal development of DV structures in *O. fusiformis*, albeit through regulating different downstream genes than the BMP signalling. Altogether, our findings clarify the ancestral axial role of the BMP pathway in Annelida and Spiralia, further supporting the deep evolutionary conservation of body-patterning mechanisms across spiralian taxa with ancestral equal spiral cleavage and a conditional mode of specifying the cell lineage that forms the embryonic organiser (the so-called D quadrant). Importantly, parallel independent transitions into unequal development and autonomous specification of the D quadrant co-occurred with developmental system drift (DSD) in DV patterning in Annelida, uncovering a unique and tractable system to explore the evolutionary forces and developmental mechanisms underpinning DSD, a widespread yet poorly understood phenomenon in evolutionary developmental biology.

## Results

### A dorsoventral gradient of pSMAD1/5/8 in O. fusiformis

To elucidate the role of BMP signalling in *O. fusiformis*, an early-branching annelid that develops via the ancestral equal spiral cleavage mode, we first looked at the expression dynamics of the core components of this pathway during embryogenesis (Fig. 1c–e). The expression of the ligands *bmp2/4* and *bmp5-8* peaks at 5 h post fertilisation (hpf) when the axial organising cell —the 4d blastomere— gets specified (Fig. 1d). At 5 and 6 hpf, *bmp2/4* is expressed on the anterior region of the embryo, overlapping anteriorly with the broad vegetal expression of the antagonist *chordin*^19^, whose expression peaks with gastrulation (Fig. 1d, e). At that stage (9 hpf), *bmp2/4*, *bmp5-8*, and *chordin* seem to be restricted to non-overlapping, staggered expression domains in the presumptive anteroventral ectoderm of the embryo (based on the position of 4d and its progeny), with *chordin* expressed more vegetally and *bmp5-8* closer to the animal pole (Fig. 1e). However, by the larval stage (24 hpf), *chordin* and *bmp2/4* are expressed in separate areas, surrounding the mouth and just beneath the hindgut, respectively (Fig. 1e). Unlike the extracellular signalling players, the two BMP receptors^25^, *bmpr1* and *bmpr2*, and the secondary messenger *smad1/5/8* are ubiquitously expressed in the blastula and gastrula stages (Supplementary Fig. 1a), suggesting that most, if not all, cells could respond to BMP signalling during axial patterning despite the localised expression of the ligands and antagonists in *O. fusiformis*.

To identify the region with active BMP signalling during axial patterning in *O. fusiformis*, we next localised the activation of pSMAD1/5/8 with a cross-reactive antibody (Fig. 1f–h; Supplementary Fig. 1d). pSMAD1/5/8 is not detected at 5 hpf during the specification of the embryonic organiser^8^, but immediately after, at 6 hpf, in the organiser and cells of the dorsoposterior region extending from the animal to the vegetal pole (Fig. 1f, g). This pattern remains as gastrulation proceeds, with pSMAD1/5/8 remaining enriched in the progeny of the embryonic organiser, the dorsoposterior half of the embryo, as well as the larval dorsal gut and ectoderm, the posterior foregut and oesophagus (Fig. 1h). Notably, active pSMAD1/5/8 at the blastula and gastrula stages occurs on the opposite side to the area of expression of *bmp2/4*, *bmp5-8*, and *chordin* (Fig. 1e). Thus, there is a gradient of pSMAD1/5/8 activity along the DV axis in the embryos of *O. fusiformis*, which, as in other bilaterians and cnidarians^5,26^, might emerge from the shuttling of BMP ligands by antagonists from the ventral—where the two signalling molecules are co-expressed—to the dorsal side.

Given that the first signs of pSMAD1/5/8 activity occur after the FGFR-ERK1/2-mediated signalling event defining the bilateral symmetry of the embryo^8^, we hypothesised that ERK1/2 activity could control the activation of pSMAD1/5/8 on the presumptive dorsoposterior side. To test this, we first examined the protein domain composition of pSMAD1/5/8 to identify ERK1/2-mediated phosphorylation sites, and then treated embryos with U0126, a specific inhibitor of ERK1/2 activation that prevents the specification of the axial embryonic organiser^8^. In vertebrates, ERK regulates SMAD1 by phosphorylating residues in the linker region between the two MAD domains^27–29^. However, these residues are conserved only across vertebrates and, to a certain extent, in invertebrate deuterostomes (Supplementary Fig. 1b, c). Treatment with U0126 from 4 to 6 hpf completely blocked activation of pSMAD1/5/8 (Fig. 1i; Supplementary Table 1), downregulating the expression of *bmp2/4* and *chordin* at the gastrula and larval stages, resulting in a radialised phenotype (Fig. 1j, k; Supplementary Table 2). Interestingly, *bmp5-8* is not downregulated at the blastula stages after pERK1/2 inhibition^8^. Therefore, FGFR-ERK1/2 establishes the bilateral symmetry in *O. fusiformis* embryos by regulating BMP signalling and the activation of pSMAD1/5/8 in the prospective dorsoposterior side, as also observed in some molluscs^12,13,24^ (Fig. 1l). Notably, the fact that ERK1/2 is only active in the 4d cell whereas pSMAD1/5/8 is broadly enriched in the dorsoposterior side suggests that the regulatory interaction between ERK1/2 and SMAD1/5/8 is, at least partially, indirect, perhaps by modulating *chordin* expression or through the Notch-Delta pathway, a direct downstream of ERK1/2 whose impairment affects dorsoposterior development^8^.

### BMP signalling specifies the DV axis in O. fusiformis

**Fig. 2:**
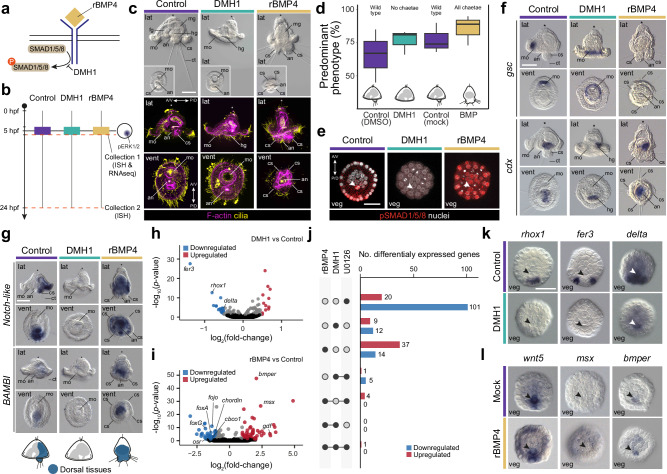
BMP controls a dorsoposterior gene regulatory network in *O. fusiformis.* **a** Simplified schematic of the BMP pathway and mode of action of DMH1 and recombinant BMP4 (rBMP4). **b** Schematic representation of the experimental design for the treatment windows and sample collections. **c** Differential interference contrast and z-stack projections of control and DMH1/rBMP4 larvae treated from 4 to 6 hpf. Insets in the first row are ventral views. Cilia (yellow) are labelled with tubulin, and F-actin (magenta) is labelled with phalloidin. **d** Median percentage of predominant phenotypes for each treatment. DMSO- and mock-control larvae have wild-type morphology. DMH1 has a reduced dorsoposterior region (i.e., no chaetae), while rBMP4-treated larvae have ectopic dorsoposterior tissue. **e** Z-stack projections of blastula (6 hpf) treated with control and DMH1/rBMP4 and stained with pSMAD1/5/8. Box plots representing the lower and upper quartiles, whiskers the minimum and maximum values (*n* = 3, per treatment). **f**,** g** Whole-mount in situ hybridisation of control and DMH1/rBMP4 larvae treated from 4 to 6 hpf for anterior (*gsc*), posterior (*cdx*) and the dorsal markers *notch-like* and *BAMBI*. **h**, **i** Volcano plots (two-sided Wald test) showing differentially expressed genes after DMH1 and rBMP4 treatment at the blastula stage (6 hpf), treated from 4 to 6 hpf. **j** Number of differentially expressed genes in the different treatments, compared with the changes in gene expression at 5.5 hpf observed after ERK1/2 inhibition. Full circles connected with a line indicated shared differentially expressed genes. **k**, **l** Validation via whole-mount in situ hybridisation of genes downregulated with DMH1 and upregulated with rBMP4 at the blastula stage (6 hpf). In all panels, the arrowheads point to the 4D organiser, and asterisks mark the animal/apical pole. Lateral views are with anteroventral to the left, ventral and apical views are with anteroventral to the top. For whole-mount in situ hybridisation and immunostaining, we show representatives of at least two independent experiments. Scale bars are 50 µm. an anus, bp blastopore, ch chaetae, cs chaetal sac, fg foregut, lat lateral, mo mouth, pt prototroch, vent ventral, veg vegetal. Drawings are not to scale. Source data are provided as a Source Data file.

To investigate the role of the pSMAD1/5/8 DV gradient in axial patterning, we altered the BMP pathway by exposing *O. fusiformis* embryos with the specific BMP receptor inhibitor dorsomorphin homologue 1 (DMH1) and recombinant zebrafish BMP4 (rBMP4) from 4 to 6 hpf at different concentrations (Fig. 2a, b; Supplementary Fig. 2a, b and Supplementary Tables 4–6). Compared to the control condition, most (77%) larvae of 20 µM DMH1-exposed embryos lacked the posterodorsally located chaetal sac but had a well-formed gut (Fig. 2c, d; Supplementary Tables 4–6). Conversely, most (86%) larvae of embryos treated with rBMP4 had an almost radial-like morphology, with only one gut opening surrounded by multiple ectopic chaetal sacs (Fig. 2c, d; Supplementary Tables 4–6). Both treatments dysregulated pSMAD1/5/8 levels but not the specification of the embryonic organiser (as observed by the presence of the large 4d cell), with DMH1 exposure leading to a complete inhibition of SMAD1/5/8 (although we cannot exclude remaining non-detectable but biologically-relevant levels of pSMAD1/5/8) and rBMP4 activating pSMAD1/5/8 in all embryonic cells, as expected by the ubiquitous expression of the receptor (Fig. 2e; Supplementary Fig. 1a and Supplementary Tables 1, 3). Consistent with the morphological phenotypes, DMH1 treatment resulted in the expansion of the oral ectodermal marker *gsc*^8,23^, the loss of the dorsal markers *Notch-like* and *BAMBI*^8^, but not the expression of the hindgut marker *cdx*, indicating the larvae have an AP axis but lack DV polarity (Fig. 2f, g; Supplementary Tables 5, 7, 8). However, rBMP treatment resulted in the loss of *gsc* expression (but not of *cdx*, which tends to localise slightly asymmetrically on one side) and the expansion of *Notch-like* and *BAMBI* (Fig. 2f–g; Supplementary Tables 5, 7, 8). Therefore, BMP signalling establishes a gradient of pSMAD1/5/8 activity that is necessary and sufficient to pattern the dorsoventral axis in *O. fusiformis* and to ensure a coordinated development of bilateral symmetry in these embryos, perhaps by determining the cells in the D quadrant that will contribute to axial elongation along the primary axis^30^.

To dissect the gene regulatory network controlled by BMP signalling, we performed comparative bulk transcriptomic profiling between control, DMH1, and rBMP4-treated embryos at the onset of pSMAD1/5/8 activation at 6 hpf (Fig. 2b; Supplementary Fig. 3a, b). Differential expression analyses uncovered 12 upregulated and 19 downregulated genes after DMH1 treatment and 68 upregulated and 53 downregulated genes after rBMP4 (Fig. 2h, i; Supplementary Tables 9–13). This difference in the number of differentially expressed genes is consistent with the broader impact of rBMP4 on the embryo, which enriches pSMAD1/5/8 across all quadrants (Fig. 2e), and with the fact that samples were collected at 6 hpf, the onset of BMP signalling and, therefore, when only the earliest response genes might be active. Notably, five of the nine downregulated genes after DMH1 treatment are also downregulated after inhibition of ERK1/2 di-phosphorylation^8^, including *rhox1*, *fer3*, and *delta* (Fig. 2j–k; Supplementary Table 14). Unlike the first two, whose expression entirely depends on normal BMP signalling, *delta* remains expressed in the embryonic organiser, suggesting that, as hypothesised above, pSMAD1/5/8 activity mediates Notch/Delta signalling on the dorsal side but not in the 4d cell. Consistent with the morphological phenotype, rBMP4 treatment downregulates genes involved in anteroventral fates, such as *chordin*, *odd-skipped related* (*osr*) and *foxG* (Supplementary Fig. 3c, d, f) and expands genes localised in the embryonic organiser and other dorsoposterior blastomeres, such as *wnt5*, *msx*, and *bmper* (*crossveinless*) (Fig. 2l; Supplementary Table 15). Altogether, our data demonstrate that BMP signalling is required to activate dorsoposterior genes and sustain Notch/Delta activity while repressing anteroventral fates during axial patterning in *O. fusiformis*.

### BMP signalling affects neurogenesis in O. fusiformis

**Fig. 3:**
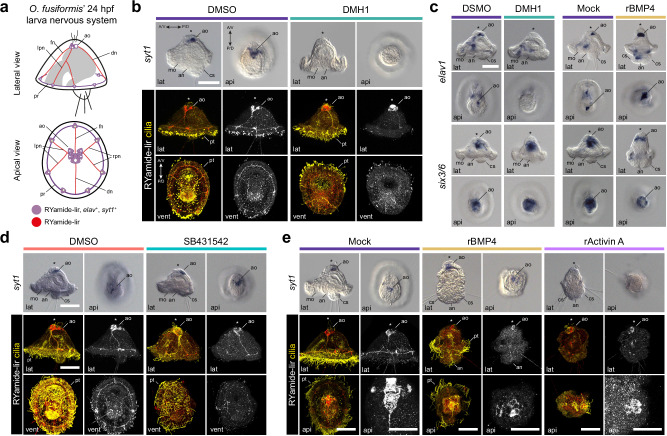
The role of BMP and Activin/Nodal in neural specification in *O. fusiformis.* **a** Schematic diagram of the larval nervous system at 24 hpf in *O. fusiformis*. **b** Whole mount in situ hybridisation of the neuronal marker *syt1* (top) and z-stack projections of RYamide immunoreactivity in control and treated larvae developed from embryos treated from 4 to 6 hpf with DMH1. **c** Whole-mount in situ hybridisation of the neural markers *elav1* and *six3/6* in control and treated larvae developed from embryos treated from 4 to 6 hpf with DMH1 and rBMP4. **d**–**e** Whole mount in situ hybridisation of the neuronal marker *syt1* (top) and z-stack projections of RYamide immunoreactivity in control and treated larvae developed from embryos treated from 4 to 6 hpf with the Activin/Nodal inhibitor SB431542 (**d**) and overactivated with rBMP4 and rActivin A (**e**). In all panels, the asterisks mark the apical pole. Lateral views are with anteroventral to the left, ventral and apical views are with anteroventral to the top. For whole-mount in situ hybridisation and immunostaining, we show representatives of at least two independent experiments. Scale bars are 50 µm. an anus, ao apical organ, api apical, at apical tuft, ch chatae, cs chaetal sac, dn dorsal nerve, fn frontal nerve, lat lateral, lpn left peripheral nerve, mg midgut, mo mouth, pr prototrochal ring, pt prototroch, rpn right peripheral nerve, vent ventral. Drawings are not to scale.

Associated with its role in setting up the DV axis, BMP signalling inhibits neural fates in vertebrates and arthropods^3,4^. Whether this also occurs in spiralians remains unclear^31^. In *O. fusiformis*, GO terms related to neural development are enriched after disturbing BMP signalling during axial patterning (Supplementary Fig. 3e, f). To further investigate the interplay between BMP signalling and neural development in this annelid, we analyse the expression of the pan-neural genes *elav1* and *synaptotagmin1* (*syt1*)^32,33^ and the conserved anterior neural marker *six3/6*, as well as the localisation of the neuropeptide RYamide^32,34–36^ (Fig. 3). The nervous system of the *O. fusiformis* larva consists of a neuropeptide-rich apical organ connected bilaterally to a neurite ring that surrounds, and presumably controls, the movement of the primary ciliary band, the prototroch^32,33,37^ (Fig. 3a). After DMH1 treatment (4 to 6 hpf), larvae lacked expression of *syt1*, *elav1* and *six3/6* in the apical organ (Fig. 3b, c; Supplementary Table 16). However, there were RYamidergic neurons in the apical organ, although the nervous system around the prototroch was disorganised and less developed (Fig. 3b; Supplementary Table 17). Paradoxically, rBMP4 treatment did not result in a lack of neural structures, as expected if BMP signalling had an anti-neural role, as in vertebrates and arthropods. Instead, *elav1*, *syt1* and *six3/6* were expressed in the apical pole of rBMP4-treated larvae, which exhibited fewer RYamidergic neurons (Fig. 3c, e; Supplementary Tables 16, 17). Therefore, even though it is difficult to discern whether the defects in the larval nervous system are a direct consequence of dysregulating BMP signalling or a result of the overall loss of axial identities after DMH1 and rBMP4 treatments, our data support a neural-related role of the BMP signalling pathway by modulating neuronal genes in addition to its axial patterning function in *O. fusiformis*.

### The role of ERK1/2 and BMP signalling in DV patterning is conserved in Annelida

**Fig. 4:**
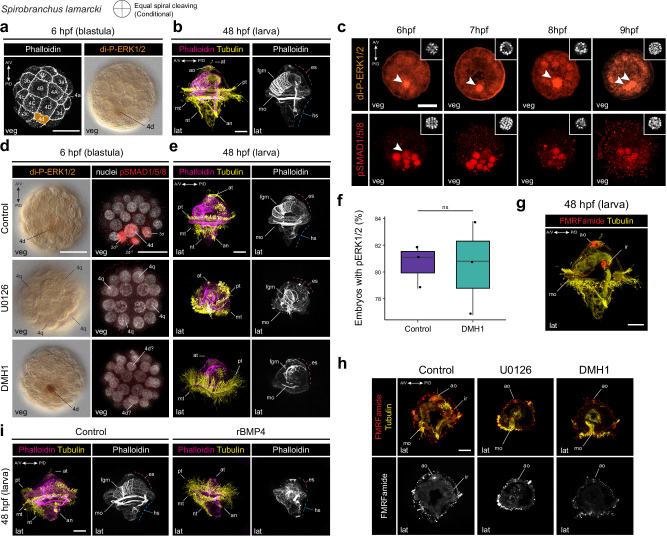
The role of ERK1/2 and BMP signalling in the annelid with equal spiral cleavage *Spirobranchus lamarcki.* **a** Z-stack projections and Differential Interference Contrast (DIC) of blastula (6 hpf) with cell membranes labelled with phalloidin (left) and anti-dp-ERK1/2 (right), which localises in the putative 4d cell. **b** Z-stack projections of a 48 hpf larva. Cilia (yellow) are labelled with tubulin, and F-actin (magenta/white) is labelled with phalloidin. **c** Z-stack projections of 6–9 hpf embryos stained against pERK1/2 and pSMAD1/5/8. The arrowhead points to the 4D cell and its putative descendants. **d** DIC images of control and U0126/DMH1-treated blastula (6 hpf) stained with dp-ERK1/2 and z-stack projections of control and U0126/DMH1-treated blastula (6 hpf) stained with DAPI (white) and pSMAD1/5/8 (red). **e** Z-stack projections of 48 hpf larvae developed from control and U0126/DMH1-treated embryos. Cilia (yellow) are labelled with tubulin, and F-actin (magenta/white) is labelled with phalloidin. **f** Boxplot showing the median percentage of embryos with pERK1/2 enrichment in the 4d in control (*n* = 3) and DMH1-treated (*n* = 3) embryos. There is not a statistically significant difference (*p*-value = 0.8323; Mantel-Haenszel chi-squared test with continuity correction). Boxplots representing the lower and upper quartiles, whiskers the minimum and maximum values. **g**, **h** Z-stack projections of wild-type (**g**) and control/treated (**h**) 48 hpf larvae labelled with FMRFamide (red) and tubulin (yellow) showing the presence of a dorsal neurosecretory ring of FMRFamide^+^ cells around the gut in the episphere in wild-type and control larvae that disappears after ERK1/2 and BMP inhibition. **i** Z-stack projections of 48 hpf larvae developed from control and rBMP4-treated embryos. Cilia (yellow) are labelled with tubulin, and F-actin (magenta/white) is labelled with phalloidin. Lateral views are with anteroventral to the left, ventral and apical views are with anteroventral to the top. For whole-mount in situ hybridisation and immunostaining, we show representatives of at least two independent experiments. Scale bars are 25 µm. an anus, ao apical organ, at apical tuft, es episphere, fgm foregut muscle, hs hyposphere, lat lateral, mo mouth, pt prototroch, mt metatroch, nt neurotroch, tt telotroch, veg vegetal. Source data are provided as a Source Data file.

The role of ERK1/2 and BMP signalling in setting the axial polarity in *O. fusiformis* markedly differs from the condition described in annelids with unequal spiral cleavage, such as *Chaetopterus pergamentaceus* and *Capitella teleta*, which use Activin/Nodal to set their DV axes^16,17,20^. To assess whether the axial patterning mechanisms found in *O. fusiformis* are conserved in other annelids with a similar equal mode of spiral cleavage, we investigated the annelid *Spirobranchus lamarcki*^38^, a member of Serpulidae that is more closely related to *C. teleta* and *Helobdella robusta*. After spiral cleavage and gastrulation, the embryos of *S. lamarcki* develop into a typical trochophore larva with an anterior episphere (i.e., head) and a posterior hyposphere (i.e., trunk) separated by two ciliary bands, the prototroch and the metatroch^39^ (Fig. 4a). In these larvae, the anus is not terminal but opens slightly dorsally at the end of the hyposphere (Fig. 4b). Similar to *O. fusiformis* and another serpulid, *Hydroides exogonus*^40^, the embryos of *S. lamarcki* have a single cell enriched with dp-ERK1/2, which we deemed the 4d micromere (Fig. 4a). Enrichment in a single cell continues as the embryo undergoes gastrulation, suggesting that, as in *O. fusiformis*, the specification of the 4d cell creates a cell division asymmetry^8^ (Fig. 4c). Likewise, as in *O. fusiformis*, pSMAD1/5/8 is asymmetrically enriched in the 4d cell, and one side of the embryo (the putative dorsoposterior side) at 6 hpf in the embryos of *S. lamarcki*, and this asymmetric localisation continues during the early stages of gastrulation (Fig. 4c). Interestingly, *S. lamarcki*, as *O. fusiformis*, but unlike other unequal spiral cleaving annelids, has retained a *chordin* ortholog in its genome. Therefore, distantly related annelids and molluscs with equal spiral cleavage appear to use homologous signalling pathways to define their axial patterning.

To further investigate the axial roles of the ERK1/2 and BMP signalling pathways in *S. lamarcki*, we treated embryos with U0126 (an ERK1/2 inhibitor), DMH1 (a BMP inhibitor), and rBMP4 (a BMP agonist) (Supplementary Fig. 4a–c). Treatment with U0126 blocked ERK1/2 and SMAD1/5/8 activation in *S. lamarcki* (Fig. 4d; Supplementary Tables 18, 19). However, the inhibition of the BMP signalling with DMH1 did not statistically affect the dpERK1/2 activation and the specification of the 4d organiser (Fig. 4d, f; Supplementary Tables 19, 20). In both cases, the inhibition of ERK1/2 and BMP signalling resulted in the loss of the hyposphere and the anus at 48 hpf, with DMH1-treated larvae exhibiting more marked anteroposterior elongation than those developing after inhibition of ERK1/2 (Fig. 4e; Supplementary Table 21). Notably, a bundle of FMRFamide-positive neurosecretory cells encircling the gut and located opposite to the mouth in the episphere (the putative dorsal side) disappeared after U0126 and DMH1 treatment (Fig. 4g, h), indicating that the axial patterning phenotypes are not solely restricted to the hyposphere but affect the larval body broadly. Treatment with rBMP4 led, however, to larvae with only a slightly reduced episphere, lacking a mouth and foregut muscles but presenting a dorsal anus and a ventral neurotroch (Fig. 4i; Supplementary Table 21). Accordingly, rBMP4 treatment did not result in ectopic pSMAD1/5/8 activation, even at high concentrations (Supplementary Fig. 4c; Supplementary Table 22), indicating species-specific sensitivities of the BMP receptor to its ligand in annelids. Therefore, ERK1/2 signalling establishes the bilateral polarity in *S. lamarcki*. Although a role in patterning the epi- and hyposphere cannot be completely ruled out, our findings are consistent with a role of the BMP pathway in patterning the DV axis in *S. lamarcki*, as in *O. fusiformis*, thereby indicating an ancestral role of this pathway in dorsoventral patterning in Annelida. Future analyses of gene expression of axial polarity markers will further clarify the precise axial functions of these signalling pathways.

### BMP signalling patterns the larval head along the DV axis in P. dumerilii

**Fig. 5:**
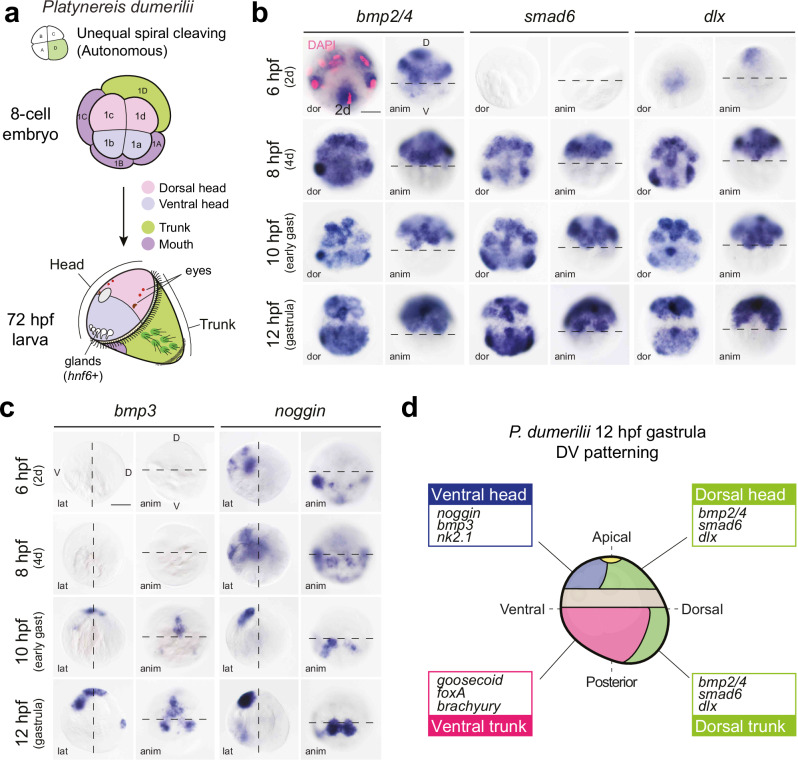
The dorsoventral embryonic patterning in *P. dumerilii.* **a** Diagram showing the origin of the dorsoventral structures from the 8-cell-stage embryo and their simplified fate map in the 72 hpf larva (see text for details). Three pairs of dorsal eyes and five ventral *hnf6*+ gland cells are shown. **b**, **c** Whole mount in situ hybridisation of the dorsoventral markers *bmp2/4*, *smad6*, *dlx*, *bmp3* and *noggin* at 6, 8, 10 and 12 hpf embryos. Dotted lines indicate the division between dorsal and ventral territories in lateral and animal/vegetal views. **d** Diagram of dorsoventral marker genes, and *goosecoid*, *foxA*, and *brachyury* expression domains in a 12 hpf gastrula stage. A ciliary ring (the prototroch; grey) separates the anterior head and the posterior trunk. See Supplementary Fig. 5 for additional expression data of dorsoventral marker genes. In all panels, lateral views are with ventral to the left and animal/vegetal views are with dorsal to the top. For whole-mount in situ hybridisation and immunostaining, we show representatives of at least two independent experiments. Scale bars are 50 µm. anim animal, D dorsal, dor dorsal, lat lateral, V ventral. Drawings are not to scale.

The ancestral role of BMP signalling in DV patterning suggests that the use of Activin/Nodal in DV specification evolved convergently in *Capitella teleta* and *Chaetopterus pergamentaceus*. To assess whether this represents a recurring feature among other annelids with unequal development, we investigated the role of BMP signalling in *P. dumerilii*, a well-established model annelid belonging to Errantia^41^, the sister clade to the group comprising *S. lamarcki* and *C. teleta* (Fig. 1b). Although BMP influences DV patterning in the 2-day-old larva in *P. dumerilii*^15^, it remains unclear whether this signalling is essential for DV polarity during embryogenesis. As in other annelids with unequal cleavage, the two larger animal micromeres at the 8-cell stage—1c and 1d—give rise to the dorsal head region, while the two smaller micromeres—1a and 1b—contribute to the ventral head region in *P. dumerilii*^42–44^ (Fig. 5a). In subsequent cell divisions, the largest macromere 1D generates two uniquely large blastomeres—2d and 4d—which demarcate the dorsal side of the trunk at an early developmental stage before eventually forming most of the larval trunk ecto- and mesoderm, respectively (Fig. 5a).

We therefore analysed the expression of core BMP pathway components and dorsoventral markers during cleavage (6 and 8 hpf, when the 2d and 4d blastomeres form, respectively), gastrulation (10 and 12 hpf, early and late gastrula, respectively), organogensis (18 hpf) and larval stages (24, 48, and 72 hpf, which correspond to early and late trochophore and nectochaete larva, respectively)^43,45^. Our expression profile analysis reveals that the genes *bmp2/4*, *smad6* (a putative BMP signalling antagonist^46^), and *dlx* (an alternative readout of BMP signalling^47^ given the lack of crossreactivity for the pSMAD1/5/8 antibody in *P. dumerilii*) start to be expressed from 6 to 8 hpf in dorsal cell lineages of the head and trunk. At these stages, *bmp2/4*, *smad6*, and *dlx* are only expressed in the 1c and 1d progeny (but not in the dorsal prototroch lineages) and later only in the progeny of 2d, including 2d^1^, 2d^2^, 2d^12^, and 2d^112^, as well as their progeny (Fig. 5b; Supplementary Fig. 5a, c). On the opposite side, the BMP antagonists *noggin* and *bmp3* are expressed ventrally in both the head and trunk (Fig. 5c; Supplementary Fig. 5b, d). Starting at 6 hpf, *noggin* is expressed in the 1a and 1b progeny in the head region (1a^1^ and 1b^1^, then 1a^11^ and 1b^11^, etc.), which correspond to the ventral cephaloblasts, and also in the trunk region in three blastomeres, most likely 2a, 2b and 2c. *Bmp3* is initially expressed at 10 hpf in 1n^111^ cells, and at 12 hpf in some progeny of 1a^112^ and 1b^112^, the ventral cephaloblasts. Their expression patterns correspond to those of known ventral head (e.g., *nk2.1*, *hnf6*) and trunk markers (e.g., *gsc*, *foxA*, and *bra*) (Supplementary Fig. 5e–f). As in *O. fusiformis*, *bmp5-8* shows an early ubiquitous expression that concentrates more on the dorsal side of the larva as development progresses (Supplementary Fig. 5g). Therefore, the expression of *bmp2/4* and putative BMP target genes on the dorsal side, in contrast to the ventral expression of genes encoding BMP antagonists (Fig. 5d), strongly suggests that the BMP pathway could play a role in DV axis specification during the unequal spiral cleavage in *P. dumerilii*.

**Fig. 6:**
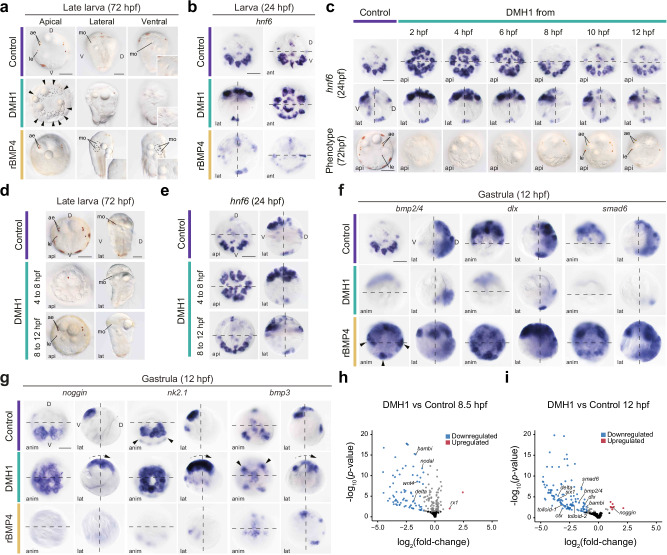
BMP controls the dorsoposterior axis of the head in *P. dumerilii.* **a** Differential Interference Contrast (DIC) image of control, DMH1, and rBMP4-treated 72 hpf larvae. Arrowheads point to the radialisation of the gland cells (dashed) after DMH1 treatment. Dashed circles show ectopic mouths after rBMP4 treatment. **b** Whole mount in situ hybridisation of the ventral marker *hnf6* in control, DMH1, and rBMP4-treated 24 hpf larvae. **c** Whole mount in situ hybridisation of *hnf6* at 24 hpf (two top rows) and Differential Interference Contrast (DIC) images at 72 hpf (bottom row) in controls (first column) and different windows of treatments with DMH1. **d**, **e** DIC images (**d**) and whole mount in situ hybridisation (**e**) of *hnf6* in control and DMH1-treated in windows that result in an abnormal dorsoventral axis (4 to 8 hpf treatment) and wild-type phenotype (8 to 12 hpf treatment). **f**, **g** Whole mount in situ hybridisation of dorsal (*bmp2/4*, *dlx* and *smad6*; **f**) and ventral (*noggin*, *nk2.1* and *bmp3*; **g**) markers in control, DMH1, and rBMP4-treated 12 hpf gastrulae, respectively. **h**, **i** Volcano plots (two-sided Wald test) depicting differentially expressed genes at 8.5 and 12 hpf in DMH1 vs control embryos treated from 4 to 8 hpf. In (**a**–**g**), dorsal is to the top in apical views, ventral to the left in lateral views and anterior to the top in ventral views. Animal views in (**f**, **g**) are at the level of the progeny of the first quartet micromeres, with the 1c/1d to the top. Dotted lines separate the dorsal and ventral hemispheres. For whole-mount in situ hybridisation and immunostaining, we show representatives of at least two independent experiments. Scale bars are 50 µm. ae adult eyes, anim animal, api apical, D dorsal, lat lateral, le larval eyes, mo mouth, V ventral. Source data are provided as a Source Data file.

To investigate this hypothesis, we first inhibited and overactivated BMP signalling by continuous treatment with DMH1 and rBMP4, respectively, from 2 hpf (Supplementary Fig. 6; Supplementary Table 23). DMH1 treatment induced the ventralisation of *P. dumerilii* larvae at 72 hpf, which lacked both larval and adult eyes, the dorsoanterior ciliary band, and the nephroblasts (Fig. 6a). These ventralised larvae exhibited radialisation of gland cells expressing *hnf6*, a gene usually restricted to the ventral region in wild-type larvae (Fig. 6b). To refine the temporal window of BMP signalling activity essential for DV patterning, we conducted staggered DMH1 treatments between 2 and 12 hpf. This revealed a critical period of BMP signalling activity around 6 hpf, when the embryo consists of ~16 cells (Fig. 6c). Indeed, inhibition of the BMP signalling from 4 to 8 hpf (8 to ~66-cell stages) led to larvae with ventralised heads, as in continuous DMH1 treatment (Fig. 6d, e; Supplementary Table 23). The ventralisation induced by BMP signalling inhibition could already be observed at 12 hpf, with the loss of dorsal markers, including *bmp2/4*, *smad6*, and *dlx*, and the ectopic expression of ventral head genes such as *noggin*, *bmp3*, and *nk2.1* throughout the entire head region (Fig. 6f, g). Nephroblasts and the akrotroch (a dorsal circlet of cilia in the head), originally formed from a pair of large cells in the dorsal head^44,48,49^, also disappeared, as revealed by the absence of expression of *fzCRD1* and *tektin2*, respectively (Supplementary Fig. 7a, b).

In contrast with the DMH1 treatment, rBMP4 exposure induced the incomplete dorsalisation of larvae at 72 hpf, which lost the ventral gland cells in 24 and 72 hpf (Fig. 6d, e; Supplementary Table 23), and did not express ventral markers (*noggin* and *nk2.1*). In addition, overactivation of the BMP signalling led to the ectopic expression of dorsal genes (*bmp2/4*, *dlx*, and *smad6*) into the ventral head territory at 12 hpf (Fig. 6f, g). Unexpectedly, both DMH1 and rBMP4-treated larvae showed only mild phenotypes in the trunk region. BMP signalling inhibition resulted in a slightly bent trunk at 72 hpf (Fig. 6a, d), reminiscent of the phenotype observed in *C. teleta* after BMP inhibition^17,50^. The trunk expression of dorsal markers in the 2d (*bmp2/4*, *dlx*, *smad6*) and 4d progeny (*brachyury*) was also reduced (Fig. 6f; Supplementary Fig. 7c), while the ventral trunk markers *foxA* and *goosecoid* showed a dorsal expansion in DMH1-treated 12 hpf embryos (Supplementary Fig. 7c). Ectopic BMP activation affected the patterning of the trunk even less, except for *goosecoid* expression and the alteration of the stomodaeum anlage (Fig. 6a; Supplementary Fig. 7d). Altogether, our findings demonstrate that BMP signalling is essential for DV patterning during early spiral cleavage in *P. dumerilii*. However, the head and trunk respond differently to this morphogen, with BMP signalling required to specify dorsal cell lineages in the head, but signs of dorsoventral patterning remain visible in the trunk after inhibiting this pathway.

To capture the early and late transcriptional responses induced by BMP signalling during head DV patterning, we performed comparative bulk transcriptomics analysis of control and DMH1-treated embryos (from 4 to 8 hpf) at four developmental time points (8.5, 12, 18, and 24 hpf) (Supplementary Fig. 7e, f; Supplementary Tables 24–31). At 8.5 and 12 hpf, genes orthologous to the vertebrate BMP synexpression group^51^, including *vent, bambi, smad6*, *dlx, tbx3*, and *id4*, as well as transcription factors like *hes-related* factors, *hmx3*, and *bsh*, and similar to *O. fusiformis*, the ligand *delta* were amongst the significantly downregulated genes in DMH1-treated embryos (Fig. 6h; Supplementary Tables 24, 26). In contrast, ventral genes such as *nk2.1*, *noggin* and *bmp3*, which expand their expression after DMH1 treatment (Fig. 6g), were significantly upregulated at these time points (Supplementary Tables 25, 27), supporting the notion that in the absence of BMP signalling, the head ventralisation is the default state for the ‘dorsal’ micromeres.

Notably, genes like *smad6, dlx, bambi, tbx3*, and *vent* were downregulated in both embryonic and larval stages, while *nk2.1* was upregulated (Fig. 6h, i; Supplementary Fig. 7g, h and Supplementary Tables 24–31). The fact that the antagonist *smad6* is a downstream target of BMP signalling suggests the existence of autoregulatory feedback loops that might help restrict the pathway activity to the dorsal head progenitors. Remarkably, only *delta* and *dlx* were shared downregulated genes between the early DMH1-treated embryos of *O. fusiformis* (6 hpf) and *P. dumerilii* (8.5 hpf). In the later larval stages, genes such as *bsh, gcm, six1*, *vsx2*, and *gata1/2/*3 were amongst the significantly downregulated genes, whereas *hnf6* and *ptf1* were among the upregulated genes (Supplementary Fig. 7g, h; Supplementary Tables 28–31), supporting their roles in the development of the dorsal and ventral head features, like the dorsoanterior brain region and the ventral gland cells, respectively. Therefore, our results demonstrate that a short early BMP signalling event is sufficient to establish a transcriptional DV asymmetry in the head of the *P. dumerilii* embryo. However, the transcriptional response after BMP signalling in *P. dumerilii* differs considerably from that observed in *O. fusiformis*, at least in its initial stages.

### The 2d blastomere is the head DV organiser in P. dumerilii

**Fig. 7:**
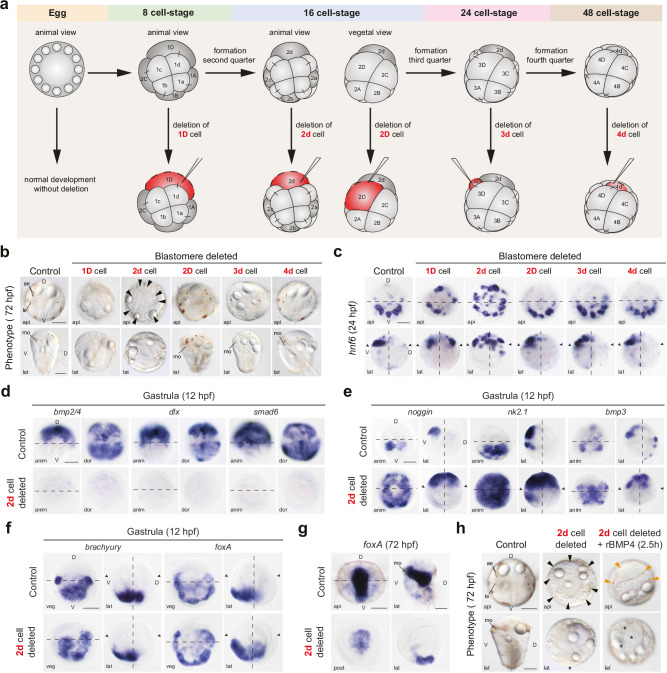
2d functions as the head embryonic organiser in *P. dumerilii.* **a** Diagram of the blastomere ablations (red) at different cleavage stages. Ablated cells (1D is the precursor cell of 2d and 2D; 2D will bud off 3D and 4D) are indicated in red. **b** Differential Interference Contrast (DIC) images of 72 hpf larvae resulting from the blastomere ablations. Note the absence of bilateral eyes and the radialisation of the larval glands (arrowheads) in comparison with controls. **c** Whole mount in situ hybridisation of *hnf6* in larvae (24 hpf) after blastomere ablations. **d**–**f** Whole mount in situ hybridisation of dorsoventral markers in controls and embryos after 2d-cell ablations in 12 hpf embryos. Ablation of the 2d cell phenocopies DMH1 treatments (compare to Fig. 6f–g). **g** Whole mount in situ hybridisation of *foxA* in larvae (72 hpf) after 2d ablation. **h** DIC images of larvae rescued with rBMP4 after the ablation of the 2d cell. At 72 hpf, larvae after 2d cell deletion exhibit the radialisation of gland cells (black arrowheads) and loss of eyes. Rescue with rBMP4 results in a reduction of gland cells and the retention of two pairs of adult eyes (orange arrowheads). In (**b**–**h**), dorsal is to the top in apical/animal views, and ventral to the left in lateral views. Black arrowheads in (**c**, **e**, **f**) indicate the approximate position of the developing prototroch. Dotted lines separate the dorsal and ventral hemispheres. We show representatives of at least two independent experiments. Scale bars are 50 µm. ae adult eyes, anim animal, api apical, D dorsal, lat lateral, le larval eyes, mo mouth, V ventral.

In the unequal cleaver annelid *C. teleta*, the 2d cell functions as a head axial organiser^16,17,52^. Given the strong expression of *bmp2/4* in the 2d cell (Fig. 5b) during the critical timing of BMP signalling, we hypothesised that a BMP-mediated signal from this cell could induce the BMP-mediated DV patterning of the head. To test this, we obtained batches of embryos at various cleavage stages and ablated a single D quadrant blastomere from each. In particular, we targeted the 2d cell, as well as its progenitor, the 1D blastomere, its sister cell, the 2D blastomere, and its early progeny, the 3d and 4d cells (Fig. 7a). As expected, only the ablation of the 2d cell and its progenitor phenocopied the DV patterning and differentiation defects observed in the head observed in DMH1-treated embryos (Fig. 6a, b; Fig. 7b, c and Supplementary Table 32). 2d-ablated embryos exhibit an epi- and hyposphere separated by a prototroch. However, 2d ablation impaired axial elongation, unlike after 2d, 3d, or 4d cell ablation (Fig. 7b). In 2d-deleted embryos, the expression of the dorsal markers *bmp2/4*, *dlx*, and *smad6* was abolished, whereas the ventral markers *noggin*, *bmp3*, and *nk2.1* expanded dorsally in the head (Fig. 7d, e). The ablation of the 2d cell also abolished the expression of *bmp2/4*, *dlx*, and *smad6* in the trunk (Fig. 7d), suggesting that the dorsal trunk domain expressing these genes derives directly from the 2d cell.

However, while the expression of *brachyury* and *foxA* was mostly retained in the 2d deleted embryo at 12 hpf, *foxA* expression and mouth position were strongly affected in the 2d deleted 72 hpf larvae (Fig. 7f, g). Notably, exposure to rBMP4 protein partially rescued the ventralised phenotype after 2d ablation (Fig. 7h). Therefore, in the early embryo of *P. dumerilli*, the 2d cell, expressing *bmp2/4* from 6 hpf, acts as an embryonic organiser responsible for the DV patterning of the head. This organiser, via BMP signalling, activates the expression of dorsal head genes, including *bmp2/4* itself, and restricts ventral head genes such as *noggin* or *bmp3* in the ventral head region. After differentiation, these two regions along the DV axis will give rise to gland cells on the ventral side, and adult and larval eyes on the dorsal side of the larval head (Supplementary Fig. 7i).

### Activin/Nodal affects dorsoposterior development in O. fusiformis

**Fig. 8:**
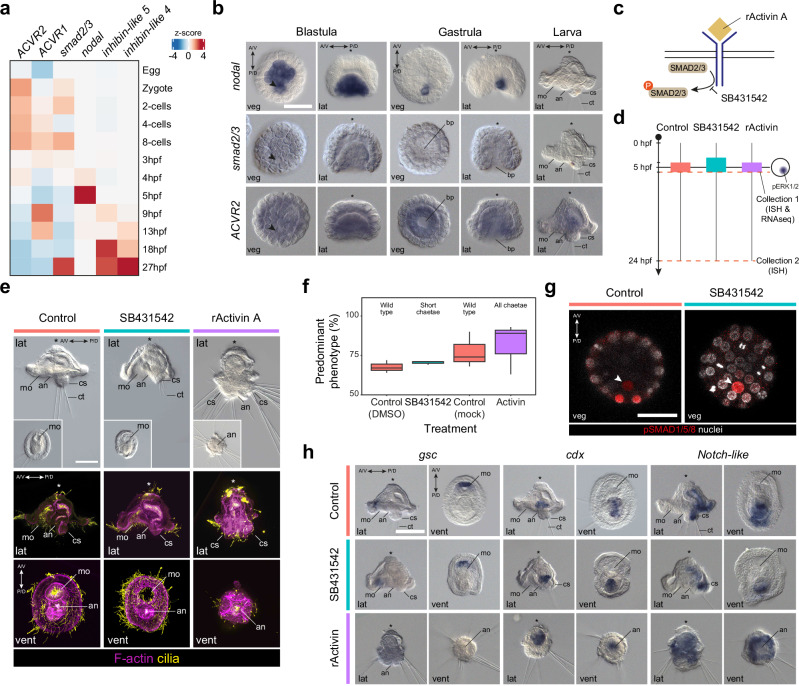
Activin/Nodal plays a role in dorsoposterior specification in *O. fusiformis* independently of pSMAD1/5/8. **a** Heatmap indicating the relative expression of core components of the Activin/Nodal pathway during the development of *O. fusiformis*. **b** Whole mount in situ hybridisation of the ligand *nodal*, secondary messenger *smad2/*3, and the activin receptor *ACVR2* in blastula (6 hpf), gastrula (9 hpf), and larval stages (24 hpf). **c** Simplified schematic of the Activin/Nodal pathway and mode of action of SB431542 and recombinant Activin A (rActivin A). **d** Schematic representation of the experimental design for the treatment windows and sample collections. **e** Differential interference contrast (top) and z-stack projections of control and treated larvae developed from embryos treated with SB431542 (3 to 6 hpf) and rActivin A (4 to 6 hpf). Insets in the first row are ventral views. Cilia (yellow) are labelled with tubulin, and F-actin (magenta) is labelled with phalloidin. **f** Box plots indicating the median percentage of embryos exhibiting the described phenotypes for each treatment. DMSO- and mock-control larvae have wild-type morphology. SB431542-treated larvae have a mild reduction of the dorsoposterior region, and rActivin-treated larvae have ectopic dorsoposterior tissue. Box plots representing the lower and upper quartiles, whiskers the minimum and maximum values (*n* = 3, per treatment). **g** Z-stack projections of blastula (6 hpf) treated with control and SB431542/rActivin A stained against pSMAD1/5/8. **h** Whole-mount in situ hybridisation of anterior (*gsc*) and posterior (*cdx*) marker genes in control and treated larvae developed from SB431542 and rActivin A-treated embryos. In all panels, the arrowheads point to the 4d organiser, and the asterisks mark the animal/apical pole. Lateral views are with anteroventral to the left, ventral and apical views are with anteroventral to the top. For whole-mount in situ hybridisation and immunostaining, we show representatives of at least two independent experiments. Scale bars are 50 µm. an anus, bp blastopore, ch chatae, cs chaetal sac, lat lateral, mo mouth, pt prototroch, veg vegetal, vent ventral. Source data are provided as a Source Data file.

The transition to a DV specification relying on Activin/Nodal signalling in some annelids with unequal spiral cleavage raises the question of the ancestral role of this pathway in annelids. To address this, we first explored the Activin/Nodal pathway in *O. fusiformis*. This annelid has three Activin/Nodal/inhibin ligands—*inhibin-like 4*, *inhibin-like 5*, and *Nodal*—and two activin receptors—*ACVR1* and *ACVR2*^25^. While *inhibin* ligands are mainly expressed in late development, *Nodal* expression peaks at 5 hpf, when it is strongly detected in the gastral plate, including the embryonic organiser (Fig. 8a, b). With gastrulation, *Nodal* becomes expressed solely in the embryonic organiser (Fig. 8b). At these stages, the *ACVR2* receptor and the secondary messenger *smad2/3* are weakly but ubiquitously expressed (Fig. 8b).

To uncover the role of Activin/Nodal signalling, we treated embryos of *O. fusiformis* with the specific Nodal inhibitor SB431542 and recombinant Activin-A at the time of axial patterning (Fig. 8c, d; Supplementary Fig. 2c, d; Supplementary Tables 3–8). Treatment with SB431542 resulted in smaller larvae (70%) with a smaller chaetal sac and shorter chaetae (Fig. 8e, f), which is mildly reminiscent of the effects of BMP signalling inhibition (Fig. 2c). However, SB431542 prevented neither the formation of the organiser nor the activation of pSMAD1/5/8 (Fig. 8g; Supplementary Table 3), and the treated larvae retained a normal expression of oral and hindgut markers, as well as expression (albeit reduced) of the dorsal marker *Notch-like* (Fig. 8h) and the neuronal gene *syt1* (Fig. 3d). Interestingly, after rActivin A treatment, larvae phenocopied the rBMP4-treated larvae, exhibiting ectopic chaetal sacs and loss of *gsc* expression (Fig. 3e; Fig. 8e, h and Supplementary Table 4–8), but not widespread activation of pSMAD1/5/8 (Supplementary Fig. 2d). Therefore, Activin/Nodal is unnecessary to establish the initial DV pattern in *O. fusiformis*. Still, it seems to contribute to its subsequent development, as evidenced by phenotypic similarities following ectopic activation of Activin/Nodal and BMP signalling.

To discern if the effect of Activin/Nodal on DV development relies on similar genes downstream of BMP signalling, we performed comparative transcriptomic profiling of control and SB431542 and rActivin A-treated embryos (Supplementary Fig. 8a, b). We found 27 upregulated and 41 downregulated genes in SB431542-treated embryos compared to the controls, which were primarily enriched for GO term categories involved in development and body patterning (Supplementary Fig. 8e; Supplementary Tables 9, 33, 34). Surprisingly, SB431542 and DMH1-treated embryos did not share any downstream genes despite leading to comparable (but weaker in SB431542) phenotypes in the larvae (Supplementary Fig. 8c; Supplementary Tables 9–11).

Amongst the downstream genes, we focused on the three transcription factors with a known developmental role: *otx*, expressed in precursors of the ciliary band and later the gut^23^; *hb*, expressed in the gastral plate and endoderm; and *prospero*, expressed in the ectoderm and oesophagus (Supplementary Fig. 8d, f). In all cases, their expression was dramatically downregulated in the blastula after SB431542 treatment (Supplementary Fig. 8g; Supplementary Table 11). Unlike SB431542, treatment with rActivin A led to many differentially expressed genes enriched for developmental GO terms, including genes expressed in the apical organ (e.g., *six3/6* and *rx1*) and internalised cells (e.g., *zag1*) (Supplementary Fig. 9a–c; Supplementary Table 12). However, only a small fraction of differentially expressed genes (~3%) were shared with those affected by rBMP4 treatment (Supplementary Fig. 9d; Supplementary Tables 36, 37). This included five downregulated (*osr*, *foxG*, *foxA*, *fojo*, and *cbc1*) and two upregulated (*wnt5* and *msx*) genes (Supplementary Fig. 9e–g; Supplementary Table 11). Altogether, our findings show that the BMP and Activin/Nodal pathways largely regulate distinct sets of genes, suggesting that the effect of Activin/Nodal in dorsoventral development is likely secondary and/or indirect in *O. fusiformis*.

### The Activin/Nodal pathway does not regulate DV patterning in P. dumerilii

**Fig. 9:**
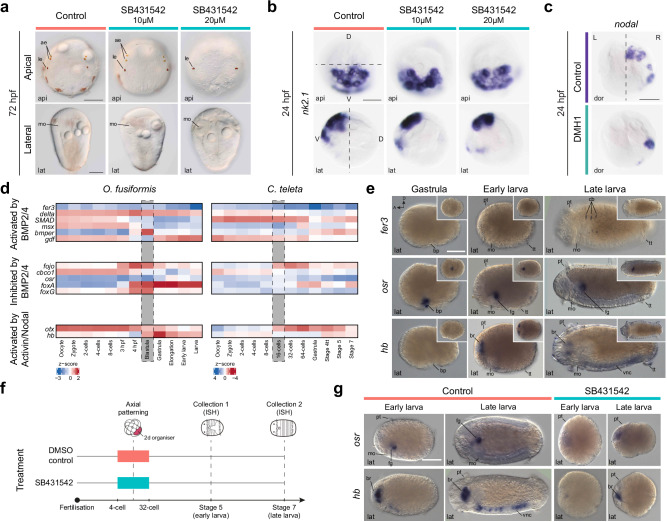
Activin/Nodal does not control dorsoventral polarity in *P. dumerilii* and uses a different downstream network in *C. teleta.* **a** Differential interference contrast images of control and SB431542 larvae at 72 hpf in *P. dumerilii*. **b** Whole mount in situ hybridisation of the ventral marker *nk2.1* in control and SB431542 24 hpf larvae of *P. dumerilii*. The dashed line marks the boundary between the dorsal (D) and ventral (V) sides. **c** Whole mount in situ hybridisation of *nodal* in control and DMH1-treated 24 hpf larvae in *P. dumerilii*. The dashed line marks the boundary between the left (L) and right (R) sides. **d** Heatmaps depicting the relative expression of differentially expressed candidate genes in *O. fusiformis* and their corresponding one-to-one orthologs in *C. teleta* during embryogenesis. **e** Whole mount in situ hybridisation of three candidate differentially expressed genes in *O. fusiformis* during the development of *C. teleta* at the gastrula, stage 5 and stage 7 larvae. Insets are ventral views. **f** Schematic diagram of the drug treatment in *C. teleta*. (**g**) Whole mount in situ hybridisation of the candidate genes *osr* (inhibited by the BMP pathway) and *hb* (activated by Activin/Nodal) in control and SB431542 stage 5 and stage 7 *C. teleta* larvae. Lateral views are with anteroventral to the left, and apical views are with dorsal to the top. For whole-mount in situ hybridisation and immunostaining, we show representatives of at least two independent experiments. Scale bars are 50 µm. an anus, api apical, bp blastopore, br brain, cb chaetoblast, dor dorsal, fg foregut, lat lateral, mo mouth, pt prototroch, tt telotroch, vnc ventral nerve cord. Source data are provided as a Source Data file.

In annelids with unequal development, such as *C. teleta* and *C. pergamentaceus*^16,17,20^, a reduced role of the BMP signalling in DV patterning co-occurs with a compensatory and more prominent axial patterning function of the Activin/Nodal pathway. To determine whether this shift also occurs in *P. dumerilii*, we continuously treated embryos with the inhibitor SB431542 at concentrations of 10 and 20 µM from 2 hpf (Fig. 9a, b). At 72 hpf, treated larvae displayed normal morphology, with a correct DV patterning of both head and trunk (Fig. 9a). However, we noted the absence of adult eyes at 20 µM in the dorsal head region, although these larvae still formed larval eyes in what appeared a left-right symmetrical phenotype (Fig. 9a). Accordingly, the expression of the ventral head marker *nk2.1*, which expands dorsally after BMP inhibition (Fig. 6g), showed a normal expression in SB431542-treated embryos (Fig. 9b), further supporting that the DV patterning of the head is not affected after inhibiting the Activin/Nodal signalling pathway, unlike in *C. teleta*^16,17^. Interestingly, Nodal showed a left-right asymmetric expression in the 24 hpf larva of *P. dumerilii*, when it is expressed on the right side and partially depends on normal BMP signalling (Fig. 9c). Therefore, unlike *O. fusiformis* and *C. teleta*, the Activin/Nodal pathway is not required for DV patterning in *P. dumerilii*. However, as in *O. fusiformis*, it might contribute to the formation of specific dorsoventrally positioned head structures, such as the eyes. Further work is needed to determine whether this is more broadly related to a potential downstream function of Nodal in left-right development.

### DV development relies on different downstream genes in O. fusiformis and C. teleta

An evolutionary transition to Activin/Nodal-mediated DV patterning might have rewired this signalling pathway to regulate axial polarity genes that were originally downstream of BMP signalling. To assess this hypothesis, we first compared the temporal expression profile of the 13 one-to-one orthologous developmental genes between *O. fusiformis* and *C. teleta*^19^ that are downregulated by DMH1 and SB431542 and upregulated by rBMP4 in *O. fusiformis*. None of them shows a pattern indicating upregulation after the organising activity of Nodal at the 16-cell stage in *C. teleta*^52^ (Fig. 9d), except for *otx* and *fojo*, which are broadly expressed in the animal pole at that time^53,54^. Considering that transcriptomic dynamics might not entirely reflect the spatiotemporal expression of these genes, we investigated the expression in *C. teleta* of three genes under different regulatory conditions in *O. fusiformis*: *fer3*, activated by BMP; *osr*, inhibited by BMP; and *hb*, activated by Nodal. Like in *O. fusiformis*, *fer3* is expressed in the chaetoblasts, *osr* marks the boundary between the foregut and the midgut, and *hb* is expressed in the developing brain and ventral nerve cord in *C. teleta* (Fig. 9e). However, inhibition of DV polarity with SB431542^17^, which also affects trunk elongation in *C. teleta* (Supplementary Table 38), restricted, but did not eliminate, the expression of *osr* and *hb* to the anterior body (Fig. 9f, g), suggesting that these are not direct downstream genes of Activin/Nodal in this annelid. Therefore, our data indicate that changes in upstream signalling specifying the DV axis rewired downstream effector genes between *O. fusiformis* and *C. teleta*.

## Discussion

**Fig. 10:**
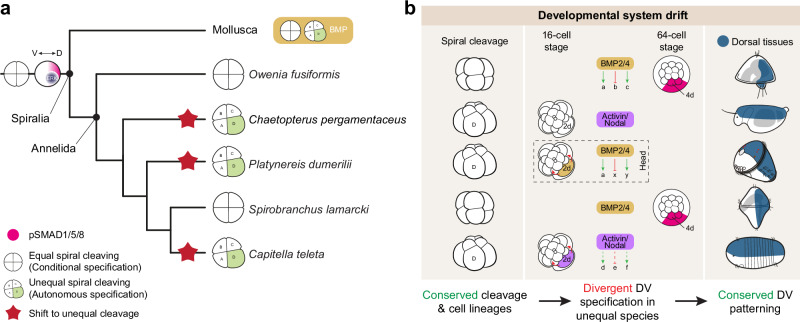
The ancestral role of BMP in Spiralia and the evolution of developmental system drift in dorsoventral patterning. **a** Evolutionary scenario for the developmental role of the BMP pathway in axial patterning in Mollusca and Annelida and its last common ancestor (assumed to be the last common ancestor to Lophotrochozoa based on current phylogenies). Given BMP’s role in DV patterning and the presence of a dorsoventral gradient of pSMAD1/5/8 in molluscs and annelids with equal spiral cleavage, an ERK1/2-BMP signalling axis defining and patterning the posterodorsal side of the embryo is likely ancestral to Spiralia and Annelida, but absent in annelid species that have transitioned to an unequal mode of spiral cleavage (red stars). **b** Schematic drawings representing the cellular and molecular specification and patterning of the dorsoventral axis in the studied annelid species. All these annelids exhibit a conserved and homologous cleavage programme, cell lineages and dorsoventral axes. However, the cells and molecular underpinnings can diverge profoundly, a phenomenon termed developmental system drift, in species that have transitioned to unequal development. While annelids with equal spiral cleavage employ ERK1/2 upstream of BMP to specify the bilateral symmetry and dorsoventral axis, respectively, species with autonomous specification of the D quadrant define the axial polarity and dorsoventral axis around the 16-cell stage, with a prominent role for the 2d blastomere, at least in *P. dumerilii* and *C. teleta*. Moreover, divergent upstream regulators trigger dorsoventral specification, with Activin/Nodal as the primary regulator in *C. pergamentaceus* and *C. teleta*, and the BMP pathway driving dorsoventral patterning mainly in the head in *P. dumerilii*. The divergence in upstream regulators is also concomitant with divergence in downstream effector genes. Drawings are not to scale. Schematics of late larval stages are oriented with anteroventral to the left.

Combining a multispecies approach with comparative transcriptomics, blastomere deletions, and pharmacological inhibition of the ERK, BMP, and Activin/Nodal signalling pathways, our study clearly reveals a conserved use of the ERK1/2 and BMP pathways to specify and pattern, respectively, the DV axis in distantly related annelids with equal spiral cleavage (Fig. 10a). This is similar to other spiralians like molluscs^12,13,24^, particularly the gastropod with conditional spiral cleavage *Lottia peitaihoensis*, which also uses FGFR, ERK1/2 and BMP to define the embryonic organiser and bilateral symmetry^12,24^. In contrast, the anti-neural role of the BMP pathway, as seen in vertebrates^3^ and arthropods^4^, remains unclear across spiralians. In some molluscs, BMP promotes neurogenesis^12,13^, while in others (e.g., *Crepidula fornicata*^14^) and in annelids (e.g., *C. teleta*, *Helobdella* and *P. dumerilii*), this signalling pathway acts locally to set up the DV coordinates in the neuroectoderm^15,18,50,55^. In *O. fusiformis*, the inhibition of BMP signalling disrupts nervous system patterning and downregulates the expression of neural-related genes (*elav1*, *syt1*, *six3/6*), but neuropeptidergic cells (as indicated by immunoreactivity against RYamide) remain present, altogether suggesting a possible pro-neurogenic role, as in some molluscs^12,13^. Therefore, an early role of the BMP signalling pathway in establishing the DV axis is likely ancestral to Spiralia, as in most other bilaterians^5^ (Figs. 1a, 10a). Yet, unlike in other non-spiralian lineages, this axial function occurs downstream of the FGFR-ERK1/2-mediated patterning system and is likely decoupled from a potential neurogenic role later in spiralian development.

Similarly, early axial patterning appears to be decoupled from the direct lineage contributions of the initial blastomere quadrants. In annelids, the specification of the blastomere from the D quadrant that acts as embryonic organiser has shifted from the 4d cell in equal cleavers (*O. fusiformis* and *S. lamarcki*) to the 2d blastomere in unequal cleavers (*C. teleta* and *P. dumerilii*). In *O. fusiformis*, the A–D quadrants have similar anteroventral potential at the blastula stage^8^. In addition, *bmp2/4*, which is expressed in the B quadrant, does not become radially expressed after pERK1/2 inhibition, indicating that there is no fate transition from D to B and that, therefore, 4d specification defines the quadrant identities and territories. Instead, pERK1/2 inhibition downregulates *bmp2/4* expression, preventing the activation of pSMAD1/5/8 and the patterning of the dorsoposterior side, which has contributions of both the C and the D quadrants. The lack of a dorsoventral gradient of pSMAD1/5/8 activity, thereafter, leads to the loss of dorsal and some posterior tissues in annelids (e.g., *Owenia*^8^ and *S. lamarcki*) and molluscs (e.g., *Lottia*^12^), presumably by disrupting a D-lineage specific regulation of axial growth^56^, but not of bilateral symmetry, as the 4d cell (that forms the hindgut and trunk mesoderm) marks the posterior body region. This loss of mainly dorsoposterior ectodermal derivatives mirrors the defects in the trunk in the unequal cleaving annelid *C. teleta* after the inhibition of Nodal/Activin^16^. Indeed, the striking phenotypic differences of losing the 2d organising signal between *C. teleta* and *P. dumerlii*, might relate to the little contribution of the 4d cell to trunk formation in *C. teleta*^57^ compared to its major role in other annelids and molluscs with both equal and unequal spiral cleavage^58^. Thus, disruption of the organising 2d signal results in similar dorsoventral phenotypes in the head in both *C. teleta* and *P. dumerilli*, but only in a major reduction of the trunk in *C. teleta* because, in *P. dumerilii*, 4d contributes to the anteroposterior elongation of the trunk and dorsoventral migration leading to the ventral midline formation^59^. Therefore, dorsoventral patterning does not necessarily rely on specific contributions of blastomere descendants, but once the major event of quadrant specification occurs, a whole embryonic region responds to a localised signal event that results in the correct formation of the dorsoposterior tissue.

An ancestral FGFR-ERK1/2-BMP dorsoventral patterning implies that distantly related annelids with unequal cleavage and autonomous specification of the D quadrant have independently diverged to use Activin/Nodal for DV specification or restricted BMP signalling to DV patterning in only a subset of tissues, such as the head^55^ or the local mid-line ectoderm^18,55^. Importantly, in both *C. teleta*^16,17,52^ and *P. dumerilii*, the same blastomere, the 2d cell, exerts this early axial organising role, albeit through Activin/Nodal and BMP signalling, respectively (Fig. 10b). Yet, BMP signalling seems to promote the establishment of the 1d micromere identity at the expense of the 1b micromeres in both species^55^. Given the conservation of cell lineages and fate maps across annelids and spiralians, a homologous body region (i.e., the DV tissue) is thus specified through different genetic and developmental mechanisms in annelids, a phenomenon called developmental system drift (DSD)^60,61^. DSD is widespread yet poorly understood in animals and reflects the variability of embryogenesis, gene regulatory networks, and morphogenic events^60^. Not surprisingly, DSD was previously invoked regarding DV specification among spiralians^9,16^, given the dramatically different role of BMP in DV specification between molluscs and autonomous annelids. Our study refines this scenario, indicating that DSD in DV patterning co-occurs with transitions to unequal spiral cleavage in annelids (Fig. 10b) and possibly in some molluscan lineages (e.g., *Crepidula fornicata*).

How could DSD occur, and what are its implications at the gene regulatory level? In *O. fusiformis*, Activin/Nodal influences DV axis development, a condition reminiscent of deuterostomes^62–64^, in an ERK1/2-independent manner (Fig. 10b). A transition to unequal cleavage and an autonomous specification of some axial identities through the asymmetric inheritance of maternal determinants^65^ bypasses the axial patterning role of ERK1/2, thereby eliminating the upstream regulator of the BMP signalling to specify the DV axis. This might favour the Activin/Nodal pathway gaining a more predominant role in DV patterning in some annelids, while, through yet-unknown maternal cues, repurposing BMP signalling predominantly for anterior/head neuroectodermal regionalisation in others^55^. Notably, our transcriptomic data in *O. fusiformis* and *P. dumerilii*, and comparisons with *C. teleta*, suggest that divergence in DV patterning upstream regulators likely involves significantly different effector genes, even when the cells contributing to each body region are conserved. This indicates that DSD does not rewire upstream signalling to activate a conserved downstream cascade but likely implies recruiting different networks for the specification of the DV axis (Fig. 10b). Therefore, crucial pathways involved in axial polarity can evolve more quickly than predicted, which, given the current uncertainties in the interrelationships of Spiralia^66^, calls for expanding the taxon sampling in developmental studies across and within spiralian phyla to better identify conserved and derived traits in body patterning. Altogether, our work addresses current questions about the ancestral role of the BMP signalling pathway in DV axis specification in Annelida and Spiralia, providing a conceptual framework and tractable systems to investigate the evolutionary and developmental mechanisms that generate DSD during animal embryogenesis.

## Methods

### Animal husbandry and specimen collections

Adults, embryos, and larvae of *O. fusiformis*, *P. dumerilii*, *C. teleta* and *S. lamarcki* were collected as described before^33,39,48,67,68^. Embryos of *O. fusiformis* and *P. dumerilii* used for RNA sequencing were snap-frozen in liquid nitrogen and stored at –80 °C. Embryos and larvae of *O. fusiformis*, *S. lamarcki* and *C. teleta* used for other analyses were fixed in 4% paraformaldehyde in artificial sea water (ASW), and stored in either 1x phosphate-buffered saline (PBS) with sodium azide at 4 °C or methanol at –20 °C. Embryos and larvae of *P. dumerilii* for whole-mount in situ hybridisation were fixed in 4% paraformaldehyde in PBS for one hour at room temperature and stored in methanol at –20 °C.

### Drug treatments

U0126 (Promega; Cat No.: V1121 or Merck-Sigma; Cat No.: 19–147), DMH1 (Merck-Sigma; Cat No.: D8946) and SB431542 hydrate (Merck-Sigma; Cat No.: S4317) 10 mM drug stocks were made in dimethyl sulfoxide (DMSO). Stocks of 100 μg/ml recombinant zebrafish BMP4 protein for *O. fusiformis* (R&D; Cat No.: 1128-BM), recombinant human BMP4/BMP7 heterodimer for *P. dumerilii* (R&D; Cat No.: 3727-BP), and recombinant human/mouse/rat Activin A protein (R&D; Cat No.: 338-AC) were made in 4 mM HCl + 0.1% BSA and stored at –80 °C. Working solutions of the treatments were prepared in ASW to the desired concentrations, using equivalent volumes of solvents (DMSO or 4 mM HCl + 0.1% BSA) as negative controls.

For *O. fusiformis*, we used a range of concentrations of DMH1 and SB431542, from 1 μM to 100 μM (final working concentration 20 μM and 40 μM, respectively), of rBMP4 from 75 ng/μl to 2000 ng/μl (final working concentration 1000 ng/μl), and rActivin A from 10 ng/μl to 1000 ng/μl (final working concentration 75 ng/μl). Initial windows encompassed a continuous treatment of 0.5 hpf–24 hpf, as well as shorter windows of 0.5–6 hpf and 6–24 hpf. After obtaining replicable phenotypes within the 0.5–6 hpf window, and based on pSMAD1/5/8 immunostaining, we narrowed the exposure window to 4–6 hpf for all treatments, except SB431542 (from 3 to 6 hpf) (Supplementary Fig. 2). We fixed embryos at 6 hpf to assess pSMAD1/5/8 enrichment and gene expression, or 24 hpf to evaluate morphological outcomes and gene expression. U0126 was used at 10 μM for 0.5–6 hpf or 4–6 hpf, as previously reported^8^.

For *S. lamarcki*, we used a range of concentrations of DMH1 and U0126, from 1 to 50 or 100 μM (final working concentration 10 μM and 75 μM, respectively), and rBMP4 from 100 ng/μl to 2000 ng/μl (final working concentration 1000 ng/μl). The same initial treatment windows were assessed as for *O. fusiformis*, until they were narrowed to 4–6 hpf. We compared the penetrance of the DMH1 treatment on the activation of pSMAD1/5/8 in *S. lamarcki* by using a Mantel-Haenszel chi-squared test with continuity correction (χ = 0.044, *p*-value = 0.83). For *C. teleta*, we used 40 µM DMH1 and SB431542 from 4-cell to stage 6 larvae, as reported previously^16,17^.

For *P. dumerilii*, we used DMH1 concentrations ranging from 1 to 50 μM (final working concentration 7.5 μM) and rBMP4 concentrations ranging from 100 to 2000 ng/μl (final working concentration 1500 ng/l). Initially, we exposed embryos to continuous treatment from 2 hpf and to a temporally staggered schedule, starting at 2, 4, 6, 8, 10, and 12 hpf until 72 hpf, then narrowed to 4–8 hpf for DMH1 and 2.5–12 hpf for rBMP4 once we identified the 2d cell as the source of BMP signalling. Embryos were fixed at 12 and 24 hpf to assess gene expression and at 72 hpf to evaluate morphological outcomes.

### RNA-seq profiling and differential gene expression analyses

For *O. fusiformis*, total RNA was extracted from 6 hpf blastulae right after treatments using the Monarch Total RNA Miniprep kit (New England Biolabs; Cat No.: #T2010) and used to prep strand-specific mRNA Illumina libraries that were sequenced at the Oxford Genomics Centre (University of Oxford, UK) over one lane of an Illumina NovaSeq6000 system in 2 × 150 bases mode. Adaptor and low-quality bases were removed using FastP v.0.20.1^69^. Cleaned reads were mapped to the *O. fusiformis* genome annotation^19^ using Kallisto v.0.46.2^70^. Read counts normalisation and differential gene expression analyses for each pairwise comparison between control and treatment conditions were performed with the R package DESeq2 v.1.26.0^71^. The enrichment of Gene Ontology terms in the differentially expressed genes was calculated with the TopGO R package^72^.

For *P. dumerilii*, RNA samples were collected at 8.5, 12, 18, and 24 hpf. A total of six biological batches were collected, each comprising eight samples representing the four developmental stages for DMSO- and DMH1-treated embryos and larvae. The top three batches were selected for RNA sequencing based on (i) developmental success rate, (ii) signal clarity and reproducibility in gene expression analyses, and (iii) RNA quality. RNA extraction was performed using TRIzol® reagent (Invitrogen; Cat No.: 15596026), following the manufacturer’s instructions. Strand-specific mRNA Illumina libraries were prepped and sequenced at the NGS core facility in Academia Sinica (Taiwan) over one lane of an Illumina® HiSeq 2500 System. Raw reads were quality-trimmed to remove adaptors and low-quality bases using Trimmomatic v.0.39^73^. Trimmed reads were mapped to the *Platynereis* reference genome v.0.2.1 using STAR v.2.7.11b^74^. Gene abundance was estimated with featureCounts v.2.0.1^75^. Read counts normalisation and differential gene expression analyses for each pairwise comparison between control and treatment conditions were performed with the R package DESeq2 v.1.26.0^71^, adjusting log_2_ fold changes using the shrinkage approach included in DESeq2.

### Gene isolation and gene expression analyses

For *O. fusiformis* and *C. teleta*, candidate and phenotypic marker genes were amplified using gene-specific primers and a T7 adaptor. Riboprobes were synthesised with the T7 MEGAscript kit (ThermoFisher; Cat No.: AM1334)^8^ and stored at a concentration of 50 ng/μl in hybridisation buffer at –20 °C. Whole-mount colourimetric and fluorescent in situ hybridisation in embryonic and larval stages were conducted as described elsewhere for *O. fusiformis*^33^ and *C. teleta*^67^. For *P. dumerilii*, DIG-labelled antisense RNA probes were synthesised using the DIG RNA Labelling Mix (Roche, Cat. No: 11277073910) and whole-mount colourimetric in situ hybridisation was performed as described elsewhere^48^.

### Whole-mount immunohistochemistry

Whole-mount immunohistochemistry, nuclear and F-actin staining, were conducted as described previously^8,32,33^. The primary antibodies used were mouse anti-acetylated α-tubulin (clone 6-11B-1, Merck-Sigma; Cat No.: #MABT868, 1:500), mouse anti-β-tubulin (E7, Developmental Studies Hybridoma Bank), and *P. dumerilii-*derived rabbit anti-RYamide^32,34,35^ diluted in 5% normal goat serum (NGS) in 1x PBS + 0.1% Triton X-100 (PTx) overnight. After several PTx washes, animals were incubated in the following secondary antibodies: 1:800 goat anti-mouse AlexaFluor 488 (ThermoFisher Scientific; Cat No.: A32731), 1:800 goat anti-mouse AlexaFluor 647 (ThermoFisher Scientific; Cat No.: A-21235), and 1:800 goat anti-rabbit AlexaFluor 555 (ThermoFisher Scientific; Cat No.: A32731). To detect the activation of SMAD1/5/8, the primary antibody rabbit anti-Phospho-Smad1/5 (Cell Signalling Technology; Cat No.: 9516) was used following a modified protocol used in other spiralians^23^. Briefly, wild-type, control and treated embryos stored in methanol were rehydrated gradually with 1x PBS + 0.1 % Tween-20 and permeabilised for one hour with 1x PBS + 0.2% Tween-20/0.2% Triton X-100 (PTwTx). Embryos were then blocked for one hour with 5% NGS in PTwTx and incubated overnight in 1:10 anti-pSmad1/5. After several washes with PTwTx, the embryos were incubated with 1:800 anti-rabbit AlexaFluor 555 (ThermoFisher Scientific, cat#: A-21428) plus 4′,6-diamidino-2-phenylindole (DAPI, ThermoFisher Scientific, #D3571, stock 2 mg/ml, 1:2000) as a nuclear marker overnight and stored in 70% glycerol in PBS. The same primary antibody against Phospho-Smad1/5 was used in *P. dumerilii*, producing no reliable staining. For dp-ERK1/2 staining in *S. lamarcki*, we use embryos stored in methanol at –20 °C and a 1:100 dilution of the primary antibody, mouse anti-dp-ERK1/2 (Merck-Sigma; Cat No.: #M9692), as described before^8^.

### Phenotype characterisation and classification

For *O. fusiformis*, we used morphological characters, such as the presence/absence of chaetal sacs, and gene expression to determine the loss or gain of dorsoposterior and neural tissue in the larva. For *S. lamarcki*, we used the presence/absence of the hyposphere (future trunk of the worm) and the dorsal ring of FMRFamide^+^ neurons around the gut in the episphere to determine the phenotype of the drug inhibitions. With *P. dumerilii*, morphological phenotypes were scored in 72 hpf larvae, which were anaesthetised using fresh fixative buffer and placed in a petri dish (35 mm diameter) for motion-capture imaging using an Axiocam 506 colour camera in conjunction with a Zeiss Axioskop microscope. Morphological scoring included adult eye number, body shape, and overall development.

### Imaging

Samples were mounted in 70% glycerol in 1x PBS. DIC images of the colourimetric in situ were obtained with a Leica 560 DMRA2 upright microscope equipped with an Infinity5 camera (Lumenera) or a Zeiss Axioskop microscope. Immunohistochemistry images were acquired with a Leica SP5 Laser Scanning Confocal, a Leica Stellaris 8, or a Nikon CSU-W1 Spinning Disk Confocal. Z projections were analysed with Fiji, and brightness/contrast was adjusted with Adobe Photoshop (v22.2.0). Figs were designed with Adobe Illustrator (v28.6).

### Reporting summary

Further information on research design is available in the Nature Portfolio Reporting Summary linked to this article.

## Supplementary information

Supplementary Information

Peer Review file

Reporting Summary

## Source data

Source Data

## Data Availability

All expression data generated in this manuscript have been uploaded to SRA (project ID PRJNA1267570) and Gene Expression Omnibus (study ID GSE297936). Source Data are provided with this paper (see Source Data File and Supplementary Information). Source data are provided with this paper.

## Code availability

No custom code was generated in this study.
