# Supplementary material for: Developmental system drift in dorsoventral patterning is linked to transitions to autonomous development in Annelida
Source: Nat Commun. 2026 Apr 18;17:5381. doi: 10.1038/s41467-026-71950-7 (PMC13275895; doi:10.1038/s41467-026-71950-7)
Supplement: Supplementary file 1 — Supplementary Information [file 41467_2026_71950_MOESM1_ESM.pdf]

Supplementary Information

Developmental system drift in dorsoventral patterning is linked to transitions to autonomous development in Annelida

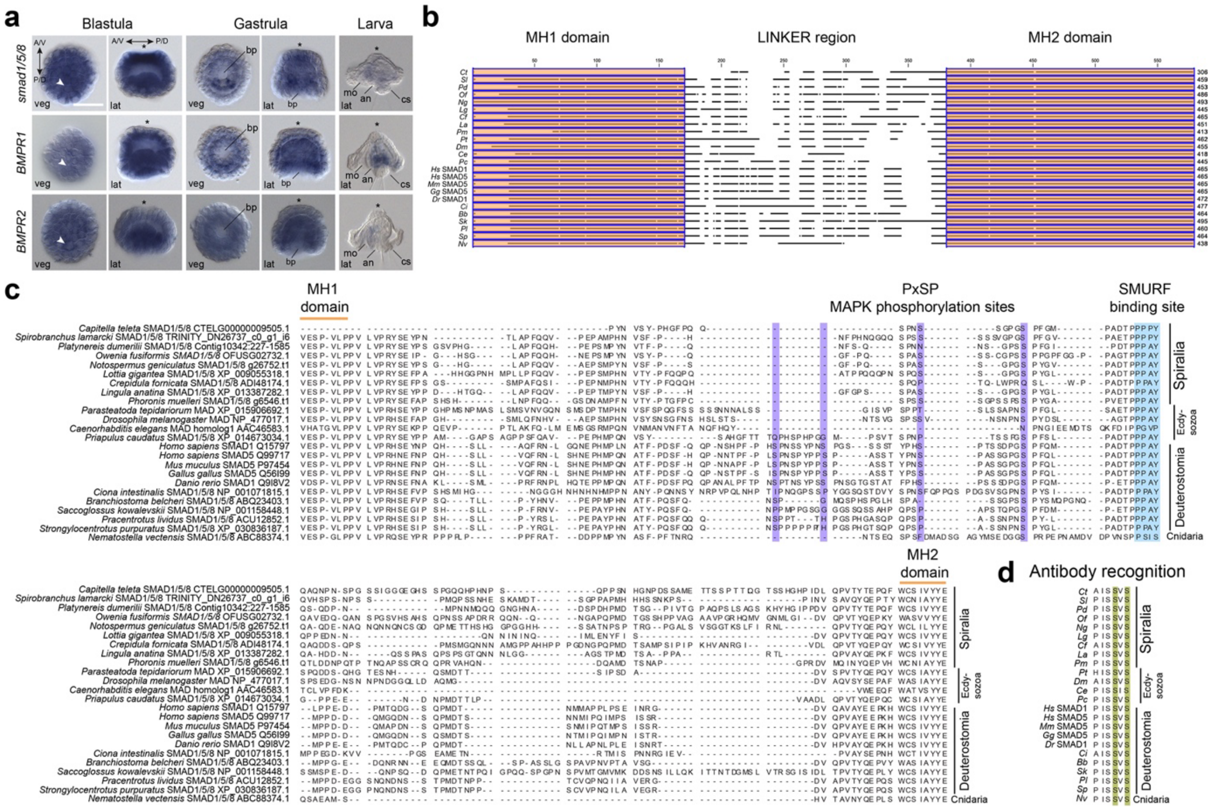

Supplementary Fig. 1 | The BMP pathway and conservation of Smad1/5/8 in *O. fusiformis*. (**a**) Whole mount *in situ* hybridisation of the secondary messenger *smad1/5/8* and receptors *bmpr1* and *bmpr2* of the BMP pathway at the blastula (6 hpf), gastrula (9 hpf) and larval (24 hpf) stages in *O. fusiformis*. (**b**) Schematic diagram of a multiple protein alignment of the MH domains and linker region of SMAD1/5/8 across bilaterian and cnidarians highlighting the high conservation of the MH domains. (**c**) Multiple protein alignment in the linker region of SMAD1/5/8 showing the presumptive MAPK phosphorylation sites (highlighted in violet). Only Deuterostomes have all sites conserved. (**d**) Multiple protein alignment of the C-terminus of SMAD1/5/8 proteins showing the conserved residues

recognised by the pSMAD1/5/8 antibody used in this study. In (**a**), the arrowheads point to the 4d organiser (always to the bottom), and the asterisks mark the animal/apical pole (always to the top). Scale bars are 50 µm. an anus, ao apical organ, bp blastopore, ch chaetae, cs chaetal sac, fg foregut, lat lateral, mo mouth, pt prototroch, veg vegetal. Source data are provided as a Source Data file.

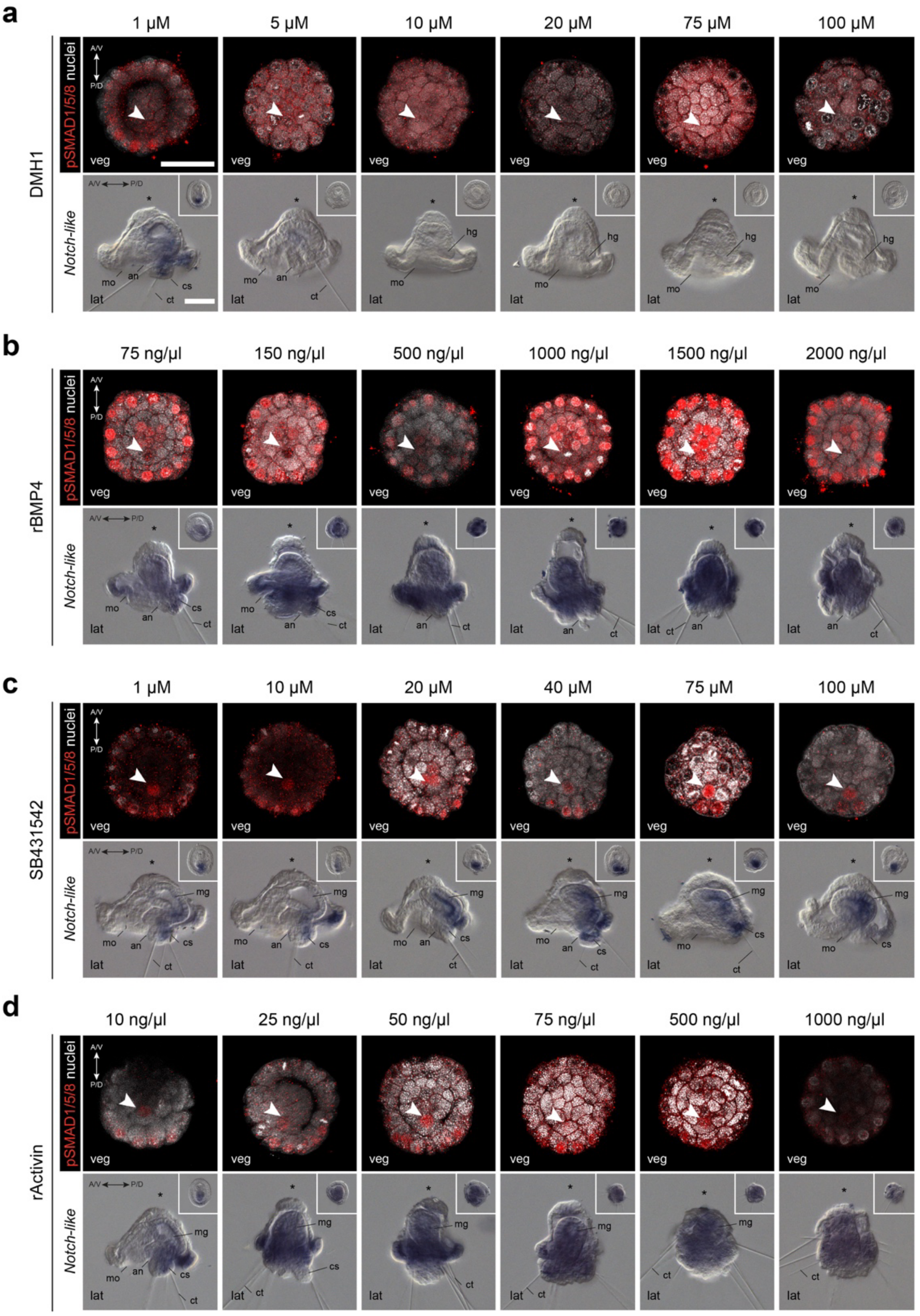

Supplementary Fig. 2 | Dose-response treatments in *O. fusiformis*. (**a**–**d**) Z-stack projections of embryos treated with increasing concentrations of DMH1 (**a**), rBMP4 (**b**), SB431542 (c), and rActivin A (**d**), fixed at 6 hpf and stained against pSMAD1/5/8 (red, counterstained with nuclei in grey; upper row), and fixed at 24 hpf and used to assess the expression of the dorsal marker gene *Notch-like* through whole mount *in situ* hybridisation

(bottom row). The minimal concentrations producing the most penetrant axial defects are 20 μM DMH1, 1000 ng/μl rBMP4, 40 μM SB431542, and 75 ng/μl rActivin A. In all panels, vegetal views are with the 4d cell and posterodorsal to the bottom, and lateral views are with anteroventral to the left. Asterisks indicate the animal/apical pole. Scale bars are 50 μm. an anus, ct chaetae, cs chaetal sac, hg hindgut, lat lateral, mg midgut, mo mouth, pt prototroch, veg vegetal.

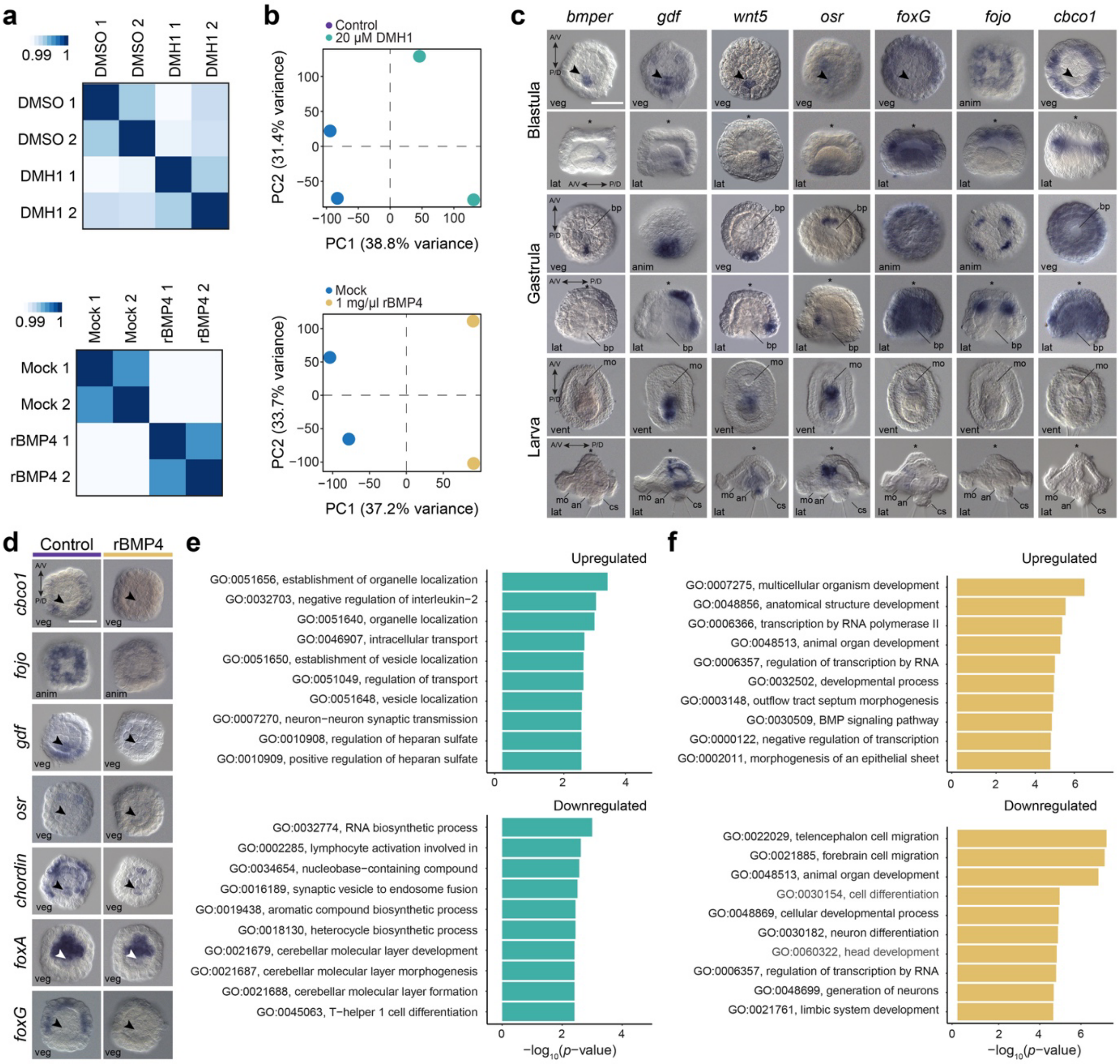

Supplementary Fig. 3 | Differential gene expression analyses in DMH1 and rBMP4-treated *O. fusiformis* embryos. (**a**) Hierarchically clustered pairwise correlation matrix between DMH1 (top) and rBMP4 (bottom) treated and control samples. The scale shows the relative difference in Euclidean distance. (**b**) Principal component (PC) analysis plots for DMH1 (top) and rBMP4 (bottom) transcriptomic analyses indicate that the main source of transcriptional variability between samples is the treatment condition (PC1). (**c**) Whole mount *in situ* hybridisation of selected candidate, differentially expressed genes in the rBMP4-treated condition, at the blastula, gastrula, and larval stages. (**d**) Whole mount *in situ* hybridisation validates the downregulation of candidate genes at the blastula stage (6 hpf)

after rBMP4 treatment. (**e**, **f**) Bar plots showing the top ten enriched Gene Ontology terms in differentially expressed genes after DMH1 (**e**) and rBMP4 treatment (**f**). In (**c**, **d**), the arrowheads point to the 4d organiser (in vegetal views always to the bottom), and the asterisks mark the animal/apical pole (in lateral views, always to the top). Scale bars are 50 µm. an anus, anim animal, ao apical organ, bp blastopore, ch chaetae, cs chaetal sac, fg foregut, lat lateral, mo mouth, pt prototroch, veg vegetal. Source data are provided as a Source Data file.

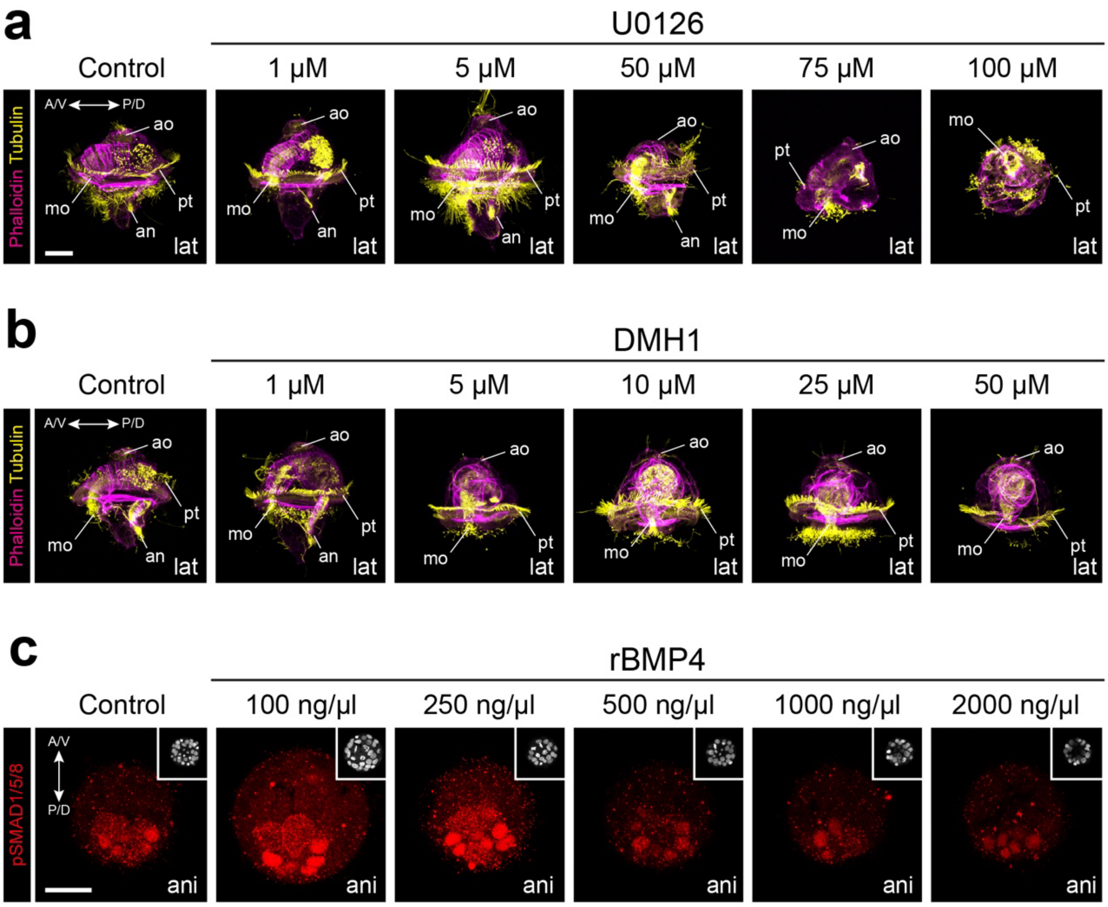

Supplementary Fig. 4 | Dose-response drug treatments in *S. lamarcki*. (**a**–**c**) Z-stack projections of control samples and embryos treated with increasing concentrations of U0126 (**a**), DMH1 (**b**), and rBMP4 (**c**), fixed at 48 hpf (**a**, **b**) and 6 hpf (**c**), and stained against tubulin (yellow) and filament actin (palloidin; magenta) (**a**, **b**) or pSMAD1/5/8 (red; **c**). (**a**, **b**) 75 µM U0126 and 5 µM DMH1 are the minimal concentrations causing axial defects. (**c**) Unlike in *O. fusiformis*, treatment with rBMP4 does not induce ectopic and ubiquitous activation of pSMAD1/5/8 in *S. lamarcki*. Insets in (**c**) are nuclear (DAPI) staining. Lateral views are with anteroventral to the left, and animal views are with posterodorsal to the bottom. Scale bars are 50 µm. an anus, ani animal, ao apical organ, lat lateral, mo mouth, pt prototroch.

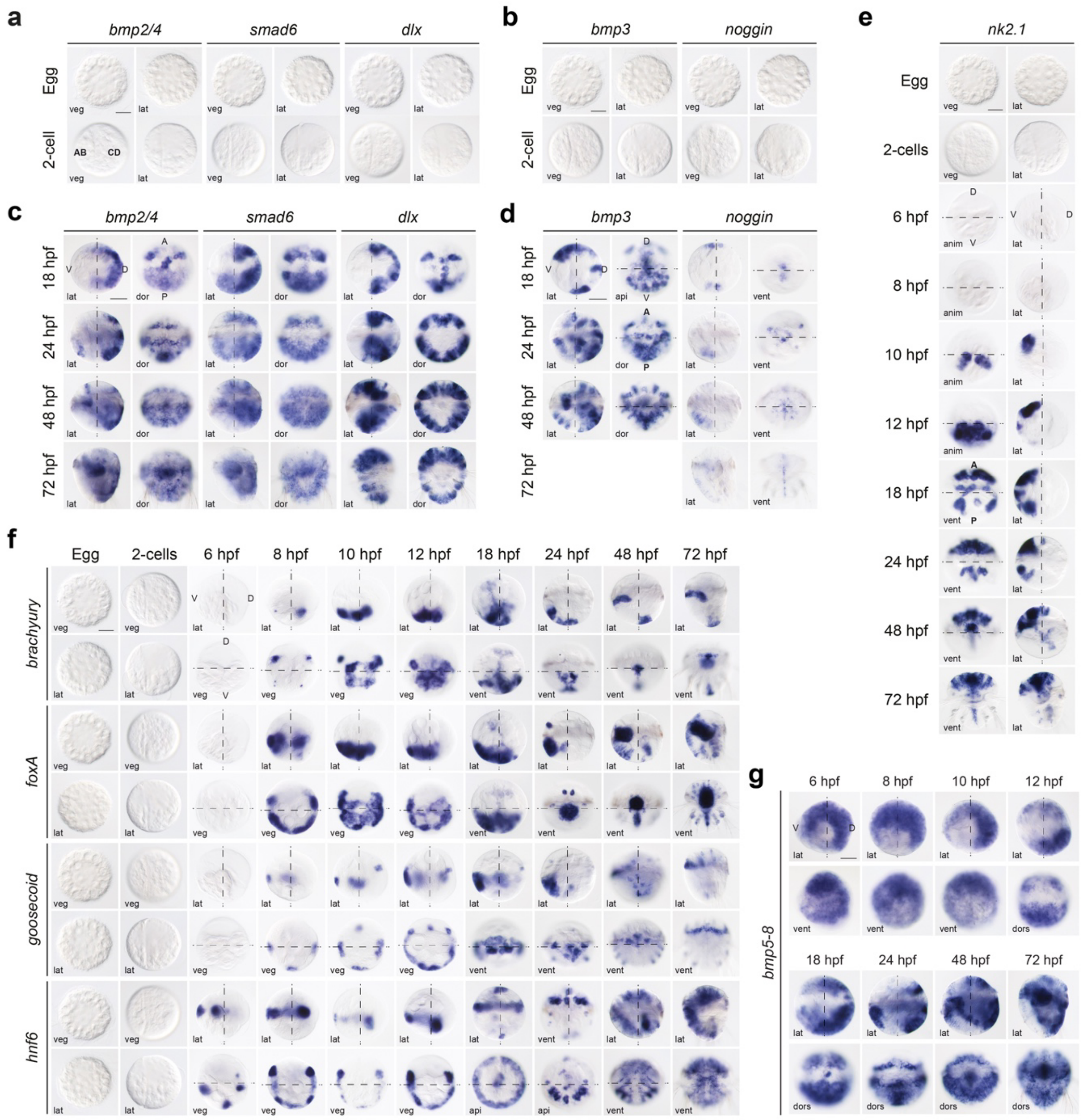

Supplementary Fig. 5 | Expression of dorsoventral marker genes in *P. dumerilii.* (a–e) Whole mount *in situ* hybridisation of dorsoventral markers at selected embryonic and larval stages. (**f**, **g**) Whole mount *in situ* hybridisation of *brachyury*, *foxA*, *goosecoid*, *hnf6*, and *bmp5-8* at selected embryonic and larval stages. In lateral views, ventral is to the left, and in animal/vegetal views, ventral is to the bottom. Dotted lines separate the dorsal and ventral hemispheres. anim animal, D dorsal, dors dorsal, veg vegetal, vent ventral, lat lateral, V ventral.

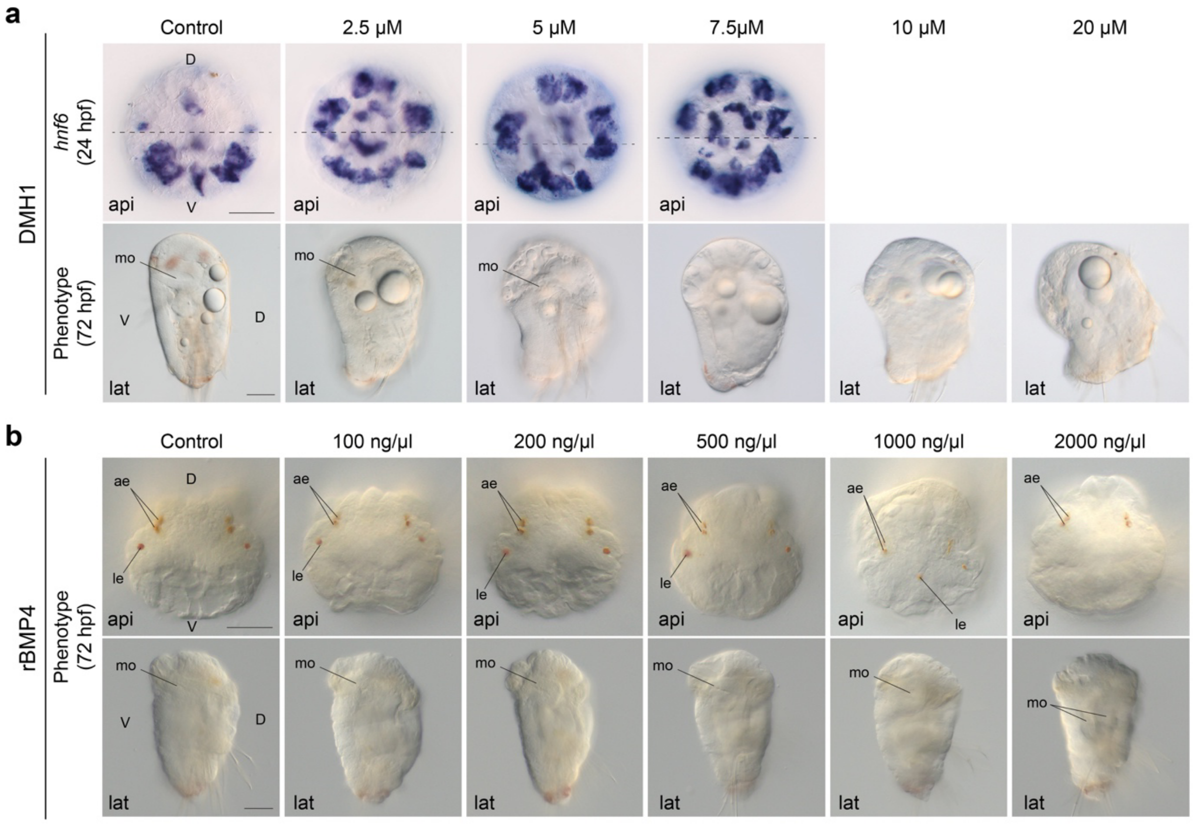

Supplementary Fig. 6 | Dose-response drug treatments in *P. dumerilii*. (**a**) Dose-response phenotypes after increasing DMH1 concentrations, showing the dorsoventral head patterning in 24 hpf larvae (top row), as revealed by whole mount in situ hybridisation of the ventral marker *hnf6*, and overall morphology of 72 hpf (bottom row). (**b**) Phenotypic characterisation of increasing concentrations of rBMP4 in 72 hpf *P. dumerilii* larvae. For DMH1 and rBMP4, 7.5 µM and 2000 ng/µl are the minimum concentrations producing a penetrant dorsoventral phenotype. In lateral views, ventral is to the left, and in apical views, ventral is to the bottom. Dotted lines separate the dorsal and ventral hemispheres. ae adult eyes, api apical, D dorsal, lat lateral, le larval eyes, mo mouth, V ventral.

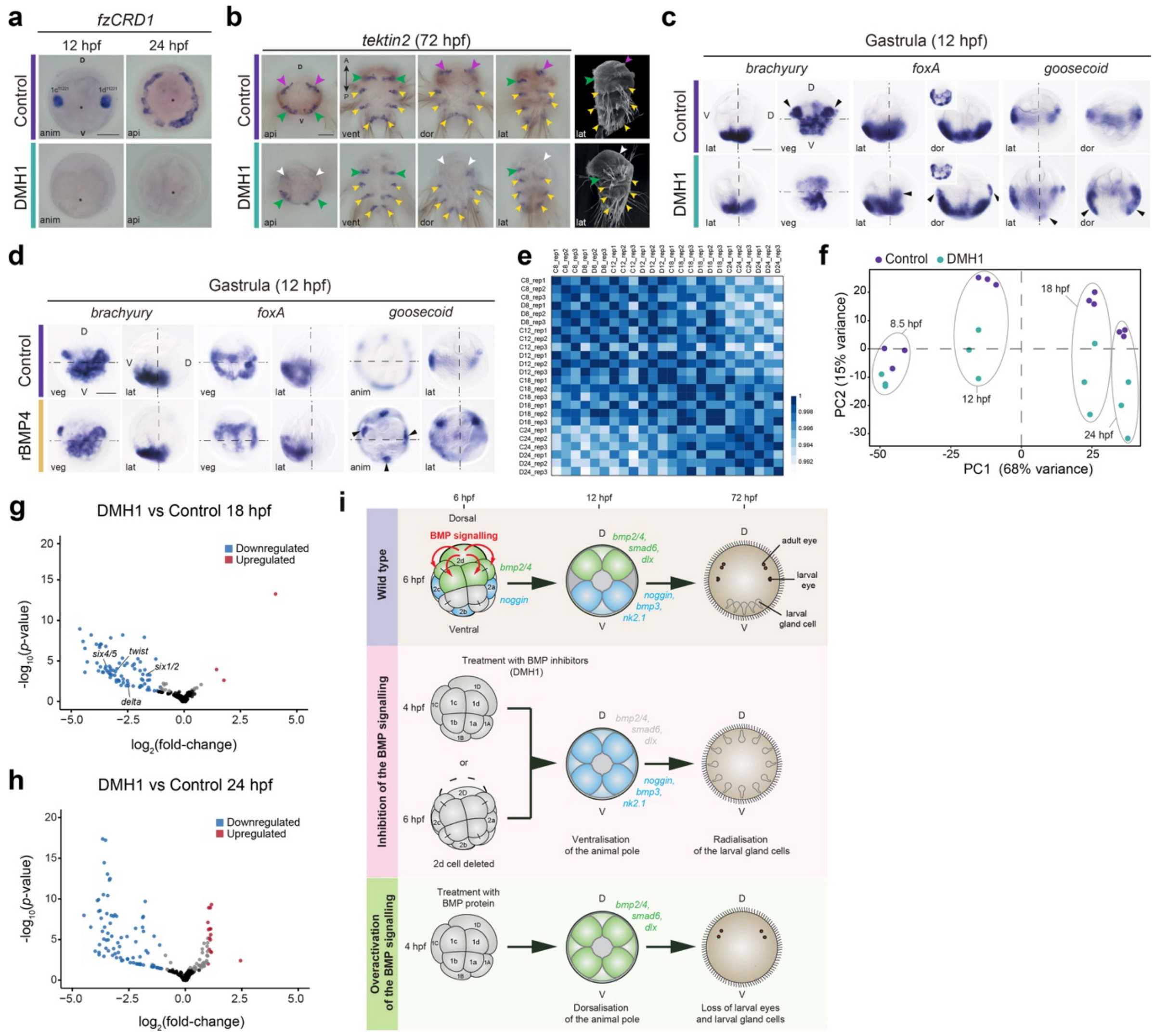

Supplementary Fig. 7 | The role of BMP in dorsoventral axis specification in *Platynereis*. (**a**) Whole mount *in situ* hybridisation of nephroblast (1c[11221]& 1d[11221])/headkidney marker *fzCRD1* in control and DMH1-treated 12 and 24 hpf larvae. (**b**) Whole mount *in situ* hybridisation of the ciliary marker *tektin-2* and scanning electron microscopy images of control and DMH1-treated 72 hpf larvae. Ciliary bands like the dorsoanterior akrotroch (present/red, absent/white), the metatroch (green), and the paratrochs (yellow) are indicated by arrows. (**c**–**d**) Whole mount *in situ* hybridisation of *brachyury*, *foxA* and *goosecoid* in control and DMH1 (**c**) and rBMP4-treated (**d**) gastrula. (**e**) Hierarchically clustered pairwise correlation matrix between DMH1-treated and control samples. The scale shows the relative difference in Euclidean distance. (**f**) Principal component (PC) analysis plot for DMH1-

treated vs control RNA-seq analyses. (**g**, **h**) Volcano plots depicting differentially expressed genes in 18 and 24 hpf larvae after DMH1 treatment from 4 to 8 hpf. (**i**) Diagram of the role of BMP signalling in wild type, rBMP4- and DMH1-treated, and 2d cell-ablated embryos, depicting the effects on dorsoventral marker gene expression at the gastrula stage (12 hpf), and the effects on morphology at 72 hpf larva. In lateral views, ventral is to the left, and in animal/apical/vegetal views, ventral is to the bottom. In dorsoventral views, anterior is to the top. Dotted lines separate the dorsal and ventral hemispheres. anim animal, api apical, D dorsal, dor dorsal, lat lateral, veg vegetal, V ventral. Drawings are not scale. Source data are provided as a Source Data file.

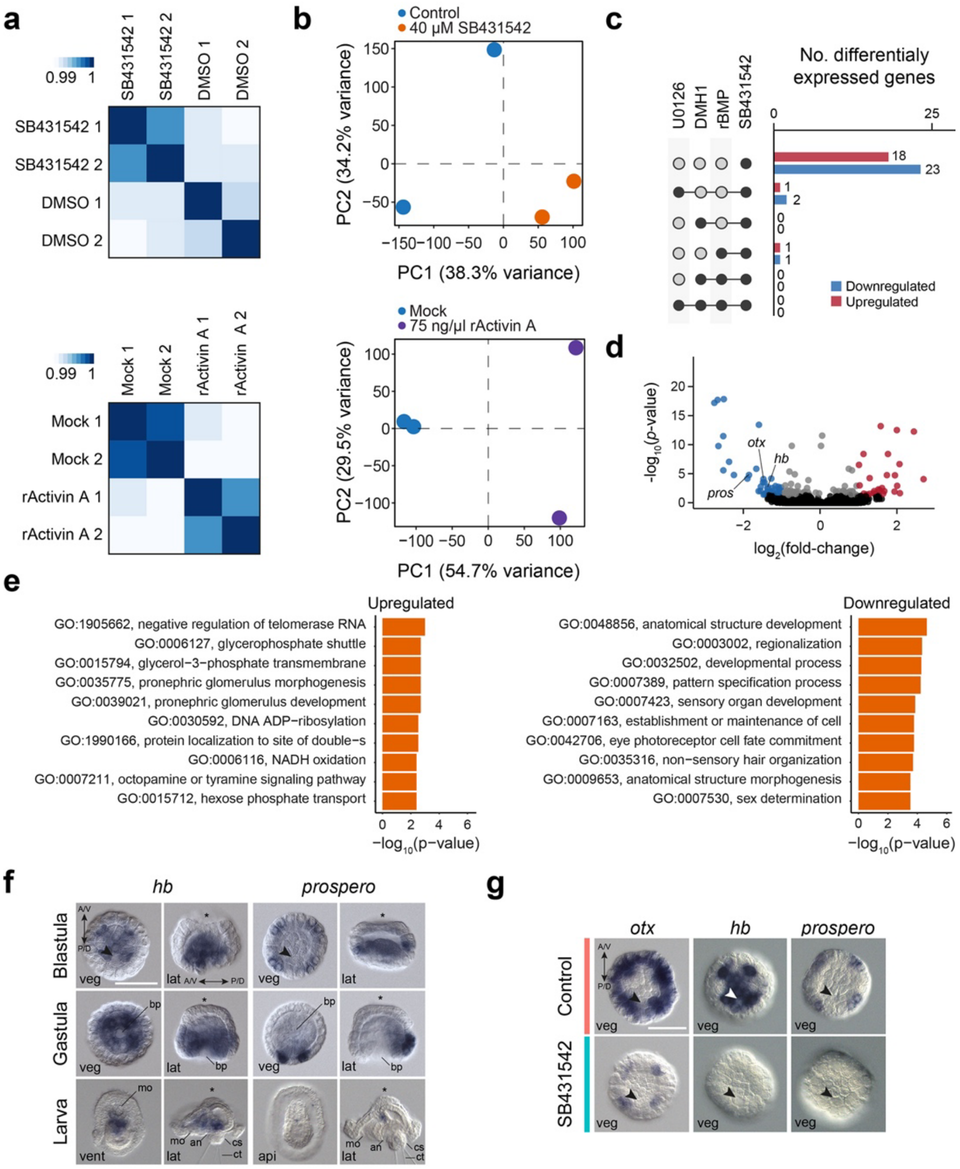

Supplementary Fig. 8 | The gene regulatory network downstream of Activin/Nodal pathway in *O. fusiformis*. (**a**) Hierarchically clustered pairwise correlation matrix between SB431542 (top) and rActivin A (down) treated and control samples. The scale shows the relative difference in Euclidean distance. (**b**) Principal component (PC) analysis plots for SB431542 (top) and rActivin (bottom) transcriptomic analyses indicate that the main source of transcriptional variability between samples is the treatment condition (PC1). (**c**) Bar plots indicating the number of differentially expressed genes in different treatments. Full circles connected with a line indicated shared differentially expressed genes. (**d**) Volcano plot

depicting differentially expressed genes after SB431542 treatment in 6 hpf blastulae. (**e**) Bar plots showing the top ten enriched Gene Ontology terms in differentially expressed genes after SB431542. (**f**, **g**) Complete mount *in situ* hybridisation of selected candidate differentially expressed genes in SB431542-treated embryos at the blastula, gastrula, and larval stages. In (**f**, **g**), the arrowheads point to the 4d organiser, and the asterisks mark the animal/apical pole. Lateral views are with anteroventral to the left, and apical views are with dorsal to the top. Scale bars are 50 µm. an anus, bp blastopore, ch chatae, cs chaetal sac, lat lateral, mo mouth, pt prototroch, veg vegetal. Source data are provided as a Source Data file.

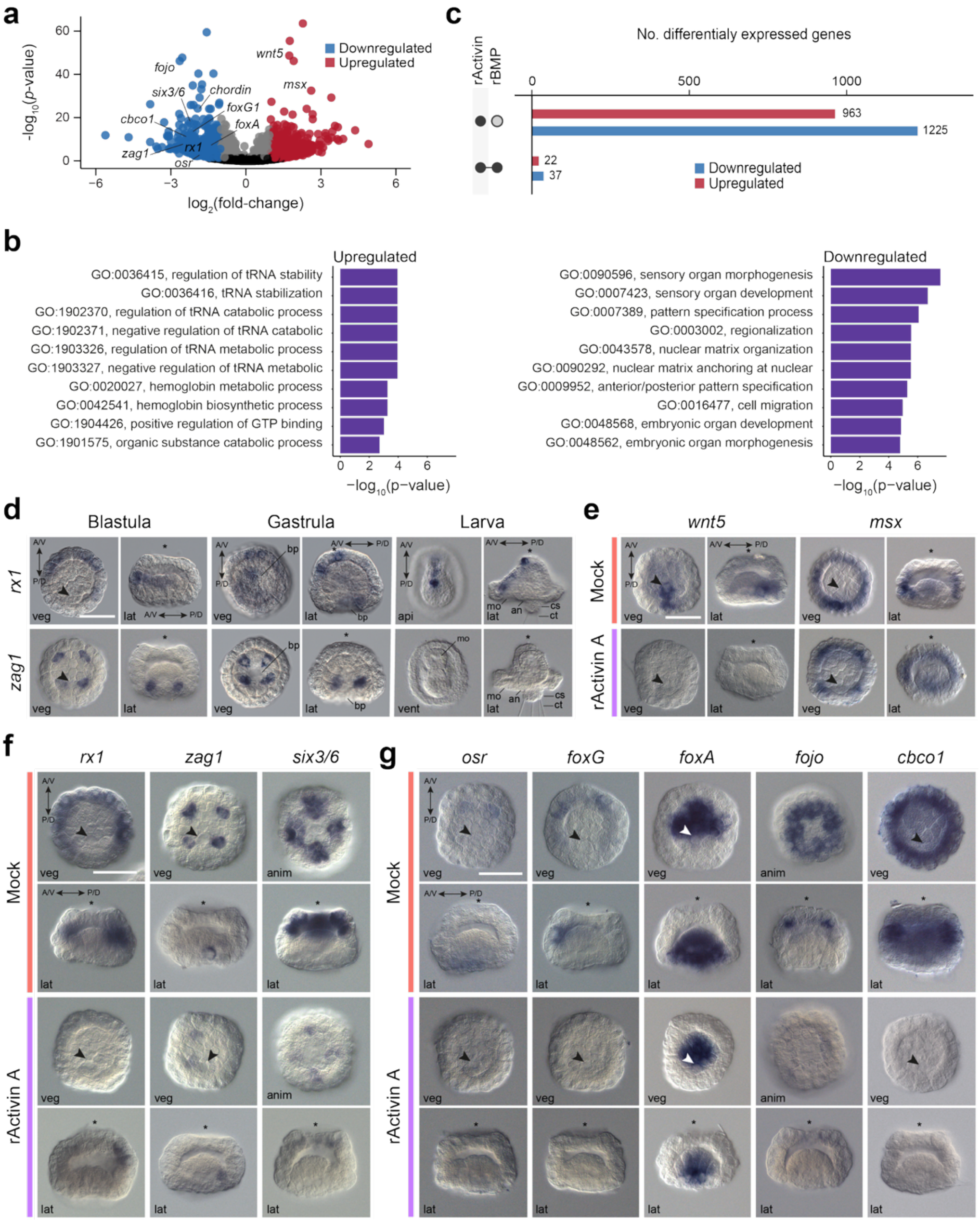

Supplementary Fig. 9 | Differential gene expression analyses in recombinant Activin A-treated *O. fusiformis* embryos. (**a**) Volcano plot depicting differentially expressed genes after rActivin A treatment in 6 hpf blastulae. (**b**) Bar plots showing the top ten enriched Gene Ontology terms in differentially expressed genes after rActivin A treatment. (**c**) Bar plots indicating the number of differentially expressed genes in different treatments. Full circles connected with a line indicated shared differentially expressed genes. (**d**) Whole mount *in situ* hybridisation of selected candidate differentially expressed genes after rActivin A treatment in wild type embryos at blastula, gastrula, and larval stages. (**e**–**f**) Whole mount *in*

situ hybridisation of candidate genes downregulated only in recombinant Activin A-treated blastula (**e**), upregulated in rBMP4 and rActivin A-treated blastula (**f**), and downregulated in rBMP4 and rActivin A-treated blastula. In (**b**–**f**), the arrowheads point to the 4d organiser, and the asterisks mark the animal/apical pole. Lateral views are with anteroventral to the left, and apical views are with dorsal to the top. Scale bars are 50 µm. an anus, anim animal, bp blastopore, ch chatae, cs chaetal sac, lat lateral, mo mouth, veg vegetal. Source data are provided as a Source Data file.

Supplementary Tables 1. Scoring of pSMAD1/5/8 immunoreactivity in blastula (6 hpf) of *O. fusiformis* embryos after U0126, DMH1, SB-431542, rBMP4 and rActivin A treatments.

Species	Treatment	Total_n	pSmad1/5_positive
Owenia_fusiformis	0.1% DMSO + 1x Mock	14	14
Owenia_fusiformis	10 uM U0126 + 1000 ng/ml rBMP	17	0
Owenia_fusiformis	10 uM U0126	5	0
Owenia_fusiformis	1000 ng/ml rBMP	9	9
Owenia_fusiformis	0.4% DMSO	25	25
Owenia_fusiformis	40 uM SB431542	57	57
Owenia_fusiformis	0.2% DMSO	35	35
Owenia_fusiformis	10 uM U0126	63	0
Owenia_fusiformis	20 uM DMH1	47	0
Owenia_fusiformis	40 uM SB431542	74	74
Owenia_fusiformis	0.2% DMSO	7	7
Owenia_fusiformis	0.2% DMSO	13	13
Owenia_fusiformis	20 uM DMH1	14	0
Owenia_fusiformis	20 uM DMH1	22	0
Owenia_fusiformis	20 uM DMH1	13	0
Owenia_fusiformis	20 uM DMH1	12	0
Owenia_fusiformis	2x Mock	106	102
Owenia_fusiformis	1000 ng/ml rBMP	45	44
Owenia_fusiformis	2000 ng/ml rBMP	70	66

Supplementary Table 2. Scoring of *chordin* and *bmp2/4* expression in larvae of *O. fusiformis* after U0126 treatment.

Species	Treatment	Stage	Total_n	*chordin*	*bmp2/4*
Owenia_fusiformis	0.1% DMSO	6hp	763	349	414
Owenia_fusiformis	10 uM U0126	6hpf	195	129	66
Owenia_fusiformis	0.1% DMSO	24hpf	479	191	288
Owenia_fusiformis	10 uM U0126	24hpf	86	39	47

Supplementary Table 3. Dose-response of treatments on the effect of pSMAD1/5/8 in blastula (6hpf) of *O. fusiformis.*

Species	Treatment	Total_n	pSmad1/5_positive	Note
Owenia_fusiformis	1% DMSO	48	48	
Owenia_fusiformis	1 uM DMH1	54	54	
Owenia_fusiformis	5 uM DMH1	39	0	
Owenia_fusiformis	10 uM DMH1	60	0	
Owenia_fusiformis	20 uM DMH1	32	0	
Owenia_fusiformis	75 uM DMH1	36	0	
Owenia_fusiformis	100 uM DMH1	28	0	
Owenia_fusiformis	1 uM SB431542	95	95	
Owenia_fusiformis	10 uM SB431542	18	18	
Owenia_fusiformis	20 uM SB431542	15	15	
Owenia_fusiformis	40 uM SB431542	25	25	
Owenia_fusiformis	75 uM SB431542	40	40	
Owenia_fusiformis	100 uM SB431542	30	30	
Owenia_fusiformis	2x Mock	54	54	
Owenia_fusiformis	75 ng/ml rBMP	56	56	BMP_everywhere
Owenia_fusiformis	150 ng/ml rBMP	67	67	BMP_everywhere
Owenia_fusiformis	500 ng/ml rBMP	40	40	BMP_everywhere
Owenia_fusiformis	1000 ng/ml rBMP	75	75	BMP_everywhere
Owenia_fusiformis	1500 ng/ml rBMP	23	23	BMP_everywhere
Owenia_fusiformis	2000 ng/ml rBMP	54	54	BMP_everywhere
Owenia_fusiformis	10 ng/ml rActivin	16	16	
Owenia_fusiformis	25 ng/ml rActivin	49	49	
Owenia_fusiformis	50 ng/ml rActivin	15	15	
Owenia_fusiformis	75 ng/ml rActivin	20	20	
Owenia_fusiformis	500 ng/ml rActivin	23	23	
Owenia_fusiformis	1000 ng/ml rActivin	50	0	

Supplementary Table 4. Scoring of morphological phenotypes in larvae of *O. fusiformis* after DMH1, SB431542, rBMP4 and rActivin A treatments.

Species	Treatment	Replica	Total n	Normal	No chaetae	Multiple chaetae	Abnormal proto large_mouth	Underdeveloped	% phenotype	% abnormal
Owenia_fusiformis	02DMSO	1	30	20	0	0	0	10	0.66	0.33
Owenia_fusiformis	02DMSO	2	33	15	0	0	0	18	0.45	0.54
Owenia_fusiformis	02DMSO	3	13	11	0	0	0	2	0.84	0.15
Owenia_fusiformis	20uMDMH1	1	35	0	29	0	0	6	0.82	0.17
Owenia_fusiformis	20uMDMH1	2	44	0	29	0	0	15	0.65	0.34
Owenia_fusiformis	20uMDMH1	3	16	0	13	0	0	3	0.81	0.18
Owenia_fusiformis	1xMock	1	40	36	0	0	0	4	0.9	0.1
Owenia_fusiformis	1xMock	2	100	68	0	0	0	32	0.68	0.32
Owenia_fusiformis	1xMock	3	19	14	0	0	0	5	0.73	0.26
Owenia_fusiformis	1000BMP	1	41	0	0	39	0	2	0.95	0.04
Owenia_fusiformis	1000BMP	2	41	0	0	30	0	11	0.73	0.26
Owenia_fusiformis	1000BMP	3	18	0	0	16	0	2	0.88	0.11
Owenia_fusiformis	75Activin	1	40	1	0	37	0	2	0.92	0.05
Owenia_fusiformis	75Activin	2	43	0	0	27	0	16	0.62	0.37
Owenia_fusiformis	75Activin	3	19	0	0	17	0	2	0.89	0.10
Owenia_fusiformis	04DMSO	1	36	26	0	0	0	10	0.72	0.27
Owenia_fusiformis	04DMSO	2	33	21	0	0	0	12	0.63	0.36
Owenia_fusiformis	04DMSO	3	18	12	0	0	0	6	0.66	0.33
Owenia_fusiformis	40SB	1	34	0	0	0	24	10	0.70	0.294
Owenia_fusiformis	40SB	2	28	0	0	0	20	8	0.71	0.28
Owenia_fusiformis	40SB	3	13	0	0	0	9	4	0.69	0.30

Supplementary Table 5. Dose-response of treatments on the effect of *Notch-like* expression in *O. fusiformis* larvae.

Species	Treatment	Total_n	*notch-like*
Owenia_fusiformis	1% DMSO	23	23
Owenia_fusiformis	1 uM DMH1	17	17
Owenia_fusiformis	5 uM DMH1	22	0
Owenia_fusiformis	10 uM DMH1	15	0
Owenia_fusiformis	20 uM DMH1	11	0
Owenia_fusiformis	75 uM DMH1	19	0
Owenia_fusiformis	100 uM DMH1	26	0
Owenia_fusiformis	1 uM SB431542	34	34
Owenia_fusiformis	10 uM SB431542	43	43
Owenia_fusiformis	20 uM SB431542	26	26
Owenia_fusiformis	40 uM SB431542	46	46
Owenia_fusiformis	75 uM SB431542	41	41
Owenia_fusiformis	100 uM SB431542	31	31
Owenia_fusiformis	2x Mock	54	54
Owenia_fusiformis	75 ng/ml rBMP	39	39
Owenia_fusiformis	150 ng/ml rBMP	16	16
Owenia_fusiformis	500 ng/ml rBMP	29	29
Owenia_fusiformis	1000 ng/ml rBMP	57	57
Owenia_fusiformis	1500 ng/ml rBMP	27	27
Owenia_fusiformis	2000 ng/ml rBMP	48	48
Owenia_fusiformis	10 ng/ml rActivin	13	13
Owenia_fusiformis	25 ng/ml rActivin	27	27
Owenia_fusiformis	50 ng/ml rActivin	24	24
Owenia_fusiformis	75 ng/ml rActivin	22	22
Owenia_fusiformis	500 ng/ml rActivin	19	19
Owenia_fusiformis	1000 ng/ml rActivin	37	37

Supplementary Table 6. Scoring of morphological phenotypes in *O. fusiformis* larvae after different treatment windows with DMH1, SB431542, rBMP4 and rActivin A.

Species	Treatment	Time window	Total n	Normal	No chaetae	Multiple chaetae	Abnormal proto large mouth
Owenia_fusiformis	0.2% DMSO	0.5hpf-to-4-cell	17	17	0	0	0
Owenia_fusiformis	20 uM DMH1	0.5hpf-to-4-cell	13	0	13	0	0
Owenia_fusiformis	20 uM SB431542	0.5hpf-to-4-cell	121	121	0	0	0
Owenia_fusiformis	1x Mock	0.5hpf-to-4-cell	19	19	0	0	0
Owenia_fusiformis	1000 ng/ml rBMP	0.5hpf-to-4-cell	85	0	0	85	0
Owenia_fusiformis	75 ng/ml rActivin A	0.5hpf-to-4-cell	29	29	0	0	0
Owenia_fusiformis	0.2% DMSO	0.5hpf-to-8-cell	16	16	0	0	0
Owenia_fusiformis	20 uM DMH1	0.5hpf-to-8-cell	13	0	13	0	0
Owenia_fusiformis	20 uM SB431542	0.5hpf-to-8-cell	17	17	0	0	0
Owenia_fusiformis	1x Mock	0.5hpf-to-8-cell	157	157	0	0	0
Owenia_fusiformis	1000 ng/ml rBMP	0.5hpf-to-8-cell	41	0	0	41	0
Owenia_fusiformis	75 ng/ml rActivin A	0.5hpf-to-8-cell	143	10	0	133	0
Owenia_fusiformis	0.2% DMSO	0.5hpf-to-3hpf	16	16	0	0	0
Owenia_fusiformis	20 uM DMH1	0.5hpf-to-3hpf	11	0	0	0	11
Owenia_fusiformis	20 uM SB431542	0.5hpf-to-3hpf	11	0	0	0	0
Owenia_fusiformis	1x Mock	0.5hpf-to-3hpf	40	40	0	0	0
Owenia_fusiformis	1000 ng/ml rBMP	0.5hpf-to-3hpf	27	0	0	27	0
Owenia_fusiformis	75 ng/ml rActivin A	0.5hpf-to-3hpf	77	0	0	77	0
Owenia_fusiformis	0.2% DMSO	0.5hpf-to-4hpf	8	8	0	0	0
Owenia_fusiformis	20 uM DMH1	0.5hpf-to-4hpf	56	0	56	0	0
Owenia_fusiformis	20 uM SB431542	0.5hpf-to-4hpf	11	0	0	0	11
Owenia_fusiformis	1x Mock	0.5hpf-to-4hpf	59	59	0	0	0
Owenia_fusiformis	1000 ng/ml rBMP	0.5hpf-to-4hpf	43	0	0	43	0
Owenia_fusiformis	75 ng/ml rActivin A	0.5hpf-to-4hpf	47	0	0	47	0
Owenia_fusiformis	0.2% DMSO	6hpf-to-24hpf	29	29	0	0	0
Owenia_fusiformis	20 uM DMH1	6hpf-to-24hpf	10	10	0	0	0
Owenia_fusiformis	20 uM SB431542	6hpf-to-24hpf	15	15	0	0	0
Owenia_fusiformis	1x Mock	6hpf-to-24hpf	34	34	0	0	0
Owenia_fusiformis	1000 ng/ml rBMP	6hpf-to-24hpf	37	37	0	0	0
Owenia_fusiformis	75 ng/ml rActivin A	6hpf-to-24hpf	28	24	0	4	0

Supplementary Table 7. Scoring of *cdx* and *gsc* expression in larvae of *O. fusiformis* after different treatment windows with DMH1, SB431542, rBMP4 and rActivin A.

Species	Treatment	Total_n	*cdx*	*gsc*	Notes
Owenia_fusiformis	0.2% DMSO	133	76	57	
Owenia_fusiformis	20uM DMH1	8	6	2	
Owenia_fusiformis	1x Mock	16	11	5	
Owenia_fusiformis	1000 ng/ml rBMP	13	4	9	
Owenia_fusiformis	0.2% DMSO	417	139	278	
Owenia_fusiformis	20uM DMH1	505	268	237	
Owenia_fusiformis	1x Mock	218	125	93	Mock is the same as s the rActivin treatment
Owenia_fusiformis	1000 ng/ml rBMP	232	95	137	Different plate
Owenia_fusiformis	0.4% DMSO	39	20	19	
Owenia_fusiformis	40uM SB431542	98	48	50	
Owenia_fusiformis	1x Mock	34	11	23	
Owenia_fusiformis	75 ng/ml rActivin A	21	12	9	
Owenia_fusiformis	0.4% DMSO	572	245	327	
Owenia_fusiformis	40uM SB431542	267	48	219	
Owenia_fusiformis	1x Mock	NA	NA	NA	Mock is the same as the rBMP treatment
Owenia_fusiformis	75 ng/ml rActivin A	122	30	92	

Supplementary Table 8. Scoring of *Notch-like* and *BAMBI* expression in *O. fusiformis* larvae after DMH1, SB-431542, rBMP4 and rActivin A treatment.

Species	Treatment	Total_n	*notch-like*	*BAMBI*
Owenia_fusiformis	0.2% DMSO	26	18	8
Owenia_fusiformis	20uM DMH1	17	8	9
Owenia_fusiformis	40uM SB431542	66	43	23
Owenia_fusiformis	1x Mock	78	62	16
Owenia_fusiformis	1000 ng/ml rBMP	44	35	9
Owenia_fusiformis	75 ng/ml rActivin A	136	87	49

Supplementary Table 9. Number of differentially expressed genes in the blastula (6 hpf) of *O. fusiformis* after DMH1, SB431542, rBMP4 and rActivin A treatments.

Species	Pair-wise	down	up
Owenia_fusiformis	02_DMSO_vs_DMH1	19	12
Owenia_fusiformis	Mock_vs_BMP	53	68
Owenia_fusiformis	04_DMSO_vs_SB	41	27
Owenia_fusiformis	Mock_vs_activin	1295	998
Owenia_fusiformis	Mock_vs_BMP_vs_activin	38	22

Supplementary Table 10. Differentially upregulated genes in the blastula (6 hpf) of *O. fusiformis* after DMH1 treatment.

Species	Gene_ID	mean	log2FoldChange	lfcSE	stat	pvalue	padj
Owenia_fusiformis	OFUSG01032.4	699.9155	0.666342	0.123274	5.424785	5.80E-08	4.97E-05
Owenia_fusiformis	OFUSG01143.1	971.1421	0.504958	0.123128	4.301531	1.70E-05	0.005493
Owenia_fusiformis	OFUSG07501.5	540.4362	0.674714	0.120425	5.869639	4.37E-09	5.89E-06
Owenia_fusiformis	OFUSG08474.1	259.8365	0.705274	0.099541	8.894378	5.87E-19	3.57E-15
Owenia_fusiformis	OFUSG09772.2	290.6948	0.592464	0.107986	6.174032	6.66E-10	1.08E-06
Owenia_fusiformis	OFUSG10355.1	2130.311	0.50347	0.124093	3.839738	0.000123	0.025569
Owenia_fusiformis	OFUSG13298.2	1189.501	0.589475	0.122749	5.159411	2.48E-07	0.000162
Owenia_fusiformis	OFUSG16391.1	12213.29	0.538037	0.080723	6.661151	2.72E-11	6.00E-08
Owenia_fusiformis	OFUSG18429.1	137558.8	0.574362	0.051555	11.14067	7.95E-29	9.66E-25
Owenia_fusiformis	OFUSG19285.1	342.5683	0.517651	0.089457	8.060393	7.60E-16	3.08E-12
Owenia_fusiformis	OFUSG26253.2	251.73	0.670696	0.097438	7.493294	6.72E-14	2.04E-10
Owenia_fusiformis	OFUSG26959.3	215.2976	0.647787	0.097384	9.288774	1.56E-20	1.26E-16

Supplementary Table 11. Differentially downregulated genes in the blastula (6 hpf) of *O. fusiformis* after DMH1 treatment.

Species	transcript_id	mean	log2FoldChange	lfcSE	stat	pvalue	padj
Owenia_fusiformis	OFUSG04687.1	679.961	-1.37045	0.123257	-11.9122	1.02E-32	2.48E-28
Owenia_fusiformis	OFUSG05666.1	1282.087	-0.54119	0.122969	-4.41913	9.91E-06	0.003882
Owenia_fusiformis	OFUSG06621.1	4754.335	-0.5377	0.096081	-5.59452	2.21E-08	2.12E-05
Owenia_fusiformis	OFUSG06854.3	376.0822	-0.59685	0.115226	-5.4147	6.14E-08	4.97E-05
Owenia_fusiformis	OFUSG07945.2	3331.442	-0.50744	0.105477	-4.80758	1.53E-06	0.000757
Owenia_fusiformis	OFUSG08512.2	1646.197	-0.52807	0.120622	-4.36387	1.28E-05	0.004564
Owenia_fusiformis	OFUSG09704.2	1510.408	-0.55986	0.121174	-4.63505	3.57E-06	0.001548
Owenia_fusiformis	OFUSG11344.1	5026.557	-0.5184	0.092737	-5.58975	2.27E-08	2.12E-05
Owenia_fusiformis	OFUSG12735.1	2887.152	-0.58274	0.109861	-5.32044	1.04E-07	7.86E-05
Owenia_fusiformis	OFUSG13298.1	939.7799	-0.75895	0.12372	-6.20693	5.40E-10	9.37E-07
Owenia_fusiformis	OFUSG16561.1	1807.501	-0.50836	0.123806	-4.1535	3.27E-05	0.009141
Owenia_fusiformis	OFUSG17182.1	10951.58	-0.5757	0.075641	-7.6108	2.72E-14	9.45E-11
Owenia_fusiformis	OFUSG17248.2	2328.199	-0.68897	0.112302	-6.12456	9.09E-10	1.38E-06
Owenia_fusiformis	OFUSG17661.2	1406.978	-0.63152	0.121725	-5.22678	1.72E-07	0.000123
Owenia_fusiformis	OFUSG19170.2	2702.823	-0.69204	0.116497	-6.04746	1.47E-09	2.10E-06
Owenia_fusiformis	OFUSG19375.2	1463.345	-0.50296	0.124199	-4.14193	3.44E-05	0.009506
Owenia_fusiformis	OFUSG20191.1	2136.699	-0.56554	0.11567	-4.9026	9.46E-07	0.00051
Owenia_fusiformis	OFUSG21500.1	449.5009	-0.7999	0.111434	-8.40126	4.42E-17	2.15E-13
Owenia_fusiformis	OFUSG25600.1	3928.322	-0.50274	0.100808	-4.9887	6.08E-07	0.000343

Supplementary Table 12. Differentially upregulated genes in the blastula (6 hpf) of *O. fusiformis* after rBMP4 treatment.

Species	Gene_ID	basal_mean	log2FoldChange	lfcSE	stat	pvalue	padj
Owenia_fusiformis	OFUSG00511.1	499.0948	1.386601	0.202732	6.818919	9.17E-12	8.49E-09
Owenia_fusiformis	OFUSG00637.1	180.8984	4.893693	0.365214	12.37143	3.73E-35	2.76E-31
Owenia_fusiformis	OFUSG00681.2	138.0529	1.126988	0.283796	3.957344	7.58E-05	0.012808
Owenia_fusiformis	OFUSG01191.1	76.14384	1.992068	0.435181	4.134976	3.55E-05	0.007366
Owenia_fusiformis	OFUSG01380.1	338.339	1.710753	0.296482	5.66275	1.49E-08	6.49E-06
Owenia_fusiformis	OFUSG01473.1	1016.293	1.282737	0.266135	4.783907	1.72E-06	0.000466
Owenia_fusiformis	OFUSG01611.1	3064.941	1.00418	0.180092	5.568287	2.57E-08	1.02E-05
Owenia_fusiformis	OFUSG01612.1	24.06521	1.969721	0.448091	4.037775	5.40E-05	0.010328
Owenia_fusiformis	OFUSG01867.1	735.778	1.755351	0.181859	9.626225	6.20E-22	1.72E-18
Owenia_fusiformis	OFUSG02824.1	2155.813	1.113479	0.208324	5.334242	9.59E-08	3.49E-05
Owenia_fusiformis	OFUSG03913.1	465.5027	1.580829	0.211542	7.443661	9.79E-14	1.21E-10
Owenia_fusiformis	OFUSG04334.1	180.0903	1.648946	0.369075	4.328867	1.50E-05	0.003361
Owenia_fusiformis	OFUSG04918.1	47.56478	3.560023	0.448241	6.272671	3.55E-10	2.46E-07
Owenia_fusiformis	OFUSG05075.1	47.48526	1.617131	0.364485	4.240151	2.23E-05	0.004898
Owenia_fusiformis	OFUSG05120.1	78.93327	1.20308	0.318441	3.768107	0.000164	0.023832
Owenia_fusiformis	OFUSG05669.1	430.1974	1.628484	0.211274	7.688687	1.49E-14	2.20E-11
Owenia_fusiformis	OFUSG05672.1	670.2907	2.398845	0.206162	11.60281	3.99E-31	2.21E-27
Owenia_fusiformis	OFUSG05673.1	29.41661	1.716158	0.395087	4.099223	4.15E-05	0.008292
Owenia_fusiformis	OFUSG05709.1	2101.984	1.788911	0.112511	15.89472	6.89E-57	1.53E-52
Owenia_fusiformis	OFUSG05920.1	2243.315	1.119976	0.101134	11.07284	1.70E-28	6.29E-25
Owenia_fusiformis	OFUSG05949.1	36.17505	2.001158	0.442983	4.338785	1.43E-05	0.003246
Owenia_fusiformis	OFUSG06182.1	286.8513	1.23388	0.310302	3.925264	8.66E-05	0.014249
Owenia_fusiformis	OFUSG06474.2	1827.976	1.764086	0.287411	6.062418	1.34E-09	7.63E-07
Owenia_fusiformis	OFUSG07729.1	521.9598	1.412855	0.300503	4.647597	3.36E-06	0.000849
Owenia_fusiformis	OFUSG08271.1	64.37793	2.497868	0.372404	6.53735	6.26E-11	4.79E-08
Owenia_fusiformis	OFUSG08948.1	82.60123	1.849441	0.434941	3.992271	6.54E-05	0.011718
Owenia_fusiformis	OFUSG10129.1	79.3377	2.167897	0.316839	6.723843	1.77E-11	1.57E-08
Owenia_fusiformis	OFUSG10204.1	63.11349	1.311412	0.329795	3.899781	9.63E-05	0.015604
Owenia_fusiformis	OFUSG10748.1	95.60598	3.403692	0.391252	8.167941	3.14E-16	5.36E-13
Owenia_fusiformis	OFUSG11014.1	610.2581	1.902461	0.20099	9.428942	4.14E-21	1.02E-17
Owenia_fusiformis	OFUSG11630.1	236.156	1.666833	0.416093	3.724087	0.000196	0.027373
Owenia_fusiformis	OFUSG11793.1	90.33635	3.153203	0.448459	5.142981	2.70E-07	9.10E-05
Owenia_fusiformis	OFUSG12458.1	9671.454	1.192149	0.136651	8.719459	2.80E-18	6.21E-15
Owenia_fusiformis	OFUSG12638.1	8998.748	1.319307	0.263291	4.997362	5.81E-07	0.000182
Owenia_fusiformis	OFUSG12738.1	340.6483	1.448432	0.254555	5.654294	1.56E-08	6.68E-06
Owenia_fusiformis	OFUSG12820.2	25.9914	1.624435	0.441651	4.660721	3.15E-06	0.000814
Owenia_fusiformis	OFUSG13379.1	162.4719	1.098029	0.232878	4.694735	2.67E-06	0.000714
Owenia_fusiformis	OFUSG13380.1	6403.665	1.01712	0.163926	6.193187	5.90E-10	3.97E-07
Owenia_fusiformis	OFUSG14328.1	134.6686	1.503554	0.327884	4.537968	5.68E-06	0.001386
Owenia_fusiformis	OFUSG15779.1	48.09032	1.575571	0.409729	3.665246	0.000247	0.032657
Owenia_fusiformis	OFUSG15932.1	45.98146	1.403199	0.345504	3.984753	6.76E-05	0.011903
Owenia_fusiformis	OFUSG16598.2	336.0428	1.141187	0.30133	3.730758	0.000191	0.026962
Owenia_fusiformis	OFUSG16664.1	5077.552	2.071374	0.135854	15.24073	1.90E-52	2.11E-48
Owenia_fusiformis	OFUSG17833.1	70.87482	1.63397	0.375184	4.176094	2.97E-05	0.006212
Owenia_fusiformis	OFUSG18037.1	1559.582	1.092151	0.202292	5.403642	6.53E-08	2.50E-05
Owenia_fusiformis	OFUSG18388.1	2558.06	3.112144	0.441926	5.769516	7.95E-09	3.68E-06
Owenia_fusiformis	OFUSG18487.1	30.35747	1.47256	0.429593	3.67165	0.000241	0.032233
Owenia_fusiformis	OFUSG18529.1	84.51942	2.305086	0.358789	6.1763	6.56E-10	4.29E-07
Owenia_fusiformis	OFUSG18600.1	98.34326	1.765925	0.303037	5.719962	1.07E-08	4.83E-06
Owenia_fusiformis	OFUSG18918.1	114.9702	2.022171	0.448495	3.684493	0.000229	0.031407
Owenia_fusiformis	OFUSG19295.1	12.72023	2.011801	0.444381	3.999764	6.34E-05	0.011635
Owenia_fusiformis	OFUSG20330.1	2192.005	1.818862	0.278714	6.451118	1.11E-10	8.22E-08
Owenia_fusiformis	OFUSG20586.1	43.47001	1.590189	0.446985	4.808828	1.52E-06	0.000427
Owenia_fusiformis	OFUSG20598.1	376.3126	1.798876	0.272691	6.571389	4.98E-11	3.95E-08
Owenia_fusiformis	OFUSG20670.1	48.1622	1.664913	0.367357	4.51952	6.20E-06	0.001496
Owenia_fusiformis	OFUSG21113.1	37.85557	2.642106	0.43469	5.848703	4.95E-09	2.39E-06
Owenia_fusiformis	OFUSG21302.1	1863.353	1.336707	0.219405	6.073082	1.25E-09	7.33E-07
Owenia_fusiformis	OFUSG21501.1	429.2641	1.25322	0.247262	5.052407	4.36E-07	0.00014
Owenia_fusiformis	OFUSG22193.1	45.8021	1.934665	0.437343	3.753814	0.000174	0.024948
Owenia_fusiformis	OFUSG22766.1	139.5634	1.821991	0.258424	7.00789	2.42E-12	2.56E-09
Owenia_fusiformis	OFUSG24251.1	42.21948	2.471956	0.431116	5.190472	2.10E-07	7.28E-05
Owenia_fusiformis	OFUSG25514.1	68.96767	2.117469	0.396076	5.019016	5.19E-07	0.000165
Owenia_fusiformis	OFUSG26088.1	32.30056	1.160349	0.428462	4.578219	4.69E-06	0.001157
Owenia_fusiformis	OFUSG26148.3	10.73053	1.388127	0.423975	3.970102	7.18E-05	0.012365
Owenia_fusiformis	OFUSG26267.1	3158.602	1.741319	0.301628	5.702676	1.18E-08	5.24E-06
Owenia_fusiformis	OFUSG26268.1	1252.839	1.808451	0.302304	5.915446	3.31E-09	1.71E-06
Owenia_fusiformis	OFUSG26690.1	162.0485	1.362821	0.273648	4.932872	8.10E-07	0.00024
Owenia_fusiformis	OFUSG26959.3	121.3454	3.486812	0.435754	7.648056	2.04E-14	2.83E-11

Supplementary Table 13. Differentially downregulated genes in the blastula (6 hpf) of *O. fusiformis* after rBMP4 treatment.

Species	Gene_ID	basal_mean	log2FoldChange	lfcSE	stat	pvalue	padj
Owenia_fusiformis	OFUSG00597.1	39.36558	-1.52232	0.448505	-3.55184	0.000383	0.047988
Owenia_fusiformis	OFUSG01533.1	165.0923	-1.44709	0.313134	-4.60757	4.07E-06	0.001016
Owenia_fusiformis	OFUSG01750.1	1888.963	-1.33968	0.170426	-7.85452	4.01E-15	6.36E-12
Owenia_fusiformis	OFUSG01771.1	277.2109	-2.49705	0.447159	-5.62505	1.85E-08	7.63E-06
Owenia_fusiformis	OFUSG03002.2	21.01921	-1.09063	0.447077	-3.83622	0.000125	0.019269
Owenia_fusiformis	OFUSG05565.1	1685.958	-1.3109	0.316242	-4.11811	3.82E-05	0.007781
Owenia_fusiformis	OFUSG07318.2	1498.713	-1.04657	0.156663	-6.67829	2.42E-11	2.06E-08
Owenia_fusiformis	OFUSG07365.1	150.0249	-1.68759	0.393943	-4.18531	2.85E-05	0.006022
Owenia_fusiformis	OFUSG07809.1	2044.206	-1.02328	0.213899	-4.79668	1.61E-06	0.000442
Owenia_fusiformis	OFUSG08557.1	114.2393	-1.337	0.331581	-4.07586	4.58E-05	0.009022
Owenia_fusiformis	OFUSG08558.1	255.4858	-1.20091	0.29655	-4.04899	5.14E-05	0.009961
Owenia_fusiformis	OFUSG08631.1	1563.373	-1.16327	0.237856	-4.88337	1.04E-06	0.000301
Owenia_fusiformis	OFUSG09552.1	333.6347	-1.29054	0.328788	-3.90312	9.50E-05	0.015503
Owenia_fusiformis	OFUSG09642.1	5915.594	-1.0876	0.164647	-6.60687	3.93E-11	3.23E-08
Owenia_fusiformis	OFUSG09693.1	1287.333	-3.00097	0.302836	-9.91131	3.72E-23	1.18E-19
Owenia_fusiformis	OFUSG09750.1	54.55189	-1.37887	0.336695	-4.02285	5.75E-05	0.010819
Owenia_fusiformis	OFUSG10344.1	1688.143	-2.33233	0.33518	-6.95454	3.54E-12	3.57E-09
Owenia_fusiformis	OFUSG10608.1	619.7061	-1.47005	0.282281	-5.1618	2.45E-07	8.35E-05
Owenia_fusiformis	OFUSG11126.1	237.9301	-1.45708	0.357805	-3.82773	0.000129	0.019803
Owenia_fusiformis	OFUSG11338.1	42.64328	-1.76759	0.447484	-3.61676	0.000298	0.038066
Owenia_fusiformis	OFUSG11550.1	605.3865	-1.42316	0.304991	-4.66617	3.07E-06	0.000802
Owenia_fusiformis	OFUSG11955.1	281.7888	-1.13427	0.28612	-3.98929	6.63E-05	0.011771
Owenia_fusiformis	OFUSG12159.1	1310.933	-1.8904	0.225377	-8.41497	3.93E-17	7.27E-14
Owenia_fusiformis	OFUSG13069.1	4157.486	-1.13537	0.228921	-4.95246	7.33E-07	0.00022
Owenia_fusiformis	OFUSG13105.1	9649.095	-1.10763	0.174999	-6.32508	2.53E-10	1.81E-07
Owenia_fusiformis	OFUSG13593.1	240.4234	-1.63986	0.31142	-5.24814	1.54E-07	5.50E-05
Owenia_fusiformis	OFUSG14953.1	470.5637	-1.19521	0.319238	-3.78037	0.000157	0.023026
Owenia_fusiformis	OFUSG15092.1	58.69484	-1.34154	0.355017	-3.73702	0.000186	0.026503
Owenia_fusiformis	OFUSG15355.1	469.6221	-1.49139	0.304107	-4.86258	1.16E-06	0.00033
Owenia_fusiformis	OFUSG16033.1	79.4402	-1.27481	0.331409	-3.86074	0.000113	0.017676
Owenia_fusiformis	OFUSG17445.1	10063.26	-1.15021	0.28585	-3.99466	6.48E-05	0.011695
Owenia_fusiformis	OFUSG17486.1	149.1404	-1.32917	0.294466	-4.47566	7.62E-06	0.001799
Owenia_fusiformis	OFUSG17729.1	706.853	-1.04964	0.175821	-5.96736	2.41E-09	1.30E-06
Owenia_fusiformis	OFUSG18179.2	19.99433	-1.13508	0.395934	-4.07551	4.59E-05	0.009022
Owenia_fusiformis	OFUSG19707.1	735.2171	-1.14779	0.312615	-3.67686	0.000236	0.031774
Owenia_fusiformis	OFUSG21342.1	1833.042	-1.87994	0.221653	-8.47839	2.28E-17	4.61E-14
Owenia_fusiformis	OFUSG21527.1	4008.546	-1.41749	0.22981	-6.1585	7.34E-10	4.66E-07
Owenia_fusiformis	OFUSG21806.1	487.7784	-2.13327	0.442093	-5.65066	1.60E-08	6.70E-06
Owenia_fusiformis	OFUSG22325.1	433.1975	-1.04812	0.27974	-3.76688	0.000165	0.023832
Owenia_fusiformis	OFUSG22325.2	247.6685	-1.40411	0.289685	-4.8877	1.02E-06	0.000298
Owenia_fusiformis	OFUSG22615.1	92.56045	-1.86444	0.436604	-4.28776	1.80E-05	0.004007
Owenia_fusiformis	OFUSG23324.1	420.4335	-1.53	0.342052	-4.44479	8.80E-06	0.002014
Owenia_fusiformis	OFUSG23343.1	143.869	-1.12227	0.294578	-3.87768	0.000105	0.016644
Owenia_fusiformis	OFUSG23836.1	1068.082	-1.26664	0.31658	-3.98098	6.86E-05	0.011998
Owenia_fusiformis	OFUSG24639.1	270.0216	-1.10094	0.267666	-4.10317	4.08E-05	0.008226
Owenia_fusiformis	OFUSG25241.1	1562.693	-1.83046	0.252426	-7.27133	3.56E-13	4.16E-10
Owenia_fusiformis	OFUSG25551.1	963.607	-1.57984	0.303064	-5.23764	1.63E-07	5.73E-05
Owenia_fusiformis	OFUSG25552.1	58.17333	-1.4177	0.403779	-3.64982	0.000262	0.034074
Owenia_fusiformis	OFUSG26269.1	410.854	-2.15446	0.425228	-4.97169	6.64E-07	0.000205
Owenia_fusiformis	OFUSG26322.5	329.9742	-2.09054	0.391277	-5.8567	4.72E-09	2.33E-06
Owenia_fusiformis	OFUSG26371.1	405.0904	-1.86911	0.37606	-4.96859	6.74E-07	0.000205
Owenia_fusiformis	OFUSG26435.1	681.2926	-1.07968	0.194177	-5.58053	2.40E-08	9.68E-06
Owenia_fusiformis	OFUSG26437.1	1024.698	-1.67178	0.282504	-5.89542	3.74E-09	1.89E-06

Supplementary Table 14. Scoring of the expression of candidate genes at the blastula stage of *O. fusiformis* after DMH1 treatment.

Species	Treatment	Total_n	*fer3*	*delta*	*rhox1*
Owenia_fusiformis	0.2% DMSO	108	7	71	30
Owenia_fusiformis	20uM DMH1	83	9	48	26

Supplementary Table 15. Scoring of the expression of candidate genes at the blastula stage of *O. fusiformis* after rBMP4 and rActivin A treatment.

Species	Treatment	Total_n	*wnt5*	*foxA*	*msx*	*foxG*	*bmp2/4*	*osr*	*six3/6*	*zag1*	*rx1*	*fojo*	*chordin*	*gdf*	*cboc1*
Owenia_fusiformis	1x Mock		22	19	58	35	14	28	36	8	7	11	22	8	13
Owenia_fusiformis	1000 ng/ml rBMP		142	17	247	78	24	62	NA	NA	NA	9	99	22	6
Owenia_fusiformis	75 ng/ml rActivin A		66	46	49	63	18	24	14	28	39	17	15	NA	24

Supplementary Table 16. Scoring of *syt1*, *elav1* and *six3/6* expression in larvae of *O. fusiformis* after DMH1, SB431542, rBMP4 and rActivin A treatment.

Species	Treatment	Total_n	syt1	elav1	six3/6
Owenia_fusiformis	0.2% DMSO	116	74	21	21
Owenia_fusiformis	20uM DMH1	157	52	53	52
Owenia_fusiformis	1x Mock	70	5	52	13
Owenia_fusiformis	1000 ng/ml rBMP	35	5	15	15
Owenia_fusiformis	0.4% DMSO	8	8	NA	NA
Owenia_fusiformis	40uM SB431542	122	122	NA	NA
Owenia_fusiformis	1x Mock	15	15	NA	NA
Owenia_fusiformis	75 ng/ml rActivin A	10	10	NA	NA
Owenia_fusiformis	0.2% DMSO	250	250	NA	NA
Owenia_fusiformis	20uM DMH1	182	182	NA	NA
Owenia_fusiformis	1x Mock	174	174	NA	NA
Owenia_fusiformis	1000 ng/ml rBMP	58	58	NA	NA
Owenia_fusiformis	75 ng/ml rActivin A	63	63	NA	NA
Owenia_fusiformis	0.4% DMSO	128	128	NA	NA
Owenia_fusiformis	40uM SB431542	79	79	NA	NA

Supplementary Table 17. Scoring of RYamide-like immunoreactivity in larvae of *O. fusiformis* after DMH1, SB431542, rBMP4 and rActivin A treatment.

Species	Treatment	Total_n_RYamide_phenotype
Owenia_fusiformis	0.2% DMSO	5
Owenia_fusiformis	20 uM DMH1	5
Owenia_fusiformis	1x Mock	18
Owenia_fusiformis	1000 ng/ml rBMP	17
Owenia_fusiformis	75 ng/ml rActivin A	19
Owenia_fusiformis	0.4% DMSO	15
Owenia_fusiformis	40 uM SB431542	25

Supplementary Table 18. Scoring of dp-ERK1/2 immunoreactivity in blastulae of *S. lamarcki* treated with U0126 and DMH1.

Species	Treatment	Total_n	dpERK1/2_positive	% dpERK1/2_positive
Spirobranchus_lamarcki	0.75% DMSO	159	141	0.88679245
Spirobranchus_lamarcki	10uM DMH1	64	46	0.71875
Spirobranchus_lamarcki	75uM U0126	99	3	0.03030303

Supplementary Table 19. Scoring of pSMAD1/5/8 immunoreactivity in blastulae of *S. lamarcki* treated with U0126 and DMH1.

Species	Treatment	Total_n	pSMAD1/5/8 positive	% pSMAD1/5/8 positive
Spirobranchus_lamarcki	0.75% DMSO	92	70	0.76086957
Spirobranchus_lamarcki	10uM DMH1	16	0	0
Spirobranchus_lamarcki	75uM U0126	33	0	0

Supplementary Table 20. Scoring of dp-ERK1/2 immunoreactivity in blastulae of *S. lamarcki* treated with DMH1.

Species	Treatment	Replicate	Total_n	dpERK1/2 positive	Positive proportion
Spirobranchus_lamarcki	0.1% DMSO	1	164	133	0.810976
Spirobranchus_lamarcki	0.1% DMSO	2	193	152	0.787565
Spirobranchus_lamarcki	0.1% DMSO	3	205	168	0.819512
Spirobranchus_lamarcki	10uM DMH1	1	99	76	0.767677
Spirobranchus_lamarcki	10uM DMH1	2	284	238	0.838028
Spirobranchus_lamarcki	10uM DMH1	3	224	181	0.808036

Supplementary Table 21. Scoring of morphological phenotypes in larvae of *S. lamarcki* after U0126, DMH1 and rBMP4 treatments.

Species	Treatment	Total_n
Spirobranchus_lamarcki	1% DMSO	40
Spirobranchus_lamarcki	75 uM U0126	177
Spirobranchus_lamarcki	10 uM DMH1	57
Spirobranchus_lamarcki	2x Mock	48
Spirobranchus_lamarcki	1000 ng/ml rBMP	14

Supplementary Table 22. Scoring of pSMAD1/5/8 immunoreactivity in blastulae of *S. lamarcki* treated with rBMP4.

Species	Treatment	Total_n
Spirobranchus_lamarcki	2x Mock	45
Spirobranchus_lamarcki	100 ng/ml rBMP	12
Spirobranchus_lamarcki	250 ng/ml rBMP	39
Spirobranchus_lamarcki	500 ng/ml rBMP	38
Spirobranchus_lamarcki	1000 ng/ml rBMP	17
Spirobranchus_lamarcki	2000 ng/ml rBMP	37

Supplementary Table 23. Dose-response of DMH1 and rBMP treatments on the morphology of 72 hpf *P. dumerilii* larvae.

Species	Treatment	Total n batch 1	Normal	Abnormal	% normal batch 1	Total n batch 2	Normal	Abnormal	% normal Batch 2	Notes
Platynereis_ dumerilii	0.5% DMSO	35	31	4	0.88	36	34	2	0.94	All larvae exhibited six eyes (2 larval, 4 adult) and normal overall morphology
Platynereis_ dumerilii	1 uM DMH1	47	42	5	0.89	30	27	3	0.9	Larvae showed variable eye numbers (six, four, or one) with overall normal morphology
Platynereis_ dumerilii	5 uM DMH1	23	20	3	0.86	28	26	2	0.92	All larvae had a single eye and displayed relatively mild morphological abnormalities
Platynereis_ dumerilii	7.5 uM DMH1	35	31	4	0.88	32	31	1	0.96	All larvae had a single eye and displayed relatively mild morphological abnormalities
Platynereis_ dumerilii	10 uM DMH1	39	38	1	0.97	34	32	2	0.94	All larvae had a single eye and showed milder morphological defects compared with the 20 µM condition
Platynereis_ dumerilii	20 uM DMH1	52	48	4	0.92	34	32	2	0.94	All larvae lacked eyes and displayed a severely abnormal morphology
Platynereis_ dumerilii	50 uM DMH1	21	21	0	1	33	33	0	1	All larvae lacked eyes and displayed a severely abnormal morphology
Platynereis_ dumerilii	2x Mock	36	32	4	0.888889	25	24	1	0.96	All larvae with six eyes (2 larval, 4 adult) and one stomodeum
Platynereis_ dumerilii	100 ng/ml rBMP	27	25	2	0.925926	38	36	2	0.947368	All larvae with six eyes (2 larval, 4 adult) and one stomodeum
Platynereis_ dumerilii	200 ng/ml rBMP	28	27	1	0.964286	23	21	2	0.913043	All larvae with six eyes (2 larval, 4 adult) and one stomodeum
Platynereis_ dumerilii	500 ng/ml rBMP	34	33	1	0.970588	27	27	0	1	Variation of number of larval, adult eyes and stomodeum
Platynereis_ dumerilii	1000 ng/ml rBMP	30	24	6	0.8	26	23	3	0.884615	Variation of number of larval, adult eyes and stomodeum

Platynereis_ dumerilii	2000 ng/ml rBMP	19	19	0	1	23	22	1	0.956522	All larvae exhibited four adult eyes and two stomodea

Supplementary Table 24. Differentially downregulated genes in 8.5 hpf embryos of *P. dumerilii* after DMH1 treatment.

Species	Gene	baseMean	log2FoldChange	lfcSE	pvalue	padj
Platynereis_dumerilii	XLOC_000913	1024.409	-3.22932	0.126328	1.18E-144	2.26E-140
Platynereis_dumerilii	XLOC_002365	132.8028	-3.69068	0.367986	2.21E-24	4.71E-21
Platynereis_dumerilii	XLOC_003846	18.6736	-2.56246	1.115611	1.33E-05	0.002118226
Platynereis_dumerilii	XLOC_005048	1869.777	-1.35278	0.088686	1.22E-52	7.82E-49
Platynereis_dumerilii	XLOC_005213	325.0936	-1.70253	0.192173	5.69E-19	7.29E-16
Platynereis_dumerilii	XLOC_005674	643.7142	-1.35863	0.12987	9.68E-26	2.33E-22
Platynereis_dumerilii	XLOC_005757	48.88534	-1.93208	0.454965	1.01E-06	0.000206016
Platynereis_dumerilii	XLOC_006924	16.95161	-3.40942	1.310056	4.80E-06	0.000853122
Platynereis_dumerilii	XLOC_006926	12.00961	-2.03014	1.995048	5.18E-05	0.007418932
Platynereis_dumerilii	XLOC_006927	24.08507	-4.11696	1.103235	6.00E-07	0.000127982
Platynereis_dumerilii	XLOC_006929	50.22062	-2.65955	0.458178	2.59E-09	9.55E-07
Platynereis_dumerilii	XLOC_007612	233.7733	-1.26085	1.492788	0.000181534	0.02235428
Platynereis_dumerilii	XLOC_007672	87.7355	-1.75248	0.489245	2.83E-06	0.000517875
Platynereis_dumerilii	XLOC_008264	23.4443	-3.49962	0.846145	6.48E-07	0.00013678
Platynereis_dumerilii	XLOC_008265	51.48353	-3.32485	1.746534	8.22E-06	0.001385272
Platynereis_dumerilii	XLOC_008608	22.41558	-3.3425	0.784497	5.87E-07	0.000126699
Platynereis_dumerilii	XLOC_008609	18.9538	-2.24574	1.495626	3.91E-05	0.005692376
Platynereis_dumerilii	XLOC_011874	104.2241	-3.47247	0.405807	2.69E-18	3.22E-15
Platynereis_dumerilii	XLOC_012051	55.21815	-3.50307	0.496084	3.58E-13	2.15E-10
Platynereis_dumerilii	XLOC_012701	36.76646	-2.45973	0.541626	5.23E-07	0.00011684
Platynereis_dumerilii	XLOC_012788	35.37498	-3.37505	0.5898	2.35E-09	8.85E-07
Platynereis_dumerilii	XLOC_012790	24.93436	-3.96903	0.862631	1.47E-07	3.62E-05
Platynereis_dumerilii	XLOC_013606	257.0431	-2.57488	0.433278	1.21E-09	4.74E-07
Platynereis_dumerilii	XLOC_013607	7.656315	-1.18146	1.915928	9.60E-05	0.012719006
Platynereis_dumerilii	XLOC_013608	11.91516	-1.36495	1.709115	0.000150259	0.018865811
Platynereis_dumerilii	XLOC_013784	46.33134	-2.95468	0.515047	3.09E-09	1.12E-06
Platynereis_dumerilii	XLOC_013785	81.39301	-3.09448	0.422196	7.17E-14	5.10E-11
Platynereis_dumerilii	XLOC_014505	137.3686	-1.30721	0.252066	3.46E-08	9.92E-06
Platynereis_dumerilii	XLOC_021421	219.9976	-2.1058	0.772554	9.21E-06	0.00151179
Platynereis_dumerilii	XLOC_022166	303.4575	-2.05425	0.176708	1.88E-31	6.01E-28
Platynereis_dumerilii	XLOC_022438	258.0276	-1.2791	0.17981	5.25E-13	3.05E-10
Platynereis_dumerilii	XLOC_022618	31.85417	-2.18491	1.013071	1.86E-05	0.002855582
Platynereis_dumerilii	XLOC_024211	50.59119	-3.68021	0.513043	1.35E-13	8.67E-11
Platynereis_dumerilii	XLOC_025705	367.584	-1.0557	0.167619	2.11E-11	1.07E-08
Platynereis_dumerilii	XLOC_026040	406.6283	-2.10672	0.362512	3.35E-09	1.16E-06
Platynereis_dumerilii	XLOC_026365	1025.345	-4.48337	0.495132	1.14E-20	1.69E-17
Platynereis_dumerilii	XLOC_033183	16.16979	-1.89043	1.616203	7.18E-05	0.009780053
Platynereis_dumerilii	XLOC_033457	48.55329	-3.65977	0.562333	1.39E-11	7.21E-09
Platynereis_dumerilii	XLOC_033486	395.5169	-1.12967	0.145222	1.87E-15	1.71E-12
Platynereis_dumerilii	XLOC_035503	1082.117	-2.57612	0.110226	3.88E-121	3.73E-117
Platynereis_dumerilii	XLOC_036060	118.2604	-2.82405	0.360253	1.74E-15	1.67E-12
Platynereis_dumerilii	XLOC_036065	159.3133	-2.99236	0.825256	2.10E-06	0.000399856
Platynereis_dumerilii	XLOC_036179	20.51305	-1.00239	1.299777	0.000259775	0.030244113
Platynereis_dumerilii	XLOC_036464	20.12775	-3.65766	1.030506	1.34E-06	0.000264836
Platynereis_dumerilii	XLOC_036746	25.27261	-3.25923	0.871124	1.48E-06	0.000291034
Platynereis_dumerilii	XLOC_038321	293.7138	-2.95757	0.238628	1.01E-35	4.84E-32
Platynereis_dumerilii	XLOC_038323	320.3991	-1.55128	0.210989	1.35E-13	8.67E-11
Platynereis_dumerilii	XLOC_039734	169.862	-3.09203	1.16149	6.21E-06	0.00107395
Platynereis_dumerilii	XLOC_041487	587.403	-2.56706	0.318609	3.55E-16	4.01E-13
Platynereis_dumerilii	XLOC_041872	78.77302	-1.39236	0.537505	9.39E-06	0.00152827
Platynereis_dumerilii	XLOC_042671	27.57603	-3.5884	0.710393	5.20E-08	1.45E-05
Platynereis_dumerilii	XLOC_042673	45.37078	-4.54404	0.620203	1.66E-14	1.33E-11
Platynereis_dumerilii	XLOC_042674	26.55148	-4.50361	0.84309	4.63E-09	1.51E-06
Platynereis_dumerilii	XLOC_043659	60.15025	-1.66455	0.665649	1.20E-05	0.00193763
Platynereis_dumerilii	XLOC_043758	161.9262	-2.48561	0.261058	8.24E-22	1.58E-18
Platynereis_dumerilii	XLOC_043773	158.4516	-2.4641	0.259673	1.14E-21	1.98E-18
Platynereis_dumerilii	XLOC_043853	75.42312	-2.07728	0.365224	6.78E-09	2.17E-06
Platynereis_dumerilii	XLOC_043854	175.2795	-2.72215	0.548292	1.39E-07	3.47E-05
Platynereis_dumerilii	XLOC_043938	61.29397	-1.30723	0.689241	2.58E-05	0.00380673
Platynereis_dumerilii	XLOC_043956	52.77212	-2.69672	0.447679	6.83E-10	2.85E-07
Platynereis_dumerilii	XLOC_044417	69.709	-1.19371	0.63747	2.52E-05	0.003747651

Platynereis_dumerilii	XLOC_045004	92.05439	-1.9383	0.359966	3.25E-08	9.45E-06
Platynereis_dumerilii	XLOC_045810	206.8458	-3.66207	0.699235	2.19E-08	6.67E-06
Platynereis_dumerilii	XLOC_049923	64.40345	-1.86083	0.406607	4.95E-07	0.000113113
Platynereis_dumerilii	XLOC_051599	24.3453	-3.4227	0.749017	2.78E-07	6.68E-05
Platynereis_dumerilii	XLOC_051694	147.9072	-2.37283	0.251304	1.88E-21	3.01E-18
Platynereis_dumerilii	XLOC_052035	200.2986	-1.36415	0.201263	6.51E-12	3.47E-09
Platynereis_dumerilii	XLOC_052601	446.5154	-2.1693	0.176475	5.75E-35	2.21E-31
Platynereis_dumerilii	XLOC_054281	175.4801	-1.95234	0.264616	1.02E-13	6.96E-11
Platynereis_dumerilii	XLOC_056200	531.1515	-1.01879	0.139685	2.07E-14	1.59E-11
Platynereis_dumerilii	XLOC_057591	17.7181	-2.67724	1.555924	2.16E-05	0.003238192
Platynereis_dumerilii	XLOC_057592	13.28262	-1.92376	1.824884	6.85E-05	0.009635819
Platynereis_dumerilii	XLOC_057593	51.56654	-3.60905	1.769863	4.90E-06	0.00086325
Platynereis_dumerilii	XLOC_057959	124.8387	-3.10341	1.278956	7.76E-06	0.001319303
Platynereis_dumerilii	XLOC_058034	180.7565	-1.55443	0.226683	4.67E-12	2.64E-09
Platynereis_dumerilii	XLOC_059186	77.60605	-3.29511	0.432053	6.39E-15	5.34E-12
Platynereis_dumerilii	XLOC_059564	43.42305	-2.57101	0.566181	5.04E-07	0.000114002
Platynereis_dumerilii	XLOC_059755	30.43705	-2.79821	0.59463	2.99E-07	7.09E-05
Platynereis_dumerilii	XLOC_059780	357.116	-1.78858	0.200816	3.60E-19	4.95E-16
Platynereis_dumerilii	XLOC_062459	45.38576	-3.94221	0.642221	1.10E-10	5.14E-08
Platynereis_dumerilii	XLOC_065187	16.74831	-1.23298	1.613233	0.000189315	0.023163984
Platynereis_dumerilii	XLOC_067817	196.4726	-1.89754	0.236045	5.90E-16	5.97E-13

Supplementary Table 25. Differentially upregulated genes in 8.5 hpf embryos of *P. dumerilii* after DMH1 treatment.

Species	Gene	baseMean	log2FoldChange	lfcSE	pvalue	padj
Platynereis_dumerilii	XLOC_004355	78.05951	2.425937	0.418681	3.17E-09	1.13E-06
Platynereis_dumerilii	XLOC_003650	37.22448	1.276686	0.954806	7.15E-05	0.00978
Platynereis_dumerilii	XLOC_060446	1481.168	1.024353	0.095314	9.81E-28	2.69E-24

Supplementary Table 26. Differentially downregulated genes in 12 hpf embryos of *P. dumerilii* after DMH1 treatment.

Species	Gene	baseMean	log2FoldChange	lfcSE	pvalue	padj
Platynereis_dumerilii	XLOC_000905	59.00689	-3.91146	0.604483	2.24E-11	1.60E-08
Platynereis_dumerilii	XLOC_000913	846.1165	-1.48316	0.230242	9.66E-11	6.06E-08
Platynereis_dumerilii	XLOC_001063	23.53816	-4.59113	0.980802	1.34E-07	4.35E-05
Platynereis_dumerilii	XLOC_001890	16.69563	-3.10966	2.116628	3.17E-05	0.00568
Platynereis_dumerilii	XLOC_002213	71.02457	-4.4603	0.564503	4.19E-16	5.61E-13
Platynereis_dumerilii	XLOC_002291	382.119	-2.0512	0.24717	7.20E-17	1.11E-13
Platynereis_dumerilii	XLOC_003809	34.85869	-2.322	0.680272	5.75E-06	0.001255
Platynereis_dumerilii	XLOC_003860	43.52038	-2.22759	0.488998	7.88E-07	0.000214
Platynereis_dumerilii	XLOC_003971	3202.154	-1.21032	0.233788	1.17E-07	3.92E-05
Platynereis_dumerilii	XLOC_004128	140.9369	-1.32829	0.643927	2.97E-05	0.005418
Platynereis_dumerilii	XLOC_004566	1389.75	-1.48058	0.234786	2.34E-10	1.42E-07
Platynereis_dumerilii	XLOC_004616	66.66355	-3.23715	0.484166	8.58E-12	6.89E-09
Platynereis_dumerilii	XLOC_004695	23.33914	-1.7292	1.326467	0.000113	0.017377
Platynereis_dumerilii	XLOC_005006	17.54149	-1.16995	1.437111	0.000348	0.048594
Platynereis_dumerilii	XLOC_005213	362.1932	-1.05297	0.396837	1.09E-05	0.002262
Platynereis_dumerilii	XLOC_005270	180.1434	-4.68798	0.729461	1.47E-11	1.09E-08
Platynereis_dumerilii	XLOC_005592	103.4562	-2.28944	0.676799	6.01E-06	0.001298
Platynereis_dumerilii	XLOC_005674	764.3842	-1.78836	0.339643	7.82E-08	2.80E-05
Platynereis_dumerilii	XLOC_005720	486.488	-2.72019	0.315958	3.75E-18	7.38E-15
Platynereis_dumerilii	XLOC_006924	333.5118	-4.82749	0.721636	2.23E-12	1.95E-09
Platynereis_dumerilii	XLOC_006925	367.3101	-4.46834	0.699624	2.37E-11	1.64E-08
Platynereis_dumerilii	XLOC_006928	244.7655	-5.95333	0.660558	6.58E-21	1.89E-17
Platynereis_dumerilii	XLOC_006929	683.7735	-4.20811	0.739438	2.10E-09	1.03E-06
Platynereis_dumerilii	XLOC_007096	24.34383	-4.65616	0.956927	6.81E-08	2.53E-05
Platynereis_dumerilii	XLOC_007672	819.3215	-4.04706	0.675618	4.17E-10	2.27E-07
Platynereis_dumerilii	XLOC_008265	912.1197	-1.2478	1.748886	0.000355	0.048796
Platynereis_dumerilii	XLOC_008605	326.6732	-4.13605	0.689083	3.59E-10	2.06E-07
Platynereis_dumerilii	XLOC_008606	290.3306	-4.36374	0.771133	2.17E-09	1.04E-06
Platynereis_dumerilii	XLOC_008609	320.7485	-2.68951	1.309872	2.91E-05	0.005366
Platynereis_dumerilii	XLOC_009797	21.17653	-2.77592	1.601458	3.95E-05	0.0069
Platynereis_dumerilii	XLOC_010049	237.3761	-2.75445	0.309531	2.84E-19	6.33E-16

Platynereis_dumerilii	XLOC_010101	269.7317	-1.4926	0.812882	4.48E-05	0.007758
Platynereis_dumerilii	XLOC_011325	259.3147	-3.02639	0.534737	6.12E-09	2.67E-06
Platynereis_dumerilii	XLOC_011835	69.22883	-1.02215	0.855331	0.000128	0.019493
Platynereis_dumerilii	XLOC_012051	978.3109	-3.55217	0.744645	2.50E-07	7.39E-05
Platynereis_dumerilii	XLOC_012610	61.5766	-3.44057	0.679114	9.08E-08	3.16E-05
Platynereis_dumerilii	XLOC_012776	252.6442	-2.14544	0.383394	1.34E-08	5.50E-06
Platynereis_dumerilii	XLOC_012790	219.0372	-4.30097	0.720342	3.74E-10	2.08E-07
Platynereis_dumerilii	XLOC_013607	287.4965	-3.81706	0.774697	1.20E-07	3.95E-05
Platynereis_dumerilii	XLOC_013784	566.3168	-3.24477	0.650611	1.41E-07	4.49E-05
Platynereis_dumerilii	XLOC_013785	1144.212	-2.90368	1.073313	1.23E-05	0.002446
Platynereis_dumerilii	XLOC_016316	30.44475	-3.55124	0.735546	2.07E-07	6.20E-05
Platynereis_dumerilii	XLOC_016317	88.13922	-2.41997	0.601098	2.25E-06	0.000551
Platynereis_dumerilii	XLOC_017776	252.4585	-1.25369	0.768497	5.79E-05	0.009538
Platynereis_dumerilii	XLOC_021517	81.16448	-1.98276	0.411255	3.95E-07	0.000113
Platynereis_dumerilii	XLOC_022059	222.2232	-3.20332	0.776887	1.47E-06	0.000374
Platynereis_dumerilii	XLOC_022406	1022.58	-3.13255	0.649788	2.72E-07	7.92E-05
Platynereis_dumerilii	XLOC_022616	753.6859	-3.32931	0.680511	1.91E-07	5.89E-05
Platynereis_dumerilii	XLOC_022618	312.5591	-4.13807	0.706495	8.54E-10	4.51E-07
Platynereis_dumerilii	XLOC_022619	62.9084	-3.88436	0.720229	1.41E-08	5.54E-06
Platynereis_dumerilii	XLOC_022620	218.9188	-2.4697	1.050552	2.18E-05	0.004097
Platynereis_dumerilii	XLOC_022621	661.5122	-2.72901	0.712021	2.89E-06	0.000683
Platynereis_dumerilii	XLOC_026040	3462.165	-2.81512	0.54887	9.97E-08	3.39E-05
Platynereis_dumerilii	XLOC_026126	1474.766	-4.79372	0.674768	1.29E-13	1.44E-10
Platynereis_dumerilii	XLOC_026650	802.6544	-1.25144	0.735261	5.11E-05	0.008546
Platynereis_dumerilii	XLOC_026850	97.30394	-3.20405	0.483928	1.36E-11	1.05E-08
Platynereis_dumerilii	XLOC_026851	31.50634	-3.26799	0.747536	8.60E-07	0.00023
Platynereis_dumerilii	XLOC_027093	599.2976	-5.01177	0.596554	4.04E-18	7.38E-15
Platynereis_dumerilii	XLOC_027894	2243.651	-1.28216	0.419121	7.54E-06	0.001593
Platynereis_dumerilii	XLOC_028263	1035.608	-2.38601	0.431047	1.69E-08	6.52E-06
Platynereis_dumerilii	XLOC_033183	256.4801	-3.32131	0.735177	5.91E-07	0.000163
Platynereis_dumerilii	XLOC_033184	89.92715	-5.35245	0.780983	4.04E-13	4.27E-10
Platynereis_dumerilii	XLOC_034470	2222.715	-2.87351	0.413796	1.77E-12	1.69E-09
Platynereis_dumerilii	XLOC_035469	169.8352	-3.95701	0.734598	1.37E-08	5.50E-06
Platynereis_dumerilii	XLOC_035503	535.0876	-1.20306	0.317841	2.71E-06	0.000647
Platynereis_dumerilii	XLOC_035598	28.93211	-3.67535	0.658472	6.08E-09	2.67E-06
Platynereis_dumerilii	XLOC_035616	110.5702	-3.42499	0.429401	5.10E-16	6.03E-13
Platynereis_dumerilii	XLOC_035749	48.06842	-5.38937	0.814561	1.91E-12	1.75E-09
Platynereis_dumerilii	XLOC_036017	253.9879	-4.02874	0.391717	1.87E-25	1.25E-21
Platynereis_dumerilii	XLOC_036060	1137.324	-4.14687	0.726587	2.02E-09	1.02E-06
Platynereis_dumerilii	XLOC_036107	269.1861	-2.10466	0.324318	5.73E-11	3.84E-08
Platynereis_dumerilii	XLOC_036179	1465.554	-6.08849	0.574138	8.60E-28	8.64E-24
Platynereis_dumerilii	XLOC_036282	66.88355	-3.00176	0.901426	5.44E-06	0.001227
Platynereis_dumerilii	XLOC_036426	286.3878	-1.34316	0.484132	1.12E-05	0.002301
Platynereis_dumerilii	XLOC_036464	538.0242	-4.33872	0.760139	1.69E-09	8.68E-07
Platynereis_dumerilii	XLOC_036679	9.833386	-2.31999	2.26986	7.58E-05	0.011897
Platynereis_dumerilii	XLOC_036681	54.17126	-4.7771	0.717719	2.95E-12	2.47E-09
Platynereis_dumerilii	XLOC_036747	23.01309	-2.64366	2.517457	3.67E-05	0.006462
Platynereis_dumerilii	XLOC_037581	40.30588	-2.21934	0.602111	3.94E-06	0.000898
Platynereis_dumerilii	XLOC_037670	88.09779	-3.12539	0.552892	5.99E-09	2.67E-06
Platynereis_dumerilii	XLOC_037865	30.13716	-3.24667	0.823879	1.99E-06	0.000494
Platynereis_dumerilii	XLOC_038322	82.10538	-3.89997	0.776882	7.82E-08	2.80E-05
Platynereis_dumerilii	XLOC_038323	2697.259	-3.83629	0.730222	2.93E-08	1.11E-05
Platynereis_dumerilii	XLOC_040234	88.53893	-3.49835	0.641091	1.36E-08	5.50E-06
Platynereis_dumerilii	XLOC_040543	76.90726	-2.35532	0.463068	1.43E-07	4.50E-05
Platynereis_dumerilii	XLOC_040820	21.75981	-1.84181	1.161318	6.96E-05	0.011009
Platynereis_dumerilii	XLOC_041078	36.07486	-3.10908	0.8504	3.31E-06	0.000773
Platynereis_dumerilii	XLOC_041676	214.7027	-2.68034	0.328778	1.85E-16	2.66E-13
Platynereis_dumerilii	XLOC_042653	8.456046	-1.07748	1.864578	0.000354	0.048796
Platynereis_dumerilii	XLOC_043724	405.2601	-3.82662	0.381535	2.97E-24	1.49E-20
Platynereis_dumerilii	XLOC_043773	1651.343	-2.87115	0.890512	6.56E-06	0.001401
Platynereis_dumerilii	XLOC_043853	768.2012	-2.6324	1.130928	2.14E-05	0.004058
Platynereis_dumerilii	XLOC_043938	669.9766	-2.46109	1.210098	3.26E-05	0.005795
Platynereis_dumerilii	XLOC_043939	198.3503	-1.37419	1.230292	0.00017	0.025071
Platynereis_dumerilii	XLOC_043941	105.1215	-2.58798	1.069952	1.94E-05	0.003717
Platynereis_dumerilii	XLOC_045504	566.7545	-1.96208	0.41486	5.16E-07	0.000146
Platynereis_dumerilii	XLOC_045810	451.099	-1.29279	0.814336	6.38E-05	0.010256
Platynereis_dumerilii	XLOC_045999	338.6096	-1.51406	1.241406	0.000136	0.020589
Platynereis_dumerilii	XLOC_046444	681.4837	-3.51033	0.310414	3.91E-30	7.86E-26
Platynereis_dumerilii	XLOC_046450	29.06676	-2.53375	1.235638	3.11E-05	0.005635
Platynereis_dumerilii	XLOC_050732	1081.952	-1.85993	0.320754	4.72E-09	2.20E-06
Platynereis_dumerilii	XLOC_050829	1192.18	-2.34063	0.331392	9.87E-13	9.91E-10
Platynereis_dumerilii	XLOC_051481	282.0896	-4.01505	0.506452	4.93E-16	6.03E-13
Platynereis_dumerilii	XLOC_052594	36.24835	-2.44788	0.658754	3.68E-06	0.000849
Platynereis_dumerilii	XLOC_053303	24.74815	-2.68863	0.926963	1.05E-05	0.002187
Platynereis_dumerilii	XLOC_053851	669.5119	-3.57247	0.587491	3.49E-10	2.06E-07

Platynereis_dumerilii	XLOC_055591	121.9425	-4.22502	0.472744	7.61E-20	1.91E-16
Platynereis_dumerilii	XLOC_056323	114.5158	-2.30781	0.576261	2.37E-06	0.000573
Platynereis_dumerilii	XLOC_057591	404.3977	-2.51646	2.248667	6.25E-05	0.010118
Platynereis_dumerilii	XLOC_057593	1088.594	-4.61001	0.744975	7.27E-11	4.71E-08
Platynereis_dumerilii	XLOC_057959	1944.099	-4.0451	0.745213	1.02E-08	4.37E-06
Platynereis_dumerilii	XLOC_059185	354.8508	-1.26377	0.297483	1.33E-06	0.000343
Platynereis_dumerilii	XLOC_059292	207.1675	-1.69343	0.376745	9.19E-07	0.00024
Platynereis_dumerilii	XLOC_059724	303.3497	-1.06048	0.667869	5.62E-05	0.009322
Platynereis_dumerilii	XLOC_059780	2030.403	-1.50683	1.339983	0.000165	0.024511
Platynereis_dumerilii	XLOC_060523	860.9962	-2.14897	0.626091	5.65E-06	0.001248
Platynereis_dumerilii	XLOC_060648	236.796	-2.94721	0.34396	4.75E-18	7.95E-15
Platynereis_dumerilii	XLOC_062459	583.8964	-4.01516	1.496563	5.62E-06	0.001248
Platynereis_dumerilii	XLOC_063790	2985.068	-1.3059	0.292493	8.95E-07	0.000237
Platynereis_dumerilii	XLOC_064620	47.21374	-3.48185	0.718209	2.02E-07	6.16E-05
Platynereis_dumerilii	XLOC_065187	117.4856	-1.99367	0.381935	9.12E-08	3.16E-05
Platynereis_dumerilii	XLOC_067017	363.0849	-2.98871	0.298473	5.94E-24	2.39E-20
Platynereis_dumerilii	XLOC_067823	173.0279	-5.67928	0.583088	9.62E-24	3.22E-20

Supplementary Table 27. Differentially upregulated genes in 12 hpf embryos of *P. dumerilii* after DMH1 treatment.

Species	Gene	baseMean	log2FoldChange	lfcSE	pvalue	padj
Platynereis_dumerilii	XLOC_001256	2957.601	1.034575	0.223761	5.59E-07	0.000156
Platynereis_dumerilii	XLOC_004355	816.0274	2.120119	0.940489	2.61E-05	0.004856
Platynereis_dumerilii	XLOC_007327	2458.957	1.083974	0.46837	1.77E-05	0.003427
Platynereis_dumerilii	XLOC_013038	455.27	1.319304	0.478269	1.13E-05	0.002301
Platynereis_dumerilii	XLOC_033209	1765.606	1.214977	0.303924	1.98E-06	0.000494
Platynereis_dumerilii	XLOC_059582	89.91265	1.184138	0.748427	6.12E-05	0.01
Platynereis_dumerilii	XLOC_060446	2032.468	1.041053	0.40057	1.16E-05	0.002335
Platynereis_dumerilii	XLOC_069537	474.6278	1.190675	0.496101	1.70E-05	0.003309

Supplementary Table 28. Differentially downregulated genes in 18 hpf embryos of *P. dumerilii* after DMH1 treatment.

Species	Gene	baseMean	log2FoldChange	lfcSE	pvalue	padj
Platynereis_dumerilii	XLOC_000033	46.89263	-2.97656	0.593817	1.32E-07	9.27E-05
Platynereis_dumerilii	XLOC_000809	93.59701	-4.4807	0.692313	1.40E-11	3.94E-08
Platynereis_dumerilii	XLOC_001615	50.81516	-3.07824	0.764844	1.38E-06	0.000597
Platynereis_dumerilii	XLOC_002213	356.1073	-3.47093	0.55006	9.05E-11	1.57E-07
Platynereis_dumerilii	XLOC_002291	248.3648	-1.94243	0.344467	1.12E-08	1.32E-05
Platynereis_dumerilii	XLOC_002898	116.5572	-2.86837	0.524283	1.84E-08	1.89E-05
Platynereis_dumerilii	XLOC_003658	59.83162	-1.8273	0.59912	6.25E-06	0.002346
Platynereis_dumerilii	XLOC_003971	2774.116	-1.2524	0.214964	4.65E-09	5.83E-06
Platynereis_dumerilii	XLOC_004566	1318.704	-1.88259	0.565155	4.40E-06	0.001772
Platynereis_dumerilii	XLOC_004695	21.34782	-1.19626	1.408132	0.000209	0.048459
Platynereis_dumerilii	XLOC_005270	179.6089	-3.85896	0.719568	1.66E-08	1.78E-05
Platynereis_dumerilii	XLOC_006924	249.6061	-3.61371	0.808758	4.48E-07	0.00024
Platynereis_dumerilii	XLOC_007585	130.6921	-1.05093	0.792739	6.26E-05	0.017193
Platynereis_dumerilii	XLOC_008606	249.1395	-2.8821	1.081805	8.94E-06	0.003101
Platynereis_dumerilii	XLOC_008609	393.7526	-2.25732	1.460123	4.41E-05	0.012585
Platynereis_dumerilii	XLOC_009797	14.02825	-2.79059	2.097584	3.35E-05	0.010216
Platynereis_dumerilii	XLOC_010049	160.698	-2.51182	0.879388	7.89E-06	0.002778
Platynereis_dumerilii	XLOC_010101	193.5395	-2.21127	0.404875	2.58E-08	2.42E-05
Platynereis_dumerilii	XLOC_010241	13.73933	-2.565	1.686829	3.82E-05	0.011321
Platynereis_dumerilii	XLOC_011325	83.5743	-3.69543	0.574286	3.44E-11	8.61E-08
Platynereis_dumerilii	XLOC_012610	259.0281	-1.62033	0.385891	1.19E-06	0.000528
Platynereis_dumerilii	XLOC_012790	152.7686	-3.26191	0.687234	2.70E-07	0.00016
Platynereis_dumerilii	XLOC_016317	50.41832	-2.63485	0.493115	3.87E-08	3.49E-05
Platynereis_dumerilii	XLOC_022059	198.2256	-3.41976	0.723477	2.62E-07	0.000159
Platynereis_dumerilii	XLOC_022616	762.6457	-3.88915	0.609606	4.25E-11	8.71E-08
Platynereis_dumerilii	XLOC_022617	86.81941	-4.39474	1.071242	5.00E-07	0.000253
Platynereis_dumerilii	XLOC_022618	293.3905	-4.12018	0.600689	1.41E-12	6.34E-09
Platynereis_dumerilii	XLOC_022619	61.02405	-3.27274	0.92278	2.57E-06	0.001072
Platynereis_dumerilii	XLOC_026040	3856.143	-2.69857	0.817549	4.36E-06	0.001772
Platynereis_dumerilii	XLOC_026365	588.3786	-1.51692	0.363006	1.20E-06	0.000528
Platynereis_dumerilii	XLOC_026597	85.88534	-1.95278	0.400782	2.78E-07	0.000161
Platynereis_dumerilii	XLOC_027127	68.46014	-5.15554	0.642427	8.26E-17	9.31E-13
Platynereis_dumerilii	XLOC_027130	10.56711	-1.62749	1.885238	0.000141	0.036151
Platynereis_dumerilii	XLOC_027894	2064.826	-1.76271	0.254507	3.28E-12	1.23E-08
Platynereis_dumerilii	XLOC_033183	285.5836	-3.04247	0.65173	3.71E-07	0.000204
Platynereis_dumerilii	XLOC_033184	101.3457	-3.79399	0.827103	3.00E-07	0.000169
Platynereis_dumerilii	XLOC_033456	123.4122	-1.96645	1.410866	6.08E-05	0.016921
Platynereis_dumerilii	XLOC_034470	1498.347	-3.26225	0.647584	1.03E-07	7.77E-05
Platynereis_dumerilii	XLOC_035469	156.9713	-1.57624	1.161791	6.87E-05	0.018649
Platynereis_dumerilii	XLOC_035598	51.71539	-1.3127	1.605107	0.000206	0.048319
Platynereis_dumerilii	XLOC_035616	390.9823	-2.0458	0.352236	4.17E-09	5.53E-06
Platynereis_dumerilii	XLOC_035749	112.9878	-4.3953	0.723695	1.90E-10	2.86E-07
Platynereis_dumerilii	XLOC_036017	317.9532	-3.49357	0.704501	1.20E-07	8.73E-05
Platynereis_dumerilii	XLOC_036060	979.2472	-1.54232	2.001714	0.000147	0.03676
Platynereis_dumerilii	XLOC_036179	935.505	-4.62206	0.649809	1.50E-13	1.12E-09
Platynereis_dumerilii	XLOC_036280	155.9463	-1.96023	1.402208	6.05E-05	0.016921
Platynereis_dumerilii	XLOC_036282	80.68425	-2.48816	1.157737	1.88E-05	0.006149
Platynereis_dumerilii	XLOC_036679	23.9917	-4.29273	0.801342	1.17E-08	1.32E-05
Platynereis_dumerilii	XLOC_036681	79.38868	-3.74585	0.591606	6.47E-11	1.22E-07
Platynereis_dumerilii	XLOC_036746	457.6176	-3.28824	0.77028	8.13E-07	0.000398
Platynereis_dumerilii	XLOC_037670	55.99964	-3.25161	0.479419	4.48E-12	1.44E-08
Platynereis_dumerilii	XLOC_038322	68.68904	-3.36344	0.750975	5.05E-07	0.000253
Platynereis_dumerilii	XLOC_038323	2296.386	-3.70648	0.730851	6.82E-08	5.30E-05
Platynereis_dumerilii	XLOC_039601	853.6975	-1.61199	0.542764	6.57E-06	0.002427
Platynereis_dumerilii	XLOC_040234	26.13827	-2.47495	1.614877	4.00E-05	0.011719
Platynereis_dumerilii	XLOC_041151	120.4215	-1.74606	0.83281	2.13E-05	0.006753
Platynereis_dumerilii	XLOC_042671	237.1839	-3.00333	1.01717	6.08E-06	0.002321
Platynereis_dumerilii	XLOC_043724	641.086	-3.67941	0.574542	4.25E-11	8.71E-08
Platynereis_dumerilii	XLOC_043773	1656.907	-3.27605	0.77732	9.06E-07	0.000435
Platynereis_dumerilii	XLOC_043853	758.8428	-2.9231	0.967268	5.77E-06	0.002241
Platynereis_dumerilii	XLOC_043956	307.5974	-3.74525	0.60194	1.30E-10	2.10E-07
Platynereis_dumerilii	XLOC_045504	370.1044	-1.53542	0.495922	5.46E-06	0.002159
Platynereis_dumerilii	XLOC_046444	641.0034	-3.30886	0.637359	5.40E-08	4.51E-05
Platynereis_dumerilii	XLOC_050105	20.09351	-3.0013	1.239756	1.12E-05	0.003828
Platynereis_dumerilii	XLOC_050829	933.6587	-2.72075	0.389129	1.39E-12	6.34E-09
Platynereis_dumerilii	XLOC_051481	199.6162	-1.93298	1.13399	3.81E-05	0.011321
Platynereis_dumerilii	XLOC_051599	126.4311	-3.14439	0.653035	2.34E-07	0.000146
Platynereis_dumerilii	XLOC_051900	26.91807	-1.87077	1.129386	4.11E-05	0.011884

Platynereis_dumerilii	XLOC_053851	748.4277	-2.52873	0.460457	1.95E-08	1.91E-05
Platynereis_dumerilii	XLOC_055591	47.00113	-3.20021	0.78439	1.19E-06	0.000528
Platynereis_dumerilii	XLOC_057959	1892.995	-2.9044	1.03744	7.57E-06	0.002707
Platynereis_dumerilii	XLOC_059161	51.66185	-3.46756	0.67419	6.00E-08	4.83E-05
Platynereis_dumerilii	XLOC_059724	127.1485	-1.82536	0.420405	9.53E-07	0.000448
Platynereis_dumerilii	XLOC_059780	1384.895	-2.77957	0.703274	1.71E-06	0.000728
Platynereis_dumerilii	XLOC_060523	2083.05	-2.28262	0.427541	4.53E-08	3.93E-05
Platynereis_dumerilii	XLOC_060648	305.5196	-2.73096	0.470707	3.20E-09	4.51E-06
Platynereis_dumerilii	XLOC_062459	105.7202	-3.57778	0.808151	5.02E-07	0.000253
Platynereis_dumerilii	XLOC_063049	95.43393	-2.19295	0.432584	1.41E-07	9.64E-05
Platynereis_dumerilii	XLOC_063790	2133.607	-1.74012	0.348556	1.95E-07	0.000126
Platynereis_dumerilii	XLOC_064620	33.37448	-2.69344	1.159474	1.43E-05	0.004808

Supplementary Table 29. **Differentially upregulated genes in 18 hpf embryos of *P. dumerilii* after DMH1 treatment.**

Species	Gene	baseMean	log2FoldChange	lfcSE	pvalue	padj
Platynereis_dumerilii	XLOC_008659	111.8516	4.057264	0.473783	2.45E-18	5.51E-14
Platynereis_dumerilii	XLOC_046029	138.0813	1.766349	0.593272	6.77E-06	0.00246
Platynereis_dumerilii	XLOC_067352	361.8514	1.44131	0.28662	1.72E-07	0.000114

Supplementary Table 30. Differentially downregulated genes in 24 hpf embryos of *P. dumerilii* after DMH1 treatment.

Species	Gene	baseMean	log2FoldChange	lfcSE	pvalue	padj
Platynereis_dumerilii	XLOC_001257	33.11497	-2.0035	0.646731	1.03E-05	0.001358
Platynereis_dumerilii	XLOC_001615	14.75357	-1.40007	1.329552	0.000242	0.022169
Platynereis_dumerilii	XLOC_002213	28.93266	-3.42642	0.621655	6.25E-09	1.72E-06
Platynereis_dumerilii	XLOC_002364	38.07479	-2.90786	0.632005	4.16E-07	7.61E-05
Platynereis_dumerilii	XLOC_002365	169.3301	-3.53017	0.377127	1.73E-21	6.02E-18
Platynereis_dumerilii	XLOC_002657	11.28193	-1.22895	1.454497	0.000369	0.031053
Platynereis_dumerilii	XLOC_003971	173.3462	-1.13432	0.231176	2.53E-07	4.81E-05
Platynereis_dumerilii	XLOC_004566	101.0679	-1.88872	0.321915	9.42E-10	3.17E-07
Platynereis_dumerilii	XLOC_004696	8.882307	-1.45243	1.75986	0.000249	0.022361
Platynereis_dumerilii	XLOC_006924	13.48502	-3.81627	1.518818	5.23E-06	0.000748
Platynereis_dumerilii	XLOC_006925	21.67788	-2.55898	1.024587	1.91E-05	0.002403
Platynereis_dumerilii	XLOC_006926	14.84083	-2.77054	1.406399	2.97E-05	0.003602
Platynereis_dumerilii	XLOC_006927	35.37364	-4.48304	0.711013	2.03E-11	1.06E-08
Platynereis_dumerilii	XLOC_006928	12.33923	-2.43463	1.635515	6.14E-05	0.006955
Platynereis_dumerilii	XLOC_006929	45.35365	-3.76994	0.565729	4.25E-12	2.77E-09
Platynereis_dumerilii	XLOC_007612	85.37446	-3.33253	0.413691	1.89E-16	3.28E-13
Platynereis_dumerilii	XLOC_007672	44.42908	-3.82853	0.578327	5.41E-12	3.32E-09
Platynereis_dumerilii	XLOC_008264	28.89617	-3.25432	0.607391	1.55E-08	3.95E-06
Platynereis_dumerilii	XLOC_008265	79.80084	-3.44174	0.418296	4.28E-17	8.92E-14
Platynereis_dumerilii	XLOC_008605	17.01173	-2.86552	1.098008	1.39E-05	0.001769
Platynereis_dumerilii	XLOC_008607	13.69079	-1.26712	1.369702	0.000324	0.028379
Platynereis_dumerilii	XLOC_008608	12.37562	-1.48645	1.60136	0.000252	0.022503
Platynereis_dumerilii	XLOC_008609	14.31639	-1.09609	1.324844	0.000434	0.035972
Platynereis_dumerilii	XLOC_009875	26.75772	-1.45488	1.04966	0.000135	0.013409
Platynereis_dumerilii	XLOC_011874	134.6247	-3.58838	0.416284	1.37E-18	3.58E-15
Platynereis_dumerilii	XLOC_012051	51.62855	-2.03686	1.797836	0.000113	0.011513
Platynereis_dumerilii	XLOC_012788	124.225	-3.04496	1.221539	1.38E-05	0.001769
Platynereis_dumerilii	XLOC_013606	166.6176	-3.51072	0.817904	6.66E-07	0.00012
Platynereis_dumerilii	XLOC_013607	19.54848	-3.79741	0.825383	2.28E-07	4.41E-05
Platynereis_dumerilii	XLOC_013608	36.73455	-3.72271	0.594411	6.04E-11	2.42E-08
Platynereis_dumerilii	XLOC_013784	24.60485	-3.41751	0.671311	5.07E-08	1.11E-05
Platynereis_dumerilii	XLOC_013785	56.37786	-3.62315	0.488671	2.32E-14	3.02E-11
Platynereis_dumerilii	XLOC_017637	48.29006	-3.18413	0.495315	2.99E-11	1.37E-08
Platynereis_dumerilii	XLOC_021421	56.2959	-3.32192	0.547461	2.70E-10	1.01E-07
Platynereis_dumerilii	XLOC_022059	10.6273	-3.59824	1.875847	8.89E-06	0.00119
Platynereis_dumerilii	XLOC_022406	47.67842	-3.27597	0.506598	2.23E-11	1.11E-08
Platynereis_dumerilii	XLOC_022616	32.64969	-3.66836	0.657386	3.59E-09	1.01E-06
Platynereis_dumerilii	XLOC_022618	15.20677	-2.11183	1.611768	9.84E-05	0.010261
Platynereis_dumerilii	XLOC_022621	26.4169	-3.49004	0.690852	5.72E-08	1.22E-05
Platynereis_dumerilii	XLOC_025578	16.8301	-1.05194	1.310173	0.00047	0.038307

Platynereis_dumerilii	XLOC_026040	285.6601	-3.30308	0.324932	7.30E-25	7.62E-21
Platynereis_dumerilii	XLOC_026126	76.08892	-1.97758	1.727629	0.000124	0.012517
Platynereis_dumerilii	XLOC_026741	26.62026	-1.39057	1.089901	0.000166	0.016076
Platynereis_dumerilii	XLOC_027093	45.6736	-3.88755	0.546602	1.69E-13	1.60E-10
Platynereis_dumerilii	XLOC_027894	101.4771	-1.88346	0.297011	4.85E-11	2.02E-08
Platynereis_dumerilii	XLOC_033183	20.009	-3.17177	0.730013	7.45E-07	0.000132
Platynereis_dumerilii	XLOC_033457	62.55038	-3.045	0.473172	3.01E-11	1.37E-08
Platynereis_dumerilii	XLOC_034470	62.49108	-3.11633	0.477808	1.67E-11	9.15E-09
Platynereis_dumerilii	XLOC_036017	16.18167	-2.55236	1.066221	2.18E-05	0.002704
Platynereis_dumerilii	XLOC_036060	51.99291	-3.57189	0.6108	8.51E-10	3.01E-07
Platynereis_dumerilii	XLOC_036179	22.88571	-3.21831	1.066164	6.57E-06	0.000905
Platynereis_dumerilii	XLOC_036464	43.85344	-3.84059	0.535925	1.20E-13	1.25E-10
Platynereis_dumerilii	XLOC_036746	15.31672	-3.81745	1.16852	2.63E-06	0.000398
Platynereis_dumerilii	XLOC_038321	170.8141	-1.99001	1.364579	9.46E-05	0.009973
Platynereis_dumerilii	XLOC_038323	112.7769	-2.29375	1.456884	6.61E-05	0.007335
Platynereis_dumerilii	XLOC_039734	171.3677	-2.96023	0.783043	2.63E-06	0.000398
Platynereis_dumerilii	XLOC_041487	198.5587	-3.40093	0.864859	1.51E-06	0.00025
Platynereis_dumerilii	XLOC_042670	12.59962	-1.85262	1.549699	0.00014	0.013821
Platynereis_dumerilii	XLOC_042673	117.9041	-3.106	0.894481	3.92E-06	0.000568
Platynereis_dumerilii	XLOC_042674	90.91685	-2.16514	1.656977	9.27E-05	0.009865
Platynereis_dumerilii	XLOC_043724	53.34515	-3.43336	0.465286	3.46E-14	4.01E-11
Platynereis_dumerilii	XLOC_043758	117.2603	-1.14211	1.545982	0.000431	0.035957
Platynereis_dumerilii	XLOC_043773	89.21536	-3.65629	0.387699	8.09E-22	4.22E-18
Platynereis_dumerilii	XLOC_043853	39.95938	-3.47062	0.529594	1.12E-11	6.51E-09
Platynereis_dumerilii	XLOC_043854	144.3775	-2.05065	1.515727	0.000101	0.010452
Platynereis_dumerilii	XLOC_043938	35.55319	-2.83477	0.53509	2.44E-08	5.91E-06
Platynereis_dumerilii	XLOC_043939	14.41645	-1.20362	1.346871	0.000358	0.030555
Platynereis_dumerilii	XLOC_043942	26.80396	-3.60216	0.703695	3.89E-08	8.83E-06
Platynereis_dumerilii	XLOC_043943	11.39561	-2.81711	1.92628	3.42E-05	0.004055
Platynereis_dumerilii	XLOC_043956	18.22554	-3.9859	1.156894	1.71E-06	0.000279
Platynereis_dumerilii	XLOC_045336	239.3034	-1.77479	0.249425	2.40E-13	2.09E-10
Platynereis_dumerilii	XLOC_046444	33.01963	-2.98503	0.605327	1.25E-07	2.55E-05
Platynereis_dumerilii	XLOC_050829	55.04759	-2.57761	0.480458	1.75E-08	4.36E-06
Platynereis_dumerilii	XLOC_052139	161.4146	-1.85058	0.327744	3.47E-09	1.01E-06
Platynereis_dumerilii	XLOC_053851	57.04165	-2.20008	0.378168	1.31E-09	4.27E-07
Platynereis_dumerilii	XLOC_057591	28.7595	-4.18239	0.728386	8.66E-10	3.01E-07
Platynereis_dumerilii	XLOC_057592	15.3098	-1.42826	1.587066	0.000276	0.024367
Platynereis_dumerilii	XLOC_057593	89.71421	-3.35576	0.420084	3.21E-16	4.79E-13
Platynereis_dumerilii	XLOC_057959	140.253	-1.7339	0.41019	1.44E-06	0.000242
Platynereis_dumerilii	XLOC_059780	52.30811	-2.18864	0.435917	9.11E-08	1.90E-05
Platynereis_dumerilii	XLOC_060523	189.0797	-1.38144	0.253962	1.53E-08	3.95E-06
Platynereis_dumerilii	XLOC_060648	25.04167	-2.30922	0.814135	1.37E-05	0.001769
Platynereis_dumerilii	XLOC_063183	80.56918	-1.65657	0.42658	3.06E-06	0.00045
Platynereis_dumerilii	XLOC_063790	118.7333	-1.85907	0.290792	3.47E-11	1.51E-08

Supplementary Table 31. Differentially upregulated genes in 24 hpf embryos of *P. dumerilii* after DMH1 treatment.

Species	Gene	baseMean	log2FoldChange	lfcSE	pvalue	padj
Platynereis_dumerilii	XLOC_001373	952.2582	1.001513	0.160209	2.08E-10	8.02E-08
Platynereis_dumerilii	XLOC_004295	800.4678	1.040223	0.14969	1.77E-12	1.23E-09
Platynereis_dumerilii	XLOC_004417	156.666	1.062914	0.237026	1.02E-06	0.000175
Platynereis_dumerilii	XLOC_007186	498.3826	1.157093	0.163669	6.29E-13	5.05E-10
Platynereis_dumerilii	XLOC_007327	389.0214	1.118923	0.189941	1.64E-09	5.03E-07
Platynereis_dumerilii	XLOC_008203	337.4824	1.106795	0.209795	5.11E-08	1.11E-05
Platynereis_dumerilii	XLOC_008392	758.1257	1.102213	0.158826	1.73E-12	1.23E-09
Platynereis_dumerilii	XLOC_008659	12.23856	2.445763	1.16912	3.32E-05	0.003983
Platynereis_dumerilii	XLOC_016764	72.42914	1.013513	0.615562	9.18E-05	0.009865
Platynereis_dumerilii	XLOC_026652	205.2083	1.123351	0.201127	9.66E-09	2.58E-06
Platynereis_dumerilii	XLOC_033209	395.8181	1.15143	0.280938	2.36E-06	0.000368
Platynereis_dumerilii	XLOC_051162	116.78	1.121835	0.267993	2.00E-06	0.000315
Platynereis_dumerilii	XLOC_051312	446.3935	1.07855	0.199973	2.91E-08	6.90E-06
Platynereis_dumerilii	XLOC_051771	331.5351	1.088416	0.184236	1.55E-09	4.90E-07
Platynereis_dumerilii	XLOC_052196	308.5645	1.03106	0.175407	2.01E-09	6.00E-07

Supplementary Table 32. Scoring of morphological phenotypes after blastomere ablations in *P. dumerilii.*

Species	Condition	Number of Embryos	With phenotype	Percentage
Platynereis_dumerilii	control	54	54	100.00
Platynereis_dumerilii	1D deleted	49	48	97.96
Platynereis_dumerilii	2D deleted	45	44	97.78
Platynereis_dumerilii	2d deleted	46	46	100.00
Platynereis_dumerilii	3d deleted	42	40	95.24
Platynereis_dumerilii	4d deleted	36	35	97.22

Supplementary Table 33. Differentially upregulated genes in the blastula (6 hpf) of *O. fusiformis* after SB431542 treatment.

Species	Gene_ID	base_mean	log2FoldChange	lfcSE	stat	pvalue	padj
Owenia_fusiformis	OFUSG00030.1	109.632	1.741238	0.309334	5.590081	2.27E-08	1.78E-05
Owenia_fusiformis	OFUSG00510.1	132.501	1.239398	0.335564	3.611119	0.000305	0.047807
Owenia_fusiformis	OFUSG01088.1	1103.256	1.131214	0.161787	6.987614	2.80E-12	4.07E-09
Owenia_fusiformis	OFUSG03087.1	165.2045	1.448512	0.447199	3.710769	0.000207	0.036001
Owenia_fusiformis	OFUSG06589.2	249.5202	1.550318	0.37065	4.290028	1.79E-05	0.006503
Owenia_fusiformis	OFUSG07415.1	65.02668	2.695204	0.401653	5.269028	1.37E-07	8.74E-05
Owenia_fusiformis	OFUSG07510.1	148.3875	1.391427	0.348005	3.877299	0.000106	0.022821
Owenia_fusiformis	OFUSG08951.1	780.885	1.768723	0.252728	6.987973	2.79E-12	4.07E-09
Owenia_fusiformis	OFUSG09546.2	170.7865	1.67292	0.420403	4.150633	3.32E-05	0.010052
Owenia_fusiformis	OFUSG09752.2	1575.501	1.01325	0.160227	6.324241	2.54E-10	3.05E-07
Owenia_fusiformis	OFUSG09765.1	267.5558	1.292069	0.239846	5.368569	7.94E-08	5.78E-05
Owenia_fusiformis	OFUSG10830.1	493.1321	1.999977	0.239282	8.314845	9.19E-17	3.12E-13
Owenia_fusiformis	OFUSG12614.1	64.94448	1.64429	0.416797	3.792694	0.000149	0.028527
Owenia_fusiformis	OFUSG12820.2	25.9914	1.613765	0.44058	4.487758	7.20E-06	0.00295
Owenia_fusiformis	OFUSG14162.1	220.5529	1.138312	0.291611	3.814118	0.000137	0.027227
Owenia_fusiformis	OFUSG14858.2	173.6435	1.207038	0.329444	3.713234	0.000205	0.03596
Owenia_fusiformis	OFUSG16976.1	111.8301	1.950379	0.445151	5.57245	2.51E-08	1.90E-05
Owenia_fusiformis	OFUSG17915.1	55.82751	1.293066	0.330576	3.793592	0.000148	0.028527
Owenia_fusiformis	OFUSG18221.1	14.78391	1.939704	0.444957	4.083729	4.43E-05	0.012046
Owenia_fusiformis	OFUSG18460.1	532.4168	1.57905	0.184894	8.520675	1.59E-17	6.47E-14
Owenia_fusiformis	OFUSG19764.2	347.8554	1.516457	0.354833	4.358122	1.31E-05	0.005046
Owenia_fusiformis	OFUSG21913.1	92.99508	2.069002	0.31462	6.382686	1.74E-10	2.22E-07
Owenia_fusiformis	OFUSG22812.1	34.96704	2.007979	0.417149	4.714845	2.42E-06	0.001241
Owenia_fusiformis	OFUSG23256.1	2333.319	1.030725	0.217157	4.763702	1.90E-06	0.001047
Owenia_fusiformis	OFUSG23903.1	479.8902	2.443843	0.289149	8.228011	1.90E-16	5.54E-13
Owenia_fusiformis	OFUSG23904.1	30.60349	2.07955	0.439457	3.875627	0.000106	0.022821
Owenia_fusiformis	OFUSG25096.1	125.2424	1.409382	0.33322	4.158154	3.21E-05	0.010052

Supplementary Table 34. Differentially downregulated genes in the blastula (6 hpf) of *O. fusiformis* after SB431542 treatment.

Species	Gene_ID	base_mean	log2FoldChange	lfcSE	stat	pvalue	padj
Owenia_fusiformis	OFUSG00915.1	623.7023	-1.24234	0.27817	-4.49636	6.91E-06	0.002936
Owenia_fusiformis	OFUSG02906.1	428.2503	-2.64794	0.366891	-7.43956	1.01E-13	1.72E-10
Owenia_fusiformis	OFUSG03058.1	338.2893	-2.5082	0.253748	-9.84585	7.14E-23	1.46E-18
Owenia_fusiformis	OFUSG03505.1	500.3474	-1.15622	0.310732	-3.71713	0.000202	0.035718
Owenia_fusiformis	OFUSG04352.1	17.33356	-1.60087	0.440833	-4.15448	3.26E-05	0.010052
Owenia_fusiformis	OFUSG04473.2	158.67	-2.24453	0.399473	-5.61045	2.02E-08	1.68E-05
Owenia_fusiformis	OFUSG05637.4	1242.222	-1.08751	0.237433	-4.60237	4.18E-06	0.001851
Owenia_fusiformis	OFUSG07842.1	95.73778	-2.3702	0.360736	-6.52975	6.59E-11	8.95E-08
Owenia_fusiformis	OFUSG07844.1	2169.228	-1.5929	0.185431	-8.60742	7.47E-18	3.81E-14
Owenia_fusiformis	OFUSG08791.1	2062.744	-1.06629	0.245317	-4.34425	1.40E-05	0.005276
Owenia_fusiformis	OFUSG09123.2	101.359	-1.23339	0.326983	-3.82297	0.000132	0.02715
Owenia_fusiformis	OFUSG09430.1	794.2881	-1.15013	0.306275	-3.81953	0.000134	0.027227
Owenia_fusiformis	OFUSG09614.3	335.35	-1.44584	0.291868	-5.01478	5.31E-07	0.000309
Owenia_fusiformis	OFUSG09661.1	92.72611	-1.44105	0.377638	-3.75875	0.000171	0.03108
Owenia_fusiformis	OFUSG11213.1	1070.384	-1.06949	0.246322	-4.41244	1.02E-05	0.004007

Owenia_fusiformis	OFUSG11532.1	2166.936	-1.47483	0.281786	-5.28363	1.27E-07	8.33E-05
Owenia_fusiformis	OFUSG11955.1	281.7888	-2.75362	0.285378	-9.58316	9.41E-22	6.40E-18
Owenia_fusiformis	OFUSG12379.1	2476.144	-1.27029	0.239703	-5.32917	9.87E-08	6.94E-05
Owenia_fusiformis	OFUSG13054.2	218.5792	-1.54258	0.44724	-4.63172	3.63E-06	0.00168
Owenia_fusiformis	OFUSG13678.1	318.7728	-1.5518	0.428974	-4.18311	2.88E-05	0.009454
Owenia_fusiformis	OFUSG14965.1	325.5819	-1.37183	0.445366	-4.43201	9.34E-06	0.003732
Owenia_fusiformis	OFUSG16118.1	1157.88	-2.52148	0.322298	-7.97601	1.51E-15	3.42E-12
Owenia_fusiformis	OFUSG16199.1	1588.542	-2.6695	0.276809	-9.74331	1.97E-22	2.01E-18
Owenia_fusiformis	OFUSG16343.3	159.3012	-2.51987	0.427843	-5.94711	2.73E-09	2.65E-06
Owenia_fusiformis	OFUSG16834.1	296.9221	-1.01702	0.223151	-4.55305	5.29E-06	0.002293
Owenia_fusiformis	OFUSG17539.2	80.67797	-1.48724	0.441952	-5.03235	4.84E-07	0.00029
Owenia_fusiformis	OFUSG17825.1	339.5321	-1.89209	0.353551	-5.31993	1.04E-07	7.05E-05
Owenia_fusiformis	OFUSG18408.1	115.4914	-1.3751	0.447122	-4.0543	5.03E-05	0.013142
Owenia_fusiformis	OFUSG18413.1	915.3889	-1.23854	0.448701	-4.1844	2.86E-05	0.009454
Owenia_fusiformis	OFUSG18416.1	1218.199	-1.10292	0.447689	-3.67161	0.000241	0.039623
Owenia_fusiformis	OFUSG20121.5	19.15386	-1.1952	0.354346	-3.69288	0.000222	0.037049
Owenia_fusiformis	OFUSG20608.2	1183.714	-1.28486	0.322363	-4.00997	6.07E-05	0.015426
Owenia_fusiformis	OFUSG21000.3	4372.762	-1.36268	0.30092	-4.60414	4.14E-06	0.001851
Owenia_fusiformis	OFUSG21187.4	191.007	-1.43642	0.417979	-3.81249	0.000138	0.027227
Owenia_fusiformis	OFUSG21715.2	180.3863	-1.30454	0.340098	-3.99356	6.51E-05	0.015986
Owenia_fusiformis	OFUSG23113.1	927.9813	-1.66096	0.275642	-6.06694	1.30E-09	1.44E-06
Owenia_fusiformis	OFUSG23557.1	174.7043	-1.16272	0.248369	-4.67956	2.87E-06	0.001368
Owenia_fusiformis	OFUSG24245.1	20.736	-1.07015	0.386517	-3.97539	7.03E-05	0.016851
Owenia_fusiformis	OFUSG25014.1	2635.899	-1.8507	0.336384	-5.60677	2.06E-08	1.68E-05
Owenia_fusiformis	OFUSG26269.1	410.854	-1.47141	0.425711	-3.66054	0.000252	0.040719
Owenia_fusiformis	OFUSG26536.2	199.3074	-1.11833	0.283269	-3.98617	6.71E-05	0.016295

Supplementary Table 35. Scoring of the expression of candidate genes at the blastula stage of *O. fusiformis* after SB431542 treatment.

Species	Treatment	Total_n	*otx*	*hb*	*prospero*
Owenia_fusiformis	0.4% DMSO	53	19	21	13
Owenia_fusiformis	40uM SB431542	69	36	19	14

Supplementary Table 36. Differentially upregulated genes in the blastula (6 hpf) of *O. fusiformis* after rActivin A treatment.

Species	Gene_ID	basal_mean	log2FoldChange	lfcSE	stat	pvalue	padj
Owenia_fusiformis	OFUSG00005.1	20.21667	1.214761	0.448085	2.970857	0.00297	0.017873
Owenia_fusiformis	OFUSG00069.2	15.86811	1.324431	0.397143	4.020914	5.80E-05	0.000765
Owenia_fusiformis	OFUSG00096.1	140.1696	2.59601	0.414133	6.001694	1.95E-09	1.10E-07
Owenia_fusiformis	OFUSG00096.3	162.751	1.340242	0.24612	5.432095	5.57E-08	2.06E-06
Owenia_fusiformis	OFUSG00102.1	43.38152	2.05315	0.448676	3.999718	6.34E-05	0.000823
Owenia_fusiformis	OFUSG00152.1	7.963748	1.571794	0.439276	3.036131	0.002396	0.015167
Owenia_fusiformis	OFUSG00170.1	118.3037	2.191518	0.379865	5.603048	2.11E-08	8.85E-07
Owenia_fusiformis	OFUSG00179.1	263.8856	1.813105	0.201271	8.992039	2.43E-19	1.03E-16
Owenia_fusiformis	OFUSG00183.1	12.7139	1.300835	0.447121	2.874907	0.004041	0.02276
Owenia_fusiformis	OFUSG00201.1	444.1775	1.058663	0.183962	5.759191	8.45E-09	3.97E-07
Owenia_fusiformis	OFUSG00256.1	644.7066	1.011638	0.200051	5.057216	4.25E-07	1.17E-05
Owenia_fusiformis	OFUSG00258.1	644.0241	1.366051	0.164254	8.315684	9.12E-17	2.77E-14
Owenia_fusiformis	OFUSG00261.1	103.5256	1.040576	0.326089	3.196838	0.001389	0.009864
Owenia_fusiformis	OFUSG00286.1	358.3874	1.534584	0.214022	7.145654	8.96E-13	1.19E-10
Owenia_fusiformis	OFUSG00306.1	18.68832	1.425812	0.448417	3.317329	0.000909	0.007038
Owenia_fusiformis	OFUSG00354.1	11.44655	1.344045	0.448562	2.786345	0.005331	0.028141
Owenia_fusiformis	OFUSG00406.1	317.3727	1.010761	0.194309	5.20252	1.97E-07	6.07E-06
Owenia_fusiformis	OFUSG00448.1	38.75483	1.076797	0.397097	2.748989	0.005978	0.030709
Owenia_fusiformis	OFUSG00456.1	2991.973	1.869462	0.18099	10.337	4.79E-25	4.07E-22
Owenia_fusiformis	OFUSG00499.1	38.21481	1.199009	0.426686	2.85496	0.004304	0.023874
Owenia_fusiformis	OFUSG00519.1	103.0413	1.096885	0.428162	2.588834	0.00963	0.044189
Owenia_fusiformis	OFUSG00538.1	928.8254	1.192277	0.231206	5.162217	2.44E-07	7.36E-06
Owenia_fusiformis	OFUSG00573.1	419.8073	1.217503	0.191949	6.341473	2.28E-10	1.59E-08
Owenia_fusiformis	OFUSG00618.1	19.74481	1.289321	0.448507	2.857339	0.004272	0.023753
Owenia_fusiformis	OFUSG00637.1	180.8984	1.197879	0.379642	3.19401	0.001403	0.009944
Owenia_fusiformis	OFUSG00655.1	64.95441	1.320232	0.355466	3.666585	0.000246	0.002496
Owenia_fusiformis	OFUSG00657.1	3348.809	1.211689	0.238305	5.131991	2.87E-07	8.37E-06
Owenia_fusiformis	OFUSG00680.1	32.63501	1.330015	0.436537	3.265142	0.001094	0.008138
Owenia_fusiformis	OFUSG00687.1	17.48167	1.35654	0.448469	3.069239	0.002146	0.013909

Owenia_fusiformis	OFUSG00711.1	113.9819	1.425454	0.300332	4.729653	2.25E-06	4.95E-05
Owenia_fusiformis	OFUSG00810.1	18.93052	1.80361	0.436347	3.933909	8.36E-05	0.001036
Owenia_fusiformis	OFUSG00831.1	179.0946	1.454552	0.234322	6.199581	5.66E-10	3.68E-08
Owenia_fusiformis	OFUSG00871.2	96.37835	1.006877	0.385635	2.560239	0.01046	0.047102
Owenia_fusiformis	OFUSG00891.1	279.211	1.519923	0.277247	5.449519	5.05E-08	1.90E-06
Owenia_fusiformis	OFUSG01092.1	19.84814	1.187494	0.4487	2.715809	0.006611	0.033228
Owenia_fusiformis	OFUSG01138.1	54.17418	1.474469	0.362366	4.065588	4.79E-05	0.000658
Owenia_fusiformis	OFUSG01191.1	76.14384	1.561504	0.436099	3.298653	0.000972	0.007398
Owenia_fusiformis	OFUSG01305.1	19.4048	2.185003	0.448262	4.651451	3.30E-06	6.84E-05
Owenia_fusiformis	OFUSG01346.1	103.3039	1.375037	0.370896	3.67276	0.00024	0.002455
Owenia_fusiformis	OFUSG01380.1	338.339	1.076949	0.298121	3.565436	0.000363	0.003404
Owenia_fusiformis	OFUSG01382.1	59.17019	2.108756	0.421887	4.694784	2.67E-06	5.71E-05
Owenia_fusiformis	OFUSG01395.1	123.9164	1.663235	0.342139	4.789765	1.67E-06	3.82E-05
Owenia_fusiformis	OFUSG01403.1	211.4828	1.716792	0.249812	6.859291	6.92E-12	7.28E-10
Owenia_fusiformis	OFUSG01418.5	248.7085	1.188407	0.249934	4.748228	2.05E-06	4.59E-05
Owenia_fusiformis	OFUSG01430.1	5695.45	1.031793	0.146005	7.070588	1.54E-12	1.91E-10
Owenia_fusiformis	OFUSG01473.1	1016.293	1.228288	0.266511	4.55863	5.15E-06	0.000101
Owenia_fusiformis	OFUSG01558.1	21.42598	1.985911	0.4486	3.836887	0.000125	0.001434
Owenia_fusiformis	OFUSG01633.1	345.0055	1.166175	0.269151	4.343671	1.40E-05	0.000236
Owenia_fusiformis	OFUSG01658.1	94.77971	1.587016	0.327308	4.832824	1.35E-06	3.17E-05
Owenia_fusiformis	OFUSG01702.1	1864.279	1.033007	0.201345	5.132213	2.86E-07	8.37E-06
Owenia_fusiformis	OFUSG01730.1	153.7423	3.288563	0.383753	6.883132	5.86E-12	6.37E-10
Owenia_fusiformis	OFUSG01770.1	203.4625	2.172582	0.355699	6.102661	1.04E-09	6.27E-08
Owenia_fusiformis	OFUSG01844.1	44.8567	1.986733	0.394844	4.991654	5.99E-07	1.56E-05
Owenia_fusiformis	OFUSG01855.1	6.267344	1.524413	0.373706	3.527753	0.000419	0.003802
Owenia_fusiformis	OFUSG01859.1	1339.081	1.003753	0.151602	6.620831	3.57E-11	3.16E-09
Owenia_fusiformis	OFUSG01890.2	415.8336	2.433482	0.222109	10.92737	8.53E-28	9.11E-25
Owenia_fusiformis	OFUSG01902.1	585.6606	1.096551	0.17451	6.284642	3.29E-10	2.22E-08
Owenia_fusiformis	OFUSG01934.1	33.92298	1.341865	0.398649	3.296034	0.000981	0.007458
Owenia_fusiformis	OFUSG01942.1	82.74917	1.22198	0.372659	3.221135	0.001277	0.009216
Owenia_fusiformis	OFUSG01969.1	64.90314	1.295536	0.352284	3.659762	0.000252	0.002545
Owenia_fusiformis	OFUSG01972.1	65.37832	1.267869	0.359474	3.473899	0.000513	0.004469
Owenia_fusiformis	OFUSG02061.1	85.70305	3.679986	0.388221	8.880694	6.64E-19	2.73E-16
Owenia_fusiformis	OFUSG02069.1	633.8525	1.060795	0.240039	4.41707	1.00E-05	0.000178
Owenia_fusiformis	OFUSG02077.1	81.44028	1.006094	0.313757	3.212626	0.001315	0.009435
Owenia_fusiformis	OFUSG02107.1	9.259218	1.463323	0.405125	3.341603	0.000833	0.006564
Owenia_fusiformis	OFUSG02115.1	66.58813	1.31593	0.314644	4.169512	3.05E-05	0.000454
Owenia_fusiformis	OFUSG02154.1	35.83059	1.995459	0.42787	4.302543	1.69E-05	0.000275
Owenia_fusiformis	OFUSG02178.1	18.18833	1.192726	0.448704	2.868287	0.004127	0.023146
Owenia_fusiformis	OFUSG02281.1	46.60569	1.955397	0.389172	5.01375	5.34E-07	1.41E-05
Owenia_fusiformis	OFUSG02338.1	9.211143	1.927414	0.435622	3.382455	0.000718	0.005834
Owenia_fusiformis	OFUSG02369.1	272.8588	1.050801	0.310303	3.389349	0.000701	0.005717
Owenia_fusiformis	OFUSG02383.2	320.3311	1.436957	0.377815	3.922289	8.77E-05	0.001075
Owenia_fusiformis	OFUSG02399.1	24.8293	1.282235	0.411999	3.067127	0.002161	0.013981
Owenia_fusiformis	OFUSG02449.1	21990.57	1.005395	0.289902	3.43645	0.000589	0.004969
Owenia_fusiformis	OFUSG02561.1	23.10627	1.444143	0.428369	3.445709	0.00057	0.004837
Owenia_fusiformis	OFUSG02571.1	35.05658	1.38968	0.430335	3.15517	0.001604	0.01114
Owenia_fusiformis	OFUSG02588.1	368.9874	1.365532	0.30236	4.499491	6.81E-06	0.000129
Owenia_fusiformis	OFUSG02589.1	24.24955	1.654709	0.434685	3.775955	0.000159	0.001755
Owenia_fusiformis	OFUSG02604.1	38.01486	2.383495	0.417193	5.543023	2.97E-08	1.19E-06
Owenia_fusiformis	OFUSG02637.3	34.75171	1.478211	0.448673	3.029376	0.002451	0.015407
Owenia_fusiformis	OFUSG02645.1	65.33715	1.445785	0.401288	2.825458	0.004721	0.025678
Owenia_fusiformis	OFUSG02654.1	1344.147	1.181387	0.205895	5.733705	9.83E-09	4.52E-07
Owenia_fusiformis	OFUSG02713.1	131.2707	1.168028	0.246573	4.732574	2.22E-06	4.90E-05
Owenia_fusiformis	OFUSG02718.1	15.4217	1.650392	0.448328	3.448319	0.000564	0.004797
Owenia_fusiformis	OFUSG02722.1	199.4601	1.525049	0.270125	5.648386	1.62E-08	7.02E-07
Owenia_fusiformis	OFUSG02765.1	106.5768	1.566367	0.415273	3.778002	0.000158	0.001743
Owenia_fusiformis	OFUSG02787.1	80.76601	1.285704	0.42264	2.827325	0.004694	0.025563
Owenia_fusiformis	OFUSG02794.1	53.19108	4.90697	0.434496	6.338338	2.32E-10	1.61E-08
Owenia_fusiformis	OFUSG02795.1	23.81849	1.22128	0.43707	2.850109	0.00437	0.024176
Owenia_fusiformis	OFUSG02797.1	104.0878	1.710913	0.411235	4.091518	4.29E-05	0.000601
Owenia_fusiformis	OFUSG02801.2	9.154654	1.805954	0.39325	3.107457	0.001887	0.012594
Owenia_fusiformis	OFUSG02808.1	528.5695	1.226512	0.216706	5.655964	1.55E-08	6.74E-07
Owenia_fusiformis	OFUSG02822.1	120.6088	1.197502	0.274687	4.353205	1.34E-05	0.000228
Owenia_fusiformis	OFUSG02888.1	86.07456	2.043087	0.448595	4.166601	3.09E-05	0.000459
Owenia_fusiformis	OFUSG02902.1	44.26874	1.293897	0.401826	3.215825	0.001301	0.009339
Owenia_fusiformis	OFUSG02937.1	154.5611	1.318782	0.315848	4.18204	2.89E-05	0.000433
Owenia_fusiformis	OFUSG02944.1	12.89521	1.246838	0.446741	2.540507	0.011069	0.049206
Owenia_fusiformis	OFUSG02981.1	8.502079	1.540538	0.420265	3.207071	0.001341	0.009572
Owenia_fusiformis	OFUSG02990.1	41.19757	1.502798	0.440457	3.227032	0.001251	0.009076
Owenia_fusiformis	OFUSG03052.2	185.5165	1.397539	0.428568	3.5007	0.000464	0.004118
Owenia_fusiformis	OFUSG03083.1	35.72577	1.362865	0.448007	3.332418	0.000861	0.006755
Owenia_fusiformis	OFUSG03113.1	235.4126	1.192701	0.32037	3.720817	0.000199	0.002096
Owenia_fusiformis	OFUSG03152.1	349.1109	1.10793	0.191579	5.777344	7.59E-09	3.59E-07
Owenia_fusiformis	OFUSG03164.1	727.7393	1.125598	0.228804	4.928187	8.30E-07	2.08E-05
Owenia_fusiformis	OFUSG03231.1	119.4472	1.442755	0.303229	4.75148	2.02E-06	4.53E-05
Owenia_fusiformis	OFUSG03232.1	119.7959	1.679567	0.282451	5.92062	3.21E-09	1.68E-07
Owenia_fusiformis	OFUSG03243.1	60.12819	3.427492	0.430999	7.427647	1.11E-13	1.78E-11
Owenia_fusiformis	OFUSG03361.1	132.1168	1.232072	0.352584	3.477923	0.000505	0.004416
Owenia_fusiformis	OFUSG03370.1	297.3641	1.099194	0.204958	5.357559	8.44E-08	2.93E-06

Owenia_fusiformis	OFUSG03389.1	82.05653	1.012487	0.357683	2.786092	0.005335	0.028141
Owenia_fusiformis	OFUSG03390.1	26.45285	1.680309	0.43612	3.968542	7.23E-05	0.00092
Owenia_fusiformis	OFUSG03391.2	36.18178	1.122478	0.439053	2.675311	0.007466	0.036496
Owenia_fusiformis	OFUSG03452.2	26.8675	1.271706	0.445183	3.180839	0.001468	0.010333
Owenia_fusiformis	OFUSG03453.1	10.8129	1.639706	0.446249	3.333314	0.000858	0.006744
Owenia_fusiformis	OFUSG03521.1	43.43825	2.625781	0.409545	6.067889	1.30E-09	7.53E-08
Owenia_fusiformis	OFUSG03549.1	126.37	1.612711	0.340161	4.775389	1.79E-06	4.08E-05
Owenia_fusiformis	OFUSG03583.1	453.9752	1.103415	0.232456	4.743458	2.10E-06	4.68E-05
Owenia_fusiformis	OFUSG03607.1	20.98901	1.265786	0.447519	3.02142	0.002516	0.015715
Owenia_fusiformis	OFUSG03619.1	75.13354	1.40611	0.397712	3.543383	0.000395	0.003635
Owenia_fusiformis	OFUSG03620.1	23.46554	1.235471	0.435314	2.909381	0.003621	0.020887
Owenia_fusiformis	OFUSG03678.1	16057.66	1.662287	0.207193	8.027919	9.91E-16	2.57E-13
Owenia_fusiformis	OFUSG03723.1	27.80742	2.254149	0.437226	4.698293	2.62E-06	5.64E-05
Owenia_fusiformis	OFUSG03746.1	8.724917	1.068634	0.428313	2.674367	0.007487	0.036561
Owenia_fusiformis	OFUSG03780.1	68.54519	1.06235	0.399841	2.707051	0.006788	0.033897
Owenia_fusiformis	OFUSG03815.1	95.10248	2.081964	0.386003	5.099364	3.41E-07	9.68E-06
Owenia_fusiformis	OFUSG03816.1	219.6689	1.331801	0.272099	4.89296	9.93E-07	2.43E-05
Owenia_fusiformis	OFUSG03824.2	23.12852	2.242486	0.446078	4.519162	6.21E-06	0.000119
Owenia_fusiformis	OFUSG03833.2	8.810938	1.571598	0.435602	4.039716	5.35E-05	0.000718
Owenia_fusiformis	OFUSG03935.1	3249.699	1.154295	0.155647	7.420641	1.17E-13	1.86E-11
Owenia_fusiformis	OFUSG03942.1	49.50023	1.264446	0.38077	4.779025	1.76E-06	4.02E-05
Owenia_fusiformis	OFUSG03951.1	195.0196	1.640211	0.309604	5.306441	1.12E-07	3.74E-06
Owenia_fusiformis	OFUSG03956.1	6.969158	1.08338	0.42377	2.736702	0.006206	0.031649
Owenia_fusiformis	OFUSG03957.2	29.20241	1.115454	0.426058	2.607108	0.009131	0.042401
Owenia_fusiformis	OFUSG04007.1	174.1943	1.228439	0.260714	4.708081	2.50E-06	5.42E-05
Owenia_fusiformis	OFUSG04032.1	15.6352	1.56155	0.447788	3.728059	0.000193	0.002047
Owenia_fusiformis	OFUSG04067.1	9.359205	1.236047	0.448633	2.657528	0.007872	0.037914
Owenia_fusiformis	OFUSG04110.1	28.94903	1.951218	0.433326	4.379553	1.19E-05	0.000205
Owenia_fusiformis	OFUSG04218.1	310.003	2.264231	0.339646	6.481077	9.11E-11	7.12E-09
Owenia_fusiformis	OFUSG04230.1	26.80715	1.609938	0.429304	3.752456	0.000175	0.001893
Owenia_fusiformis	OFUSG04239.1	35.95216	2.445636	0.414321	5.507819	3.63E-08	1.42E-06
Owenia_fusiformis	OFUSG04258.1	74.97304	1.545091	0.307164	5.013149	5.35E-07	1.41E-05
Owenia_fusiformis	OFUSG04298.1	56.61746	1.119919	0.407832	2.656775	0.007889	0.037976
Owenia_fusiformis	OFUSG04299.1	107.6312	2.547152	0.310978	8.008567	1.16E-15	2.98E-13
Owenia_fusiformis	OFUSG04352.2	17.06231	1.038899	0.384496	3.030517	0.002441	0.015369
Owenia_fusiformis	OFUSG04412.1	56.01117	1.517236	0.353836	4.246584	2.17E-05	0.00034
Owenia_fusiformis	OFUSG04484.1	400.8913	1.129591	0.172126	6.561495	5.33E-11	4.40E-09
Owenia_fusiformis	OFUSG04521.2	592.8026	1.052901	0.178016	5.915913	3.30E-09	1.72E-07
Owenia_fusiformis	OFUSG04539.2	171.3131	1.037986	0.37175	2.7404	0.006136	0.031353
Owenia_fusiformis	OFUSG04580.2	63.99355	1.608403	0.440356	5.361583	8.25E-08	2.89E-06
Owenia_fusiformis	OFUSG04601.1	154.2017	2.058383	0.351167	5.717867	1.08E-08	4.91E-07
Owenia_fusiformis	OFUSG04654.1	2747.373	1.263749	0.14557	8.681014	3.92E-18	1.44E-15
Owenia_fusiformis	OFUSG04663.1	282.5531	1.460717	0.212898	6.859885	6.89E-12	7.28E-10
Owenia_fusiformis	OFUSG04704.1	21.44171	1.465477	0.443852	3.191567	0.001415	0.010011
Owenia_fusiformis	OFUSG04709.1	413.5981	1.293531	0.378017	3.440109	0.000581	0.004923
Owenia_fusiformis	OFUSG04721.1	10.38516	1.10776	0.444604	2.72092	0.00651	0.032887
Owenia_fusiformis	OFUSG04728.1	31.88136	1.47092	0.421909	3.499479	0.000466	0.004134
Owenia_fusiformis	OFUSG04730.2	93.57293	1.262812	0.424089	2.954358	0.003133	0.018667
Owenia_fusiformis	OFUSG04732.1	28.62225	2.632923	0.444269	5.054807	4.31E-07	1.19E-05
Owenia_fusiformis	OFUSG04796.1	127.8082	1.128498	0.252345	4.465956	7.97E-06	0.000147
Owenia_fusiformis	OFUSG04807.1	39.48028	1.845844	0.391497	4.419881	9.88E-06	0.000176
Owenia_fusiformis	OFUSG04831.1	13.47039	1.074329	0.440939	2.625594	0.00865	0.040761
Owenia_fusiformis	OFUSG04836.1	195.073	1.539203	0.309033	5.012189	5.38E-07	1.42E-05
Owenia_fusiformis	OFUSG04876.1	4.376295	1.215803	0.325361	3.054717	0.002253	0.014436
Owenia_fusiformis	OFUSG04881.1	34.57943	1.295515	0.436648	2.769717	0.00561	0.029275
Owenia_fusiformis	OFUSG04933.1	69.89439	1.753309	0.410419	4.15443	3.26E-05	0.000479
Owenia_fusiformis	OFUSG04945.1	1168.496	1.023457	0.17942	5.706408	1.15E-08	5.22E-07
Owenia_fusiformis	OFUSG04961.1	12942.22	1.164036	0.256482	4.550226	5.36E-06	0.000105
Owenia_fusiformis	OFUSG05020.1	102.5608	2.03617	0.416157	4.529838	5.90E-06	0.000114
Owenia_fusiformis	OFUSG05027.1	16.29599	1.903214	0.447614	3.567207	0.000361	0.003386
Owenia_fusiformis	OFUSG05052.1	68.72776	1.264589	0.344307	3.644577	0.000268	0.002666
Owenia_fusiformis	OFUSG05112.1	8.909516	1.782275	0.444463	3.555297	0.000378	0.003511
Owenia_fusiformis	OFUSG05166.1	182.0319	1.210186	0.240626	5.031197	4.87E-07	1.31E-05
Owenia_fusiformis	OFUSG05176.1	156.6401	1.22703	0.438079	2.713649	0.006655	0.033405
Owenia_fusiformis	OFUSG05222.2	122.6947	2.518038	0.402993	6.335833	2.36E-10	1.63E-08
Owenia_fusiformis	OFUSG05235.1	79.19527	1.479253	0.375567	3.882147	0.000104	0.001232
Owenia_fusiformis	OFUSG05276.1	26.41431	1.16821	0.444356	2.583399	0.009783	0.044758
Owenia_fusiformis	OFUSG05314.1	40.13879	1.623344	0.441968	3.641938	0.000271	0.002689
Owenia_fusiformis	OFUSG05347.1	7836.307	1.010284	0.288677	3.465072	0.00053	0.004579
Owenia_fusiformis	OFUSG05348.2	44.13731	2.21305	0.421597	4.905506	9.32E-07	2.30E-05
Owenia_fusiformis	OFUSG05376.1	134.5872	2.266972	0.34826	6.510134	7.51E-11	6.00E-09
Owenia_fusiformis	OFUSG05417.1	351.4437	1.38063	0.284187	4.834798	1.33E-06	3.15E-05
Owenia_fusiformis	OFUSG05483.1	810.6557	1.006339	0.234407	4.293919	1.76E-05	0.000284
Owenia_fusiformis	OFUSG05527.1	110.393	1.219808	0.292743	4.169599	3.05E-05	0.000454
Owenia_fusiformis	OFUSG05528.1	670.1867	1.022811	0.150543	6.794406	1.09E-11	1.10E-09
Owenia_fusiformis	OFUSG05548.1	949.3819	1.495853	0.281302	5.402438	6.57E-08	2.38E-06
Owenia_fusiformis	OFUSG05552.2	467.2643	1.091069	0.265288	4.118025	3.82E-05	0.000546
Owenia_fusiformis	OFUSG05567.1	261.3089	1.122089	0.244518	4.590907	4.41E-06	8.86E-05
Owenia_fusiformis	OFUSG05568.1	56.08204	2.190044	0.411637	5.125572	2.97E-07	8.60E-06
Owenia_fusiformis	OFUSG05618.1	468.7079	1.029039	0.234106	4.394653	1.11E-05	0.000194

Owenia_fusiformis	OFUSG05672.1	670.2907	2.613816	0.206684	12.61159	1.82E-36	3.45E-33
Owenia_fusiformis	OFUSG05679.1	16.55847	1.306956	0.443338	2.845251	0.004438	0.024476
Owenia_fusiformis	OFUSG05682.2	46.50143	1.007565	0.400192	2.54692	0.010868	0.048495
Owenia_fusiformis	OFUSG05709.1	2101.984	1.737586	0.113187	15.34841	3.63E-53	2.23E-49
Owenia_fusiformis	OFUSG05715.1	149.7149	1.037432	0.272059	3.813023	0.000137	0.00155
Owenia_fusiformis	OFUSG05742.1	1636.294	1.045221	0.152228	6.861751	6.80E-12	7.22E-10
Owenia_fusiformis	OFUSG05756.1	27.04744	1.966698	0.441991	3.819681	0.000134	0.001516
Owenia_fusiformis	OFUSG05769.1	417.7362	1.11911	0.226642	4.93636	7.96E-07	2.01E-05
Owenia_fusiformis	OFUSG05797.2	133.2111	1.282906	0.305963	4.190005	2.79E-05	0.000421
Owenia_fusiformis	OFUSG05920.1	2243.315	1.000714	0.102032	9.809478	1.02E-22	6.82E-20
Owenia_fusiformis	OFUSG05940.1	1826.175	1.128153	0.16717	6.748493	1.49E-11	1.44E-09
Owenia_fusiformis	OFUSG05949.1	36.17505	3.71577	0.441251	6.943198	3.83E-12	4.33E-10
Owenia_fusiformis	OFUSG05964.1	22.95041	1.387265	0.43908	2.866828	0.004146	0.023222
Owenia_fusiformis	OFUSG05967.1	987.7667	1.294899	0.241069	5.356901	8.47E-08	2.94E-06
Owenia_fusiformis	OFUSG05968.1	43.82638	2.294207	0.404537	5.394307	6.88E-08	2.47E-06
Owenia_fusiformis	OFUSG05991.1	1103.244	1.023122	0.17426	5.871751	4.31E-09	2.19E-07
Owenia_fusiformis	OFUSG06068.2	478.9345	1.317655	0.199677	6.59796	4.17E-11	3.58E-09
Owenia_fusiformis	OFUSG06071.1	13.71222	1.815453	0.444042	3.783261	0.000155	0.001712
Owenia_fusiformis	OFUSG06116.1	148.9018	1.174507	0.247629	4.735597	2.18E-06	4.84E-05
Owenia_fusiformis	OFUSG06117.1	13.63551	1.661511	0.395321	2.950316	0.003174	0.018865
Owenia_fusiformis	OFUSG06134.1	12.10738	1.129054	0.438068	2.70531	0.006824	0.034026
Owenia_fusiformis	OFUSG06144.1	5785.696	1.315504	0.257449	5.138661	2.77E-07	8.15E-06
Owenia_fusiformis	OFUSG06159.1	41.81803	1.150214	0.39048	2.952719	0.00315	0.018746
Owenia_fusiformis	OFUSG06182.1	286.8513	1.338185	0.311038	4.250277	2.14E-05	0.000336
Owenia_fusiformis	OFUSG06183.1	272.6922	1.898062	0.353687	5.225661	1.74E-07	5.50E-06
Owenia_fusiformis	OFUSG06191.1	103.333	1.057119	0.312845	3.366422	0.000762	0.006111
Owenia_fusiformis	OFUSG06195.4	139.8711	1.371929	0.442279	2.916849	0.003536	0.020484
Owenia_fusiformis	OFUSG06249.2	862.6087	1.100044	0.186227	5.905147	3.52E-09	1.83E-07
Owenia_fusiformis	OFUSG06250.1	134.376	1.02414	0.307549	3.311813	0.000927	0.00714
Owenia_fusiformis	OFUSG06277.2	28.71337	1.7804	0.44304	3.391933	0.000694	0.005674
Owenia_fusiformis	OFUSG06282.1	128.9165	1.392594	0.319057	4.34469	1.39E-05	0.000235
Owenia_fusiformis	OFUSG06291.1	135.4247	1.017933	0.302741	3.366386	0.000762	0.006111
Owenia_fusiformis	OFUSG06296.1	3670.939	1.037726	0.208537	4.981541	6.31E-07	1.64E-05
Owenia_fusiformis	OFUSG06301.1	69.82351	1.212745	0.367737	3.278544	0.001043	0.007825
Owenia_fusiformis	OFUSG06341.1	36.56672	1.526686	0.428897	3.284905	0.00102	0.007704
Owenia_fusiformis	OFUSG06457.1	196.4892	1.174747	0.321201	3.670479	0.000242	0.00247
Owenia_fusiformis	OFUSG06475.1	1319.442	1.00689	0.207686	4.841166	1.29E-06	3.06E-05
Owenia_fusiformis	OFUSG06515.1	3639.679	1.068827	0.158911	6.72646	1.74E-11	1.65E-09
Owenia_fusiformis	OFUSG06519.1	14.28183	1.344878	0.44861	2.783693	0.005374	0.028281
Owenia_fusiformis	OFUSG06573.1	125.0112	1.287668	0.290269	4.429507	9.44E-06	0.00017
Owenia_fusiformis	OFUSG06671.1	338.9637	1.003464	0.184358	5.439475	5.34E-08	1.99E-06
Owenia_fusiformis	OFUSG06676.1	256.911	1.244729	0.434442	2.937626	0.003307	0.019461
Owenia_fusiformis	OFUSG06698.2	1620.239	1.237922	0.214321	5.780632	7.44E-09	3.55E-07
Owenia_fusiformis	OFUSG06724.1	37.70838	1.333087	0.385961	3.363171	0.000771	0.006177
Owenia_fusiformis	OFUSG06728.1	10.80966	1.425449	0.446212	2.935602	0.003329	0.019566
Owenia_fusiformis	OFUSG06734.1	369.2691	1.40272	0.179473	7.813298	5.57E-15	1.24E-12
Owenia_fusiformis	OFUSG06835.1	5.194587	1.564416	0.392487	3.218104	0.00129	0.009291
Owenia_fusiformis	OFUSG06845.1	16.44769	1.278207	0.447058	2.77156	0.005579	0.029143
Owenia_fusiformis	OFUSG06848.1	43.7875	1.520283	0.417601	3.550099	0.000385	0.003569
Owenia_fusiformis	OFUSG06860.1	321.3374	1.086712	0.223722	4.858117	1.19E-06	2.85E-05
Owenia_fusiformis	OFUSG06863.1	186.7939	1.179803	0.269038	4.377288	1.20E-05	0.000207
Owenia_fusiformis	OFUSG06905.1	32.58365	1.180063	0.406979	2.880019	0.003977	0.022481
Owenia_fusiformis	OFUSG06981.2	642.8557	1.544093	0.351347	4.445636	8.76E-06	0.000159
Owenia_fusiformis	OFUSG06988.1	22.68046	1.425787	0.448533	3.397903	0.000679	0.005582
Owenia_fusiformis	OFUSG06991.1	56.4156	1.245617	0.379822	3.299321	0.000969	0.007387
Owenia_fusiformis	OFUSG07009.1	59.23274	1.234526	0.413667	2.963225	0.003044	0.018242
Owenia_fusiformis	OFUSG07009.4	107.6578	1.363241	0.36403	3.720865	0.000199	0.002096
Owenia_fusiformis	OFUSG07053.1	151.3076	1.885656	0.447067	4.424201	9.68E-06	0.000173
Owenia_fusiformis	OFUSG07055.1	46.92315	1.998108	0.394657	4.995818	5.86E-07	1.53E-05
Owenia_fusiformis	OFUSG07101.1	57.83868	1.148426	0.368263	3.08972	0.002003	0.013199
Owenia_fusiformis	OFUSG07105.1	386.0985	1.44713	0.308382	4.706093	2.53E-06	5.47E-05
Owenia_fusiformis	OFUSG07110.2	1218.163	1.190402	0.184694	6.446146	1.15E-10	8.67E-09
Owenia_fusiformis	OFUSG07116.1	1311.099	1.096083	0.246225	4.453491	8.45E-06	0.000154
Owenia_fusiformis	OFUSG07154.1	478.9037	1.136122	0.300296	3.796156	0.000147	0.001639
Owenia_fusiformis	OFUSG07211.1	111.7455	1.055149	0.31543	3.305928	0.000947	0.007265
Owenia_fusiformis	OFUSG07236.1	11.66107	2.032939	0.431576	4.014936	5.95E-05	0.000781
Owenia_fusiformis	OFUSG07268.1	135.2296	1.847131	0.339507	5.381105	7.40E-08	2.63E-06
Owenia_fusiformis	OFUSG07317.1	1992.119	2.651331	0.445465	4.968901	6.73E-07	1.73E-05
Owenia_fusiformis	OFUSG07330.1	431.697	1.203216	0.213584	5.627536	1.83E-08	7.80E-07
Owenia_fusiformis	OFUSG07337.1	81.70067	4.370759	0.434873	8.477504	2.30E-17	7.76E-15
Owenia_fusiformis	OFUSG07342.1	82.33586	1.104489	0.337824	3.281572	0.001032	0.007779
Owenia_fusiformis	OFUSG07355.1	33.07258	1.116493	0.411469	2.663602	0.007731	0.037426
Owenia_fusiformis	OFUSG07375.1	14.95071	1.341455	0.445797	3.337179	0.000846	0.006657
Owenia_fusiformis	OFUSG07403.1	41.37979	1.531312	0.422727	3.602587	0.000315	0.003048
Owenia_fusiformis	OFUSG07404.1	41.37979	1.531312	0.422727	3.602587	0.000315	0.003048
Owenia_fusiformis	OFUSG07408.1	127.0551	1.207836	0.31043	3.880729	0.000104	0.001238
Owenia_fusiformis	OFUSG07413.1	25.53276	1.383357	0.433461	3.101121	0.001928	0.012814
Owenia_fusiformis	OFUSG07417.1	194.1243	1.401504	0.38396	3.701463	0.000214	0.002235
Owenia_fusiformis	OFUSG07443.2	231.5251	1.184563	0.229648	5.170218	2.34E-07	7.08E-06
Owenia_fusiformis	OFUSG07514.1	24.04864	1.503088	0.447848	2.942128	0.00326	0.019218

Owenia_fusiformis	OFUSG07529.1	38.90542	1.301229	0.429408	2.877712	0.004006	0.02262
Owenia_fusiformis	OFUSG07583.1	86.19244	1.076276	0.416385	2.616375	0.008887	0.041496
Owenia_fusiformis	OFUSG07598.2	1714.599	1.012217	0.252949	3.994077	6.49E-05	0.00084
Owenia_fusiformis	OFUSG07641.1	253.4823	1.433333	0.257039	5.573942	2.49E-08	1.02E-06
Owenia_fusiformis	OFUSG07676.1	113.7382	1.077797	0.30663	3.515534	0.000439	0.003934
Owenia_fusiformis	OFUSG07682.2	21.10513	1.341237	0.444976	2.835054	0.004582	0.025069
Owenia_fusiformis	OFUSG07698.2	82.76987	1.360734	0.359621	3.701494	0.000214	0.002235
Owenia_fusiformis	OFUSG07767.1	1554.876	1.028984	0.185747	5.552857	2.81E-08	1.14E-06
Owenia_fusiformis	OFUSG07842.1	95.73778	1.381734	0.347672	3.940282	8.14E-05	0.001013
Owenia_fusiformis	OFUSG07851.1	1396.268	1.105355	0.129473	8.536483	1.38E-17	4.73E-15
Owenia_fusiformis	OFUSG07869.1	16.33841	1.613624	0.447672	3.426052	0.000612	0.005126
Owenia_fusiformis	OFUSG07880.1	4.900042	1.182587	0.419377	2.657874	0.007864	0.037905
Owenia_fusiformis	OFUSG07914.2	34.49737	1.224425	0.431066	2.575668	0.010005	0.0455
Owenia_fusiformis	OFUSG07928.2	232.8973	1.30898	0.370898	3.470983	0.000519	0.004509
Owenia_fusiformis	OFUSG07959.1	56.11896	1.22393	0.36767	3.328276	0.000874	0.006829
Owenia_fusiformis	OFUSG08054.2	29.58529	1.319383	0.440092	2.871161	0.00409	0.022974
Owenia_fusiformis	OFUSG08138.1	27.73501	1.240362	0.428606	2.945355	0.003226	0.01906
Owenia_fusiformis	OFUSG08170.1	26.05627	1.307337	0.445838	3.330022	0.000868	0.006798
Owenia_fusiformis	OFUSG08204.1	36.1389	1.966825	0.433406	4.482606	7.37E-06	0.000138
Owenia_fusiformis	OFUSG08259.3	101.9087	1.142354	0.442144	2.580263	0.009873	0.045091
Owenia_fusiformis	OFUSG08266.1	128.2584	1.212017	0.340185	3.69557	0.000219	0.00228
Owenia_fusiformis	OFUSG08271.1	64.37793	3.562904	0.37096	9.049964	1.43E-19	6.44E-17
Owenia_fusiformis	OFUSG08290.1	14.6378	1.260999	0.446127	2.702861	0.006875	0.034209
Owenia_fusiformis	OFUSG08305.1	76.26105	1.357764	0.393938	3.558316	0.000373	0.003481
Owenia_fusiformis	OFUSG08315.1	783.9971	1.440845	0.245164	5.887029	3.93E-09	2.01E-07
Owenia_fusiformis	OFUSG08316.1	88.253	1.029587	0.405098	2.5756	0.010007	0.045501
Owenia_fusiformis	OFUSG08317.2	416.0101	1.38191	0.315423	4.412972	1.02E-05	0.000181
Owenia_fusiformis	OFUSG08318.1	594.1161	1.886937	0.266402	7.084217	1.40E-12	1.76E-10
Owenia_fusiformis	OFUSG08320.1	72.77575	2.205252	0.376304	5.800763	6.60E-09	3.18E-07
Owenia_fusiformis	OFUSG08321.1	54.28487	2.211171	0.422085	5.104688	3.31E-07	9.46E-06
Owenia_fusiformis	OFUSG08323.1	42.02899	1.043811	0.398432	2.570842	0.010145	0.046029
Owenia_fusiformis	OFUSG08337.2	44.95725	1.660689	0.390192	4.235669	2.28E-05	0.000355
Owenia_fusiformis	OFUSG08379.1	38.14937	1.335525	0.422131	3.223736	0.001265	0.009157
Owenia_fusiformis	OFUSG08406.1	90.96685	1.606205	0.375415	4.155021	3.25E-05	0.000478
Owenia_fusiformis	OFUSG08418.1	3419.69	1.14547	0.161712	7.082108	1.42E-12	1.77E-10
Owenia_fusiformis	OFUSG08432.1	30.55392	1.542327	0.415456	3.599298	0.000319	0.003073
Owenia_fusiformis	OFUSG08505.1	140.7361	2.060095	0.270045	7.59127	3.17E-14	5.78E-12
Owenia_fusiformis	OFUSG08613.1	16.58402	1.549929	0.438369	3.329015	0.000872	0.006817
Owenia_fusiformis	OFUSG08634.1	10.116	1.512498	0.4264	2.620369	0.008783	0.041155
Owenia_fusiformis	OFUSG08655.2	545.0004	1.072639	0.340395	3.219702	0.001283	0.009246
Owenia_fusiformis	OFUSG08669.1	858.5692	1.308855	0.194738	6.721318	1.80E-11	1.70E-09
Owenia_fusiformis	OFUSG08681.1	82.05606	1.20926	0.448504	2.924276	0.003453	0.02011
Owenia_fusiformis	OFUSG08709.1	129.3374	1.254234	0.399123	3.029387	0.002451	0.015407
Owenia_fusiformis	OFUSG08728.1	33.11788	1.401881	0.448515	2.786741	0.005324	0.028116
Owenia_fusiformis	OFUSG08747.1	61.82348	1.429281	0.356903	3.889451	0.0001	0.001202
Owenia_fusiformis	OFUSG08786.1	6.433532	1.23755	0.395603	2.957043	0.003106	0.01853
Owenia_fusiformis	OFUSG08808.1	36.84226	2.93321	0.433963	6.097163	1.08E-09	6.44E-08
Owenia_fusiformis	OFUSG08810.1	353.3415	1.227635	0.269328	4.532329	5.83E-06	0.000113
Owenia_fusiformis	OFUSG08823.1	132.3728	1.384172	0.314738	4.430057	9.42E-06	0.000169
Owenia_fusiformis	OFUSG08854.1	33.68274	1.099639	0.411616	2.621769	0.008747	0.041057
Owenia_fusiformis	OFUSG08857.1	28.95687	1.184703	0.423209	2.752726	0.00591	0.030462
Owenia_fusiformis	OFUSG08883.1	45.63247	1.150091	0.426585	2.670715	0.007569	0.03686
Owenia_fusiformis	OFUSG08941.1	192.0842	1.710952	0.326378	5.21376	1.85E-07	5.77E-06
Owenia_fusiformis	OFUSG08948.1	82.60123	2.654823	0.434862	5.54644	2.92E-08	1.17E-06
Owenia_fusiformis	OFUSG08961.1	32.38418	1.590555	0.407548	3.965234	7.33E-05	0.00093
Owenia_fusiformis	OFUSG08983.1	7.185066	1.054262	0.408559	2.604477	0.009201	0.042667
Owenia_fusiformis	OFUSG08990.1	249.2945	1.042045	0.178832	5.827644	5.62E-09	2.75E-07
Owenia_fusiformis	OFUSG08994.1	862.3079	1.113258	0.122594	9.08459	1.04E-19	4.84E-17
Owenia_fusiformis	OFUSG09036.1	13.12618	2.173229	0.442836	4.110807	3.94E-05	0.000559
Owenia_fusiformis	OFUSG09049.2	102.6094	1.276724	0.330442	3.843946	0.000121	0.001399
Owenia_fusiformis	OFUSG09051.1	17.69609	1.601795	0.44327	3.454051	0.000552	0.004726
Owenia_fusiformis	OFUSG09074.1	586.2863	2.284756	0.439075	3.617694	0.000297	0.002908
Owenia_fusiformis	OFUSG09089.1	8.603746	1.434215	0.445247	3.021714	0.002513	0.015714
Owenia_fusiformis	OFUSG09101.1	29.26737	1.162521	0.413273	2.793693	0.005211	0.027652
Owenia_fusiformis	OFUSG09206.1	427.2807	1.22606	0.276167	4.482738	7.37E-06	0.000138
Owenia_fusiformis	OFUSG09221.1	161.4411	1.048795	0.267905	3.915132	9.04E-05	0.0011
Owenia_fusiformis	OFUSG09227.1	66.47772	1.129021	0.397485	2.718383	0.00656	0.033038
Owenia_fusiformis	OFUSG09238.1	39.03001	1.329749	0.422833	2.831769	0.004629	0.025272
Owenia_fusiformis	OFUSG09313.1	857.7803	1.913433	0.127915	14.94863	1.59E-50	6.53E-47
Owenia_fusiformis	OFUSG09316.1	181.2624	1.004286	0.238841	4.205094	2.61E-05	0.000396
Owenia_fusiformis	OFUSG09394.1	6.495495	1.301364	0.361669	2.75519	0.005866	0.03031
Owenia_fusiformis	OFUSG09462.1	137.901	1.096654	0.321787	3.407546	0.000655	0.005428
Owenia_fusiformis	OFUSG09531.1	692.69	1.280383	0.209698	6.10466	1.03E-09	6.20E-08
Owenia_fusiformis	OFUSG09562.1	14.44531	1.290668	0.448611	2.584157	0.009762	0.044676
Owenia_fusiformis	OFUSG09595.3	424.1721	1.320221	0.417651	3.16823	0.001534	0.010715
Owenia_fusiformis	OFUSG09601.2	1950.097	1.012959	0.154232	6.567628	5.11E-11	4.25E-09
Owenia_fusiformis	OFUSG09608.1	85.01374	1.409244	0.292377	4.812148	1.49E-06	3.47E-05
Owenia_fusiformis	OFUSG09610.1	12.54854	1.518881	0.410122	3.575582	0.000349	0.00331
Owenia_fusiformis	OFUSG09627.1	2218.553	1.007714	0.141978	7.097387	1.27E-12	1.62E-10
Owenia_fusiformis	OFUSG09644.1	42.93825	1.449515	0.440218	2.982657	0.002858	0.017321

Owenia_fusiformis	OFUSG09656.1	13.42459	1.070215	0.44837	2.550415	0.010759	0.048116
Owenia_fusiformis	OFUSG09695.1	874.8574	1.060773	0.174901	6.064673	1.32E-09	7.64E-08
Owenia_fusiformis	OFUSG09710.1	9.699212	1.479958	0.448271	3.055964	0.002243	0.014395
Owenia_fusiformis	OFUSG09745.1	387.823	1.020832	0.166251	6.142291	8.13E-10	5.02E-08
Owenia_fusiformis	OFUSG09773.1	40.42734	1.532184	0.421866	3.689056	0.000225	0.002325
Owenia_fusiformis	OFUSG09778.1	268.2326	1.704644	0.269519	6.33003	2.45E-10	1.68E-08
Owenia_fusiformis	OFUSG09808.1	4.571566	1.058741	0.373763	3.14342	0.00167	0.0115
Owenia_fusiformis	OFUSG09884.1	2308.534	1.023988	0.08814	11.61958	3.28E-31	5.04E-28
Owenia_fusiformis	OFUSG09885.1	825.6693	1.312884	0.196798	6.676688	2.44E-11	2.22E-09
Owenia_fusiformis	OFUSG09895.2	14.80023	1.428215	0.431872	3.265309	0.001093	0.008135
Owenia_fusiformis	OFUSG09937.1	211.8633	1.16473	0.208791	5.580694	2.40E-08	9.85E-07
Owenia_fusiformis	OFUSG09938.1	832.4644	1.057879	0.246675	4.295305	1.74E-05	0.000283
Owenia_fusiformis	OFUSG09952.1	128.5051	1.072988	0.37363	2.907984	0.003638	0.020946
Owenia_fusiformis	OFUSG09960.2	35.40125	1.228332	0.44552	4.447065	8.71E-06	0.000158
Owenia_fusiformis	OFUSG09971.1	292.7796	1.073009	0.234336	4.577044	4.72E-06	9.39E-05
Owenia_fusiformis	OFUSG10053.1	688.0627	1.473876	0.18562	7.939253	2.03E-15	4.96E-13
Owenia_fusiformis	OFUSG10060.1	19.095	1.539454	0.446375	3.02	0.002528	0.015768
Owenia_fusiformis	OFUSG10063.4	142.5186	1.095638	0.412336	2.606232	0.009154	0.042487
Owenia_fusiformis	OFUSG10114.1	113.5641	2.073165	0.275118	7.481815	7.33E-14	1.24E-11
Owenia_fusiformis	OFUSG10120.1	530.8687	1.029745	0.210052	4.905717	9.31E-07	2.30E-05
Owenia_fusiformis	OFUSG10201.1	6.41493	1.288236	0.387092	2.749874	0.005962	0.030664
Owenia_fusiformis	OFUSG10257.1	409.4142	1.008713	0.309081	3.253803	0.001139	0.008411
Owenia_fusiformis	OFUSG10259.2	23.816	1.15496	0.448304	2.619628	0.008803	0.041221
Owenia_fusiformis	OFUSG10351.1	15.58255	2.483432	0.420003	4.228784	2.35E-05	0.000364
Owenia_fusiformis	OFUSG10378.1	419.9322	1.17925	0.254153	4.651128	3.30E-06	6.85E-05
Owenia_fusiformis	OFUSG10419.1	377.1022	1.925138	0.243898	7.890378	3.01E-15	7.13E-13
Owenia_fusiformis	OFUSG10472.1	161.0254	1.134768	0.244813	4.632312	3.62E-06	7.43E-05
Owenia_fusiformis	OFUSG10492.1	16.01544	1.540378	0.448412	3.076969	0.002091	0.013653
Owenia_fusiformis	OFUSG10525.1	15.83436	1.983561	0.445586	4.074703	4.61E-05	0.000637
Owenia_fusiformis	OFUSG10554.1	991.1195	1.141383	0.218508	5.223877	1.75E-07	5.53E-06
Owenia_fusiformis	OFUSG10579.1	24.04941	1.750765	0.431142	4.387801	1.15E-05	0.000199
Owenia_fusiformis	OFUSG10647.2	23.33802	1.055101	0.441498	2.796351	0.005168	0.02752
Owenia_fusiformis	OFUSG10656.1	13.89783	1.154401	0.441156	2.684962	0.007254	0.035692
Owenia_fusiformis	OFUSG10657.1	158.4703	1.467536	0.230881	6.353759	2.10E-10	1.49E-08
Owenia_fusiformis	OFUSG10697.1	12.31504	1.391035	0.436707	3.112231	0.001857	0.012449
Owenia_fusiformis	OFUSG10708.1	8.657799	1.369182	0.430505	2.700547	0.006923	0.034399
Owenia_fusiformis	OFUSG10722.1	682.9005	1.067399	0.258929	4.114277	3.88E-05	0.000553
Owenia_fusiformis	OFUSG10748.1	95.60598	3.461547	0.392348	8.313771	9.27E-17	2.78E-14
Owenia_fusiformis	OFUSG10774.1	209.4211	1.01519	0.286806	3.511326	0.000446	0.003981
Owenia_fusiformis	OFUSG10777.1	20.4884	1.306871	0.445401	2.761318	0.005757	0.029841
Owenia_fusiformis	OFUSG10786.1	294.8048	1.906339	0.21416	8.89007	6.11E-19	2.55E-16
Owenia_fusiformis	OFUSG10799.1	92.90007	1.724389	0.351414	4.918864	8.70E-07	2.17E-05
Owenia_fusiformis	OFUSG10811.3	39.15221	1.645604	0.4487	3.814371	0.000137	0.001543
Owenia_fusiformis	OFUSG10832.1	873.4137	1.042237	0.309382	3.384262	0.000714	0.005808
Owenia_fusiformis	OFUSG10892.1	12.20823	1.34493	0.447664	2.691277	0.007118	0.035171
Owenia_fusiformis	OFUSG10899.1	566.9053	1.486395	0.223821	6.631718	3.32E-11	2.96E-09
Owenia_fusiformis	OFUSG10924.1	55.36991	1.375583	0.349524	3.894572	9.84E-05	0.00118
Owenia_fusiformis	OFUSG10936.1	87.97465	1.494222	0.339354	4.409899	1.03E-05	0.000183
Owenia_fusiformis	OFUSG10957.1	9.640778	1.304609	0.441297	3.079896	0.002071	0.013534
Owenia_fusiformis	OFUSG10967.1	706.2826	1.129928	0.217459	5.188444	2.12E-07	6.49E-06
Owenia_fusiformis	OFUSG10989.1	202.5129	1.052272	0.317838	3.303678	0.000954	0.007301
Owenia_fusiformis	OFUSG10997.1	19.44182	1.083327	0.448257	2.716659	0.006594	0.033163
Owenia_fusiformis	OFUSG11018.1	1058.578	1.823239	0.448504	3.878719	0.000105	0.001248
Owenia_fusiformis	OFUSG11027.1	48.91211	1.124985	0.398023	2.785137	0.005351	0.028178
Owenia_fusiformis	OFUSG11106.1	103.516	1.130603	0.340983	3.332091	0.000862	0.00676
Owenia_fusiformis	OFUSG11136.1	1192.54	1.048803	0.210181	4.985656	6.18E-07	1.61E-05
Owenia_fusiformis	OFUSG11168.1	25.60657	1.101716	0.44321	2.561343	0.010427	0.046995
Owenia_fusiformis	OFUSG11238.4	228.7846	1.163599	0.37365	3.067682	0.002157	0.013962
Owenia_fusiformis	OFUSG11261.1	75.73548	1.248874	0.353518	3.540327	0.0004	0.003661
Owenia_fusiformis	OFUSG11263.1	117.1271	1.216514	0.341618	3.544131	0.000394	0.00363
Owenia_fusiformis	OFUSG11278.1	13.94472	1.385849	0.435199	2.903254	0.003693	0.02119
Owenia_fusiformis	OFUSG11329.1	7.177332	1.381735	0.428641	2.848098	0.004398	0.024302
Owenia_fusiformis	OFUSG11332.1	41.98463	1.165063	0.381832	2.996517	0.002731	0.016734
Owenia_fusiformis	OFUSG11367.3	73.4514	2.656365	0.448162	6.100998	1.05E-09	6.32E-08
Owenia_fusiformis	OFUSG11376.2	13.54194	1.056743	0.325239	3.472663	0.000515	0.004486
Owenia_fusiformis	OFUSG11394.1	7.703784	1.263131	0.437885	2.925842	0.003435	0.020028
Owenia_fusiformis	OFUSG11435.1	161.6771	1.078743	0.368019	2.901039	0.003719	0.021306
Owenia_fusiformis	OFUSG11460.1	47.8769	1.857975	0.410224	4.334156	1.46E-05	0.000244
Owenia_fusiformis	OFUSG11482.2	82.93098	1.532249	0.385134	3.876142	0.000106	0.001259
Owenia_fusiformis	OFUSG11503.1	47.89593	1.042289	0.389314	2.679849	0.007366	0.036145
Owenia_fusiformis	OFUSG11515.1	25.52079	1.901901	0.430385	4.15157	3.30E-05	0.000485
Owenia_fusiformis	OFUSG11589.1	1393.597	1.150105	0.222201	5.178211	2.24E-07	6.81E-06
Owenia_fusiformis	OFUSG11602.1	13.88475	1.45009	0.447626	2.977328	0.002908	0.017538
Owenia_fusiformis	OFUSG11618.1	1044.377	1.000027	0.213792	4.682884	2.83E-06	5.99E-05
Owenia_fusiformis	OFUSG11625.1	145.7878	1.148081	0.335255	3.427789	0.000609	0.005099
Owenia_fusiformis	OFUSG11679.1	12.02528	1.180585	0.446706	2.840794	0.0045	0.024743
Owenia_fusiformis	OFUSG11711.1	28.01178	1.36216	0.448367	3.061767	0.0022	0.014167
Owenia_fusiformis	OFUSG11720.2	1734.693	1.541203	0.166557	9.256569	2.11E-20	1.18E-17
Owenia_fusiformis	OFUSG11738.1	1006.264	1.048908	0.208282	5.03664	4.74E-07	1.28E-05
Owenia_fusiformis	OFUSG11739.1	750.2782	1.456009	0.215071	6.754109	1.44E-11	1.39E-09

Owenia_fusiformis	OFUSG11778.1	304.6518	1.118634	0.227143	4.922921	8.53E-07	2.13E-05
Owenia_fusiformis	OFUSG11791.2	2613.174	1.042053	0.156492	6.661678	2.71E-11	2.45E-09
Owenia_fusiformis	OFUSG11797.1	126.2912	1.306738	0.251899	5.163505	2.42E-07	7.31E-06
Owenia_fusiformis	OFUSG11843.1	805.0334	1.226206	0.244445	5.023811	5.07E-07	1.35E-05
Owenia_fusiformis	OFUSG11941.1	604.5244	1.382655	0.247095	5.597348	2.18E-08	9.04E-07
Owenia_fusiformis	OFUSG11953.1	11.57466	1.758581	0.445094	3.405823	0.00066	0.005455
Owenia_fusiformis	OFUSG11962.1	23.56216	1.326499	0.431306	2.816249	0.004859	0.026206
Owenia_fusiformis	OFUSG11988.1	33.16458	2.468931	0.401829	5.929045	3.05E-09	1.60E-07
Owenia_fusiformis	OFUSG11997.1	25.42738	1.18127	0.421465	2.718391	0.00656	0.033038
Owenia_fusiformis	OFUSG12030.1	2464.69	1.120423	0.196664	5.698777	1.21E-08	5.44E-07
Owenia_fusiformis	OFUSG12059.2	7.213877	1.254378	0.344861	2.722244	0.006484	0.032816
Owenia_fusiformis	OFUSG12070.1	17.71856	1.5671	0.438683	2.761904	0.005747	0.029818
Owenia_fusiformis	OFUSG12114.3	329.0071	1.182176	0.199192	5.930935	3.01E-09	1.59E-07
Owenia_fusiformis	OFUSG12191.1	18.30871	2.218058	0.442136	3.8705	0.000109	0.001281
Owenia_fusiformis	OFUSG12212.1	11.59856	1.28385	0.448398	2.820599	0.004793	0.025927
Owenia_fusiformis	OFUSG12241.1	10.12559	1.285344	0.426075	2.966153	0.003016	0.0181
Owenia_fusiformis	OFUSG12319.1	206.7624	1.366829	0.200171	6.819526	9.13E-12	9.37E-10
Owenia_fusiformis	OFUSG12325.1	231.2064	1.346185	0.314208	4.29197	1.77E-05	0.000286
Owenia_fusiformis	OFUSG12353.1	953.3486	1.125666	0.20777	5.417771	6.03E-08	2.20E-06
Owenia_fusiformis	OFUSG12364.1	26.74136	1.302656	0.419606	3.105505	0.0019	0.012657
Owenia_fusiformis	OFUSG12384.1	47.14188	1.403251	0.445673	2.979336	0.002889	0.017445
Owenia_fusiformis	OFUSG12401.1	26.38806	1.354678	0.43484	3.243718	0.00118	0.008642
Owenia_fusiformis	OFUSG12402.1	115.2311	1.14843	0.356431	3.226734	0.001252	0.009077
Owenia_fusiformis	OFUSG12428.1	9.015489	1.028673	0.432134	2.665601	0.007685	0.037249
Owenia_fusiformis	OFUSG12476.1	39.33608	1.817666	0.427052	4.054712	5.02E-05	0.000683
Owenia_fusiformis	OFUSG12491.1	378.3688	1.215493	0.271935	4.482684	7.37E-06	0.000138
Owenia_fusiformis	OFUSG12493.1	6.580205	1.487986	0.432058	2.70957	0.006737	0.033721
Owenia_fusiformis	OFUSG12496.1	75.32505	1.345696	0.392123	3.430358	0.000603	0.005066
Owenia_fusiformis	OFUSG12502.1	11.28384	1.12477	0.438277	2.838087	0.004538	0.02491
Owenia_fusiformis	OFUSG12509.1	20.41488	1.579503	0.43591	3.38241	0.000719	0.005834
Owenia_fusiformis	OFUSG12534.2	317.5262	1.622831	0.30325	5.330212	9.81E-08	3.35E-06
Owenia_fusiformis	OFUSG12548.1	411.2718	1.269512	0.247187	5.137432	2.79E-07	8.19E-06
Owenia_fusiformis	OFUSG12638.1	8998.748	1.710844	0.263319	6.481896	9.06E-11	7.10E-09
Owenia_fusiformis	OFUSG12653.1	13.48467	1.441423	0.418943	2.627134	0.008611	0.040616
Owenia_fusiformis	OFUSG12720.1	147.1571	1.51867	0.410895	3.752499	0.000175	0.001893
Owenia_fusiformis	OFUSG12729.1	43.02644	1.250536	0.427792	2.884564	0.00392	0.022216
Owenia_fusiformis	OFUSG12730.1	149.7913	1.184699	0.275595	4.291174	1.78E-05	0.000287
Owenia_fusiformis	OFUSG12736.1	1651.679	1.75745	0.10748	16.34954	4.38E-60	3.60E-56
Owenia_fusiformis	OFUSG12784.2	59.48266	1.169569	0.434098	2.563605	0.010359	0.046793
Owenia_fusiformis	OFUSG12785.1	14.39601	2.473438	0.447339	4.844386	1.27E-06	3.03E-05
Owenia_fusiformis	OFUSG12792.1	27.01833	1.415362	0.413994	3.435169	0.000592	0.004989
Owenia_fusiformis	OFUSG12794.1	20.32473	1.142275	0.43298	2.601991	0.009268	0.042905
Owenia_fusiformis	OFUSG12796.1	25.36508	1.863778	0.445745	3.797194	0.000146	0.001634
Owenia_fusiformis	OFUSG12799.1	2577.702	1.170298	0.181293	6.452129	1.10E-10	8.38E-09
Owenia_fusiformis	OFUSG12846.4	35.26256	1.915514	0.446849	4.483707	7.34E-06	0.000137
Owenia_fusiformis	OFUSG12880.1	24.44612	1.914011	0.44777	3.797399	0.000146	0.001633
Owenia_fusiformis	OFUSG12882.1	14.98465	2.903765	0.443318	4.753175	2.00E-06	4.50E-05
Owenia_fusiformis	OFUSG12884.1	12.73575	3.035095	0.445662	4.522804	6.10E-06	0.000117
Owenia_fusiformis	OFUSG12952.1	20.66563	1.679912	0.446432	3.561652	0.000369	0.003443
Owenia_fusiformis	OFUSG12954.1	28.42886	1.386136	0.432791	3.014774	0.002572	0.015973
Owenia_fusiformis	OFUSG12971.1	38.32352	1.844931	0.445869	4.579884	4.65E-06	9.28E-05
Owenia_fusiformis	OFUSG13062.1	86.09481	2.234397	0.424124	5.054353	4.32E-07	1.19E-05
Owenia_fusiformis	OFUSG13086.1	94.47867	1.35539	0.333929	4.02708	5.65E-05	0.000749
Owenia_fusiformis	OFUSG13098.1	967.3664	1.016236	0.148013	6.863929	6.70E-12	7.17E-10
Owenia_fusiformis	OFUSG13175.1	625.7921	1.331456	0.198783	6.702717	2.05E-11	1.89E-09
Owenia_fusiformis	OFUSG13234.1	42.46521	1.160306	0.366961	3.136496	0.00171	0.011706
Owenia_fusiformis	OFUSG13236.1	333.0133	1.406943	0.194123	7.243777	4.36E-13	6.36E-11
Owenia_fusiformis	OFUSG13247.1	212.1738	1.055589	0.33747	3.17194	0.001514	0.010594
Owenia_fusiformis	OFUSG13248.1	581.3214	1.084858	0.17655	6.14796	7.85E-10	4.89E-08
Owenia_fusiformis	OFUSG13254.1	276.5595	1.007505	0.249959	4.029061	5.60E-05	0.000745
Owenia_fusiformis	OFUSG13327.1	191.6325	1.876004	0.303617	6.116365	9.57E-10	5.82E-08
Owenia_fusiformis	OFUSG13342.1	261.6695	1.66858	0.421174	3.7795	0.000157	0.001735
Owenia_fusiformis	OFUSG13342.2	459.66	1.136475	0.340382	3.249227	0.001157	0.008509
Owenia_fusiformis	OFUSG13414.1	389.4978	1.048194	0.21508	4.865902	1.14E-06	2.75E-05
Owenia_fusiformis	OFUSG13417.1	161.085	1.925696	0.276133	6.956294	3.49E-12	4.00E-10
Owenia_fusiformis	OFUSG13448.1	300.1993	1.015298	0.257027	3.953218	7.71E-05	0.000969
Owenia_fusiformis	OFUSG13461.2	394.7142	1.405909	0.239353	5.856842	4.72E-09	2.36E-07
Owenia_fusiformis	OFUSG13465.1	265.2934	1.016077	0.363139	2.785079	0.005351	0.028178
Owenia_fusiformis	OFUSG13482.2	2184.139	1.141352	0.184778	6.181402	6.35E-10	4.07E-08
Owenia_fusiformis	OFUSG13485.1	100.1052	1.283742	0.359063	3.569805	0.000357	0.003357
Owenia_fusiformis	OFUSG13520.1	1917.733	1.153641	0.189731	6.090199	1.13E-09	6.68E-08
Owenia_fusiformis	OFUSG13534.1	7.789618	1.717682	0.407464	2.901656	0.003712	0.021274
Owenia_fusiformis	OFUSG13544.2	29.23538	1.156316	0.432992	2.752944	0.005906	0.030448
Owenia_fusiformis	OFUSG13581.1	17.45441	1.227768	0.44636	2.832647	0.004616	0.025214
Owenia_fusiformis	OFUSG13587.1	897.0465	1.150176	0.231911	4.9507	7.39E-07	1.88E-05
Owenia_fusiformis	OFUSG13612.1	151.3891	1.03174	0.311061	3.32463	0.000885	0.006893
Owenia_fusiformis	OFUSG13616.1	40.84935	1.39016	0.422836	3.118123	0.00182	0.01227
Owenia_fusiformis	OFUSG13633.1	16.86562	1.697994	0.448281	3.305402	0.000948	0.007274
Owenia_fusiformis	OFUSG13676.1	31.40452	1.556412	0.405668	3.771536	0.000162	0.001777
Owenia_fusiformis	OFUSG13683.1	445.2392	1.228494	0.1923	6.394936	1.61E-10	1.17E-08

Owenia_fusiformis	OFUSG13698.2	86.88257	2.878035	0.441568	6.225505	4.80E-10	3.17E-08
Owenia_fusiformis	OFUSG13701.2	30.2449	1.205034	0.408007	2.897765	0.003758	0.021456
Owenia_fusiformis	OFUSG13734.1	118.1447	1.056566	0.276384	3.819346	0.000134	0.001517
Owenia_fusiformis	OFUSG13783.1	1522.658	1.44077	0.198258	7.265709	3.71E-13	5.54E-11
Owenia_fusiformis	OFUSG13784.1	129.9541	1.269331	0.431002	3.066806	0.002164	0.013989
Owenia_fusiformis	OFUSG13790.1	40.34061	1.354034	0.442199	3.098798	0.001943	0.012898
Owenia_fusiformis	OFUSG13814.1	12.27029	1.239227	0.442784	2.665004	0.007699	0.0373
Owenia_fusiformis	OFUSG13817.3	539.3796	1.134842	0.309026	3.664145	0.000248	0.002513
Owenia_fusiformis	OFUSG13896.2	546.6281	1.121624	0.32099	3.537298	0.000404	0.003695
Owenia_fusiformis	OFUSG13906.1	988.4564	1.012169	0.194585	5.198869	2.01E-07	6.18E-06
Owenia_fusiformis	OFUSG13922.1	29.72144	1.594193	0.436307	3.718812	0.0002	0.00211
Owenia_fusiformis	OFUSG13989.1	438.9214	1.647482	0.198663	8.280279	1.23E-16	3.65E-14
Owenia_fusiformis	OFUSG14031.1	86.30181	1.1007	0.399054	2.760352	0.005774	0.029904
Owenia_fusiformis	OFUSG14068.1	262.2704	1.035165	0.250745	4.127642	3.67E-05	0.000527
Owenia_fusiformis	OFUSG14096.1	13.67178	2.599236	0.445977	4.66661	3.06E-06	6.42E-05
Owenia_fusiformis	OFUSG14160.1	40.42508	1.699946	0.428101	3.9967	6.42E-05	0.000832
Owenia_fusiformis	OFUSG14162.1	220.5529	1.699813	0.309364	5.461143	4.73E-08	1.79E-06
Owenia_fusiformis	OFUSG14172.2	470.2939	1.159075	0.199223	5.819402	5.91E-09	2.88E-07
Owenia_fusiformis	OFUSG14198.1	104.7525	1.832291	0.319862	5.658565	1.53E-08	6.65E-07
Owenia_fusiformis	OFUSG14206.1	2018.313	1.629994	0.179249	9.086651	1.02E-19	4.84E-17
Owenia_fusiformis	OFUSG14224.5	1060.495	1.255364	0.249445	5.039896	4.66E-07	1.27E-05
Owenia_fusiformis	OFUSG14251.1	5746.451	1.061876	0.180459	5.882035	4.05E-09	2.07E-07
Owenia_fusiformis	OFUSG14287.1	11.87628	1.768977	0.441051	3.603687	0.000314	0.003037
Owenia_fusiformis	OFUSG14316.1	1654.005	1.05157	0.18523	5.675895	1.38E-08	6.10E-07
Owenia_fusiformis	OFUSG14317.3	23740.61	1.111974	0.179886	6.175629	6.59E-10	4.20E-08
Owenia_fusiformis	OFUSG14319.1	57.083	2.305347	0.374954	6.111875	9.85E-10	5.97E-08
Owenia_fusiformis	OFUSG14322.1	126.9083	1.147458	0.434462	3.208338	0.001335	0.009538
Owenia_fusiformis	OFUSG14364.1	16.71724	1.438546	0.447947	3.011036	0.002604	0.016126
Owenia_fusiformis	OFUSG14378.5	522.6304	1.077669	0.245116	4.402005	1.07E-05	0.000189
Owenia_fusiformis	OFUSG14491.1	449.4511	1.444937	0.280213	5.127511	2.94E-07	8.55E-06
Owenia_fusiformis	OFUSG14502.1	128.9426	1.983879	0.308997	6.373658	1.85E-10	1.33E-08
Owenia_fusiformis	OFUSG14505.1	96.61338	1.475456	0.339423	4.277326	1.89E-05	0.000302
Owenia_fusiformis	OFUSG14559.1	22.18024	1.177547	0.432037	2.859369	0.004245	0.023647
Owenia_fusiformis	OFUSG14720.1	10.71427	1.496757	0.445353	3.112662	0.001854	0.012442
Owenia_fusiformis	OFUSG14727.1	937.44	1.393507	0.283472	4.978537	6.41E-07	1.66E-05
Owenia_fusiformis	OFUSG14798.1	417.8456	1.003437	0.231921	4.322804	1.54E-05	0.000255
Owenia_fusiformis	OFUSG14800.1	438.137	1.318044	0.257851	5.104863	3.31E-07	9.46E-06
Owenia_fusiformis	OFUSG14836.1	291.9481	1.572479	0.185599	8.464355	2.58E-17	8.57E-15
Owenia_fusiformis	OFUSG14839.1	15.97091	1.652445	0.443515	3.457598	0.000545	0.00468
Owenia_fusiformis	OFUSG14857.1	537.8924	1.064189	0.267831	3.985962	6.72E-05	0.000864
Owenia_fusiformis	OFUSG14904.1	1178.993	1.068979	0.156047	6.847403	7.52E-12	7.78E-10
Owenia_fusiformis	OFUSG14930.1	1047.722	1.165646	0.207254	5.624796	1.86E-08	7.90E-07
Owenia_fusiformis	OFUSG14942.1	59.51273	1.365204	0.392799	3.542516	0.000396	0.003641
Owenia_fusiformis	OFUSG14945.1	69.08859	1.683332	0.446722	5.051367	4.39E-07	1.20E-05
Owenia_fusiformis	OFUSG15009.1	8.357269	1.345504	0.433666	3.82725	0.00013	0.001478
Owenia_fusiformis	OFUSG15014.1	29.48398	1.792517	0.448281	3.240241	0.001194	0.00873
Owenia_fusiformis	OFUSG15038.1	524.0578	1.19311	0.336939	3.534936	0.000408	0.003723
Owenia_fusiformis	OFUSG15097.1	2090.052	1.109486	0.205447	5.397129	6.77E-08	2.44E-06
Owenia_fusiformis	OFUSG15121.1	19.16078	1.619912	0.436973	3.483167	0.000496	0.004346
Owenia_fusiformis	OFUSG15198.1	26.09892	1.207443	0.447759	2.682312	0.007312	0.035933
Owenia_fusiformis	OFUSG15236.1	67.81309	1.079884	0.369452	2.948162	0.003197	0.018943
Owenia_fusiformis	OFUSG15238.1	46.10541	1.335382	0.392471	3.404741	0.000662	0.005471
Owenia_fusiformis	OFUSG15240.1	240.973	1.154846	0.214062	5.40017	6.66E-08	2.41E-06
Owenia_fusiformis	OFUSG15251.1	18.73919	2.123719	0.424129	3.85183	0.000117	0.001361
Owenia_fusiformis	OFUSG15284.1	61.77474	1.222928	0.340766	3.565615	0.000363	0.003403
Owenia_fusiformis	OFUSG15396.1	682.6326	1.543457	0.219368	7.038881	1.94E-12	2.35E-10
Owenia_fusiformis	OFUSG15422.1	19.08545	1.335395	0.441398	2.677543	0.007416	0.03634
Owenia_fusiformis	OFUSG15489.2	449.4613	1.208857	0.218948	5.513186	3.52E-08	1.38E-06
Owenia_fusiformis	OFUSG15531.1	7.333848	1.292675	0.422896	2.73949	0.006153	0.03142
Owenia_fusiformis	OFUSG15576.1	544.2926	1.182402	0.216422	5.461306	4.73E-08	1.79E-06
Owenia_fusiformis	OFUSG15582.1	73.88161	1.121193	0.370326	3.018172	0.002543	0.015841
Owenia_fusiformis	OFUSG15600.2	10.07993	1.612356	0.43481	3.548397	0.000388	0.003588
Owenia_fusiformis	OFUSG15603.2	20.69385	2.383582	0.441397	4.709606	2.48E-06	5.39E-05
Owenia_fusiformis	OFUSG15613.1	20.16495	2.072717	0.446384	4.080874	4.49E-05	0.000625
Owenia_fusiformis	OFUSG15658.1	62.42978	2.6725	0.43398	5.952109	2.65E-09	1.43E-07
Owenia_fusiformis	OFUSG15765.1	220.4701	1.168143	0.26511	4.370958	1.24E-05	0.000212
Owenia_fusiformis	OFUSG15768.1	30.96438	2.393147	0.447792	4.319821	1.56E-05	0.000258
Owenia_fusiformis	OFUSG15793.1	181.8446	1.030754	0.331668	3.13326	0.001729	0.01181
Owenia_fusiformis	OFUSG15795.2	342.3733	1.090227	0.23934	4.583296	4.58E-06	9.16E-05
Owenia_fusiformis	OFUSG15814.1	77.29169	1.909091	0.404593	4.693335	2.69E-06	5.74E-05
Owenia_fusiformis	OFUSG15820.1	388.5912	1.049784	0.284771	3.694148	0.000221	0.00229
Owenia_fusiformis	OFUSG15968.1	1120.847	1.154264	0.17882	6.453836	1.09E-10	8.31E-09
Owenia_fusiformis	OFUSG16023.1	150.6868	1.692179	0.348509	4.916053	8.83E-07	2.19E-05
Owenia_fusiformis	OFUSG16027.1	37.27748	1.55013	0.429808	3.671633	0.000241	0.002461
Owenia_fusiformis	OFUSG16077.1	6.866816	1.299506	0.393746	2.749403	0.00597	0.030696
Owenia_fusiformis	OFUSG16085.1	7.947116	1.194479	0.44143	2.545024	0.010927	0.048724
Owenia_fusiformis	OFUSG16087.1	87.48381	1.576719	0.368387	4.338903	1.43E-05	0.00024
Owenia_fusiformis	OFUSG16094.1	14.90275	1.6493	0.400257	4.134947	3.55E-05	0.000513
Owenia_fusiformis	OFUSG16118.1	1157.88	1.465387	0.317933	4.542467	5.56E-06	0.000108
Owenia_fusiformis	OFUSG16131.1	78.35844	1.381657	0.417449	3.152705	0.001618	0.011212

Owenia_fusiformis	OFUSG16140.1	118.0858	1.25375	0.342229	3.657476	0.000255	0.002561
Owenia_fusiformis	OFUSG16147.1	702.2895	1.018586	0.182115	5.592412	2.24E-08	9.27E-07
Owenia_fusiformis	OFUSG16183.1	102.751	1.406723	0.296121	4.730498	2.24E-06	4.94E-05
Owenia_fusiformis	OFUSG16206.1	9.884907	1.047878	0.429659	2.877549	0.004008	0.02262
Owenia_fusiformis	OFUSG16237.1	7054.592	1.027267	0.136969	7.50081	6.34E-14	1.08E-11
Owenia_fusiformis	OFUSG16240.1	27.34364	2.554134	0.441321	5.257239	1.46E-07	4.72E-06
Owenia_fusiformis	OFUSG16288.1	6.705899	1.245199	0.319022	2.676411	0.007442	0.036405
Owenia_fusiformis	OFUSG16314.1	43.3352	1.269386	0.403494	3.043898	0.002335	0.014854
Owenia_fusiformis	OFUSG16371.1	15.31975	1.120123	0.43618	3.180188	0.001472	0.010353
Owenia_fusiformis	OFUSG16390.1	14672.39	1.245489	0.172713	7.220657	5.17E-13	7.24E-11
Owenia_fusiformis	OFUSG16391.1	3614.582	1.649037	0.217929	7.577514	3.52E-14	6.24E-12
Owenia_fusiformis	OFUSG16453.1	16.61963	1.218737	0.447654	2.989106	0.002798	0.017025
Owenia_fusiformis	OFUSG16597.1	8182.863	1.39017	0.233993	5.986922	2.14E-09	1.19E-07
Owenia_fusiformis	OFUSG16604.1	24.13083	2.09545	0.445713	4.152255	3.29E-05	0.000483
Owenia_fusiformis	OFUSG16675.2	310.433	1.599778	0.229992	6.92912	4.23E-12	4.70E-10
Owenia_fusiformis	OFUSG16761.1	9.824527	1.239746	0.440477	3.0635	0.002188	0.014107
Owenia_fusiformis	OFUSG16778.1	9.866445	1.203052	0.385495	3.667706	0.000245	0.002492
Owenia_fusiformis	OFUSG16822.1	48.60945	1.565104	0.434436	3.857653	0.000114	0.001337
Owenia_fusiformis	OFUSG16829.1	36.86033	1.127375	0.416172	2.86695	0.004144	0.023218
Owenia_fusiformis	OFUSG16848.1	373.1075	2.331024	0.3994	6.388435	1.68E-10	1.22E-08
Owenia_fusiformis	OFUSG16897.1	25.03435	1.558878	0.446606	3.669934	0.000243	0.002473
Owenia_fusiformis	OFUSG16899.1	209.3984	1.226229	0.35435	3.485895	0.00049	0.004311
Owenia_fusiformis	OFUSG16919.1	42.72212	1.143666	0.424302	2.677448	0.007419	0.036343
Owenia_fusiformis	OFUSG17047.1	8.403243	2.103582	0.389134	4.115641	3.86E-05	0.000551
Owenia_fusiformis	OFUSG17058.1	22.18367	1.237	0.436016	2.738493	0.006172	0.03149
Owenia_fusiformis	OFUSG17076.2	209.7965	2.029233	0.392437	5.15961	2.47E-07	7.43E-06
Owenia_fusiformis	OFUSG17092.1	256.9309	1.164264	0.213969	5.437192	5.41E-08	2.01E-06
Owenia_fusiformis	OFUSG17094.1	38.54964	1.220341	0.404745	3.020593	0.002523	0.015746
Owenia_fusiformis	OFUSG17117.2	31.15205	1.060179	0.441285	2.622976	0.008717	0.04099
Owenia_fusiformis	OFUSG17160.1	622.6631	1.181497	0.329853	3.588844	0.000332	0.003174
Owenia_fusiformis	OFUSG17175.1	23.01245	2.306946	0.445684	4.638004	3.52E-06	7.25E-05
Owenia_fusiformis	OFUSG17176.1	5.865609	1.282713	0.406923	3.590204	0.00033	0.00316
Owenia_fusiformis	OFUSG17248.1	2535.896	2.058208	0.442314	4.028346	5.62E-05	0.000747
Owenia_fusiformis	OFUSG17259.1	50.94829	2.008842	0.447954	4.0996	4.14E-05	0.000583
Owenia_fusiformis	OFUSG17263.1	66.92594	2.890688	0.34944	7.975443	1.52E-15	3.78E-13
Owenia_fusiformis	OFUSG17297.1	1874.562	1.285987	0.168507	7.632266	2.31E-14	4.35E-12
Owenia_fusiformis	OFUSG17299.1	56341.01	1.18315	0.210539	5.626428	1.84E-08	7.84E-07
Owenia_fusiformis	OFUSG17314.1	156.3623	1.021563	0.331022	3.130773	0.001743	0.011887
Owenia_fusiformis	OFUSG17323.1	15.00979	1.222817	0.44861	2.922767	0.003469	0.020175
Owenia_fusiformis	OFUSG17352.3	175.3435	1.093481	0.447836	2.926611	0.003427	0.019988
Owenia_fusiformis	OFUSG17405.1	159.6678	1.368149	0.28309	4.806708	1.53E-06	3.56E-05
Owenia_fusiformis	OFUSG17410.2	24.98429	1.392965	0.434423	2.97777	0.002904	0.017517
Owenia_fusiformis	OFUSG17419.1	11.31047	1.183575	0.415228	2.839694	0.004516	0.024823
Owenia_fusiformis	OFUSG17440.1	14.67192	1.383776	0.442611	2.749187	0.005974	0.0307
Owenia_fusiformis	OFUSG17442.1	89.97155	1.522159	0.334977	4.502602	6.71E-06	0.000128
Owenia_fusiformis	OFUSG17476.1	99.81407	1.354644	0.270865	5.011569	5.40E-07	1.42E-05
Owenia_fusiformis	OFUSG17520.1	26.91859	1.792036	0.433838	3.85403	0.000116	0.001352
Owenia_fusiformis	OFUSG17530.1	160.1348	1.610679	0.448395	4.879926	1.06E-06	2.57E-05
Owenia_fusiformis	OFUSG17531.1	1389.587	1.347093	0.243553	5.541541	3.00E-08	1.20E-06
Owenia_fusiformis	OFUSG17544.1	699.3979	1.147675	0.212677	5.397722	6.75E-08	2.44E-06
Owenia_fusiformis	OFUSG17551.1	12.33482	1.262393	0.433491	3.053357	0.002263	0.01449
Owenia_fusiformis	OFUSG17595.2	223.7413	1.045702	0.399222	2.666081	0.007674	0.037218
Owenia_fusiformis	OFUSG17602.1	11.39758	1.498808	0.445597	3.315013	0.000916	0.007077
Owenia_fusiformis	OFUSG17616.1	483.0125	1.026049	0.213382	4.805595	1.54E-06	3.57E-05
Owenia_fusiformis	OFUSG17704.1	87.06769	1.125474	0.355828	3.115357	0.001837	0.01236
Owenia_fusiformis	OFUSG17714.1	14.15052	1.065732	0.435997	3.037867	0.002383	0.015096
Owenia_fusiformis	OFUSG17716.1	58.67785	1.195068	0.432208	2.887314	0.003885	0.022063
Owenia_fusiformis	OFUSG17718.1	95.12226	1.68235	0.314255	5.356356	8.49E-08	2.94E-06
Owenia_fusiformis	OFUSG17724.1	28.782	1.768372	0.415193	3.897272	9.73E-05	0.001169
Owenia_fusiformis	OFUSG17750.2	425.679	1.310381	0.302182	4.3172	1.58E-05	0.00026
Owenia_fusiformis	OFUSG17781.1	23.57655	1.72755	0.435922	3.723022	0.000197	0.002081
Owenia_fusiformis	OFUSG17784.1	3143.925	1.91015	0.260171	7.346873	2.03E-13	3.13E-11
Owenia_fusiformis	OFUSG17796.1	15.79033	1.141841	0.438912	2.642763	0.008223	0.039232
Owenia_fusiformis	OFUSG17797.1	154.602	1.086825	0.318675	3.449333	0.000562	0.004789
Owenia_fusiformis	OFUSG17798.1	227.3519	1.01168	0.256605	3.923961	8.71E-05	0.00107
Owenia_fusiformis	OFUSG17853.1	370.2324	1.013381	0.179439	5.647253	1.63E-08	7.04E-07
Owenia_fusiformis	OFUSG17877.1	112.1444	1.154654	0.33341	3.492458	0.000479	0.004226
Owenia_fusiformis	OFUSG17899.1	11.08349	1.857926	0.440014	3.663908	0.000248	0.002513
Owenia_fusiformis	OFUSG17945.1	128.1791	1.527627	0.378167	4.039424	5.36E-05	0.000719
Owenia_fusiformis	OFUSG17985.1	17.43198	1.606593	0.447286	3.331857	0.000863	0.006764
Owenia_fusiformis	OFUSG18037.1	1559.582	1.511906	0.202569	7.463676	8.41E-14	1.38E-11
Owenia_fusiformis	OFUSG18171.1	1388.223	1.261917	0.137823	9.155767	5.40E-20	2.71E-17
Owenia_fusiformis	OFUSG18228.1	126.0876	1.147659	0.313829	3.681726	0.000232	0.002384
Owenia_fusiformis	OFUSG18233.1	6.28836	1.260688	0.399905	2.610348	0.009045	0.042068
Owenia_fusiformis	OFUSG18235.1	54.94806	1.437758	0.418459	3.191939	0.001413	0.010001
Owenia_fusiformis	OFUSG18292.1	36.33045	1.358619	0.420499	3.220558	0.001279	0.009224
Owenia_fusiformis	OFUSG18329.1	5.564059	1.14767	0.419778	2.576089	0.009992	0.045462
Owenia_fusiformis	OFUSG18341.1	11.81433	1.688001	0.401999	2.975125	0.002929	0.017647
Owenia_fusiformis	OFUSG18380.3	207.9924	1.079719	0.307913	3.49066	0.000482	0.004248
Owenia_fusiformis	OFUSG18405.1	70.61431	1.951229	0.397567	4.90553	9.32E-07	2.30E-05

Owenia_fusiformis	OFUSG18429.1	37936.26	1.657186	0.2438	6.84787	7.50E-12	7.78E-10
Owenia_fusiformis	OFUSG18429.2	133.5925	1.474413	0.41396	3.381511	0.000721	0.005849
Owenia_fusiformis	OFUSG18449.3	238.3658	1.156796	0.3165	3.591779	0.000328	0.003142
Owenia_fusiformis	OFUSG18463.1	1249.014	1.125688	0.236111	4.770783	1.84E-06	4.16E-05
Owenia_fusiformis	OFUSG18487.1	30.35747	2.377984	0.428805	5.463118	4.68E-08	1.78E-06
Owenia_fusiformis	OFUSG18557.2	31.30048	1.301228	0.421345	3.06948	0.002144	0.013904
Owenia_fusiformis	OFUSG18560.1	533.5205	1.931986	0.448676	3.476969	0.000507	0.004429
Owenia_fusiformis	OFUSG18575.1	15.68969	1.231193	0.446714	2.874925	0.004041	0.02276
Owenia_fusiformis	OFUSG18579.1	115.1072	1.78478	0.334748	5.370306	7.86E-08	2.78E-06
Owenia_fusiformis	OFUSG18583.1	7.866924	1.394793	0.40307	3.174846	0.001499	0.010512
Owenia_fusiformis	OFUSG18594.1	21.54904	2.200575	0.44817	4.35156	1.35E-05	0.000229
Owenia_fusiformis	OFUSG18649.1	1859.986	1.367049	0.265605	5.15604	2.52E-07	7.55E-06
Owenia_fusiformis	OFUSG18665.1	68.12499	1.939942	0.384458	5.035718	4.76E-07	1.28E-05
Owenia_fusiformis	OFUSG18686.1	25.17154	1.524779	0.439527	3.305564	0.000948	0.007272
Owenia_fusiformis	OFUSG18698.1	634.1003	1.215405	0.174287	6.96653	3.25E-12	3.74E-10
Owenia_fusiformis	OFUSG18767.1	228.2268	1.428211	0.229048	6.231653	4.62E-10	3.06E-08
Owenia_fusiformis	OFUSG18768.1	447.0081	1.0817	0.241041	4.49113	7.08E-06	0.000133
Owenia_fusiformis	OFUSG18776.1	9.644811	1.325956	0.443643	2.818195	0.004829	0.026076
Owenia_fusiformis	OFUSG18859.2	277.8109	1.663949	0.396079	4.055402	5.00E-05	0.000682
Owenia_fusiformis	OFUSG18862.1	1417.179	1.070346	0.15314	6.988348	2.78E-12	3.25E-10
Owenia_fusiformis	OFUSG18896.1	45.56031	1.074993	0.388354	2.724105	0.006448	0.032706
Owenia_fusiformis	OFUSG18903.1	144.9263	1.181911	0.262962	4.499233	6.82E-06	0.000129
Owenia_fusiformis	OFUSG18989.2	13.56212	1.74797	0.447207	3.516611	0.000437	0.003921
Owenia_fusiformis	OFUSG19000.2	283.8546	1.623374	0.324787	5.059493	4.20E-07	1.16E-05
Owenia_fusiformis	OFUSG19023.1	45.54508	1.5145	0.406631	3.486154	0.00049	0.004308
Owenia_fusiformis	OFUSG19045.1	33.57701	2.180361	0.425318	4.695993	2.65E-06	5.68E-05
Owenia_fusiformis	OFUSG19057.1	135.6444	1.047723	0.34363	3.023054	0.002502	0.015657
Owenia_fusiformis	OFUSG19092.1	21.58515	1.187285	0.448502	2.655604	0.007917	0.038057
Owenia_fusiformis	OFUSG19140.1	155.6268	1.549134	0.404061	4.024015	5.72E-05	0.000757
Owenia_fusiformis	OFUSG19142.1	60.03503	1.172503	0.37651	3.093034	0.001981	0.01309
Owenia_fusiformis	OFUSG19166.1	508.9864	1.429299	0.21649	6.601558	4.07E-11	3.50E-09
Owenia_fusiformis	OFUSG19168.1	1119.981	1.108838	0.192926	5.748371	9.01E-09	4.21E-07
Owenia_fusiformis	OFUSG19170.5	1323.066	1.271157	0.218942	5.8125	6.15E-09	2.99E-07
Owenia_fusiformis	OFUSG19186.2	55.68784	1.101886	0.379447	4.910286	9.09E-07	2.25E-05
Owenia_fusiformis	OFUSG19241.1	273.6073	2.011106	0.286361	7.022286	2.18E-12	2.62E-10
Owenia_fusiformis	OFUSG19288.1	23.08784	2.025712	0.434414	4.356488	1.32E-05	0.000225
Owenia_fusiformis	OFUSG19289.2	78.40544	1.117497	0.396536	2.78693	0.005321	0.028114
Owenia_fusiformis	OFUSG19301.1	69.21266	1.138454	0.342544	3.342503	0.00083	0.006557
Owenia_fusiformis	OFUSG19305.1	237.6187	1.357761	0.401466	3.154225	0.001609	0.011167
Owenia_fusiformis	OFUSG19306.2	35.85994	1.488935	0.436707	3.172439	0.001512	0.010583
Owenia_fusiformis	OFUSG19309.1	32.0017	1.836877	0.420628	4.198841	2.68E-05	0.000406
Owenia_fusiformis	OFUSG19391.1	7.916668	1.005024	0.381002	2.6512	0.008021	0.038406
Owenia_fusiformis	OFUSG19413.1	14464.05	1.107808	0.178936	6.203388	5.53E-10	3.62E-08
Owenia_fusiformis	OFUSG19459.2	807.4534	1.1245	0.260045	4.338025	1.44E-05	0.000241
Owenia_fusiformis	OFUSG19471.1	46.2579	1.63991	0.415501	3.624414	0.00029	0.002847
Owenia_fusiformis	OFUSG19491.1	17.1208	1.342225	0.446195	2.785999	0.005336	0.028141
Owenia_fusiformis	OFUSG19541.1	350.5662	1.002675	0.292563	3.465871	0.000529	0.004569
Owenia_fusiformis	OFUSG19557.1	71.82579	1.211956	0.370127	3.341715	0.000833	0.006564
Owenia_fusiformis	OFUSG19567.1	456.4298	1.019457	0.20578	4.955072	7.23E-07	1.85E-05
Owenia_fusiformis	OFUSG19573.1	605.4465	1.478021	0.300143	4.943738	7.66E-07	1.94E-05
Owenia_fusiformis	OFUSG19581.1	61.88434	1.238208	0.428347	2.94697	0.003209	0.018993
Owenia_fusiformis	OFUSG19583.1	73.79715	1.252121	0.307741	4.062819	4.85E-05	0.000664
Owenia_fusiformis	OFUSG19653.1	8.668176	2.008876	0.41395	3.316406	0.000912	0.007053
Owenia_fusiformis	OFUSG19661.1	2104.546	1.696513	0.30204	5.68465	1.31E-08	5.84E-07
Owenia_fusiformis	OFUSG19671.1	223.0766	2.498599	0.391508	6.006695	1.89E-09	1.07E-07
Owenia_fusiformis	OFUSG19674.1	334.0516	1.0139	0.220284	4.599027	4.24E-06	8.57E-05
Owenia_fusiformis	OFUSG19687.1	1611.451	1.254543	0.185114	6.778202	1.22E-11	1.21E-09
Owenia_fusiformis	OFUSG19700.2	15.55225	1.578556	0.44845	3.288463	0.001007	0.007616
Owenia_fusiformis	OFUSG19723.1	7.617439	1.883571	0.419065	3.530728	0.000414	0.003767
Owenia_fusiformis	OFUSG19736.1	146.6009	1.172683	0.319443	3.663034	0.000249	0.002519
Owenia_fusiformis	OFUSG19743.1	322.5495	1.036474	0.305934	3.37507	0.000738	0.005963
Owenia_fusiformis	OFUSG19759.1	22.32143	1.08139	0.430075	2.540613	0.011066	0.0492
Owenia_fusiformis	OFUSG19861.1	326.6063	1.116236	0.255591	4.351974	1.35E-05	0.000229
Owenia_fusiformis	OFUSG19879.1	44.45831	1.163303	0.403916	2.882025	0.003951	0.022359
Owenia_fusiformis	OFUSG19891.1	16.23479	1.589889	0.44788	3.711649	0.000206	0.002161
Owenia_fusiformis	OFUSG19904.1	1120.924	1.147138	0.147913	7.754891	8.84E-15	1.89E-12
Owenia_fusiformis	OFUSG19909.1	33.91202	1.4037	0.418095	3.21806	0.001291	0.009291
Owenia_fusiformis	OFUSG19949.1	15.23394	2.453436	0.44542	4.657633	3.20E-06	6.66E-05
Owenia_fusiformis	OFUSG19974.1	419.7427	1.120838	0.263623	4.254505	2.10E-05	0.00033
Owenia_fusiformis	OFUSG20006.1	88.75262	1.097682	0.324526	3.369137	0.000754	0.006065
Owenia_fusiformis	OFUSG20019.1	150.9809	3.187078	0.41495	7.678879	1.60E-14	3.14E-12
Owenia_fusiformis	OFUSG20020.1	46.78756	3.891242	0.425283	7.226407	4.96E-13	7.02E-11
Owenia_fusiformis	OFUSG20021.2	54.95508	1.146564	0.37861	3.018134	0.002543	0.015841
Owenia_fusiformis	OFUSG20054.1	11.04674	1.607974	0.448576	3.132273	0.001735	0.011846
Owenia_fusiformis	OFUSG20109.1	9.703481	1.389505	0.430548	2.948387	0.003194	0.018939
Owenia_fusiformis	OFUSG20138.1	31.38623	1.475637	0.405395	3.540007	0.0004	0.003664
Owenia_fusiformis	OFUSG20202.1	72.53857	2.390536	0.34374	6.841148	7.86E-12	8.10E-10
Owenia_fusiformis	OFUSG20249.1	140.8045	1.011657	0.289323	3.484365	0.000493	0.004334
Owenia_fusiformis	OFUSG20253.1	818.941	1.039993	0.212487	4.891361	1.00E-06	2.44E-05
Owenia_fusiformis	OFUSG20304.1	9.191831	1.296664	0.446752	2.723449	0.00646	0.032744

Owenia_fusiformis	OFUSG20310.1	34.21084	1.603922	0.422547	3.870885	0.000108	0.00128
Owenia_fusiformis	OFUSG20319.1	133.6653	1.392796	0.332833	4.209603	2.56E-05	0.00039
Owenia_fusiformis	OFUSG20322.1	3345.74	1.015383	0.157157	6.46332	1.02E-10	7.93E-09
Owenia_fusiformis	OFUSG20329.1	20.02286	2.159414	0.44853	4.227802	2.36E-05	0.000365
Owenia_fusiformis	OFUSG20342.1	334.5981	1.120124	0.28592	3.939789	8.16E-05	0.001015
Owenia_fusiformis	OFUSG20507.1	2752.274	2.28131	0.13027	17.50735	1.26E-68	3.10E-64
Owenia_fusiformis	OFUSG20526.1	67.99717	1.720056	0.397872	4.27533	1.91E-05	0.000304
Owenia_fusiformis	OFUSG20586.1	43.47001	1.92019	0.44728	5.161071	2.46E-07	7.39E-06
Owenia_fusiformis	OFUSG20590.1	115.5362	1.35239	0.377174	3.640511	0.000272	0.002702
Owenia_fusiformis	OFUSG20596.1	11.19914	1.452774	0.433229	3.186201	0.001442	0.010172
Owenia_fusiformis	OFUSG20598.1	376.3126	2.226889	0.273081	8.11225	4.97E-16	1.37E-13
Owenia_fusiformis	OFUSG20601.1	18.15702	1.354945	0.447228	3.079964	0.00207	0.013534
Owenia_fusiformis	OFUSG20610.1	241.3009	2.022401	0.252616	7.970459	1.58E-15	3.89E-13
Owenia_fusiformis	OFUSG20615.1	40.93343	1.50461	0.448439	3.759253	0.00017	0.001851
Owenia_fusiformis	OFUSG20615.4	125.3626	1.118855	0.401657	2.898243	0.003753	0.021432
Owenia_fusiformis	OFUSG20649.1	12.50724	1.323829	0.439393	3.13635	0.001711	0.011709
Owenia_fusiformis	OFUSG20658.1	46.17715	3.650573	0.434122	7.207864	5.68E-13	7.89E-11
Owenia_fusiformis	OFUSG20662.1	145.01	1.304438	0.281452	4.634854	3.57E-06	7.34E-05
Owenia_fusiformis	OFUSG20662.2	291.7911	1.081417	0.277349	3.892563	9.92E-05	0.001189
Owenia_fusiformis	OFUSG20670.1	48.1622	1.05861	0.375438	2.823073	0.004757	0.025808
Owenia_fusiformis	OFUSG20705.2	63.10724	1.986287	0.368494	5.283023	1.27E-07	4.18E-06
Owenia_fusiformis	OFUSG20766.1	14.53522	1.512043	0.448658	3.103094	0.001915	0.012747
Owenia_fusiformis	OFUSG20784.1	198.3491	2.674816	0.269471	9.83591	7.89E-23	5.55E-20
Owenia_fusiformis	OFUSG20804.2	194.9323	1.280137	0.271455	4.76413	1.90E-06	4.29E-05
Owenia_fusiformis	OFUSG20811.1	9.393259	2.202239	0.416667	4.02754	5.64E-05	0.000748
Owenia_fusiformis	OFUSG20839.1	218.2752	1.141593	0.333927	3.452525	0.000555	0.004749
Owenia_fusiformis	OFUSG20880.1	351.1455	1.104082	0.320406	3.447286	0.000566	0.00481
Owenia_fusiformis	OFUSG20882.1	62.02605	1.0362	0.336966	3.068695	0.00215	0.013922
Owenia_fusiformis	OFUSG20909.1	115.672	1.150774	0.333	3.455561	0.000549	0.004709
Owenia_fusiformis	OFUSG20942.1	106.2869	1.006053	0.312485	3.220612	0.001279	0.009224
Owenia_fusiformis	OFUSG21110.1	2359.029	1.186946	0.176949	6.713171	1.90E-11	1.78E-09
Owenia_fusiformis	OFUSG21113.1	37.85557	1.343713	0.438847	3.323164	0.00089	0.006917
Owenia_fusiformis	OFUSG21162.1	1019.51	1.331811	0.175684	7.579906	3.46E-14	6.17E-12
Owenia_fusiformis	OFUSG21172.2	50.83502	1.015109	0.372669	2.710953	0.006709	0.033609
Owenia_fusiformis	OFUSG21246.1	234.2013	1.016332	0.233351	4.354906	1.33E-05	0.000226
Owenia_fusiformis	OFUSG21247.1	105.524	1.773705	0.323469	5.459773	4.77E-08	1.80E-06
Owenia_fusiformis	OFUSG21259.1	24.35281	1.632611	0.44135	2.896608	0.003772	0.021529
Owenia_fusiformis	OFUSG21268.1	239.0878	3.417639	0.282386	12.00686	3.27E-33	5.65E-30
Owenia_fusiformis	OFUSG21276.1	83.49554	1.37377	0.347896	3.986274	6.71E-05	0.000864
Owenia_fusiformis	OFUSG21297.1	152.8699	1.183764	0.299481	3.962185	7.43E-05	0.000938
Owenia_fusiformis	OFUSG21337.1	532.4126	1.160605	0.20264	5.72086	1.06E-08	4.83E-07
Owenia_fusiformis	OFUSG21399.1	19.28233	1.168086	0.436085	2.627048	0.008613	0.040619
Owenia_fusiformis	OFUSG21487.1	144.8732	1.6823	0.343757	4.927284	8.34E-07	2.08E-05
Owenia_fusiformis	OFUSG21508.1	8.515521	1.36428	0.434792	2.908784	0.003628	0.020912
Owenia_fusiformis	OFUSG21523.1	26.53228	1.386989	0.440868	3.220338	0.00128	0.009228
Owenia_fusiformis	OFUSG21555.1	395.8171	1.11167	0.222631	4.993362	5.93E-07	1.55E-05
Owenia_fusiformis	OFUSG21556.1	573.5506	1.388727	0.210196	6.610011	3.84E-11	3.36E-09
Owenia_fusiformis	OFUSG21558.1	345.2285	1.046405	0.304972	3.480615	0.0005	0.004378
Owenia_fusiformis	OFUSG21602.1	444.364	1.123784	0.184395	6.094739	1.10E-09	6.52E-08
Owenia_fusiformis	OFUSG21901.2	1536.486	1.009487	0.154379	6.544202	5.98E-11	4.89E-09
Owenia_fusiformis	OFUSG21912.1	23.38501	1.577066	0.44806	3.24707	0.001166	0.008564
Owenia_fusiformis	OFUSG21915.1	456.9326	1.17797	0.231059	5.101634	3.37E-07	9.59E-06
Owenia_fusiformis	OFUSG21920.1	44.24138	1.707148	0.443947	3.70328	0.000213	0.002223
Owenia_fusiformis	OFUSG21945.1	290.4881	1.152573	0.220541	5.224081	1.75E-07	5.53E-06
Owenia_fusiformis	OFUSG21970.1	20.99249	1.238975	0.437379	2.669613	0.007594	0.036952
Owenia_fusiformis	OFUSG22070.1	523.4847	1.361869	0.231089	5.893501	3.78E-09	1.95E-07
Owenia_fusiformis	OFUSG22085.1	132.5629	1.812391	0.314666	5.780142	7.46E-09	3.56E-07
Owenia_fusiformis	OFUSG22095.1	266.2999	1.105336	0.266233	4.182712	2.88E-05	0.000432
Owenia_fusiformis	OFUSG22096.1	75.11316	1.271098	0.391739	3.143846	0.001667	0.01149
Owenia_fusiformis	OFUSG22118.1	21106.47	1.10519	0.132524	8.342818	7.25E-17	2.29E-14
Owenia_fusiformis	OFUSG22163.1	1467.183	1.1929	0.203471	5.845729	5.04E-09	2.51E-07
Owenia_fusiformis	OFUSG22227.1	113.6685	1.23935	0.285788	4.326144	1.52E-05	0.000252
Owenia_fusiformis	OFUSG22305.1	111.7919	1.351154	0.445836	2.909812	0.003616	0.020882
Owenia_fusiformis	OFUSG22305.2	5191.418	1.365497	0.186742	7.315685	2.56E-13	3.89E-11
Owenia_fusiformis	OFUSG22381.1	13.37884	1.861713	0.424742	3.684354	0.000229	0.002361
Owenia_fusiformis	OFUSG22385.1	12.07756	1.888662	0.448106	4.055502	5.00E-05	0.000682
Owenia_fusiformis	OFUSG22427.1	1384.998	1.5653	0.216512	7.23889	4.52E-13	6.51E-11
Owenia_fusiformis	OFUSG22435.1	107.4248	1.466514	0.360064	3.957545	7.57E-05	0.000953
Owenia_fusiformis	OFUSG22440.1	93.87482	2.459201	0.389105	6.235373	4.51E-10	3.01E-08
Owenia_fusiformis	OFUSG22486.1	29.18205	1.276632	0.431652	2.981803	0.002866	0.017343
Owenia_fusiformis	OFUSG22523.1	26.48551	1.437605	0.426914	3.267654	0.001084	0.008083
Owenia_fusiformis	OFUSG22596.1	60.81223	1.10772	0.363203	3.063064	0.002191	0.014117
Owenia_fusiformis	OFUSG22636.1	12.55028	1.58769	0.441275	3.147858	0.001645	0.011358
Owenia_fusiformis	OFUSG22659.1	3252.828	1.043671	0.239653	4.359391	1.30E-05	0.000223
Owenia_fusiformis	OFUSG22663.1	9.43124	1.210211	0.441083	2.669136	0.007605	0.036968
Owenia_fusiformis	OFUSG22728.1	120.378	1.464521	0.33892	4.294304	1.75E-05	0.000284
Owenia_fusiformis	OFUSG22735.1	100.8607	1.133203	0.324236	3.513207	0.000443	0.00396
Owenia_fusiformis	OFUSG22737.1	52.4012	1.122373	0.379224	2.900197	0.003729	0.021333
Owenia_fusiformis	OFUSG22771.1	1735.85	1.203419	0.153274	7.852557	4.08E-15	9.38E-13
Owenia_fusiformis	OFUSG22786.1	30.92883	1.087133	0.448576	2.887445	0.003884	0.022059

Owenia_fusiformis	OFUSG22812.1	34.96704	1.509865	0.406866	3.542539	0.000396	0.003641
Owenia_fusiformis	OFUSG22819.1	1643.91	1.094686	0.133981	8.170361	3.07E-16	8.70E-14
Owenia_fusiformis	OFUSG22821.1	11.26467	1.032008	0.435192	2.683391	0.007288	0.035839
Owenia_fusiformis	OFUSG22912.1	12.82172	1.045688	0.445179	2.609634	0.009064	0.04214
Owenia_fusiformis	OFUSG22915.1	397.0604	1.440693	0.343175	4.262885	2.02E-05	0.00032
Owenia_fusiformis	OFUSG22920.1	13.66254	1.185162	0.448517	2.796398	0.005168	0.02752
Owenia_fusiformis	OFUSG22927.1	24.0464	3.087882	0.447706	5.646882	1.63E-08	7.05E-07
Owenia_fusiformis	OFUSG22970.1	574.5613	1.037274	0.182779	5.673414	1.40E-08	6.18E-07
Owenia_fusiformis	OFUSG23003.1	6.39046	1.035352	0.339909	2.561772	0.010414	0.046954
Owenia_fusiformis	OFUSG23007.1	378.0618	1.672432	0.197468	8.459098	2.69E-17	8.85E-15
Owenia_fusiformis	OFUSG23024.1	411.6157	1.104918	0.239802	4.596226	4.30E-06	8.68E-05
Owenia_fusiformis	OFUSG23043.1	120.7628	1.031295	0.416869	2.752156	0.00592	0.030503
Owenia_fusiformis	OFUSG23045.1	5.096477	1.135876	0.375869	3.279107	0.001041	0.007816
Owenia_fusiformis	OFUSG23047.1	85.75989	1.074454	0.391699	2.749156	0.005975	0.0307
Owenia_fusiformis	OFUSG23057.1	12.16114	1.696783	0.438417	3.401174	0.000671	0.005525
Owenia_fusiformis	OFUSG23080.1	4.094865	1.142055	0.358265	2.584331	0.009757	0.044662
Owenia_fusiformis	OFUSG23081.1	8.021488	1.31358	0.433536	2.983081	0.002854	0.017301
Owenia_fusiformis	OFUSG23115.1	15.19652	1.327166	0.437609	2.895235	0.003789	0.021613
Owenia_fusiformis	OFUSG23124.1	13.21571	1.178214	0.435338	2.554402	0.010637	0.047698
Owenia_fusiformis	OFUSG23143.1	27.21204	1.969953	0.4242	4.485404	7.28E-06	0.000136
Owenia_fusiformis	OFUSG23182.2	84.96082	1.417672	0.348428	4.075265	4.60E-05	0.000637
Owenia_fusiformis	OFUSG23191.2	68.68183	2.069062	0.426771	4.620819	3.82E-06	7.81E-05
Owenia_fusiformis	OFUSG23224.2	434.0857	1.496004	0.232451	6.439636	1.20E-10	8.96E-09
Owenia_fusiformis	OFUSG23235.1	62.8229	1.295775	0.340229	3.791394	0.00015	0.001666
Owenia_fusiformis	OFUSG23249.1	106.5038	2.110762	0.342726	6.065745	1.31E-09	7.61E-08
Owenia_fusiformis	OFUSG23250.1	88.66266	1.286299	0.295931	4.333983	1.46E-05	0.000244
Owenia_fusiformis	OFUSG23249.2	358.1947	2.218025	0.282101	7.827567	4.97E-15	1.11E-12
Owenia_fusiformis	OFUSG23253.1	18.91985	1.348528	0.447195	2.852907	0.004332	0.024002
Owenia_fusiformis	OFUSG23261.1	9.534713	1.508932	0.4058	2.593079	0.009512	0.043786
Owenia_fusiformis	OFUSG23272.2	212.8149	1.383818	0.243296	5.689641	1.27E-08	5.69E-07
Owenia_fusiformis	OFUSG23283.1	37.36844	1.149267	0.396869	2.852068	0.004344	0.024049
Owenia_fusiformis	OFUSG23287.1	38.9243	1.110021	0.373839	2.944299	0.003237	0.019112
Owenia_fusiformis	OFUSG23293.1	25.1834	2.16271	0.446998	3.798823	0.000145	0.001626
Owenia_fusiformis	OFUSG23297.1	84.33597	1.034968	0.31355	3.291155	0.000998	0.007563
Owenia_fusiformis	OFUSG23326.1	51.08484	1.247056	0.398844	3.110354	0.001869	0.012507
Owenia_fusiformis	OFUSG23413.1	2991.329	1.671098	0.234812	7.092781	1.31E-12	1.67E-10
Owenia_fusiformis	OFUSG23415.1	62.82117	2.057003	0.387579	5.277509	1.31E-07	4.27E-06
Owenia_fusiformis	OFUSG23428.1	87.97778	2.296808	0.415583	5.237441	1.63E-07	5.19E-06
Owenia_fusiformis	OFUSG23435.1	19.54745	1.232797	0.448428	2.786076	0.005335	0.028141
Owenia_fusiformis	OFUSG23438.2	97.0612	1.453307	0.413205	3.400098	0.000674	0.005545
Owenia_fusiformis	OFUSG23441.1	21.07079	1.504918	0.448512	3.110008	0.001871	0.01251
Owenia_fusiformis	OFUSG23457.1	1041.679	1.16893	0.224005	5.219305	1.80E-07	5.64E-06
Owenia_fusiformis	OFUSG23521.1	2224.932	1.087972	0.17467	6.23313	4.57E-10	3.04E-08
Owenia_fusiformis	OFUSG23564.1	402.3627	1.118065	0.304707	3.656317	0.000256	0.002566
Owenia_fusiformis	OFUSG23576.1	209.7947	2.005384	0.448697	3.515453	0.000439	0.003934
Owenia_fusiformis	OFUSG23577.1	7.896436	1.955311	0.431585	3.484031	0.000494	0.004335
Owenia_fusiformis	OFUSG23588.1	459.8899	1.246509	0.224786	5.543032	2.97E-08	1.19E-06
Owenia_fusiformis	OFUSG23596.2	1687.116	1.018581	0.166127	6.13116	8.72E-10	5.33E-08
Owenia_fusiformis	OFUSG23673.1	29.02545	1.898576	0.423969	4.242701	2.21E-05	0.000345
Owenia_fusiformis	OFUSG23765.1	5.3646	1.254936	0.412815	2.641137	0.008263	0.039382
Owenia_fusiformis	OFUSG23785.1	2872.412	1.532505	0.143879	10.65052	1.73E-26	1.64E-23
Owenia_fusiformis	OFUSG23800.1	249.377	1.292349	0.267457	4.817388	1.45E-06	3.39E-05
Owenia_fusiformis	OFUSG23872.1	113.3212	1.027969	0.308351	3.332448	0.000861	0.006755
Owenia_fusiformis	OFUSG23873.1	46.86077	1.329795	0.415158	3.083462	0.002046	0.013423
Owenia_fusiformis	OFUSG23903.1	479.8902	1.029795	0.322124	3.215354	0.001303	0.009352
Owenia_fusiformis	OFUSG23969.1	196.013	1.044842	0.217437	4.799616	1.59E-06	3.67E-05
Owenia_fusiformis	OFUSG24016.1	53.10016	1.43851	0.430935	3.460216	0.00054	0.004648
Owenia_fusiformis	OFUSG24065.1	57.63857	2.346801	0.435839	4.761905	1.92E-06	4.33E-05
Owenia_fusiformis	OFUSG24066.1	53.03735	1.27617	0.352753	3.592651	0.000327	0.003135
Owenia_fusiformis	OFUSG24071.1	240.2736	2.475416	0.328688	7.346652	2.03E-13	3.13E-11
Owenia_fusiformis	OFUSG24095.1	20.64527	1.486142	0.445756	3.326889	0.000878	0.006851
Owenia_fusiformis	OFUSG24151.1	22.88335	1.864834	0.433222	4.234177	2.29E-05	0.000357
Owenia_fusiformis	OFUSG24162.1	29.57195	1.517638	0.398345	3.641415	0.000271	0.002694
Owenia_fusiformis	OFUSG24163.1	216.3909	3.282218	0.416072	7.220642	5.17E-13	7.24E-11
Owenia_fusiformis	OFUSG24168.1	92.12976	1.200474	0.376988	3.200129	0.001374	0.009769
Owenia_fusiformis	OFUSG24186.1	94.68676	1.116089	0.319713	3.45187	0.000557	0.004756
Owenia_fusiformis	OFUSG24189.1	216.3231	2.670759	0.255349	10.3716	3.34E-25	2.94E-22
Owenia_fusiformis	OFUSG24190.2	62.58169	1.718614	0.333573	5.049158	4.44E-07	1.22E-05
Owenia_fusiformis	OFUSG24202.1	611.7035	1.263598	0.228411	5.535383	3.11E-08	1.24E-06
Owenia_fusiformis	OFUSG24209.1	288.3724	1.321849	0.298324	4.419827	9.88E-06	0.000176
Owenia_fusiformis	OFUSG24248.1	855.7238	1.207411	0.297417	4.069173	4.72E-05	0.000649
Owenia_fusiformis	OFUSG24251.1	42.21948	1.749507	0.433593	3.7823	0.000155	0.001718
Owenia_fusiformis	OFUSG24258.2	9.553466	1.211724	0.352889	3.021395	0.002516	0.015715
Owenia_fusiformis	OFUSG24285.3	340.8101	1.652545	0.299562	5.549196	2.87E-08	1.16E-06
Owenia_fusiformis	OFUSG24324.1	6.71781	1.35745	0.429489	2.993041	0.002762	0.016867
Owenia_fusiformis	OFUSG24366.1	19.16034	1.187251	0.441034	2.630667	0.008522	0.040304
Owenia_fusiformis	OFUSG24445.4	916.5104	1.432657	0.310458	4.625568	3.74E-06	7.65E-05
Owenia_fusiformis	OFUSG24633.1	30.62067	1.235286	0.425371	2.982241	0.002861	0.017333
Owenia_fusiformis	OFUSG24637.2	58.26732	2.997159	0.444023	6.173827	6.67E-10	4.24E-08
Owenia_fusiformis	OFUSG24711.1	70.32693	1.245218	0.409982	2.974711	0.002933	0.017663

Owenia_fusiformis	OFUSG24717.1	120.5912	1.320963	0.345122	3.786281	0.000153	0.001696
Owenia_fusiformis	OFUSG24734.1	15.43224	2.087069	0.448481	4.09791	4.17E-05	0.000587
Owenia_fusiformis	OFUSG24738.2	42.34516	2.067221	0.398745	4.988724	6.08E-07	1.58E-05
Owenia_fusiformis	OFUSG24752.1	31.59195	1.085265	0.420704	4.37524	1.21E-05	0.000208
Owenia_fusiformis	OFUSG24760.1	1076.518	1.067833	0.162007	6.590979	4.37E-11	3.72E-09
Owenia_fusiformis	OFUSG24798.1	18.87028	1.591774	0.44711	3.056499	0.002239	0.014381
Owenia_fusiformis	OFUSG24809.1	9.541439	1.222132	0.421224	2.585593	0.009721	0.044543
Owenia_fusiformis	OFUSG24830.1	55.05771	2.105676	0.41871	4.894449	9.86E-07	2.41E-05
Owenia_fusiformis	OFUSG24834.1	377.9148	1.437097	0.220911	6.502801	7.88E-11	6.22E-09
Owenia_fusiformis	OFUSG24902.1	14.89369	1.005229	0.385016	3.68068	0.000233	0.002393
Owenia_fusiformis	OFUSG24950.1	49.09773	1.207972	0.354836	3.41441	0.000639	0.005313
Owenia_fusiformis	OFUSG24974.1	167.8614	1.008733	0.354469	2.858183	0.004261	0.023707
Owenia_fusiformis	OFUSG25003.1	23.48007	1.6104	0.447327	3.596293	0.000323	0.003104
Owenia_fusiformis	OFUSG25088.1	372.3185	1.165611	0.245237	4.757842	1.96E-06	4.40E-05
Owenia_fusiformis	OFUSG25206.1	216.1998	1.076943	0.334394	3.223349	0.001267	0.009164
Owenia_fusiformis	OFUSG25207.3	198.0726	2.356524	0.400488	5.886955	3.93E-09	2.01E-07
Owenia_fusiformis	OFUSG25261.1	279.4642	1.140022	0.303432	3.760603	0.00017	0.001844
Owenia_fusiformis	OFUSG25274.1	29.82672	1.359837	0.414016	3.301346	0.000962	0.007346
Owenia_fusiformis	OFUSG25276.1	63.34007	1.025177	0.381673	2.571756	0.010118	0.045924
Owenia_fusiformis	OFUSG25330.1	22.22757	1.858411	0.420818	4.171209	3.03E-05	0.000451
Owenia_fusiformis	OFUSG25334.1	21.29085	1.349906	0.439413	3.076748	0.002093	0.01366
Owenia_fusiformis	OFUSG25339.1	257.6239	1.116147	0.255976	4.355691	1.33E-05	0.000226
Owenia_fusiformis	OFUSG25344.2	719.7851	1.068383	0.254927	4.202253	2.64E-05	0.0004
Owenia_fusiformis	OFUSG25368.1	8.407943	1.35665	0.446104	3.004116	0.002664	0.016403
Owenia_fusiformis	OFUSG25383.1	717.1278	1.202599	0.252368	4.769093	1.85E-06	4.19E-05
Owenia_fusiformis	OFUSG25520.1	212.3956	1.059055	0.245674	4.314473	1.60E-05	0.000263
Owenia_fusiformis	OFUSG25524.1	92.12633	1.583496	0.41903	3.770745	0.000163	0.001781
Owenia_fusiformis	OFUSG25559.1	1162.148	1.097258	0.153395	7.153077	8.49E-13	1.14E-10
Owenia_fusiformis	OFUSG25562.2	10.17411	1.374402	0.341668	3.049837	0.00229	0.014627
Owenia_fusiformis	OFUSG25572.1	2136.598	1.074426	0.201281	5.336493	9.48E-08	3.25E-06
Owenia_fusiformis	OFUSG25615.1	32.58494	1.068599	0.406655	2.585347	0.009728	0.044547
Owenia_fusiformis	OFUSG25637.1	573.2306	1.015817	0.220192	4.59916	4.24E-06	8.57E-05
Owenia_fusiformis	OFUSG25662.1	5.978454	2.061067	0.396121	4.068725	4.73E-05	0.00065
Owenia_fusiformis	OFUSG25688.2	123.6939	1.195853	0.290169	4.125393	3.70E-05	0.000531
Owenia_fusiformis	OFUSG25781.1	47.53256	1.388101	0.386551	3.522277	0.000428	0.003855
Owenia_fusiformis	OFUSG25799.1	171.5502	1.092257	0.28182	3.87373	0.000107	0.001268
Owenia_fusiformis	OFUSG25810.1	105.6619	1.417923	0.299197	4.7033	2.56E-06	5.53E-05
Owenia_fusiformis	OFUSG25806.2	36.8434	1.678447	0.414018	4.050681	5.11E-05	0.000691
Owenia_fusiformis	OFUSG25851.1	56.35473	2.56131	0.407093	6.261535	3.81E-10	2.57E-08
Owenia_fusiformis	OFUSG25881.1	699.0854	1.152018	0.292778	3.934167	8.35E-05	0.001036
Owenia_fusiformis	OFUSG25938.1	10.03659	1.134269	0.411423	2.794031	0.005206	0.027635
Owenia_fusiformis	OFUSG25951.1	61.88826	1.429495	0.401633	3.468987	0.000522	0.004527
Owenia_fusiformis	OFUSG26029.1	40.28112	1.250384	0.382003	3.293649	0.000989	0.007505
Owenia_fusiformis	OFUSG26048.1	8.412815	1.723095	0.389234	3.345428	0.000822	0.00651
Owenia_fusiformis	OFUSG26116.1	36.17594	1.105299	0.446211	2.582614	0.009806	0.044843
Owenia_fusiformis	OFUSG26149.1	29.3996	1.299747	0.426761	3.075703	0.0021	0.013693
Owenia_fusiformis	OFUSG26148.3	10.73053	1.024314	0.420664	3.540555	0.000399	0.003661
Owenia_fusiformis	OFUSG26179.1	70.70921	1.887324	0.360673	5.143967	2.69E-07	7.99E-06
Owenia_fusiformis	OFUSG26180.1	176.6501	1.206448	0.327893	3.729824	0.000192	0.002035
Owenia_fusiformis	OFUSG26210.1	5.825049	1.645083	0.406641	3.143928	0.001667	0.01149
Owenia_fusiformis	OFUSG26219.1	140.0325	1.699817	0.294838	5.740181	9.46E-09	4.37E-07
Owenia_fusiformis	OFUSG26225.1	34.16389	2.163779	0.447457	5.06656	4.05E-07	1.12E-05
Owenia_fusiformis	OFUSG26241.1	5.728658	1.060407	0.42713	2.661424	0.007781	0.03761
Owenia_fusiformis	OFUSG26258.1	213.4473	1.505062	0.313478	4.791774	1.65E-06	3.80E-05
Owenia_fusiformis	OFUSG26260.1	39.63431	2.961603	0.40173	6.954501	3.54E-12	4.03E-10
Owenia_fusiformis	OFUSG26268.1	1252.839	1.18388	0.30276	3.867326	0.00011	0.001295
Owenia_fusiformis	OFUSG26271.1	29.02265	1.483076	0.420088	3.488053	0.000487	0.004281
Owenia_fusiformis	OFUSG26273.1	46.06025	1.276297	0.384599	3.276146	0.001052	0.007882
Owenia_fusiformis	OFUSG26341.1	28.77421	1.559502	0.437528	3.349811	0.000809	0.006422
Owenia_fusiformis	OFUSG26368.1	36.88466	1.237405	0.414494	2.966846	0.003009	0.018077
Owenia_fusiformis	OFUSG26369.1	986.1458	1.113129	0.175646	6.33727	2.34E-10	1.62E-08
Owenia_fusiformis	OFUSG26421.1	99.58293	1.059563	0.397467	2.775533	0.005511	0.028846
Owenia_fusiformis	OFUSG26426.1	48.42062	1.299193	0.382896	3.269302	0.001078	0.008046
Owenia_fusiformis	OFUSG26433.1	1170.625	1.163458	0.188416	6.17939	6.43E-10	4.12E-08
Owenia_fusiformis	OFUSG26447.1	136.0219	1.409911	0.35995	3.861056	0.000113	0.001323
Owenia_fusiformis	OFUSG26451.1	54.30187	3.182094	0.372675	8.095028	5.73E-16	1.57E-13
Owenia_fusiformis	OFUSG26469.1	344.7881	1.17014	0.258927	4.516564	6.29E-06	0.00012
Owenia_fusiformis	OFUSG26474.1	197.4504	1.403792	0.285258	4.899012	9.63E-07	2.36E-05
Owenia_fusiformis	OFUSG26516.1	48.93654	1.058808	0.432845	2.544244	0.010951	0.048797
Owenia_fusiformis	OFUSG26531.1	47.20728	1.206687	0.390046	3.139815	0.001691	0.01161
Owenia_fusiformis	OFUSG26559.1	58.02771	1.276605	0.361296	3.558073	0.000374	0.003482
Owenia_fusiformis	OFUSG26581.1	235.0932	1.248092	0.278762	4.466955	7.93E-06	0.000147
Owenia_fusiformis	OFUSG26582.1	364.6547	1.269175	0.236385	5.358017	8.41E-08	2.93E-06
Owenia_fusiformis	OFUSG26611.1	351.6353	1.645844	0.357049	4.654009	3.26E-06	6.78E-05
Owenia_fusiformis	OFUSG26634.1	27.41894	1.355909	0.441628	2.747109	0.006012	0.030828
Owenia_fusiformis	OFUSG26718.1	32.86763	1.081452	0.448126	2.611383	0.009018	0.041957
Owenia_fusiformis	OFUSG26737.1	73.38891	2.263249	0.346932	6.443514	1.17E-10	8.76E-09
Owenia_fusiformis	OFUSG26790.1	11.81591	1.008607	0.435145	2.835545	0.004575	0.025055
Owenia_fusiformis	OFUSG26826.1	196.6677	1.15841	0.328437	3.582967	0.00034	0.00323
Owenia_fusiformis	OFUSG26827.1	1768.088	1.008395	0.22886	4.392848	1.12E-05	0.000195

Owenia_fusiformis	OFUSG26839.1	72.43646	1.398672	0.339297	4.088189	4.35E-05	0.000608
Owenia_fusiformis	OFUSG26843.1	133.8982	1.700971	0.409397	4.40098	1.08E-05	0.00019
Owenia_fusiformis	OFUSG26845.2	89.57001	1.082317	0.299438	3.592178	0.000328	0.00314
Owenia_fusiformis	OFUSG26864.1	1364.158	1.362944	0.216272	6.36113	2.00E-10	1.43E-08
Owenia_fusiformis	OFUSG26899.1	146.7874	1.16561	0.448537	2.913416	0.003575	0.020672
Owenia_fusiformis	OFUSG26907.1	39.61627	1.659686	0.428886	3.637962	0.000275	0.002722
Owenia_fusiformis	OFUSG26917.1	34.06216	1.174294	0.435524	2.716526	0.006597	0.03317
Owenia_fusiformis	OFUSG26918.2	20.42897	1.134509	0.437551	2.944083	0.003239	0.019116
Owenia_fusiformis	OFUSG26929.1	872.4872	1.169303	0.198369	5.89307	3.79E-09	1.95E-07

Supplementary Table 37. Differentially downregulated genes in the blastula (6 hpf) of O. fusiformis after rActivin A treatment.

Species	Gene_ID	basal_mean	log2FoldChange	lfcSE	stat	pvalue	padj
Owenia_fusiformis	OFUSG00018.1	81.13933	-1.10478	0.418399	-2.70207	0.006891	0.034269
Owenia_fusiformis	OFUSG00051.1	151.0024	-1.12993	0.294487	-3.81081	0.000139	0.00156
Owenia_fusiformis	OFUSG00064.2	125.6503	-1.10564	0.309925	-3.56405	0.000365	0.003419
Owenia_fusiformis	OFUSG00070.1	41.67298	-1.08591	0.443175	-2.63225	0.008482	0.040156
Owenia_fusiformis	OFUSG00092.1	86.47888	-1.15723	0.415568	-2.76461	0.005699	0.029644
Owenia_fusiformis	OFUSG00104.1	10566.97	-1.08681	0.151517	-7.17159	7.41E-13	1.01E-10
Owenia_fusiformis	OFUSG00130.1	57.9573	-1.14155	0.443544	-2.92372	0.003459	0.020122
Owenia_fusiformis	OFUSG00155.1	53.3739	-1.80609	0.446664	-4.08048	4.49E-05	0.000625
Owenia_fusiformis	OFUSG00164.1	477.1737	-1.61821	0.311517	-5.1533	2.56E-07	7.64E-06
Owenia_fusiformis	OFUSG00177.1	899.0233	-1.41358	0.163141	-8.66625	4.47E-18	1.59E-15
Owenia_fusiformis	OFUSG00214.1	1319.84	-1.16319	0.254265	-4.56181	5.07E-06	9.99E-05
Owenia_fusiformis	OFUSG00236.2	851.3463	-1.6015	0.213882	-7.52842	5.14E-14	8.91E-12
Owenia_fusiformis	OFUSG00279.2	33.56473	-1.44965	0.447817	-3.69874	0.000217	0.002256
Owenia_fusiformis	OFUSG00302.2	356.8204	-1.53967	0.331045	-4.79173	1.65E-06	3.80E-05
Owenia_fusiformis	OFUSG00344.3	61.80132	-1.35249	0.383398	-3.57458	0.000351	0.003316
Owenia_fusiformis	OFUSG00350.1	47.42425	-1.90635	0.428701	-4.40326	1.07E-05	0.000188
Owenia_fusiformis	OFUSG00377.1	520.6122	-1.15599	0.318018	-3.68987	0.000224	0.002319
Owenia_fusiformis	OFUSG00378.1	605.0682	-1.40078	0.362941	-3.79996	0.000145	0.001621
Owenia_fusiformis	OFUSG00394.1	1525.024	-1.81207	0.251224	-7.20382	5.85E-13	8.06E-11
Owenia_fusiformis	OFUSG00427.1	32.46915	-1.68382	0.446169	-3.87824	0.000105	0.00125
Owenia_fusiformis	OFUSG00455.1	275.049	-1.0538	0.241137	-4.38065	1.18E-05	0.000204
Owenia_fusiformis	OFUSG00486.1	7048.996	-1.48707	0.391556	-3.78479	0.000154	0.001703
Owenia_fusiformis	OFUSG00487.1	40.29123	-2.25971	0.431989	-5.01955	5.18E-07	1.38E-05
Owenia_fusiformis	OFUSG00494.1	23.06436	-1.36154	0.439327	-3.30502	0.00095	0.007281
Owenia_fusiformis	OFUSG00496.1	10.70165	-1.14962	0.430931	-2.78067	0.005425	0.028497
Owenia_fusiformis	OFUSG00497.1	181.555	-1.28598	0.337917	-3.77399	0.000161	0.001763
Owenia_fusiformis	OFUSG00498.3	70.22688	-1.71897	0.441363	-4.16679	3.09E-05	0.000459
Owenia_fusiformis	OFUSG00548.1	33.67241	-1.22468	0.414671	-2.7214	0.006501	0.032859
Owenia_fusiformis	OFUSG00552.1	73.56321	-1.0534	0.375723	-2.89103	0.00384	0.021864
Owenia_fusiformis	OFUSG00575.1	1392.446	-1.1533	0.298654	-3.82899	0.000129	0.001471
Owenia_fusiformis	OFUSG00610.1	154.2827	-1.50955	0.372085	-3.99304	6.52E-05	0.000844
Owenia_fusiformis	OFUSG00631.1	89.92808	-1.0595	0.442486	-2.70469	0.006837	0.034062
Owenia_fusiformis	OFUSG00652.3	509.493	-1.12234	0.339881	-3.31913	0.000903	0.006998
Owenia_fusiformis	OFUSG00656.2	363.9766	-1.18065	0.384729	-3.16048	0.001575	0.010967
Owenia_fusiformis	OFUSG00668.1	367.4451	-2.10972	0.348249	-6.20127	5.60E-10	3.66E-08
Owenia_fusiformis	OFUSG00669.1	414.7202	-2.16777	0.36189	-5.98209	2.20E-09	1.22E-07
Owenia_fusiformis	OFUSG00710.2	80.16064	-1.1078	0.442238	-2.86732	0.00414	0.023201
Owenia_fusiformis	OFUSG00716.3	82.21486	-1.51325	0.441638	-5.08915	3.60E-07	1.01E-05
Owenia_fusiformis	OFUSG00792.1	191.0296	-2.22885	0.280742	-7.82887	4.92E-15	1.11E-12
Owenia_fusiformis	OFUSG00842.1	698.295	-1.62062	0.319752	-5.03685	4.73E-07	1.28E-05
Owenia_fusiformis	OFUSG00856.1	576.8085	-1.96211	0.300052	-6.62558	3.46E-11	3.08E-09
Owenia_fusiformis	OFUSG00861.1	27.96824	-2.06952	0.444958	-4.39637	1.10E-05	0.000193
Owenia_fusiformis	OFUSG00870.1	42.38692	-1.15405	0.43058	-2.58148	0.009838	0.044974
Owenia_fusiformis	OFUSG00893.1	37.43623	-1.29722	0.444858	-2.98851	0.002803	0.017051
Owenia_fusiformis	OFUSG00895.2	71.03654	-1.41364	0.439146	-2.99589	0.002736	0.016756
Owenia_fusiformis	OFUSG00918.1	13.77973	-1.15181	0.446188	-2.56299	0.010377	0.046858
Owenia_fusiformis	OFUSG00935.1	85.02527	-2.30062	0.356177	-6.27217	3.56E-10	2.40E-08
Owenia_fusiformis	OFUSG01023.1	1122.701	-1.12531	0.152701	-7.3877	1.49E-13	2.33E-11
Owenia_fusiformis	OFUSG01034.2	78.79707	-1.26488	0.305844	-4.09143	4.29E-05	0.000601
Owenia_fusiformis	OFUSG01100.1	133.1457	-1.24043	0.289167	-4.26625	1.99E-05	0.000316
Owenia_fusiformis	OFUSG01116.1	71.44294	-1.22114	0.431181	-2.99866	0.002712	0.016649
Owenia_fusiformis	OFUSG01117.5	35.06192	-1.05948	0.375794	-3.92845	8.55E-05	0.001054
Owenia_fusiformis	OFUSG01136.1	350.3754	-1.45528	0.320342	-4.52547	6.03E-06	0.000116
Owenia_fusiformis	OFUSG01224.1	687.4599	-1.15246	0.127428	-9.03308	1.67E-19	7.21E-17
Owenia_fusiformis	OFUSG01228.1	25.82885	-2.06031	0.43847	-4.13341	3.57E-05	0.000515
Owenia_fusiformis	OFUSG01253.1	838.543	-1.23263	0.173381	-7.10306	1.22E-12	1.57E-10
Owenia_fusiformis	OFUSG01309.1	382.8477	-2.59225	0.259069	-9.96137	2.25E-23	1.79E-20
Owenia_fusiformis	OFUSG01339.1	107.282	-1.23092	0.413169	-2.93222	0.003365	0.019743

Owenia_fusiformis	OFUSG01342.2	128.7273	-1.40811	0.265694	-5.24434	1.57E-07	5.02E-06
Owenia_fusiformis	OFUSG01360.1	274.0443	-1.01091	0.307281	-3.27859	0.001043	0.007825
Owenia_fusiformis	OFUSG01396.1	37.72189	-1.46267	0.416188	-3.61787	0.000297	0.002907
Owenia_fusiformis	OFUSG01407.1	85.64179	-1.78172	0.343426	-5.19309	2.07E-07	6.35E-06
Owenia_fusiformis	OFUSG01422.1	7.400019	-1.04383	0.411559	-2.55555	0.010602	0.047585
Owenia_fusiformis	OFUSG01449.1	469.1406	-1.25403	0.208729	-5.99841	1.99E-09	1.11E-07
Owenia_fusiformis	OFUSG01450.1	23.33161	-2.00729	0.435752	-3.85884	0.000114	0.001332
Owenia_fusiformis	OFUSG01471.1	1713.506	-1.23337	0.263453	-4.68371	2.82E-06	5.98E-05
Owenia_fusiformis	OFUSG01482.1	224.6302	-1.4319	0.339917	-4.2585	2.06E-05	0.000325
Owenia_fusiformis	OFUSG01497.1	121.4632	-2.19405	0.389747	-5.73941	9.50E-09	4.38E-07
Owenia_fusiformis	OFUSG01551.2	543.307	-1.58439	0.309394	-5.1329	2.85E-07	8.35E-06
Owenia_fusiformis	OFUSG01553.2	635.5281	-1.34521	0.356709	-3.67248	0.00024	0.002456
Owenia_fusiformis	OFUSG01567.2	30.424	-1.44473	0.447851	-3.51339	0.000442	0.00396
Owenia_fusiformis	OFUSG01601.1	81.24756	-1.39803	0.428552	-3.73313	0.000189	0.002019
Owenia_fusiformis	OFUSG01614.1	1119.533	-1.17757	0.229116	-5.1304	2.89E-07	8.43E-06
Owenia_fusiformis	OFUSG01617.1	26.28859	-1.29061	0.447947	-3.04182	0.002352	0.014937
Owenia_fusiformis	OFUSG01680.1	61.17592	-1.25264	0.406278	-3.2621	0.001106	0.008198
Owenia_fusiformis	OFUSG01689.1	10.13099	-1.092	0.438344	-3.21612	0.001299	0.009337
Owenia_fusiformis	OFUSG01743.1	309.9064	-1.17974	0.337253	-3.49779	0.000469	0.004153
Owenia_fusiformis	OFUSG01750.1	1888.963	-2.64324	0.176832	-14.9333	2.00E-50	7.04E-47
Owenia_fusiformis	OFUSG01758.2	29.3733	-1.49301	0.447139	-3.51749	0.000436	0.003911
Owenia_fusiformis	OFUSG01771.1	277.2109	-1.76186	0.447149	-3.64478	0.000268	0.002666
Owenia_fusiformis	OFUSG01772.1	24.00703	-1.64761	0.441582	-3.60135	0.000317	0.003057
Owenia_fusiformis	OFUSG01783.1	56.39788	-1.20323	0.429676	-2.76164	0.005751	0.029824
Owenia_fusiformis	OFUSG01815.1	811.4015	-1.06323	0.170495	-6.22573	4.79E-10	3.17E-08
Owenia_fusiformis	OFUSG01838.1	1635.02	-1.08965	0.33904	-3.17972	0.001474	0.010364
Owenia_fusiformis	OFUSG01904.1	31.18618	-1.9816	0.445242	-4.16396	3.13E-05	0.000463
Owenia_fusiformis	OFUSG01931.2	1097.023	-1.08614	0.179892	-6.04117	1.53E-09	8.76E-08
Owenia_fusiformis	OFUSG01935.1	23.67711	-1.54223	0.447623	-3.48421	0.000494	0.004335
Owenia_fusiformis	OFUSG01941.1	19.23327	-1.2009	0.445203	-2.62432	0.008682	0.040867
Owenia_fusiformis	OFUSG01955.1	56.12148	-1.23892	0.399065	-2.84137	0.004492	0.024715
Owenia_fusiformis	OFUSG01956.2	42.44975	-1.88211	0.448195	-3.48743	0.000488	0.004289
Owenia_fusiformis	OFUSG01978.1	19.55203	-1.01738	0.447611	-2.61985	0.008797	0.04121
Owenia_fusiformis	OFUSG02033.1	129.1496	-3.07384	0.388976	-7.64222	2.14E-14	4.08E-12
Owenia_fusiformis	OFUSG02036.1	7.901514	-1.34795	0.409835	-3.28865	0.001007	0.007614
Owenia_fusiformis	OFUSG02050.1	71.99797	-2.687	0.35996	-6.98835	2.78E-12	3.25E-10
Owenia_fusiformis	OFUSG02051.1	15.28091	-1.1356	0.442712	-3.52002	0.000432	0.003884
Owenia_fusiformis	OFUSG02052.1	304.3564	-1.45879	0.236267	-6.1473	7.88E-10	4.90E-08
Owenia_fusiformis	OFUSG02054.1	52.09049	-1.57945	0.39789	-3.83423	0.000126	0.001446
Owenia_fusiformis	OFUSG02151.1	568.0829	-1.56584	0.32357	-4.78921	1.67E-06	3.83E-05
Owenia_fusiformis	OFUSG02152.1	22.96297	-2.31504	0.445096	-4.43398	9.25E-06	0.000167
Owenia_fusiformis	OFUSG02164.1	86.62015	-1.16674	0.317055	-3.65379	0.000258	0.002589
Owenia_fusiformis	OFUSG02176.2	935.9615	-1.08164	0.145026	-7.46952	8.05E-14	1.34E-11
Owenia_fusiformis	OFUSG02185.1	7.666274	-1.4091	0.371896	-3.42154	0.000623	0.005194
Owenia_fusiformis	OFUSG02187.1	11.07799	-1.34292	0.429305	-3.5715	0.000355	0.003345
Owenia_fusiformis	OFUSG02201.1	10.07159	-1.94589	0.429119	-3.94245	8.07E-05	0.001007
Owenia_fusiformis	OFUSG02202.1	80.45037	-1.84173	0.402177	-4.50674	6.58E-06	0.000125
Owenia_fusiformis	OFUSG02209.1	1186.052	-1.31859	0.303977	-4.33334	1.47E-05	0.000245
Owenia_fusiformis	OFUSG02232.1	4797.408	-1.84804	0.273414	-6.74735	1.51E-11	1.44E-09
Owenia_fusiformis	OFUSG02271.1	17.16956	-1.17795	0.436888	-3.66995	0.000243	0.002473
Owenia_fusiformis	OFUSG02277.1	216.2386	-1.10754	0.287619	-3.83977	0.000123	0.001418
Owenia_fusiformis	OFUSG02312.2	421.9066	-1.28423	0.315348	-4.04059	5.33E-05	0.000716
Owenia_fusiformis	OFUSG02319.1	7.716477	-1.18954	0.40928	-3.21138	0.001321	0.009468
Owenia_fusiformis	OFUSG02381.1	67.09817	-1.25881	0.377087	-3.24045	0.001193	0.008726
Owenia_fusiformis	OFUSG02428.1	21.10403	-1.04178	0.447836	-2.56751	0.010243	0.046379
Owenia_fusiformis	OFUSG02429.1	332.9902	-1.2767	0.350239	-3.76824	0.000164	0.001796
Owenia_fusiformis	OFUSG02429.2	226.7629	-1.12049	0.409334	-2.94892	0.003189	0.018928
Owenia_fusiformis	OFUSG02429.3	138.0893	-1.38532	0.446091	-3.79178	0.00015	0.001664
Owenia_fusiformis	OFUSG02439.1	321.8976	-1.84134	0.447818	-4.1757	2.97E-05	0.000443
Owenia_fusiformis	OFUSG02440.2	182.6611	-1.35465	0.389041	-3.70548	0.000211	0.002205
Owenia_fusiformis	OFUSG02441.1	185.0635	-1.18614	0.334803	-3.51967	0.000432	0.003888
Owenia_fusiformis	OFUSG02484.1	407.6475	-1.22135	0.326371	-3.75316	0.000175	0.00189
Owenia_fusiformis	OFUSG02485.1	7196.813	-1.03811	0.127022	-8.1719	3.04E-16	8.69E-14
Owenia_fusiformis	OFUSG02525.2	19.97736	-1.49291	0.438941	-3.45988	0.00054	0.004652
Owenia_fusiformis	OFUSG02543.3	980.4605	-1.19951	0.285993	-4.16283	3.14E-05	0.000464
Owenia_fusiformis	OFUSG02570.2	70.09133	-1.49054	0.352151	-4.16151	3.16E-05	0.000466
Owenia_fusiformis	OFUSG02576.1	44.45283	-1.01224	0.439788	-3.54297	0.000396	0.003637
Owenia_fusiformis	OFUSG02594.1	31.86728	-1.00827	0.442232	-2.56136	0.010426	0.046995
Owenia_fusiformis	OFUSG02595.1	44.91657	-1.53661	0.436832	-3.72663	0.000194	0.002057
Owenia_fusiformis	OFUSG02634.3	193.3643	-1.29379	0.447196	-3.90883	9.27E-05	0.001123
Owenia_fusiformis	OFUSG02675.2	205.4606	-1.22945	0.448514	-2.73246	0.006286	0.032013
Owenia_fusiformis	OFUSG02679.2	3916.062	-1.09737	0.204157	-5.39086	7.01E-08	2.52E-06
Owenia_fusiformis	OFUSG02686.1	9.692612	-1.21065	0.427059	-2.59112	0.009566	0.043979
Owenia_fusiformis	OFUSG02703.1	37.6146	-1.47019	0.43805	-4.48503	7.29E-06	0.000137
Owenia_fusiformis	OFUSG02723.1	4301.309	-1.69943	0.172492	-9.8585	6.30E-23	4.56E-20
Owenia_fusiformis	OFUSG02760.1	8.280495	-1.19682	0.42669	-2.67479	0.007478	0.036545
Owenia_fusiformis	OFUSG02786.1	20.89772	-1.2083	0.446607	-2.90135	0.003716	0.02129
Owenia_fusiformis	OFUSG02805.2	244.1303	-1.08058	0.320547	-3.33127	0.000864	0.006774
Owenia_fusiformis	OFUSG02818.1	1036.669	-1.24637	0.360095	-3.40948	0.000651	0.005394
Owenia_fusiformis	OFUSG02906.1	428.2503	-1.43327	0.363948	-3.91203	9.15E-05	0.001112

Owenia_fusiformis	OFUSG02910.1	829.2603	-1.06962	0.183908	-5.83412	5.41E-09	2.66E-07
Owenia_fusiformis	OFUSG02983.3	240.0335	-1.60425	0.437783	-3.80302	0.000143	0.001603
Owenia_fusiformis	OFUSG03018.1	113.5934	-1.33277	0.443712	-3.08582	0.00203	0.013342
Owenia_fusiformis	OFUSG03071.1	3106.283	-1.00815	0.218762	-4.60491	4.13E-06	8.36E-05
Owenia_fusiformis	OFUSG03102.1	10.37535	-1.27567	0.40013	-3.39216	0.000693	0.005672
Owenia_fusiformis	OFUSG03129.1	151.5245	-1.09924	0.448579	-2.9064	0.003656	0.021028
Owenia_fusiformis	OFUSG03148.1	56.64768	-1.45342	0.406914	-3.68721	0.000227	0.002339
Owenia_fusiformis	OFUSG03175.1	1110.631	-2.20378	0.272869	-8.0801	6.47E-16	1.73E-13
Owenia_fusiformis	OFUSG03182.1	115.6323	-1.16247	0.376247	-3.29083	0.000999	0.007567
Owenia_fusiformis	OFUSG03185.1	233.5462	-2.03434	0.2679	-7.57618	3.56E-14	6.26E-12
Owenia_fusiformis	OFUSG03200.1	11.88047	-1.11796	0.437246	-3.21673	0.001297	0.009322
Owenia_fusiformis	OFUSG03210.1	23.02493	-2.03735	0.446542	-3.96521	7.33E-05	0.00093
Owenia_fusiformis	OFUSG03224.1	59.7401	-1.17391	0.387624	-3.09276	0.001983	0.013092
Owenia_fusiformis	OFUSG03239.1	72.53342	-1.3543	0.433214	-3.17848	0.001481	0.010391
Owenia_fusiformis	OFUSG03321.1	348.9586	-1.771	0.351575	-5.35946	8.35E-08	2.91E-06
Owenia_fusiformis	OFUSG03326.1	30.03604	-1.39779	0.421725	-3.42902	0.000606	0.005084
Owenia_fusiformis	OFUSG03383.1	17.55863	-1.57214	0.447211	-3.31105	0.000929	0.007155
Owenia_fusiformis	OFUSG03393.1	21.27714	-1.72708	0.441465	-3.66389	0.000248	0.002513
Owenia_fusiformis	OFUSG03400.1	59.22477	-1.21005	0.379923	-3.11332	0.00185	0.012421
Owenia_fusiformis	OFUSG03408.1	16.1393	-1.61557	0.407716	-3.8571	0.000115	0.001339
Owenia_fusiformis	OFUSG03420.1	67.84564	-1.01763	0.414735	-2.92528	0.003441	0.020055
Owenia_fusiformis	OFUSG03459.1	240.5873	-1.18843	0.219099	-5.38886	7.09E-08	2.54E-06
Owenia_fusiformis	OFUSG03478.1	72.02656	-1.09474	0.446331	-3.15871	0.001585	0.011018
Owenia_fusiformis	OFUSG03480.1	9.170561	-1.06376	0.421068	-2.62126	0.00876	0.041086
Owenia_fusiformis	OFUSG03485.1	46.86158	-1.43566	0.448387	-3.86057	0.000113	0.001325
Owenia_fusiformis	OFUSG03503.1	12.82583	-1.516	0.441899	-3.19003	0.001423	0.010053
Owenia_fusiformis	OFUSG03504.1	16.76112	-1.2056	0.438359	-3.00688	0.002639	0.016298
Owenia_fusiformis	OFUSG03505.1	500.3474	-1.05603	0.312399	-3.43809	0.000586	0.004943
Owenia_fusiformis	OFUSG03509.1	1600.023	-1.14555	0.168957	-6.77783	1.22E-11	1.21E-09
Owenia_fusiformis	OFUSG03530.1	33.60557	-1.72451	0.425412	-3.89283	9.91E-05	0.001188
Owenia_fusiformis	OFUSG03540.1	20.08029	-1.48881	0.437449	-3.42066	0.000625	0.005205
Owenia_fusiformis	OFUSG03563.1	35.10758	-1.58066	0.435647	-3.49483	0.000474	0.004195
Owenia_fusiformis	OFUSG03565.1	86.06052	-1.12842	0.361565	-3.05655	0.002239	0.014381
Owenia_fusiformis	OFUSG03608.1	501.048	-1.5443	0.278416	-5.52456	3.30E-08	1.31E-06
Owenia_fusiformis	OFUSG03668.1	163.4076	-1.19468	0.296965	-3.97351	7.08E-05	0.000905
Owenia_fusiformis	OFUSG03699.1	13.74231	-1.65458	0.429243	-3.91136	9.18E-05	0.001113
Owenia_fusiformis	OFUSG03734.1	46.65615	-1.0902	0.420143	-2.68541	0.007244	0.035664
Owenia_fusiformis	OFUSG03806.2	26.58999	-2.08607	0.446273	-4.64464	3.41E-06	7.04E-05
Owenia_fusiformis	OFUSG03822.1	74.01642	-1.23433	0.349263	-3.47357	0.000514	0.004472
Owenia_fusiformis	OFUSG03831.1	25.25694	-1.39834	0.448235	-3.14935	0.001636	0.01132
Owenia_fusiformis	OFUSG03926.1	275.7831	-1.5671	0.299718	-5.22904	1.70E-07	5.41E-06
Owenia_fusiformis	OFUSG03940.1	437.4404	-1.10614	0.264331	-4.17842	2.94E-05	0.000439
Owenia_fusiformis	OFUSG03942.2	117.254	-1.5878	0.395277	-4.09032	4.31E-05	0.000603
Owenia_fusiformis	OFUSG03979.1	77.53904	-1.24769	0.388662	-3.43944	0.000583	0.004931
Owenia_fusiformis	OFUSG03997.2	98.72977	-1.16464	0.392094	-2.99635	0.002732	0.016738
Owenia_fusiformis	OFUSG04001.1	432.3649	-1.13795	0.275009	-4.10671	4.01E-05	0.000568
Owenia_fusiformis	OFUSG04004.1	384.8986	-1.03679	0.192537	-5.36429	8.13E-08	2.86E-06
Owenia_fusiformis	OFUSG04033.2	59.37401	-1.59822	0.448045	-4.70245	2.57E-06	5.54E-05
Owenia_fusiformis	OFUSG04040.1	46.5113	-1.83101	0.42997	-4.29673	1.73E-05	0.000282
Owenia_fusiformis	OFUSG04050.2	199.58	-1.03193	0.266643	-3.86969	0.000109	0.001285
Owenia_fusiformis	OFUSG04071.5	462.014	-1.34346	0.287191	-4.65191	3.29E-06	6.83E-05
Owenia_fusiformis	OFUSG04095.1	27.54578	-1.37138	0.447953	-3.08324	0.002048	0.013423
Owenia_fusiformis	OFUSG04111.3	31.48	-1.93176	0.441691	-4.49989	6.80E-06	0.000129
Owenia_fusiformis	OFUSG04121.1	38.87232	-1.34874	0.438119	-3.04615	0.002318	0.014766
Owenia_fusiformis	OFUSG04145.1	96.51472	-1.06322	0.284826	-3.76159	0.000169	0.001838
Owenia_fusiformis	OFUSG04149.1	540.0967	-1.09277	0.200541	-5.4477	5.10E-08	1.91E-06
Owenia_fusiformis	OFUSG04155.1	354.2859	-1.01002	0.234058	-4.3051	1.67E-05	0.000273
Owenia_fusiformis	OFUSG04220.1	251.4992	-1.30427	0.408781	-3.19468	0.0014	0.009924
Owenia_fusiformis	OFUSG04232.2	310.7374	-1.60145	0.331769	-4.84102	1.29E-06	3.06E-05
Owenia_fusiformis	OFUSG04248.1	93.19759	-1.12752	0.422066	-2.8763	0.004024	0.022688
Owenia_fusiformis	OFUSG04279.1	21.6662	-1.62056	0.442524	-3.5505	0.000385	0.003567
Owenia_fusiformis	OFUSG04312.1	33.48169	-1.56933	0.420623	-3.66687	0.000246	0.002496
Owenia_fusiformis	OFUSG04314.1	135.5273	-1.17044	0.387845	-3.07632	0.002096	0.013675
Owenia_fusiformis	OFUSG04328.1	15.4619	-2.04526	0.433078	-3.42585	0.000613	0.005129
Owenia_fusiformis	OFUSG04343.1	251.2562	-1.22771	0.299648	-4.11843	3.81E-05	0.000546
Owenia_fusiformis	OFUSG04352.1	17.33356	-1.94803	0.438432	-4.2658	1.99E-05	0.000316
Owenia_fusiformis	OFUSG04378.1	829.2086	-2.72747	0.365829	-7.50087	6.34E-14	1.08E-11
Owenia_fusiformis	OFUSG04390.1	358.5522	-2.46607	0.313901	-7.83718	4.61E-15	1.05E-12
Owenia_fusiformis	OFUSG04401.1	49.55642	-1.27389	0.399594	-3.20444	0.001353	0.009643
Owenia_fusiformis	OFUSG04443.1	44.64561	-2.54141	0.418274	-5.58345	2.36E-08	9.71E-07
Owenia_fusiformis	OFUSG04443.2	42.37409	-1.78659	0.419318	-4.04889	5.15E-05	0.000696
Owenia_fusiformis	OFUSG04459.1	8.714305	-1.12321	0.431393	-2.56537	0.010307	0.046632
Owenia_fusiformis	OFUSG04463.1	43.80801	-3.34678	0.444229	-5.73317	9.86E-09	4.53E-07
Owenia_fusiformis	OFUSG04464.1	192.8212	-1.13938	0.393934	-2.79574	0.005178	0.027542
Owenia_fusiformis	OFUSG04490.1	1867.344	-1.04292	0.14432	-7.22884	4.87E-13	6.93E-11
Owenia_fusiformis	OFUSG04491.1	2049.579	-1.18572	0.208104	-5.69367	1.24E-08	5.59E-07
Owenia_fusiformis	OFUSG04500.1	21.79161	-1.4774	0.446573	-3.03449	0.002409	0.015223
Owenia_fusiformis	OFUSG04526.1	317.9627	-1.76773	0.298596	-5.931	3.01E-09	1.59E-07
Owenia_fusiformis	OFUSG04527.1	4816.739	-1.74945	0.133183	-13.1399	1.94E-39	4.79E-36
Owenia_fusiformis	OFUSG04533.1	14.03922	-1.20447	0.378354	-3.61263	0.000303	0.002956

Owenia_fusiformis	OFUSG04563.1	3201.764	-1.04647	0.185118	-5.64802	1.62E-08	7.02E-07
Owenia_fusiformis	OFUSG04580.1	125.9963	-1.38705	0.432736	-3.69576	0.000219	0.002279
Owenia_fusiformis	OFUSG04611.4	28.88237	-3.01156	0.445354	-5.32271	1.02E-07	3.45E-06
Owenia_fusiformis	OFUSG04625.1	114.4187	-1.22119	0.293689	-4.1349	3.55E-05	0.000513
Owenia_fusiformis	OFUSG04632.1	22.85814	-1.5271	0.447086	-3.00682	0.00264	0.016298
Owenia_fusiformis	OFUSG04646.2	75.46976	-1.35836	0.320057	-4.11422	3.88E-05	0.000553
Owenia_fusiformis	OFUSG04654.2	413.7074	-1.16216	0.438582	-4.07658	4.57E-05	0.000634
Owenia_fusiformis	OFUSG04757.1	386.7653	-3.08419	0.400372	-8.3268	8.31E-17	2.56E-14
Owenia_fusiformis	OFUSG04795.1	269.9426	-1.35265	0.243334	-5.57979	2.41E-08	9.88E-07
Owenia_fusiformis	OFUSG04821.1	85.36814	-1.50257	0.444092	-3.21115	0.001322	0.00947
Owenia_fusiformis	OFUSG04855.1	44.25654	-1.74805	0.42617	-4.36825	1.25E-05	0.000214
Owenia_fusiformis	OFUSG04856.1	43.4478	-1.80432	0.415426	-4.33232	1.48E-05	0.000246
Owenia_fusiformis	OFUSG04872.1	23.59003	-1.54985	0.447214	-3.61458	0.000301	0.002936
Owenia_fusiformis	OFUSG04908.1	31.33277	-1.41813	0.448198	-3.30768	0.000941	0.007231
Owenia_fusiformis	OFUSG04987.2	15.76693	-2.24542	0.443997	-4.45368	8.44E-06	0.000154
Owenia_fusiformis	OFUSG04995.1	3155.79	-1.0716	0.094406	-11.3506	7.36E-30	9.54E-27
Owenia_fusiformis	OFUSG05042.1	228.4885	-1.41616	0.330853	-4.33006	1.49E-05	0.000248
Owenia_fusiformis	OFUSG05075.1	47.48526	-1.06147	0.391287	-2.71221	0.006684	0.033502
Owenia_fusiformis	OFUSG05089.2	45.99846	-1.14844	0.435424	-2.70927	0.006743	0.033732
Owenia_fusiformis	OFUSG05093.1	36.17741	-1.36099	0.404935	-3.40127	0.000671	0.005525
Owenia_fusiformis	OFUSG05115.1	5428.057	-1.13805	0.247653	-4.58825	4.47E-06	8.96E-05
Owenia_fusiformis	OFUSG05118.1	282.8535	-1.08397	0.209777	-5.19273	2.07E-07	6.36E-06
Owenia_fusiformis	OFUSG05126.2	2089.618	-1.3064	0.229497	-5.68552	1.30E-08	5.82E-07
Owenia_fusiformis	OFUSG05150.1	573.5509	-1.8092	0.3692	-4.85288	1.22E-06	2.93E-05
Owenia_fusiformis	OFUSG05153.1	99.78939	-1.41653	0.398499	-3.64596	0.000266	0.002658
Owenia_fusiformis	OFUSG05157.1	79.22855	-1.15995	0.445294	-2.65175	0.008008	0.038366
Owenia_fusiformis	OFUSG05159.1	37.87318	-1.03882	0.418932	-2.65397	0.007955	0.038175
Owenia_fusiformis	OFUSG05193.1	50.21814	-1.3823	0.412108	-3.23167	0.001231	0.008954
Owenia_fusiformis	OFUSG05212.1	436.1816	-1.10974	0.21918	-5.06286	4.13E-07	1.14E-05
Owenia_fusiformis	OFUSG05219.1	171.5822	-1.58359	0.262201	-6.00814	1.88E-09	1.06E-07
Owenia_fusiformis	OFUSG05245.1	83.64698	-1.45595	0.423431	-3.38726	0.000706	0.005753
Owenia_fusiformis	OFUSG05271.1	296.1324	-1.28216	0.300438	-4.34917	1.37E-05	0.000231
Owenia_fusiformis	OFUSG05278.1	106.5157	-2.06393	0.373934	-5.43778	5.39E-08	2.01E-06
Owenia_fusiformis	OFUSG05306.1	470.5082	-1.08895	0.221929	-4.89489	9.84E-07	2.41E-05
Owenia_fusiformis	OFUSG05307.1	346.5357	-1.06665	0.44634	-2.59144	0.009558	0.043946
Owenia_fusiformis	OFUSG05327.1	44.86033	-1.073	0.412884	-2.57902	0.009908	0.045195
Owenia_fusiformis	OFUSG05342.1	51.98115	-1.43272	0.385096	-3.82995	0.000128	0.001468
Owenia_fusiformis	OFUSG05370.1	27.56537	-1.16761	0.443538	-2.80537	0.005026	0.026919
Owenia_fusiformis	OFUSG05381.1	556.2128	-1.10347	0.210259	-5.24571	1.56E-07	4.99E-06
Owenia_fusiformis	OFUSG05407.1	70.94256	-1.33476	0.447857	-3.51804	0.000435	0.003905
Owenia_fusiformis	OFUSG05412.2	114.7465	-1.41232	0.441435	-3.22687	0.001252	0.009076
Owenia_fusiformis	OFUSG05440.1	153.389	-1.01234	0.354607	-3.02345	0.002499	0.015649
Owenia_fusiformis	OFUSG05441.1	672.7723	-2.54094	0.166381	-15.1894	4.16E-52	2.05E-48
Owenia_fusiformis	OFUSG05461.2	795.7366	-1.08655	0.290459	-3.71921	0.0002	0.002108
Owenia_fusiformis	OFUSG05522.1	102.4062	-1.6097	0.327982	-4.90321	9.43E-07	2.32E-05
Owenia_fusiformis	OFUSG05526.1	92.78769	-1.12276	0.37418	-3.00995	0.002613	0.016168
Owenia_fusiformis	OFUSG05533.1	24.15146	-1.76096	0.439351	-3.73051	0.000191	0.002031
Owenia_fusiformis	OFUSG05558.1	12.42177	-1.15991	0.446953	-2.57837	0.009927	0.045263
Owenia_fusiformis	OFUSG05565.1	1685.958	-2.36374	0.317836	-7.46473	8.35E-14	1.38E-11
Owenia_fusiformis	OFUSG05604.1	493.6266	-1.01605	0.371076	-2.72845	0.006363	0.032351
Owenia_fusiformis	OFUSG05607.1	81.51118	-1.15626	0.298126	-3.85368	0.000116	0.001352
Owenia_fusiformis	OFUSG05623.1	47.52428	-1.5806	0.439191	-3.62921	0.000284	0.002806
Owenia_fusiformis	OFUSG05637.1	4094.914	-1.08399	0.157364	-6.90702	4.95E-12	5.44E-10
Owenia_fusiformis	OFUSG05637.4	1242.222	-2.22968	0.236647	-9.40092	5.41E-21	3.25E-18
Owenia_fusiformis	OFUSG05665.2	328.0743	-1.01535	0.290854	-3.49502	0.000474	0.004193
Owenia_fusiformis	OFUSG05700.1	559.2001	-1.0984	0.277753	-3.93975	8.16E-05	0.001015
Owenia_fusiformis	OFUSG05711.1	59.1032	-1.43905	0.40347	-3.54946	0.000386	0.003575
Owenia_fusiformis	OFUSG05732.1	39.0548	-1.14099	0.429984	-2.55445	0.010636	0.047698
Owenia_fusiformis	OFUSG05745.1	86.04593	-1.82933	0.390496	-4.72021	2.36E-06	5.14E-05
Owenia_fusiformis	OFUSG05747.1	397.3954	-1.61451	0.361564	-4.41779	9.97E-06	0.000177
Owenia_fusiformis	OFUSG05765.1	103.8614	-1.16748	0.375832	-3.22879	0.001243	0.009028
Owenia_fusiformis	OFUSG05797.1	9.297891	-1.16577	0.429468	-3.23442	0.001219	0.008887
Owenia_fusiformis	OFUSG05830.1	318.1583	-1.60767	0.42276	-3.69403	0.000221	0.00229
Owenia_fusiformis	OFUSG05831.1	82.74647	-2.65266	0.360697	-7.01949	2.23E-12	2.66E-10
Owenia_fusiformis	OFUSG05836.1	258.4122	-1.39963	0.446999	-2.87159	0.004084	0.022948
Owenia_fusiformis	OFUSG05840.1	322.706	-1.56137	0.362827	-4.31359	1.61E-05	0.000263
Owenia_fusiformis	OFUSG05860.1	393.9476	-1.14995	0.246779	-4.64713	3.37E-06	6.97E-05
Owenia_fusiformis	OFUSG05862.1	134.5084	-1.35246	0.34796	-3.93208	8.42E-05	0.001042
Owenia_fusiformis	OFUSG05864.1	25.84351	-2.41162	0.440257	-4.73443	2.20E-06	4.87E-05
Owenia_fusiformis	OFUSG05865.1	51.97476	-1.83131	0.428163	-3.86206	0.000112	0.00132
Owenia_fusiformis	OFUSG05902.1	17.68829	-1.40115	0.403772	-3.52376	0.000425	0.003844
Owenia_fusiformis	OFUSG05943.1	16.18752	-1.28623	0.44726	-2.95349	0.003142	0.018704
Owenia_fusiformis	OFUSG05962.2	36.46576	-1.17202	0.430075	-2.66353	0.007732	0.037427
Owenia_fusiformis	OFUSG05973.1	556.0683	-1.65181	0.182315	-9.04934	1.44E-19	6.44E-17
Owenia_fusiformis	OFUSG06020.3	1574.243	-1.61494	0.265456	-6.09919	1.07E-09	6.37E-08
Owenia_fusiformis	OFUSG06124.1	366.6455	-1.65355	0.244862	-6.75587	1.42E-11	1.38E-09
Owenia_fusiformis	OFUSG06133.1	97.15958	-1.29102	0.379004	-3.51321	0.000443	0.00396
Owenia_fusiformis	OFUSG06175.2	95.85774	-1.43583	0.448547	-3.67646	0.000236	0.002427
Owenia_fusiformis	OFUSG06181.3	26.58955	-1.173	0.445807	-3.82447	0.000131	0.001493
Owenia_fusiformis	OFUSG06195.1	181.0882	-1.37359	0.448058	-3.77763	0.000158	0.001744

Owenia_fusiformis	OFUSG06205.1	279.2556	-1.33228	0.243291	-5.51512	3.49E-08	1.37E-06
Owenia_fusiformis	OFUSG06228.1	38.62047	-2.30883	0.447642	-4.83873	1.31E-06	3.09E-05
Owenia_fusiformis	OFUSG06252.1	16.35294	-1.67538	0.446332	-3.51854	0.000434	0.0039
Owenia_fusiformis	OFUSG06253.1	187.6127	-1.23689	0.345296	-3.63193	0.000281	0.002781
Owenia_fusiformis	OFUSG06256.1	267.3567	-1.11745	0.260763	-4.2781	1.88E-05	0.000301
Owenia_fusiformis	OFUSG06285.1	109.2115	-1.85177	0.361401	-5.21846	1.80E-07	5.66E-06
Owenia_fusiformis	OFUSG06290.1	791.9782	-1.19449	0.225335	-5.27343	1.34E-07	4.35E-06
Owenia_fusiformis	OFUSG06293.1	48.77771	-1.93737	0.446331	-4.82334	1.41E-06	3.30E-05
Owenia_fusiformis	OFUSG06310.1	133.492	-1.15299	0.323244	-3.56737	0.000361	0.003385
Owenia_fusiformis	OFUSG06378.1	32.19908	-1.35988	0.436469	-3.17859	0.00148	0.010389
Owenia_fusiformis	OFUSG06395.1	249.4125	-1.52543	0.388615	-3.96889	7.22E-05	0.000919
Owenia_fusiformis	OFUSG06403.1	67.19496	-1.13637	0.331999	-3.3871	0.000706	0.005754
Owenia_fusiformis	OFUSG06435.1	3020.659	-1.08208	0.157236	-6.87789	6.07E-12	6.56E-10
Owenia_fusiformis	OFUSG06448.1	13.8848	-1.59128	0.443452	-3.20935	0.00133	0.009512
Owenia_fusiformis	OFUSG06452.1	9.36331	-1.43574	0.437066	-3.5724	0.000354	0.00334
Owenia_fusiformis	OFUSG06464.1	95.46076	-1.03541	0.392409	-2.6183	0.008837	0.041319
Owenia_fusiformis	OFUSG06464.2	517.0171	-1.0113	0.300072	-3.34404	0.000826	0.00653
Owenia_fusiformis	OFUSG06471.1	15.47671	-1.36594	0.445057	-3.73043	0.000191	0.002031
Owenia_fusiformis	OFUSG06495.1	78.28075	-1.01776	0.332759	-3.12177	0.001798	0.012163
Owenia_fusiformis	OFUSG06507.1	379.6814	-1.01077	0.356796	-2.78278	0.005389	0.028342
Owenia_fusiformis	OFUSG06508.1	451.9946	-1.38711	0.298887	-4.6472	3.36E-06	6.97E-05
Owenia_fusiformis	OFUSG06509.1	32.83224	-1.73946	0.427643	-3.90107	9.58E-05	0.001153
Owenia_fusiformis	OFUSG06566.2	217.6793	-1.27328	0.448593	-3.52583	0.000422	0.003825
Owenia_fusiformis	OFUSG06604.1	206.1563	-1.42548	0.282315	-5.07432	3.89E-07	1.08E-05
Owenia_fusiformis	OFUSG06614.1	67.90352	-1.22876	0.406924	-3.13738	0.001705	0.011687
Owenia_fusiformis	OFUSG06618.1	40.04126	-1.13091	0.426173	-2.61179	0.009007	0.041915
Owenia_fusiformis	OFUSG06634.1	889.2066	-1.06583	0.226968	-4.69418	2.68E-06	5.72E-05
Owenia_fusiformis	OFUSG06647.1	150.0049	-1.48515	0.376878	-4.03843	5.38E-05	0.000721
Owenia_fusiformis	OFUSG06699.1	323.5089	-1.02441	0.336333	-3.00557	0.002651	0.016345
Owenia_fusiformis	OFUSG06753.1	694.8632	-1.28116	0.201505	-6.34544	2.22E-10	1.55E-08
Owenia_fusiformis	OFUSG06759.1	558.5158	-1.43906	0.348885	-4.08141	4.48E-05	0.000624
Owenia_fusiformis	OFUSG06768.1	95.98115	-1.33744	0.373648	-3.56741	0.000361	0.003385
Owenia_fusiformis	OFUSG06784.1	71.26522	-1.67651	0.376677	-4.31335	1.61E-05	0.000263
Owenia_fusiformis	OFUSG06786.3	110.3329	-1.42415	0.429841	-3.7803	0.000157	0.001731
Owenia_fusiformis	OFUSG06824.1	25.20268	-1.15398	0.431133	-2.66469	0.007706	0.037321
Owenia_fusiformis	OFUSG06827.1	15.87141	-1.20222	0.445803	-2.63824	0.008334	0.039629
Owenia_fusiformis	OFUSG06851.1	8.946824	-1.42578	0.430747	-2.80654	0.005008	0.026838
Owenia_fusiformis	OFUSG06873.2	94.36257	-1.18867	0.409194	-2.93758	0.003308	0.019461
Owenia_fusiformis	OFUSG06895.1	4429.917	-1.0041	0.21241	-4.72527	2.30E-06	5.05E-05
Owenia_fusiformis	OFUSG06907.1	173.2994	-1.43123	0.34674	-4.05779	4.95E-05	0.000677
Owenia_fusiformis	OFUSG06911.1	600.2885	-1.04299	0.276021	-3.75217	0.000175	0.001894
Owenia_fusiformis	OFUSG06924.1	763.719	-1.18517	0.128488	-9.21352	3.16E-20	1.73E-17
Owenia_fusiformis	OFUSG06934.1	1894.828	-1.56556	0.092423	-16.9232	3.03E-64	3.73E-60
Owenia_fusiformis	OFUSG06935.1	210.4412	-1.17589	0.261323	-4.50893	6.52E-06	0.000124
Owenia_fusiformis	OFUSG06979.1	11.83857	-1.26323	0.442171	-2.78896	0.005288	0.027974
Owenia_fusiformis	OFUSG06985.5	251.3696	-1.17962	0.267214	-4.35325	1.34E-05	0.000228
Owenia_fusiformis	OFUSG06989.3	259.9954	-1.01398	0.302156	-3.37895	0.000728	0.005898
Owenia_fusiformis	OFUSG06990.3	246.0491	-1.08794	0.409903	-2.92916	0.003399	0.019877
Owenia_fusiformis	OFUSG07026.1	308.6021	-1.04958	0.254204	-4.14074	3.46E-05	0.000503
Owenia_fusiformis	OFUSG07047.1	139.7069	-1.65756	0.337495	-4.96762	6.78E-07	1.74E-05
Owenia_fusiformis	OFUSG07109.1	32.13629	-1.11884	0.419771	-2.53704	0.011179	0.049633
Owenia_fusiformis	OFUSG07116.2	1199.074	-1.57116	0.23601	-6.69196	2.20E-11	2.02E-09
Owenia_fusiformis	OFUSG07151.1	65.11366	-1.2019	0.409864	-2.83044	0.004648	0.025366
Owenia_fusiformis	OFUSG07153.2	30.03825	-1.65972	0.434184	-3.87776	0.000105	0.001252
Owenia_fusiformis	OFUSG07168.1	1201.789	-1.84935	0.153923	-12.0026	3.44E-33	5.65E-30
Owenia_fusiformis	OFUSG07173.3	146.175	-1.36012	0.326265	-4.10461	4.05E-05	0.000572
Owenia_fusiformis	OFUSG07174.1	70.31807	-1.19312	0.419974	-2.96722	0.003005	0.018059
Owenia_fusiformis	OFUSG07185.1	288.9543	-1.83758	0.341262	-5.36867	7.93E-08	2.79E-06
Owenia_fusiformis	OFUSG07192.1	57.59915	-1.22088	0.424943	-2.90994	0.003615	0.020882
Owenia_fusiformis	OFUSG07206.1	10.55259	-1.27853	0.439959	-2.91781	0.003525	0.020439
Owenia_fusiformis	OFUSG07220.1	126.9374	-1.35953	0.332795	-4.17654	2.96E-05	0.000442
Owenia_fusiformis	OFUSG07221.1	14.23849	-1.08113	0.4441	-2.63949	0.008303	0.039529
Owenia_fusiformis	OFUSG07247.2	47.3731	-1.91133	0.437348	-4.48152	7.41E-06	0.000138
Owenia_fusiformis	OFUSG07259.1	2905.609	-1.10595	0.278864	-3.95864	7.54E-05	0.00095
Owenia_fusiformis	OFUSG07265.1	129.9562	-1.30277	0.363259	-3.5761	0.000349	0.003305
Owenia_fusiformis	OFUSG07266.1	524.4702	-1.68321	0.348168	-4.77858	1.77E-06	4.02E-05
Owenia_fusiformis	OFUSG07286.1	53.82318	-1.0051	0.40754	-2.64578	0.00815	0.038963
Owenia_fusiformis	OFUSG07308.1	9.568059	-1.20754	0.434752	-2.60247	0.009256	0.042862
Owenia_fusiformis	OFUSG07365.1	150.0249	-2.17919	0.398198	-5.45013	5.03E-08	1.89E-06
Owenia_fusiformis	OFUSG07376.1	1126.366	-1.12753	0.197361	-5.70656	1.15E-08	5.22E-07
Owenia_fusiformis	OFUSG07384.1	78.06539	-1.01558	0.362596	-2.95934	0.003083	0.018424
Owenia_fusiformis	OFUSG07415.1	65.02668	-3.58005	0.399612	-5.8491	4.94E-09	2.46E-07
Owenia_fusiformis	OFUSG07417.2	222.9764	-1.4866	0.412161	-3.60852	0.000308	0.002992
Owenia_fusiformis	OFUSG07421.1	893.2817	-1.11477	0.220923	-5.04194	4.61E-07	1.26E-05
Owenia_fusiformis	OFUSG07428.1	1267.487	-1.45488	0.166595	-8.73484	2.44E-18	9.10E-16
Owenia_fusiformis	OFUSG07460.2	24.44048	-1.25206	0.445151	-3.04134	0.002355	0.014957
Owenia_fusiformis	OFUSG07469.1	815.2457	-1.17072	0.31228	-3.72216	0.000198	0.002087
Owenia_fusiformis	OFUSG07470.1	478.0442	-1.02295	0.27036	-3.76528	0.000166	0.001817
Owenia_fusiformis	OFUSG07488.2	788.0843	-1.01093	0.186312	-5.4332	5.54E-08	2.05E-06
Owenia_fusiformis	OFUSG07528.1	27.13681	-1.2787	0.441212	-4.0515	5.09E-05	0.00069

Owenia_fusiformis	OFUSG07531.1	11.66067	-1.35027	0.446139	-2.94748	0.003204	0.01897
Owenia_fusiformis	OFUSG07555.1	265.1246	-1.13214	0.240906	-4.6881	2.76E-06	5.86E-05
Owenia_fusiformis	OFUSG07568.1	144.9	-2.17269	0.344139	-6.15769	7.38E-10	4.64E-08
Owenia_fusiformis	OFUSG07570.1	70.77998	-1.24476	0.448055	-3.97057	7.17E-05	0.000915
Owenia_fusiformis	OFUSG07572.2	27.97216	-1.36586	0.412744	-3.23439	0.001219	0.008887
Owenia_fusiformis	OFUSG07588.3	207.0561	-1.03196	0.28512	-3.64029	0.000272	0.002703
Owenia_fusiformis	OFUSG07593.1	2902.332	-1.18741	0.193179	-6.15069	7.71E-10	4.83E-08
Owenia_fusiformis	OFUSG07606.1	12.4291	-1.59526	0.445257	-3.16572	0.001547	0.010799
Owenia_fusiformis	OFUSG07629.1	51.07479	-1.06711	0.417453	-2.61326	0.008968	0.041759
Owenia_fusiformis	OFUSG07642.1	51.25636	-1.32549	0.441066	-3.12567	0.001774	0.012049
Owenia_fusiformis	OFUSG07669.1	22.13935	-1.9522	0.445562	-3.96708	7.28E-05	0.000925
Owenia_fusiformis	OFUSG07722.3	369.0391	-1.68521	0.328305	-5.08254	3.72E-07	1.04E-05
Owenia_fusiformis	OFUSG07724.1	133.9342	-1.35329	0.285729	-4.76642	1.88E-06	4.24E-05
Owenia_fusiformis	OFUSG07765.1	54.14041	-1.35244	0.42577	-3.12737	0.001764	0.011996
Owenia_fusiformis	OFUSG07768.2	428.8799	-1.00497	0.283737	-3.56284	0.000367	0.003431
Owenia_fusiformis	OFUSG07779.1	24.19903	-2.24396	0.44556	-4.64799	3.35E-06	6.95E-05
Owenia_fusiformis	OFUSG07800.1	67.64185	-1.11865	0.383518	-2.9094	0.003621	0.020887
Owenia_fusiformis	OFUSG07801.1	283.2929	-1.08943	0.371946	-3.08008	0.002069	0.013533
Owenia_fusiformis	OFUSG07807.1	136.277	-1.43776	0.31135	-4.6354	3.56E-06	7.33E-05
Owenia_fusiformis	OFUSG07844.1	2169.228	-1.03407	0.182307	-5.6729	1.40E-08	6.19E-07
Owenia_fusiformis	OFUSG07863.1	3831.307	-1.12091	0.156683	-7.1544	8.40E-13	1.13E-10
Owenia_fusiformis	OFUSG07886.1	79.15365	-1.54466	0.359764	-4.34366	1.40E-05	0.000236
Owenia_fusiformis	OFUSG07888.1	267.42	-1.43275	0.373697	-3.76878	0.000164	0.001794
Owenia_fusiformis	OFUSG07898.3	14.69884	-1.03165	0.446294	-3.31697	0.00091	0.007042
Owenia_fusiformis	OFUSG07901.2	160.6198	-1.46696	0.356756	-4.12745	3.67E-05	0.000527
Owenia_fusiformis	OFUSG07915.1	143.794	-2.45389	0.402252	-6.01422	1.81E-09	1.03E-07
Owenia_fusiformis	OFUSG07919.1	28.96748	-1.1147	0.430373	-2.70457	0.006839	0.034068
Owenia_fusiformis	OFUSG07931.1	14561.84	-1.12764	0.364659	-3.06284	0.002192	0.014122
Owenia_fusiformis	OFUSG07976.1	241.7233	-1.26227	0.280004	-4.50236	6.72E-06	0.000128
Owenia_fusiformis	OFUSG08000.1	102.3698	-1.53418	0.386946	-4.18833	2.81E-05	0.000423
Owenia_fusiformis	OFUSG08024.1	722.8708	-1.05904	0.334415	-3.31587	0.000914	0.00706
Owenia_fusiformis	OFUSG08038.1	405.4974	-1.01639	0.318772	-3.14431	0.001665	0.011478
Owenia_fusiformis	OFUSG08046.1	14.65707	-1.40495	0.446026	-3.11213	0.001857	0.012449
Owenia_fusiformis	OFUSG08080.1	771.5202	-1.12435	0.203687	-5.49491	3.91E-08	1.51E-06
Owenia_fusiformis	OFUSG08093.3	775.7125	-1.11646	0.224597	-4.94573	7.59E-07	1.93E-05
Owenia_fusiformis	OFUSG08095.1	423.9427	-1.05192	0.306588	-3.39168	0.000695	0.005676
Owenia_fusiformis	OFUSG08098.1	18280.93	-1.29573	0.092622	-13.9922	1.74E-44	4.76E-41
Owenia_fusiformis	OFUSG08169.1	176.3427	-1.11667	0.369564	-2.99611	0.002735	0.016748
Owenia_fusiformis	OFUSG08169.3	373.2607	-1.14102	0.351156	-3.24897	0.001158	0.008514
Owenia_fusiformis	OFUSG08185.2	65.69257	-1.0822	0.443547	-2.72927	0.006347	0.032291
Owenia_fusiformis	OFUSG08207.1	31.20867	-1.06967	0.436025	-2.66954	0.007596	0.036953
Owenia_fusiformis	OFUSG08212.1	53.26391	-1.14295	0.379257	-3.14801	0.001644	0.011356
Owenia_fusiformis	OFUSG08218.1	313.6058	-1.20547	0.251244	-4.79475	1.63E-06	3.75E-05
Owenia_fusiformis	OFUSG08221.4	176.8826	-1.27736	0.42397	-2.88021	0.003974	0.022473
Owenia_fusiformis	OFUSG08283.1	29.28131	-1.31493	0.43502	-3.13033	0.001746	0.011899
Owenia_fusiformis	OFUSG08351.1	263.4195	-1.8938	0.432361	-4.47543	7.63E-06	0.000142
Owenia_fusiformis	OFUSG08361.1	99.61755	-1.50938	0.384242	-3.89627	9.77E-05	0.001173
Owenia_fusiformis	OFUSG08364.1	19.56121	-1.53766	0.445905	-3.38473	0.000712	0.005801
Owenia_fusiformis	OFUSG08366.1	115.3984	-2.23504	0.379358	-5.89525	3.74E-09	1.93E-07
Owenia_fusiformis	OFUSG08387.1	811.4437	-1.48349	0.345576	-4.27028	1.95E-05	0.000311
Owenia_fusiformis	OFUSG08392.1	83.54423	-1.33612	0.412605	-3.34424	0.000825	0.006529
Owenia_fusiformis	OFUSG08405.1	74.35348	-1.41906	0.439389	-3.10908	0.001877	0.012546
Owenia_fusiformis	OFUSG08414.1	33.66749	-1.12618	0.398708	-2.7569	0.005835	0.03019
Owenia_fusiformis	OFUSG08422.1	523.9179	-1.57422	0.264667	-5.98532	2.16E-09	1.20E-07
Owenia_fusiformis	OFUSG08461.1	141.1604	-1.74141	0.422585	-4.38133	1.18E-05	0.000204
Owenia_fusiformis	OFUSG08467.1	18.26977	-1.50075	0.446154	-3.91494	9.04E-05	0.0011
Owenia_fusiformis	OFUSG08556.1	71.47018	-1.49534	0.413563	-3.70799	0.000209	0.002188
Owenia_fusiformis	OFUSG08557.1	114.2393	-1.21661	0.335212	-3.6716	0.000241	0.002461
Owenia_fusiformis	OFUSG08558.1	255.4858	-1.65502	0.303292	-5.47098	4.48E-08	1.71E-06
Owenia_fusiformis	OFUSG08559.1	53.44778	-1.30855	0.40492	-3.29898	0.00097	0.007392
Owenia_fusiformis	OFUSG08563.1	235.1054	-1.08701	0.37232	-3.05305	0.002265	0.014497
Owenia_fusiformis	OFUSG08587.1	32.52363	-1.32473	0.444347	-3.32423	0.000887	0.006899
Owenia_fusiformis	OFUSG08588.1	684.1147	-1.44437	0.202519	-7.14535	8.98E-13	1.19E-10
Owenia_fusiformis	OFUSG08593.1	72.50261	-1.448	0.366004	-3.77516	0.00016	0.001757
Owenia_fusiformis	OFUSG08631.1	1563.373	-1.58617	0.239381	-6.61974	3.60E-11	3.18E-09
Owenia_fusiformis	OFUSG08638.1	333.2643	-1.76263	0.397381	-4.3953	1.11E-05	0.000194
Owenia_fusiformis	OFUSG08659.1	1719.113	-1.57319	0.447196	-3.57462	0.000351	0.003316
Owenia_fusiformis	OFUSG08660.1	647.8004	-2.00433	0.447758	-4.60036	4.22E-06	8.53E-05
Owenia_fusiformis	OFUSG08661.1	60.79856	-1.50751	0.393751	-3.95265	7.73E-05	0.000971
Owenia_fusiformis	OFUSG08663.1	136.8736	-1.43461	0.272444	-5.24952	1.52E-07	4.90E-06
Owenia_fusiformis	OFUSG08684.1	390.3235	-1.07236	0.289838	-3.79184	0.00015	0.001664
Owenia_fusiformis	OFUSG08729.2	62.21006	-1.95874	0.448184	-4.64049	3.48E-06	7.17E-05
Owenia_fusiformis	OFUSG08826.1	187.9493	-1.26591	0.413158	-2.98719	0.002816	0.017112
Owenia_fusiformis	OFUSG08836.1	51.93896	-1.97791	0.421036	-4.71131	2.46E-06	5.35E-05
Owenia_fusiformis	OFUSG08840.1	1726.151	-1.4742	0.353681	-4.16698	3.09E-05	0.000458
Owenia_fusiformis	OFUSG08841.1	53.29937	-1.4744	0.433108	-3.62037	0.000294	0.002882
Owenia_fusiformis	OFUSG08906.2	19.82777	-1.382	0.441667	-3.98207	6.83E-05	0.000878
Owenia_fusiformis	OFUSG08914.1	6.709903	-1.1184	0.432929	-3.15276	0.001617	0.011212
Owenia_fusiformis	OFUSG08967.1	417.1009	-1.00902	0.199469	-5.04345	4.57E-07	1.25E-05
Owenia_fusiformis	OFUSG09006.2	241.3373	-3.26644	0.332547	-9.48177	2.50E-21	1.54E-18

Owenia_fusiformis	OFUSG09118.1	615.0181	-1.14457	0.215447	-5.31673	1.06E-07	3.54E-06
Owenia_fusiformis	OFUSG09122.1	347.4694	-1.20077	0.267336	-4.48763	7.20E-06	0.000135
Owenia_fusiformis	OFUSG09123.2	101.359	-1.50603	0.316422	-4.69208	2.70E-06	5.77E-05
Owenia_fusiformis	OFUSG09141.2	778.5463	-1.35263	0.229906	-5.87308	4.28E-09	2.17E-07
Owenia_fusiformis	OFUSG09153.2	15.37835	-1.31491	0.439742	-3.75486	0.000173	0.001879
Owenia_fusiformis	OFUSG09166.1	112.919	-1.08858	0.309609	-3.51567	0.000439	0.003934
Owenia_fusiformis	OFUSG09207.1	186.0458	-1.33096	0.273478	-4.83447	1.33E-06	3.15E-05
Owenia_fusiformis	OFUSG09209.1	48.65819	-1.88205	0.400591	-4.55144	5.33E-06	0.000104
Owenia_fusiformis	OFUSG09298.2	34.81846	-1.12158	0.447938	-2.62166	0.00875	0.041059
Owenia_fusiformis	OFUSG09314.2	480.4488	-4.68677	0.376	-7.47972	7.45E-14	1.25E-11
Owenia_fusiformis	OFUSG09324.1	75.75958	-1.17135	0.420993	-2.77732	0.005481	0.028719
Owenia_fusiformis	OFUSG09325.1	273.2814	-1.28116	0.347092	-3.66451	0.000248	0.002511
Owenia_fusiformis	OFUSG09377.1	34.91961	-1.02126	0.443306	-2.5943	0.009478	0.04368
Owenia_fusiformis	OFUSG09382.1	55.1168	-1.06603	0.436772	-2.58733	0.009672	0.044357
Owenia_fusiformis	OFUSG09386.1	1036.179	-1.06538	0.266167	-3.99121	6.57E-05	0.000849
Owenia_fusiformis	OFUSG09395.1	130.2809	-1.80671	0.326271	-5.45113	5.01E-08	1.88E-06
Owenia_fusiformis	OFUSG09416.2	271.5258	-1.42226	0.243011	-5.85733	4.70E-09	2.36E-07
Owenia_fusiformis	OFUSG09454.1	54.45937	-1.53915	0.446851	-3.94897	7.85E-05	0.000984
Owenia_fusiformis	OFUSG09454.2	83.35271	-1.21496	0.429331	-2.80681	0.005003	0.026822
Owenia_fusiformis	OFUSG09456.1	345.7577	-1.81623	0.346429	-5.20818	1.91E-07	5.91E-06
Owenia_fusiformis	OFUSG09457.1	85.42699	-1.37489	0.430406	-3.08652	0.002025	0.013324
Owenia_fusiformis	OFUSG09471.1	254.1513	-1.1656	0.231021	-5.03684	4.73E-07	1.28E-05
Owenia_fusiformis	OFUSG09506.1	52.86143	-1.23502	0.428937	-2.92399	0.003456	0.02011
Owenia_fusiformis	OFUSG09507.1	232.5009	-1.20276	0.232642	-5.13894	2.76E-07	8.15E-06
Owenia_fusiformis	OFUSG09546.1	1430.222	-1.17078	0.316822	-3.69374	0.000221	0.00229
Owenia_fusiformis	OFUSG09546.3	415.1318	-1.4483	0.372421	-3.84276	0.000122	0.001403
Owenia_fusiformis	OFUSG09548.1	30.76605	-1.59663	0.446597	-4.28269	1.85E-05	0.000296
Owenia_fusiformis	OFUSG09574.1	63.96768	-2.08417	0.398414	-5.20985	1.89E-07	5.87E-06
Owenia_fusiformis	OFUSG09595.2	285.1048	-1.88304	0.316536	-6.05801	1.38E-09	7.95E-08
Owenia_fusiformis	OFUSG09614.3	335.35	-1.11712	0.285209	-3.91879	8.90E-05	0.001087
Owenia_fusiformis	OFUSG09615.1	173.577	-1.1252	0.329403	-3.49369	0.000476	0.004211
Owenia_fusiformis	OFUSG09642.1	5915.594	-1.18453	0.165222	-7.17106	7.44E-13	1.01E-10
Owenia_fusiformis	OFUSG09691.1	15.87671	-1.24376	0.446662	-3.00976	0.002615	0.01617
Owenia_fusiformis	OFUSG09693.1	1287.333	-2.01053	0.302341	-6.61753	3.65E-11	3.21E-09
Owenia_fusiformis	OFUSG09700.1	68.16061	-1.82793	0.408111	-4.34545	1.39E-05	0.000234
Owenia_fusiformis	OFUSG09700.3	225.452	-1.12089	0.2387	-4.67699	2.91E-06	6.14E-05
Owenia_fusiformis	OFUSG09702.1	123.6746	-1.05192	0.28126	-3.71871	0.0002	0.00211
Owenia_fusiformis	OFUSG09704.2	470.6395	-2.14589	0.353863	-5.98485	2.17E-09	1.20E-07
Owenia_fusiformis	OFUSG09704.3	116.3874	-1.63469	0.434603	-3.73238	0.00019	0.002021
Owenia_fusiformis	OFUSG09735.1	58.02605	-1.04986	0.42393	-2.55057	0.010755	0.048103
Owenia_fusiformis	OFUSG09752.2	1575.501	-1.5475	0.159546	-9.68385	3.53E-22	2.29E-19
Owenia_fusiformis	OFUSG09764.1	935.6578	-1.4051	0.128519	-10.9237	8.88E-28	9.11E-25
Owenia_fusiformis	OFUSG09796.1	99.12568	-1.28349	0.411413	-3.04782	0.002305	0.014699
Owenia_fusiformis	OFUSG09846.1	46.99447	-1.21303	0.446641	-3.00979	0.002614	0.01617
Owenia_fusiformis	OFUSG09847.1	1442.262	-1.36382	0.170578	-7.99044	1.34E-15	3.41E-13
Owenia_fusiformis	OFUSG09886.2	305.4044	-1.40388	0.352854	-3.96243	7.42E-05	0.000938
Owenia_fusiformis	OFUSG09917.2	53.53081	-2.08706	0.433182	-4.93088	8.19E-07	2.05E-05
Owenia_fusiformis	OFUSG09921.3	75.3727	-1.15056	0.410751	-3.45432	0.000552	0.004724
Owenia_fusiformis	OFUSG09966.1	577.965	-1.52569	0.174013	-8.74616	2.21E-18	8.36E-16
Owenia_fusiformis	OFUSG09971.2	275.152	-1.0712	0.322246	-3.37703	0.000733	0.005928
Owenia_fusiformis	OFUSG09986.1	21.40288	-1.86513	0.446304	-3.76265	0.000168	0.001831
Owenia_fusiformis	OFUSG10006.1	213.7732	-1.44989	0.409104	-3.44925	0.000562	0.004789
Owenia_fusiformis	OFUSG10008.1	15.92287	-1.09317	0.446144	-2.59509	0.009457	0.043615
Owenia_fusiformis	OFUSG10018.1	19.50077	-1.38633	0.444482	-3.16057	0.001575	0.010967
Owenia_fusiformis	OFUSG10063.2	282.5211	-1.16451	0.419485	-3.02874	0.002456	0.01542
Owenia_fusiformis	OFUSG10089.1	251.7995	-1.75259	0.434869	-3.95236	7.74E-05	0.000971
Owenia_fusiformis	OFUSG10130.1	133.3116	-1.39801	0.323331	-4.33264	1.47E-05	0.000246
Owenia_fusiformis	OFUSG10172.1	1297.346	-1.4507	0.147678	-9.81997	9.24E-23	6.32E-20
Owenia_fusiformis	OFUSG10173.1	807.3119	-1.13406	0.339997	-3.3491	0.000811	0.006434
Owenia_fusiformis	OFUSG10202.1	38.40655	-1.33381	0.419031	-3.07199	0.002126	0.013813
Owenia_fusiformis	OFUSG10231.1	338.948	-1.01845	0.273378	-3.74059	0.000184	0.001972
Owenia_fusiformis	OFUSG10270.1	33.41642	-1.36574	0.446982	-3.12121	0.001801	0.012172
Owenia_fusiformis	OFUSG10276.4	150.9786	-1.00468	0.381583	-2.77411	0.005535	0.028954
Owenia_fusiformis	OFUSG10277.1	23.4321	-1.25583	0.427206	-3.09151	0.001991	0.013134
Owenia_fusiformis	OFUSG10314.2	26.00409	-1.46211	0.401884	-4.03557	5.45E-05	0.000728
Owenia_fusiformis	OFUSG10342.1	49.6887	-1.03927	0.427904	-2.79311	0.00522	0.027685
Owenia_fusiformis	OFUSG10344.1	1688.143	-1.15927	0.334899	-3.41678	0.000634	0.005271
Owenia_fusiformis	OFUSG10373.1	510.4039	-1.12797	0.193552	-5.83762	5.30E-09	2.62E-07
Owenia_fusiformis	OFUSG10390.2	81.42794	-1.00323	0.344914	-2.87354	0.004059	0.022843
Owenia_fusiformis	OFUSG10393.1	31.0068	-1.307	0.41597	-2.98326	0.002852	0.017295
Owenia_fusiformis	OFUSG10405.1	22.58599	-1.47689	0.44788	-3.32346	0.000889	0.006911
Owenia_fusiformis	OFUSG10421.1	1039.189	-1.09548	0.230221	-4.74473	2.09E-06	4.66E-05
Owenia_fusiformis	OFUSG10421.4	98.8861	-2.54873	0.401593	-6.14878	7.81E-10	4.88E-08
Owenia_fusiformis	OFUSG10436.1	28.22916	-1.9005	0.444441	-4.12748	3.67E-05	0.000527
Owenia_fusiformis	OFUSG10449.4	12.11558	-1.67168	0.439157	-3.23863	0.001201	0.008772
Owenia_fusiformis	OFUSG10451.1	12.51183	-1.69705	0.435866	-3.89678	9.75E-05	0.001171
Owenia_fusiformis	OFUSG10461.2	732.7565	-1.35169	0.36043	-3.77121	0.000162	0.001778
Owenia_fusiformis	OFUSG10485.1	9.71513	-1.28419	0.444024	-2.58625	0.009703	0.044489
Owenia_fusiformis	OFUSG10499.2	339.4611	-1.49283	0.357218	-4.20367	2.63E-05	0.000398
Owenia_fusiformis	OFUSG10522.3	113.8732	-1.02049	0.365118	-2.88233	0.003947	0.022345

Owenia_fusiformis	OFUSG10533.1	275.103	-1.09475	0.331346	-3.34232	0.000831	0.006557
Owenia_fusiformis	OFUSG10549.1	33.69409	-1.23059	0.404248	-2.92684	0.003424	0.019978
Owenia_fusiformis	OFUSG10556.1	1998.776	-1.15578	0.255683	-4.50651	6.59E-06	0.000126
Owenia_fusiformis	OFUSG10595.1	1080.778	-1.17285	0.168683	-6.94422	3.81E-12	4.32E-10
Owenia_fusiformis	OFUSG10608.1	619.7061	-2.66176	0.287914	-9.18426	4.14E-20	2.22E-17
Owenia_fusiformis	OFUSG10613.1	383.2281	-1.66214	0.43293	-3.77518	0.00016	0.001757
Owenia_fusiformis	OFUSG10674.1	283.9268	-1.63501	0.233483	-6.9757	3.04E-12	3.52E-10
Owenia_fusiformis	OFUSG10726.1	71.97819	-1.31491	0.390621	-3.5633	0.000366	0.003428
Owenia_fusiformis	OFUSG10752.1	8.988996	-1.32066	0.439745	-2.80056	0.005101	0.027242
Owenia_fusiformis	OFUSG10810.1	57.98392	-1.07284	0.356355	-2.95992	0.003077	0.018399
Owenia_fusiformis	OFUSG10815.2	39.9411	-1.14341	0.448434	-2.74597	0.006033	0.030903
Owenia_fusiformis	OFUSG10823.2	22.79661	-1.40875	0.446911	-3.38733	0.000706	0.005753
Owenia_fusiformis	OFUSG10859.2	562.1516	-1.05744	0.250536	-4.21959	2.45E-05	0.000376
Owenia_fusiformis	OFUSG10864.2	1676.456	-1.06368	0.31689	-3.3385	0.000842	0.006632
Owenia_fusiformis	OFUSG10932.1	29.78714	-1.40685	0.404072	-3.4383	0.000585	0.004941
Owenia_fusiformis	OFUSG10977.1	87.7153	-1.27195	0.334159	-3.73915	0.000185	0.00198
Owenia_fusiformis	OFUSG11016.1	39.20467	-1.31582	0.38619	-3.28012	0.001038	0.007803
Owenia_fusiformis	OFUSG11024.1	24.3599	-1.35355	0.434995	-3.03491	0.002406	0.015213
Owenia_fusiformis	OFUSG11046.1	90.54694	-1.72907	0.379027	-4.44249	8.89E-06	0.000161
Owenia_fusiformis	OFUSG11066.1	85.73327	-1.02282	0.342559	-2.91541	0.003552	0.020564
Owenia_fusiformis	OFUSG11119.1	343.2781	-1.03456	0.28793	-3.60684	0.00031	0.003008
Owenia_fusiformis	OFUSG11126.1	237.9301	-2.5012	0.362567	-6.50323	7.86E-11	6.22E-09
Owenia_fusiformis	OFUSG11128.1	344.9705	-1.84393	0.324287	-5.66589	1.46E-08	6.40E-07
Owenia_fusiformis	OFUSG11148.1	367.654	-1.69575	0.321653	-5.32762	9.95E-08	3.38E-06
Owenia_fusiformis	OFUSG11177.1	74.19402	-1.81041	0.366293	-4.92159	8.58E-07	2.14E-05
Owenia_fusiformis	OFUSG11178.1	331.1022	-1.53592	0.216302	-7.06083	1.66E-12	2.04E-10
Owenia_fusiformis	OFUSG11179.4	81.23578	-1.3211	0.445392	-3.23994	0.001196	0.008737
Owenia_fusiformis	OFUSG11179.5	30.22153	-2.00321	0.447569	-4.67522	2.94E-06	6.19E-05
Owenia_fusiformis	OFUSG11184.1	479.0304	-1.04142	0.393156	-2.7992	0.005123	0.027324
Owenia_fusiformis	OFUSG11185.1	7068.113	-1.04565	0.175029	-5.97194	2.34E-09	1.28E-07
Owenia_fusiformis	OFUSG11186.1	937.116	-1.13179	0.161397	-7.01587	2.29E-12	2.71E-10
Owenia_fusiformis	OFUSG11211.1	17.8555	-1.49658	0.445156	-3.19215	0.001412	0.009997
Owenia_fusiformis	OFUSG11219.1	43.89442	-1.36382	0.428783	-3.22105	0.001277	0.009216
Owenia_fusiformis	OFUSG11274.1	178.3181	-1.09142	0.307062	-3.54775	0.000389	0.003596
Owenia_fusiformis	OFUSG11327.1	12.85908	-1.05288	0.438047	-3.39434	0.000688	0.005636
Owenia_fusiformis	OFUSG11338.1	42.64328	-1.9005	0.446658	-3.94114	8.11E-05	0.00101
Owenia_fusiformis	OFUSG11342.1	106.3067	-1.30035	0.318168	-4.07805	4.54E-05	0.000631
Owenia_fusiformis	OFUSG11367.1	123.5202	-1.36391	0.444892	-3.3931	0.000691	0.005658
Owenia_fusiformis	OFUSG11397.1	19.40518	-1.40863	0.447687	-2.98127	0.002871	0.017365
Owenia_fusiformis	OFUSG11415.1	244.1734	-1.34542	0.242657	-5.54645	2.92E-08	1.17E-06
Owenia_fusiformis	OFUSG11422.1	12.01827	-1.05345	0.442006	-2.65285	0.007981	0.038263
Owenia_fusiformis	OFUSG11434.1	23.0157	-1.61631	0.447283	-3.53135	0.000413	0.003764
Owenia_fusiformis	OFUSG11469.1	274.3313	-1.00466	0.206657	-4.85979	1.18E-06	2.83E-05
Owenia_fusiformis	OFUSG11492.1	284.5407	-1.55488	0.235648	-6.57593	4.84E-11	4.08E-09
Owenia_fusiformis	OFUSG11519.1	168.1058	-1.66191	0.356692	-4.74871	2.05E-06	4.59E-05
Owenia_fusiformis	OFUSG11532.1	2166.936	-1.41095	0.282078	-5.03209	4.85E-07	1.31E-05
Owenia_fusiformis	OFUSG11532.2	18514.75	-1.41893	0.20267	-7.0011	2.54E-12	2.99E-10
Owenia_fusiformis	OFUSG11534.1	25.05753	-1.23771	0.445674	-2.80571	0.005021	0.026896
Owenia_fusiformis	OFUSG11553.1	36.06246	-1.09997	0.438906	-2.56183	0.010412	0.046954
Owenia_fusiformis	OFUSG11569.1	309.8386	-1.48711	0.219729	-6.74759	1.50E-11	1.44E-09
Owenia_fusiformis	OFUSG11583.1	197.0837	-1.19813	0.360641	-3.41253	0.000644	0.005342
Owenia_fusiformis	OFUSG11586.1	710.7743	-1.00793	0.186877	-5.38299	7.33E-08	2.62E-06
Owenia_fusiformis	OFUSG11654.1	22.47065	-2.0611	0.438321	-4.57445	4.77E-06	9.47E-05
Owenia_fusiformis	OFUSG11669.1	5869.747	-1.3206	0.287792	-4.57176	4.84E-06	9.57E-05
Owenia_fusiformis	OFUSG11674.1	272.9871	-1.44047	0.343464	-4.14942	3.33E-05	0.000487
Owenia_fusiformis	OFUSG11676.1	149.2544	-1.2109	0.369908	-3.2666	0.001088	0.008103
Owenia_fusiformis	OFUSG11691.1	33.84893	-1.33653	0.445884	-2.79437	0.0052	0.027618
Owenia_fusiformis	OFUSG11695.1	55.26624	-1.40332	0.392552	-3.67903	0.000234	0.002406
Owenia_fusiformis	OFUSG11725.1	1000.893	-1.14649	0.25717	-4.44318	8.86E-06	0.000161
Owenia_fusiformis	OFUSG11740.1	959.7528	-1.60178	0.438319	-4.08497	4.41E-05	0.000615
Owenia_fusiformis	OFUSG11791.1	1645.704	-2.0614	0.27886	-7.44749	9.51E-14	1.54E-11
Owenia_fusiformis	OFUSG11791.3	1429.048	-1.14327	0.236857	-4.83684	1.32E-06	3.12E-05
Owenia_fusiformis	OFUSG11800.1	110.6168	-1.34764	0.431559	-4.29274	1.76E-05	0.000285
Owenia_fusiformis	OFUSG11805.1	302.2875	-1.26282	0.311357	-4.09375	4.24E-05	0.000596
Owenia_fusiformis	OFUSG11817.1	3613.875	-1.0872	0.220428	-4.92007	8.65E-07	2.16E-05
Owenia_fusiformis	OFUSG11837.1	41.74581	-1.70547	0.444291	-3.83604	0.000125	0.001438
Owenia_fusiformis	OFUSG11931.1	21.35004	-1.56258	0.447271	-4.14994	3.33E-05	0.000487
Owenia_fusiformis	OFUSG11975.1	55.69307	-1.29788	0.445469	-3.16011	0.001577	0.010978
Owenia_fusiformis	OFUSG11999.1	21.73298	-1.53265	0.444868	-4.19196	2.77E-05	0.000418
Owenia_fusiformis	OFUSG12064.1	158.0923	-1.16779	0.306526	-3.84361	0.000121	0.0014
Owenia_fusiformis	OFUSG12068.1	123.8756	-1.20666	0.318433	-3.8242	0.000131	0.001493
Owenia_fusiformis	OFUSG12109.1	102.4782	-1.177	0.339539	-3.50953	0.000449	0.004003
Owenia_fusiformis	OFUSG12116.1	20.15973	-1.15348	0.446804	-2.6531	0.007976	0.038243
Owenia_fusiformis	OFUSG12154.1	223.6922	-1.04035	0.24179	-4.32571	1.52E-05	0.000252
Owenia_fusiformis	OFUSG12159.1	1310.933	-1.20446	0.225564	-5.34486	9.05E-08	3.11E-06
Owenia_fusiformis	OFUSG12162.2	93.64096	-1.19711	0.421724	-2.90022	0.003729	0.021333
Owenia_fusiformis	OFUSG12220.1	69.0291	-1.00939	0.369244	-2.70616	0.006807	0.033967
Owenia_fusiformis	OFUSG12258.4	549.1541	-1.08623	0.425923	-2.64172	0.008249	0.03933
Owenia_fusiformis	OFUSG12296.1	64.98485	-1.51051	0.363365	-4.05651	4.98E-05	0.00068
Owenia_fusiformis	OFUSG12362.1	18.67667	-1.22156	0.447886	-2.75597	0.005852	0.030252

Owenia_fusiformis	OFUSG12368.1	2885.902	-2.29719	0.239237	-9.62885	6.04E-22	3.81E-19
Owenia_fusiformis	OFUSG12395.1	276.5771	-1.2755	0.291598	-4.38303	1.17E-05	0.000203
Owenia_fusiformis	OFUSG12487.1	19.2417	-2.09054	0.445528	-3.98	6.89E-05	0.000883
Owenia_fusiformis	OFUSG12514.1	706.8276	-1.12026	0.127421	-8.77597	1.69E-18	6.56E-16
Owenia_fusiformis	OFUSG12537.1	94.18217	-1.63254	0.314417	-5.08429	3.69E-07	1.03E-05
Owenia_fusiformis	OFUSG12539.1	7274.867	-1.39275	0.179662	-7.75112	9.11E-15	1.93E-12
Owenia_fusiformis	OFUSG12609.2	332.2644	-1.14487	0.267575	-4.30425	1.68E-05	0.000274
Owenia_fusiformis	OFUSG12614.1	64.94448	-2.77974	0.429745	-5.43266	5.55E-08	2.05E-06
Owenia_fusiformis	OFUSG12616.1	788.2498	-1.0581	0.137063	-7.71155	1.24E-14	2.51E-12
Owenia_fusiformis	OFUSG12695.1	641.0155	-1.65023	0.251675	-6.52729	6.70E-11	5.43E-09
Owenia_fusiformis	OFUSG12733.1	74.60638	-1.3555	0.342498	-3.87189	0.000108	0.001275
Owenia_fusiformis	OFUSG12735.1	1324.383	-1.18908	0.445968	-2.66623	0.007671	0.037218
Owenia_fusiformis	OFUSG12749.1	7.823511	-1.11995	0.415227	-2.57324	0.010075	0.04577
Owenia_fusiformis	OFUSG12751.1	100.7333	-2.07016	0.390244	-5.09292	3.53E-07	9.99E-06
Owenia_fusiformis	OFUSG12775.1	208.7186	-1.23648	0.381007	-3.17038	0.001522	0.010645
Owenia_fusiformis	OFUSG12782.1	100.2419	-1.47048	0.407826	-3.54738	0.000389	0.003596
Owenia_fusiformis	OFUSG12789.1	214.7589	-1.59913	0.306776	-5.28276	1.27E-07	4.18E-06
Owenia_fusiformis	OFUSG12833.1	177.3893	-1.11716	0.279213	-3.96043	7.48E-05	0.000944
Owenia_fusiformis	OFUSG12846.2	248.1338	-1.29041	0.406014	-3.128	0.00176	0.011977
Owenia_fusiformis	OFUSG12846.3	163.2372	-1.57976	0.381206	-4.24415	2.19E-05	0.000344
Owenia_fusiformis	OFUSG12862.1	3107.68	-1.14839	0.166156	-6.90867	4.89E-12	5.40E-10
Owenia_fusiformis	OFUSG12913.1	433.1512	-1.42209	0.239641	-5.95812	2.55E-09	1.38E-07
Owenia_fusiformis	OFUSG12925.1	20.43703	-1.08404	0.437988	-2.62178	0.008747	0.041057
Owenia_fusiformis	OFUSG12982.2	20.26177	-1.24708	0.446528	-3.02056	0.002523	0.015746
Owenia_fusiformis	OFUSG12995.1	31.86727	-1.44668	0.404304	-3.38533	0.000711	0.00579
Owenia_fusiformis	OFUSG12997.1	27.95395	-1.13861	0.442589	-2.73924	0.006158	0.031438
Owenia_fusiformis	OFUSG13018.1	148.5106	-2.4386	0.370826	-6.60359	4.01E-11	3.47E-09
Owenia_fusiformis	OFUSG13045.1	154.1796	-1.18862	0.351035	-3.41932	0.000628	0.005227
Owenia_fusiformis	OFUSG13048.1	11.4092	-1.0224	0.417905	-3.29776	0.000975	0.007417
Owenia_fusiformis	OFUSG13057.1	153.7392	-1.64134	0.385184	-4.29316	1.76E-05	0.000285
Owenia_fusiformis	OFUSG13069.1	4157.486	-1.65595	0.229538	-7.20746	5.70E-13	7.89E-11
Owenia_fusiformis	OFUSG13069.2	642.7053	-1.27342	0.246336	-5.16552	2.40E-07	7.25E-06
Owenia_fusiformis	OFUSG13080.1	219.9488	-1.03189	0.333782	-3.13067	0.001744	0.011888
Owenia_fusiformis	OFUSG13105.1	9649.095	-1.92668	0.175589	-10.9714	5.24E-28	5.87E-25
Owenia_fusiformis	OFUSG13106.1	14.98786	-1.20159	0.446797	-2.86007	0.004236	0.023637
Owenia_fusiformis	OFUSG13126.2	11.19151	-1.95105	0.441935	-3.34403	0.000826	0.00653
Owenia_fusiformis	OFUSG13140.1	1461.301	-1.30351	0.322971	-4.03097	5.55E-05	0.00074
Owenia_fusiformis	OFUSG13147.1	277.7172	-1.09981	0.305722	-3.67778	0.000235	0.002415
Owenia_fusiformis	OFUSG13184.1	584.0951	-1.01732	0.165638	-6.14255	8.12E-10	5.02E-08
Owenia_fusiformis	OFUSG13220.1	92.63449	-1.7578	0.433583	-4.44348	8.85E-06	0.000161
Owenia_fusiformis	OFUSG13251.1	49.3077	-2.79077	0.418806	-5.14023	2.74E-07	8.11E-06
Owenia_fusiformis	OFUSG13266.2	123.8691	-1.21684	0.326161	-3.74877	0.000178	0.001916
Owenia_fusiformis	OFUSG13352.1	27.37733	-1.45986	0.428156	-3.53218	0.000412	0.003755
Owenia_fusiformis	OFUSG13353.1	74.67134	-1.72892	0.342982	-4.9383	7.88E-07	1.99E-05
Owenia_fusiformis	OFUSG13359.1	347.4267	-1.21873	0.210315	-5.77433	7.73E-09	3.65E-07
Owenia_fusiformis	OFUSG13374.1	39.02349	-1.12962	0.416113	-2.65439	0.007945	0.03816
Owenia_fusiformis	OFUSG13377.1	25.83292	-1.17677	0.378615	-3.85372	0.000116	0.001352
Owenia_fusiformis	OFUSG13419.1	43.60215	-1.22077	0.42997	-3.01579	0.002563	0.015928
Owenia_fusiformis	OFUSG13428.1	36.20306	-1.48559	0.448402	-3.43826	0.000585	0.004941
Owenia_fusiformis	OFUSG13429.1	480.2134	-5.61467	0.315056	-7.78798	6.81E-15	1.47E-12
Owenia_fusiformis	OFUSG13460.1	27.47864	-1.05828	0.414528	-3.90365	9.48E-05	0.001142
Owenia_fusiformis	OFUSG13483.2	85.60769	-1.45631	0.441697	-2.9599	0.003077	0.018399
Owenia_fusiformis	OFUSG13500.1	83.87276	-1.06085	0.317712	-3.30207	0.00096	0.007329
Owenia_fusiformis	OFUSG13523.1	927.1248	-1.88254	0.287967	-6.53383	6.41E-11	5.21E-09
Owenia_fusiformis	OFUSG13532.1	157.4148	-1.02663	0.299699	-3.44271	0.000576	0.004882
Owenia_fusiformis	OFUSG13543.4	247.417	-2.13668	0.322042	-6.61666	3.67E-11	3.22E-09
Owenia_fusiformis	OFUSG13593.1	240.4234	-1.91022	0.316478	-6.0109	1.84E-09	1.04E-07
Owenia_fusiformis	OFUSG13597.1	12.80975	-2.16983	0.42548	-4.13575	3.54E-05	0.000512
Owenia_fusiformis	OFUSG13611.1	8.576023	-1.019	0.39397	-2.82552	0.00472	0.025678
Owenia_fusiformis	OFUSG13611.2	50.64494	-1.7012	0.443388	-4.30998	1.63E-05	0.000267
Owenia_fusiformis	OFUSG13628.1	238.9704	-1.58784	0.375977	-4.20537	2.61E-05	0.000396
Owenia_fusiformis	OFUSG13669.1	27.01592	-1.31178	0.445735	-3.11412	0.001845	0.012394
Owenia_fusiformis	OFUSG13678.1	318.7728	-1.33686	0.428061	-2.93702	0.003314	0.019491
Owenia_fusiformis	OFUSG13731.1	19.88773	-1.2113	0.448095	-2.65505	0.00793	0.038111
Owenia_fusiformis	OFUSG13749.1	443.9045	-1.35949	0.265778	-5.10528	3.30E-07	9.46E-06
Owenia_fusiformis	OFUSG13786.1	24.21227	-1.60248	0.441442	-3.60563	0.000311	0.003018
Owenia_fusiformis	OFUSG13834.1	36.65698	-1.10298	0.419203	-4.18358	2.87E-05	0.00043
Owenia_fusiformis	OFUSG13835.1	2453.094	-1.05514	0.212159	-4.97065	6.67E-07	1.72E-05
Owenia_fusiformis	OFUSG13857.1	16.65739	-1.20638	0.447129	-2.65397	0.007955	0.038175
Owenia_fusiformis	OFUSG13888.1	65.77657	-1.31317	0.3786	-3.47853	0.000504	0.004408
Owenia_fusiformis	OFUSG13896.1	135.4841	-2.21251	0.428314	-5.60263	2.11E-08	8.86E-07
Owenia_fusiformis	OFUSG13919.1	18.16207	-1.92854	0.446299	-3.93244	8.41E-05	0.001041
Owenia_fusiformis	OFUSG13932.1	159.7219	-1.7654	0.316632	-5.53879	3.05E-08	1.22E-06
Owenia_fusiformis	OFUSG13933.1	2026.452	-1.4475	0.40254	-3.56577	0.000363	0.003402
Owenia_fusiformis	OFUSG13947.2	481.6886	-1.07159	0.324217	-3.28938	0.001004	0.007599
Owenia_fusiformis	OFUSG13983.1	461.8972	-2.1997	0.193709	-11.2973	1.35E-29	1.59E-26
Owenia_fusiformis	OFUSG14006.1	2643.182	-1.056	0.17781	-5.93545	2.93E-09	1.56E-07
Owenia_fusiformis	OFUSG14015.1	258.4096	-1.23645	0.316455	-3.884	0.000103	0.001224
Owenia_fusiformis	OFUSG14066.1	7330.281	-1.01646	0.13935	-7.29326	3.03E-13	4.54E-11
Owenia_fusiformis	OFUSG14140.1	17.75853	-1.22437	0.443806	-2.61415	0.008945	0.041666

Owenia_fusiformis	OFUSG14143.1	583.857	-1.26297	0.213865	-5.89943	3.65E-09	1.89E-07
Owenia_fusiformis	OFUSG14159.1	489.6337	-1.14737	0.223317	-5.1191	3.07E-07	8.88E-06
Owenia_fusiformis	OFUSG14176.1	36.68374	-1.06533	0.415603	-2.67331	0.007511	0.036642
Owenia_fusiformis	OFUSG14179.1	39.31067	-1.72918	0.442161	-4.07777	4.55E-05	0.000631
Owenia_fusiformis	OFUSG14189.1	73.44137	-1.41748	0.422661	-3.67812	0.000235	0.002414
Owenia_fusiformis	OFUSG14205.1	40.68357	-1.08972	0.443678	-2.63254	0.008475	0.040144
Owenia_fusiformis	OFUSG14207.1	118.4649	-1.75388	0.405193	-4.57856	4.68E-06	9.33E-05
Owenia_fusiformis	OFUSG14256.1	146.0458	-1.06306	0.306036	-3.45458	0.000551	0.004721
Owenia_fusiformis	OFUSG14267.2	9.525258	-1.20034	0.425731	-2.64538	0.00816	0.038979
Owenia_fusiformis	OFUSG14271.1	13.84874	-1.08542	0.441076	-2.58551	0.009724	0.044543
Owenia_fusiformis	OFUSG14288.1	7.739013	-1.49988	0.429853	-3.50612	0.000455	0.004045
Owenia_fusiformis	OFUSG14293.1	6.841397	-1.04298	0.362618	-2.93672	0.003317	0.019505
Owenia_fusiformis	OFUSG14318.1	67.99767	-1.1007	0.388758	-2.8905	0.003846	0.021896
Owenia_fusiformis	OFUSG14380.1	44.06698	-1.62649	0.428088	-3.63046	0.000283	0.002794
Owenia_fusiformis	OFUSG14462.1	37.12435	-1.0802	0.42231	-2.66517	0.007695	0.03729
Owenia_fusiformis	OFUSG14476.1	305.5682	-1.42024	0.316783	-4.50135	6.75E-06	0.000128
Owenia_fusiformis	OFUSG14509.1	46.15047	-1.2585	0.434097	-3.06876	0.002149	0.013922
Owenia_fusiformis	OFUSG14519.1	173.3119	-1.53179	0.277987	-5.51959	3.40E-08	1.34E-06
Owenia_fusiformis	OFUSG14537.1	103.0997	-1.47441	0.448337	-5.04716	4.48E-07	1.23E-05
Owenia_fusiformis	OFUSG14599.1	123.6285	-1.49731	0.370911	-4.00608	6.17E-05	0.000804
Owenia_fusiformis	OFUSG14605.1	15.44694	-2.18467	0.435535	-4.34146	1.42E-05	0.000238
Owenia_fusiformis	OFUSG14623.2	102.0775	-1.02075	0.383223	-2.62969	0.008546	0.040397
Owenia_fusiformis	OFUSG14673.1	488.9152	-2.68779	0.332165	-8.21889	2.05E-16	5.95E-14
Owenia_fusiformis	OFUSG14676.1	24.49626	-1.38365	0.43437	-3.05906	0.00222	0.01428
Owenia_fusiformis	OFUSG14716.1	669.0146	-1.09991	0.217106	-5.08614	3.65E-07	1.03E-05
Owenia_fusiformis	OFUSG14785.1	38.12555	-1.21231	0.448462	-3.24237	0.001185	0.008676
Owenia_fusiformis	OFUSG14794.1	16.9789	-1.75758	0.444675	-3.63934	0.000273	0.002711
Owenia_fusiformis	OFUSG14796.1	52.63105	-1.25428	0.441631	-3.2331	0.001225	0.008917
Owenia_fusiformis	OFUSG14804.1	108.9665	-1.25427	0.33384	-3.73274	0.000189	0.002021
Owenia_fusiformis	OFUSG14810.1	10.09889	-1.43484	0.425959	-2.70907	0.006747	0.033739
Owenia_fusiformis	OFUSG14836.3	79.16177	-2.79735	0.406721	-5.48453	4.15E-08	1.60E-06
Owenia_fusiformis	OFUSG14838.2	52.2672	-1.45486	0.439398	-3.41198	0.000645	0.005351
Owenia_fusiformis	OFUSG14900.1	19.36807	-1.91903	0.44685	-3.73091	0.000191	0.00203
Owenia_fusiformis	OFUSG14915.1	341.7001	-1.50245	0.434364	-3.6758	0.000237	0.002431
Owenia_fusiformis	OFUSG14946.2	82.30854	-1.13717	0.420819	-2.67045	0.007575	0.036882
Owenia_fusiformis	OFUSG14954.1	11.13365	-1.13516	0.446113	-2.56375	0.010355	0.046782
Owenia_fusiformis	OFUSG15023.1	34.4097	-1.04827	0.440967	-2.81201	0.004923	0.026473
Owenia_fusiformis	OFUSG15026.1	90.43022	-1.18368	0.346723	-3.39858	0.000677	0.005574
Owenia_fusiformis	OFUSG15060.2	355.8962	-1.1054	0.232324	-4.80225	1.57E-06	3.63E-05
Owenia_fusiformis	OFUSG15076.1	12.1938	-1.45532	0.444351	-3.61009	0.000306	0.002976
Owenia_fusiformis	OFUSG15083.1	14.83318	-1.53283	0.444241	-3.14607	0.001655	0.011418
Owenia_fusiformis	OFUSG15092.1	58.69484	-1.0048	0.358808	-2.72145	0.0065	0.032859
Owenia_fusiformis	OFUSG15106.1	153.0215	-1.04691	0.263124	-3.96931	7.21E-05	0.000918
Owenia_fusiformis	OFUSG15127.1	122.6253	-1.49449	0.338259	-4.44143	8.94E-06	0.000162
Owenia_fusiformis	OFUSG15169.1	366.0736	-1.4732	0.392371	-3.65293	0.000259	0.002597
Owenia_fusiformis	OFUSG15172.3	360.9737	-1.06349	0.270612	-3.92276	8.75E-05	0.001073
Owenia_fusiformis	OFUSG15175.1	3026.468	-1.04513	0.170923	-6.11025	9.95E-10	6.02E-08
Owenia_fusiformis	OFUSG15241.1	583.3025	-1.21434	0.362499	-3.42083	0.000624	0.005205
Owenia_fusiformis	OFUSG15276.1	11.6625	-1.31215	0.436599	-2.90951	0.00362	0.020887
Owenia_fusiformis	OFUSG15302.1	43.86075	-1.904	0.446345	-4.07798	4.54E-05	0.000631
Owenia_fusiformis	OFUSG15303.1	77.76925	-1.58825	0.418926	-3.91501	9.04E-05	0.0011
Owenia_fusiformis	OFUSG15355.1	469.6221	-2.02276	0.307437	-6.60885	3.87E-11	3.37E-09
Owenia_fusiformis	OFUSG15451.2	156.4813	-1.33511	0.31291	-4.23528	2.28E-05	0.000356
Owenia_fusiformis	OFUSG15493.1	11.16497	-1.30294	0.441138	-2.8082	0.004982	0.026718
Owenia_fusiformis	OFUSG15499.1	137.2583	-1.22455	0.297617	-4.14724	3.37E-05	0.000491
Owenia_fusiformis	OFUSG15520.1	102.0527	-2.05883	0.448543	-5.02428	5.05E-07	1.35E-05
Owenia_fusiformis	OFUSG15584.2	16.49467	-1.1339	0.431389	-3.62544	0.000288	0.002837
Owenia_fusiformis	OFUSG15592.1	952.1152	-1.41433	0.43676	-3.08812	0.002014	0.013263
Owenia_fusiformis	OFUSG15598.1	5694.832	-1.92395	0.211099	-9.14043	6.22E-20	3.06E-17
Owenia_fusiformis	OFUSG15606.1	68.55047	-2.06044	0.432697	-4.88017	1.06E-06	2.57E-05
Owenia_fusiformis	OFUSG15614.1	49.53937	-2.54481	0.447929	-5.2439	1.57E-07	5.03E-06
Owenia_fusiformis	OFUSG15623.1	1209.876	-1.33928	0.325171	-4.07244	4.65E-05	0.000642
Owenia_fusiformis	OFUSG15668.1	158.0294	-1.21021	0.312101	-3.85975	0.000114	0.001328
Owenia_fusiformis	OFUSG15671.1	85.15357	-1.17693	0.328438	-3.55054	0.000384	0.003567
Owenia_fusiformis	OFUSG15680.1	11.57122	-1.32034	0.440756	-3.62263	0.000292	0.002861
Owenia_fusiformis	OFUSG15681.1	40.10847	-1.15896	0.447717	-2.78943	0.00528	0.027946
Owenia_fusiformis	OFUSG15721.1	3844.443	-1.08151	0.159759	-6.77239	1.27E-11	1.25E-09
Owenia_fusiformis	OFUSG15736.1	255.9998	-2.05054	0.273772	-7.41062	1.26E-13	2.00E-11
Owenia_fusiformis	OFUSG15754.1	86.83128	-1.31084	0.381532	-3.30298	0.000957	0.007316
Owenia_fusiformis	OFUSG15778.1	90.0977	-2.40066	0.357358	-6.36546	1.95E-10	1.39E-08
Owenia_fusiformis	OFUSG15783.1	21.86766	-1.33052	0.447066	-3.10024	0.001934	0.012839
Owenia_fusiformis	OFUSG15799.1	78.06271	-1.07867	0.389643	-2.76306	0.005726	0.029745
Owenia_fusiformis	OFUSG15819.1	96.32019	-1.06669	0.321073	-3.31691	0.00091	0.007042
Owenia_fusiformis	OFUSG15881.3	90.57837	-1.04066	0.36428	-2.84995	0.004373	0.024182
Owenia_fusiformis	OFUSG15888.1	797.4923	-1.14577	0.217525	-5.26505	1.40E-07	4.54E-06
Owenia_fusiformis	OFUSG15949.1	3066.462	-1.22843	0.139955	-8.77522	1.71E-18	6.56E-16
Owenia_fusiformis	OFUSG15966.1	49.66465	-1.60979	0.426719	-3.82721	0.00013	0.001478
Owenia_fusiformis	OFUSG16016.1	156.4426	-1.00893	0.326736	-3.07136	0.002131	0.013839
Owenia_fusiformis	OFUSG16033.1	79.4402	-1.26947	0.337893	-3.73963	0.000184	0.001978
Owenia_fusiformis	OFUSG16034.1	1692.408	-1.46579	0.1341	-10.919	9.36E-28	9.22E-25

Owenia_fusiformis	OFUSG16044.1	24.43175	-1.11494	0.441747	-3.85203	0.000117	0.00136
Owenia_fusiformis	OFUSG16101.1	29.7168	-1.67079	0.446017	-3.84474	0.000121	0.001396
Owenia_fusiformis	OFUSG16106.1	31.50176	-1.66317	0.426786	-3.83738	0.000124	0.001432
Owenia_fusiformis	OFUSG16116.1	241.5821	-1.10054	0.354275	-3.08584	0.00203	0.013342
Owenia_fusiformis	OFUSG16127.1	34.68334	-1.32234	0.434492	-2.98927	0.002796	0.017021
Owenia_fusiformis	OFUSG16186.5	1402.141	-1.16607	0.326783	-3.52273	0.000427	0.003853
Owenia_fusiformis	OFUSG16220.1	215.4653	-1.00728	0.290857	-3.47145	0.000518	0.004503
Owenia_fusiformis	OFUSG16234.1	22.03508	-1.16889	0.444994	-2.95381	0.003139	0.018694
Owenia_fusiformis	OFUSG16276.1	252.3922	-2.32905	0.253294	-9.04593	1.48E-19	6.53E-17
Owenia_fusiformis	OFUSG16287.1	59.57254	-1.13662	0.356068	-3.18093	0.001468	0.010332
Owenia_fusiformis	OFUSG16297.1	89.33202	-1.53596	0.325274	-4.67379	2.96E-06	6.21E-05
Owenia_fusiformis	OFUSG16306.2	2780.805	-1.02517	0.235007	-4.35514	1.33E-05	0.000226
Owenia_fusiformis	OFUSG16318.1	179.325	-1.09466	0.304416	-3.55225	0.000382	0.003547
Owenia_fusiformis	OFUSG16343.3	159.3012	-1.59414	0.428344	-3.64337	0.000269	0.002678
Owenia_fusiformis	OFUSG16372.1	53.45448	-1.09698	0.396043	-2.77963	0.005442	0.02857
Owenia_fusiformis	OFUSG16373.1	69.53197	-1.9232	0.365173	-5.17047	2.34E-07	7.08E-06
Owenia_fusiformis	OFUSG16401.1	44.13826	-2.45163	0.446276	-5.10367	3.33E-07	9.50E-06
Owenia_fusiformis	OFUSG16402.1	23.31961	-1.52716	0.440934	-3.57085	0.000356	0.00335
Owenia_fusiformis	OFUSG16404.3	9.620542	-1.00432	0.400096	-2.94764	0.003202	0.018965
Owenia_fusiformis	OFUSG16406.2	119.1209	-1.18562	0.421756	-3.47945	0.000502	0.004394
Owenia_fusiformis	OFUSG16411.1	947.7672	-1.17564	0.18381	-6.42191	1.35E-10	9.98E-09
Owenia_fusiformis	OFUSG16413.1	10.37426	-1.32449	0.438138	-2.8394	0.00452	0.024829
Owenia_fusiformis	OFUSG16414.1	4428.552	-1.38658	0.176107	-7.87096	3.52E-15	8.25E-13
Owenia_fusiformis	OFUSG16436.1	14.43363	-1.21592	0.445536	-2.65566	0.007915	0.038057
Owenia_fusiformis	OFUSG16470.1	152.8472	-2.3509	0.299098	-7.73054	1.07E-14	2.22E-12
Owenia_fusiformis	OFUSG16476.3	21.76826	-1.70085	0.428349	-4.25627	2.08E-05	0.000328
Owenia_fusiformis	OFUSG16491.1	243.0862	-1.51931	0.276232	-5.47251	4.44E-08	1.70E-06
Owenia_fusiformis	OFUSG16505.1	27.78667	-1.68117	0.429141	-3.74329	0.000182	0.001952
Owenia_fusiformis	OFUSG16515.1	51.6486	-1.2947	0.448545	-2.70813	0.006766	0.033814
Owenia_fusiformis	OFUSG16546.1	44.00099	-1.47134	0.416158	-3.33254	0.000861	0.006755
Owenia_fusiformis	OFUSG16574.2	193.5447	-1.77616	0.43922	-4.22608	2.38E-05	0.000368
Owenia_fusiformis	OFUSG16584.1	53.25524	-1.44349	0.4301	-3.59205	0.000328	0.00314
Owenia_fusiformis	OFUSG16598.2	336.0428	-1.40629	0.310266	-4.53034	5.89E-06	0.000114
Owenia_fusiformis	OFUSG16599.1	97.39756	-1.70848	0.366078	-4.67999	2.87E-06	6.07E-05
Owenia_fusiformis	OFUSG16623.2	370.0488	-1.24031	0.376959	-3.23261	0.001227	0.00893
Owenia_fusiformis	OFUSG16657.1	79.36735	-1.94122	0.358285	-5.27335	1.34E-07	4.35E-06
Owenia_fusiformis	OFUSG16663.2	34.9649	-1.19048	0.447927	-3.87185	0.000108	0.001275
Owenia_fusiformis	OFUSG16683.1	175.5158	-1.51208	0.347452	-4.4246	9.66E-06	0.000173
Owenia_fusiformis	OFUSG16741.1	5.997795	-1.00194	0.417066	-2.99063	0.002784	0.016975
Owenia_fusiformis	OFUSG16797.1	100.6595	-1.51142	0.407751	-3.57945	0.000344	0.003266
Owenia_fusiformis	OFUSG16858.1	84.444	-1.62606	0.41577	-3.8503	0.000118	0.001368
Owenia_fusiformis	OFUSG16863.1	135.1531	-1.00612	0.310229	-3.2725	0.001066	0.00797
Owenia_fusiformis	OFUSG16866.2	126.5512	-2.42215	0.445954	-5.97881	2.25E-09	1.23E-07
Owenia_fusiformis	OFUSG16890.2	209.2933	-1.11785	0.445392	-3.27574	0.001054	0.007891
Owenia_fusiformis	OFUSG16931.1	383.2046	-1.12927	0.24921	-4.51304	6.39E-06	0.000122
Owenia_fusiformis	OFUSG16935.1	169.5456	-1.17729	0.308087	-3.80754	0.00014	0.001578
Owenia_fusiformis	OFUSG16943.1	115.7518	-1.10244	0.313293	-3.50862	0.00045	0.004013
Owenia_fusiformis	OFUSG16958.2	1878.249	-1.97833	0.215599	-9.1679	4.82E-20	2.47E-17
Owenia_fusiformis	OFUSG16982.1	113.7823	-1.20101	0.402731	-3.06524	0.002175	0.014051
Owenia_fusiformis	OFUSG17001.1	10.87484	-1.32575	0.44312	-2.79334	0.005217	0.027676
Owenia_fusiformis	OFUSG17008.1	48.85296	-1.92548	0.436613	-4.32092	1.55E-05	0.000257
Owenia_fusiformis	OFUSG17018.1	10.14247	-1.18356	0.436225	-3.31632	0.000912	0.007053
Owenia_fusiformis	OFUSG17028.2	53.37548	-1.52323	0.411665	-4.77336	1.81E-06	4.11E-05
Owenia_fusiformis	OFUSG17037.2	29.61417	-1.87079	0.441859	-4.4646	8.02E-06	0.000148
Owenia_fusiformis	OFUSG17051.1	97.42325	-1.45413	0.379311	-3.85981	0.000113	0.001328
Owenia_fusiformis	OFUSG17054.1	53.34326	-1.25379	0.446603	-3.39585	0.000684	0.005611
Owenia_fusiformis	OFUSG17056.1	208.037	-1.19315	0.304345	-3.99131	6.57E-05	0.000849
Owenia_fusiformis	OFUSG17070.1	11.33052	-1.22809	0.445884	-2.79429	0.005201	0.027618
Owenia_fusiformis	OFUSG17127.1	473.2915	-1.48224	0.256632	-5.77745	7.58E-09	3.59E-07
Owenia_fusiformis	OFUSG17133.2	1202.559	-1.02	0.247318	-4.1378	3.51E-05	0.000508
Owenia_fusiformis	OFUSG17166.1	193.5448	-1.02792	0.420608	-2.56376	0.010354	0.046782
Owenia_fusiformis	OFUSG17166.4	294.4107	-1.69445	0.421779	-3.82921	0.000129	0.001471
Owenia_fusiformis	OFUSG17181.1	50.72241	-1.262	0.407902	-3.14009	0.001689	0.011603
Owenia_fusiformis	OFUSG17192.1	131.7391	-1.42118	0.349921	-4.03384	5.49E-05	0.000732
Owenia_fusiformis	OFUSG17230.1	117.9531	-1.75629	0.343275	-5.02794	4.96E-07	1.33E-05
Owenia_fusiformis	OFUSG17240.1	3305.675	-1.11981	0.14575	-7.68156	1.57E-14	3.10E-12
Owenia_fusiformis	OFUSG17247.1	703.0571	-1.37693	0.155259	-8.86128	7.91E-19	3.19E-16
Owenia_fusiformis	OFUSG17309.1	400.4568	-1.05102	0.222354	-4.71226	2.45E-06	5.33E-05
Owenia_fusiformis	OFUSG17351.2	130.8193	-1.30987	0.373608	-3.47067	0.000519	0.004511
Owenia_fusiformis	OFUSG17403.1	632.7976	-1.09766	0.239984	-4.61375	3.95E-06	8.05E-05
Owenia_fusiformis	OFUSG17444.1	41.19581	-2.54206	0.446402	-5.31709	1.05E-07	3.54E-06
Owenia_fusiformis	OFUSG17445.1	10063.26	-2.51326	0.286248	-8.80999	1.25E-18	4.97E-16
Owenia_fusiformis	OFUSG17472.1	235.4555	-1.54019	0.233378	-6.5716	4.98E-11	4.17E-09
Owenia_fusiformis	OFUSG17486.1	149.1404	-1.50854	0.301038	-4.93908	7.85E-07	1.98E-05
Owenia_fusiformis	OFUSG17493.1	8.915459	-1.26161	0.422101	-3.48068	0.0005	0.004378
Owenia_fusiformis	OFUSG17526.1	15.84992	-1.00238	0.439944	-2.69995	0.006935	0.034454
Owenia_fusiformis	OFUSG17531.3	57.32932	-1.15458	0.398358	-2.96637	0.003013	0.018096
Owenia_fusiformis	OFUSG17583.1	190.4024	-1.50843	0.400854	-4.12166	3.76E-05	0.000539
Owenia_fusiformis	OFUSG17605.1	26.99853	-1.19432	0.448021	-3.03719	0.002388	0.015125
Owenia_fusiformis	OFUSG17640.1	147.6305	-1.41552	0.417695	-3.51112	0.000446	0.003983

Owenia_fusiformis	OFUSG17644.1	18.09481	-1.84325	0.438059	-3.51138	0.000446	0.003981
Owenia_fusiformis	OFUSG17646.1	210.7524	-1.19627	0.37862	-3.13342	0.001728	0.011807
Owenia_fusiformis	OFUSG17690.1	1430.953	-1.0773	0.201356	-5.34941	8.82E-08	3.05E-06
Owenia_fusiformis	OFUSG17727.1	109.9867	-3.11646	0.388465	-7.58725	3.27E-14	5.92E-12
Owenia_fusiformis	OFUSG17729.1	706.853	-2.09256	0.184648	-11.3059	1.23E-29	1.51E-26
Owenia_fusiformis	OFUSG17738.1	18.43507	-1.37473	0.443138	-3.18857	0.00143	0.010101
Owenia_fusiformis	OFUSG17825.1	339.5321	-3.82386	0.382981	-6.69929	2.09E-11	1.93E-09
Owenia_fusiformis	OFUSG17894.1	1264.11	-1.4378	0.274933	-5.22124	1.78E-07	5.60E-06
Owenia_fusiformis	OFUSG17894.3	556.9719	-1.04506	0.347587	-2.99504	0.002744	0.016794
Owenia_fusiformis	OFUSG17925.1	25.20228	-2.0287	0.447719	-3.7031	0.000213	0.002223
Owenia_fusiformis	OFUSG17934.1	8.418092	-1.27641	0.40589	-3.37422	0.00074	0.005975
Owenia_fusiformis	OFUSG17967.1	41.00022	-1.24241	0.436087	-2.73163	0.006302	0.032087
Owenia_fusiformis	OFUSG17995.1	37.74785	-1.33845	0.421763	-3.25156	0.001148	0.008458
Owenia_fusiformis	OFUSG18002.1	142.7498	-1.43187	0.326677	-4.38734	1.15E-05	0.000199
Owenia_fusiformis	OFUSG18007.1	725.2814	-1.55888	0.288287	-5.39712	6.77E-08	2.44E-06
Owenia_fusiformis	OFUSG18051.1	42.54156	-1.09349	0.391237	-2.81212	0.004922	0.02647
Owenia_fusiformis	OFUSG18065.1	23.72632	-1.28625	0.443536	-3.73863	0.000185	0.001983
Owenia_fusiformis	OFUSG18066.1	80.89221	-1.55142	0.435753	-3.6225	0.000292	0.002862
Owenia_fusiformis	OFUSG18097.1	20.54784	-1.17386	0.445901	-2.73885	0.006166	0.031463
Owenia_fusiformis	OFUSG18102.1	308.5795	-1.60593	0.230321	-6.9396	3.93E-12	4.40E-10
Owenia_fusiformis	OFUSG18103.1	103.0036	-1.09347	0.310508	-3.52254	0.000427	0.003854
Owenia_fusiformis	OFUSG18109.1	717.196	-1.06277	0.260177	-4.08933	4.33E-05	0.000605
Owenia_fusiformis	OFUSG18134.1	36.36696	-1.42083	0.418729	-4.31542	1.59E-05	0.000262
Owenia_fusiformis	OFUSG18136.1	15.17074	-1.68917	0.44706	-3.55666	0.000376	0.003495
Owenia_fusiformis	OFUSG18151.3	444.9818	-1.02703	0.203965	-5.03937	4.67E-07	1.27E-05
Owenia_fusiformis	OFUSG18174.1	140.6061	-1.06473	0.267623	-3.96937	7.21E-05	0.000918
Owenia_fusiformis	OFUSG18179.2	19.99433	-1.11203	0.39371	-3.74641	0.000179	0.001929
Owenia_fusiformis	OFUSG18183.1	400.1272	-1.2179	0.385656	-3.08176	0.002058	0.013478
Owenia_fusiformis	OFUSG18188.1	180.6129	-1.43764	0.367505	-3.94674	7.92E-05	0.000992
Owenia_fusiformis	OFUSG18202.1	116.8347	-1.01179	0.3677	-2.66947	0.007597	0.036953
Owenia_fusiformis	OFUSG18215.1	25.99597	-1.19864	0.442774	-2.6771	0.007426	0.036359
Owenia_fusiformis	OFUSG18239.1	30.16903	-1.40406	0.402703	-3.38336	0.000716	0.005822
Owenia_fusiformis	OFUSG18240.1	112.6844	-1.92204	0.335989	-5.60064	2.14E-08	8.90E-07
Owenia_fusiformis	OFUSG18241.1	1127.322	-1.03127	0.239038	-4.31448	1.60E-05	0.000263
Owenia_fusiformis	OFUSG18254.1	170.7697	-1.66255	0.269717	-6.13327	8.61E-10	5.28E-08
Owenia_fusiformis	OFUSG18266.1	93.7822	-1.01003	0.320681	-3.10543	0.0019	0.012657
Owenia_fusiformis	OFUSG18275.1	999.8778	-1.89706	0.306046	-6.1871	6.13E-10	3.94E-08
Owenia_fusiformis	OFUSG18276.1	14.97539	-1.14626	0.424993	-3.6055	0.000312	0.003018
Owenia_fusiformis	OFUSG18291.1	712.1616	-1.11895	0.164739	-6.79302	1.10E-11	1.10E-09
Owenia_fusiformis	OFUSG18304.1	63.43661	-1.80098	0.419025	-4.32775	1.51E-05	0.00025
Owenia_fusiformis	OFUSG18363.1	802.711	-1.13458	0.154622	-7.3402	2.13E-13	3.26E-11
Owenia_fusiformis	OFUSG18370.1	21.99405	-1.46765	0.447331	-3.55387	0.00038	0.003528
Owenia_fusiformis	OFUSG18428.1	155.4788	-1.07211	0.34169	-3.00463	0.002659	0.016383
Owenia_fusiformis	OFUSG18430.1	28.26515	-1.39984	0.442709	-3.44277	0.000576	0.004882
Owenia_fusiformis	OFUSG18458.1	33.10266	-1.55352	0.439967	-3.28387	0.001024	0.007728
Owenia_fusiformis	OFUSG18502.1	23.1508	-1.87166	0.446131	-3.85773	0.000114	0.001337
Owenia_fusiformis	OFUSG18519.1	72.75942	-1.76059	0.394389	-4.60776	4.07E-06	8.26E-05
Owenia_fusiformis	OFUSG18531.4	35.69699	-1.01739	0.447601	-3.66368	0.000249	0.002513
Owenia_fusiformis	OFUSG18533.1	18.69139	-1.12447	0.44553	-2.77765	0.005475	0.028696
Owenia_fusiformis	OFUSG18537.1	822.2089	-1.01435	0.178311	-5.68404	1.32E-08	5.85E-07
Owenia_fusiformis	OFUSG18548.1	624.9013	-2.00676	0.202179	-9.89783	4.25E-23	3.27E-20
Owenia_fusiformis	OFUSG18550.1	34.74814	-1.67349	0.43868	-3.33265	0.00086	0.006755
Owenia_fusiformis	OFUSG18589.1	288.845	-1.88564	0.294682	-6.40223	1.53E-10	1.12E-08
Owenia_fusiformis	OFUSG18593.1	94.0616	-1.83894	0.365205	-4.89175	9.99E-07	2.44E-05
Owenia_fusiformis	OFUSG18602.1	124.0814	-1.57457	0.294036	-5.34687	8.95E-08	3.08E-06
Owenia_fusiformis	OFUSG18623.1	115.5133	-1.89697	0.315824	-5.94156	2.82E-09	1.51E-07
Owenia_fusiformis	OFUSG18667.1	345.7595	-1.7072	0.238656	-7.13791	9.48E-13	1.25E-10
Owenia_fusiformis	OFUSG18682.1	29.45834	-1.41557	0.420983	-4.26026	2.04E-05	0.000323
Owenia_fusiformis	OFUSG18705.1	38.78868	-1.87637	0.415037	-4.4663	7.96E-06	0.000147
Owenia_fusiformis	OFUSG18709.2	16.06803	-1.25172	0.441125	-3.65064	0.000262	0.002618
Owenia_fusiformis	OFUSG18759.1	36.91435	-1.21137	0.429809	-2.70939	0.006741	0.033727
Owenia_fusiformis	OFUSG18809.1	1565.035	-1.73301	0.30768	-5.62353	1.87E-08	7.94E-07
Owenia_fusiformis	OFUSG18819.1	11.88813	-1.11183	0.439685	-2.66837	0.007622	0.037035
Owenia_fusiformis	OFUSG18820.1	4924.371	-2.6622	0.255614	-10.4119	2.19E-25	2.00E-22
Owenia_fusiformis	OFUSG18821.1	26.25584	-1.47621	0.442003	-3.71344	0.000204	0.002149
Owenia_fusiformis	OFUSG18899.1	79.75196	-1.03419	0.440936	-2.76702	0.005657	0.029454
Owenia_fusiformis	OFUSG18902.1	32.59963	-1.10264	0.421867	-2.66611	0.007673	0.037218
Owenia_fusiformis	OFUSG18925.1	8.517315	-1.20571	0.43444	-2.72634	0.006404	0.032512
Owenia_fusiformis	OFUSG18929.1	38.12553	-1.09242	0.448126	-2.53679	0.011187	0.04966
Owenia_fusiformis	OFUSG18931.1	64.14079	-1.22817	0.415538	-2.91514	0.003555	0.020577
Owenia_fusiformis	OFUSG18950.1	37.92584	-1.00856	0.393498	-2.58969	0.009606	0.044095
Owenia_fusiformis	OFUSG18989.1	49.17251	-2.00811	0.444244	-4.24854	2.15E-05	0.000338
Owenia_fusiformis	OFUSG19000.1	531.9025	-1.45286	0.230014	-6.31637	2.68E-10	1.82E-08
Owenia_fusiformis	OFUSG19022.1	21.08506	-1.39273	0.438527	-2.98235	0.00286	0.017333
Owenia_fusiformis	OFUSG19035.1	60.8575	-1.16328	0.42434	-3.56993	0.000357	0.003357
Owenia_fusiformis	OFUSG19054.1	8.547413	-1.12253	0.434193	-2.53768	0.011159	0.04956
Owenia_fusiformis	OFUSG19065.1	22.26867	-1.27951	0.423832	-3.14006	0.001689	0.011603
Owenia_fusiformis	OFUSG19069.1	1043.295	-1.50945	0.360943	-4.1605	3.18E-05	0.000468
Owenia_fusiformis	OFUSG19090.1	55.6132	-2.70563	0.424491	-5.78691	7.17E-09	3.43E-07
Owenia_fusiformis	OFUSG19104.3	75.89636	-1.06445	0.38464	-2.60755	0.009119	0.042373

Owenia_fusiformis	OFUSG19126.1	1623.698	-1.20608	0.179153	-6.73108	1.68E-11	1.60E-09
Owenia_fusiformis	OFUSG19152.3	10.57152	-1.21992	0.423813	-3.34593	0.00082	0.0065
Owenia_fusiformis	OFUSG19167.1	1119.058	-1.62558	0.44811	-3.46986	0.000521	0.004522
Owenia_fusiformis	OFUSG19202.1	144.9457	-1.0301	0.267538	-3.83043	0.000128	0.001466
Owenia_fusiformis	OFUSG19203.1	429.7694	-1.07082	0.267178	-4.00063	6.32E-05	0.000821
Owenia_fusiformis	OFUSG19204.1	70.80323	-1.12739	0.373781	-2.98517	0.002834	0.017205
Owenia_fusiformis	OFUSG19230.1	41.30565	-1.82321	0.395184	-4.3401	1.42E-05	0.000239
Owenia_fusiformis	OFUSG19280.1	1174.633	-1.60785	0.233818	-6.9345	4.08E-12	4.54E-10
Owenia_fusiformis	OFUSG19289.1	158.0241	-1.00095	0.290448	-3.43845	0.000585	0.004941
Owenia_fusiformis	OFUSG19327.1	54.69493	-1.28476	0.441201	-2.69183	0.007106	0.035127
Owenia_fusiformis	OFUSG19347.1	241.0619	-1.05357	0.273963	-3.81801	0.000135	0.001524
Owenia_fusiformis	OFUSG19357.1	7.822305	-1.04641	0.431152	-3.08404	0.002042	0.013407
Owenia_fusiformis	OFUSG19360.1	1483.878	-1.00797	0.14311	-7.04838	1.81E-12	2.21E-10
Owenia_fusiformis	OFUSG19367.1	1493.331	-1.0138	0.214165	-4.72516	2.30E-06	5.05E-05
Owenia_fusiformis	OFUSG19367.2	1144.39	-1.68645	0.292044	-5.74552	9.16E-09	4.26E-07
Owenia_fusiformis	OFUSG19379.2	144.1612	-1.31376	0.281353	-4.63085	3.64E-06	7.47E-05
Owenia_fusiformis	OFUSG19411.1	207.5018	-1.11279	0.235848	-4.67787	2.90E-06	6.12E-05
Owenia_fusiformis	OFUSG19432.1	18.94828	-1.19039	0.444858	-2.8457	0.004431	0.024453
Owenia_fusiformis	OFUSG19469.1	474.4243	-1.15803	0.389867	-2.90298	0.003696	0.021204
Owenia_fusiformis	OFUSG19485.1	118.9717	-1.09871	0.315938	-3.44133	0.000579	0.004906
Owenia_fusiformis	OFUSG19487.1	523.7626	-1.48382	0.217576	-6.80572	1.01E-11	1.02E-09
Owenia_fusiformis	OFUSG19523.1	2531.891	-1.17376	0.183179	-6.41017	1.45E-10	1.07E-08
Owenia_fusiformis	OFUSG19563.1	300.999	-1.21916	0.220777	-5.50298	3.73E-08	1.46E-06
Owenia_fusiformis	OFUSG19590.1	1357.154	-1.23399	0.182331	-6.76636	1.32E-11	1.30E-09
Owenia_fusiformis	OFUSG19609.1	2088.456	-1.74146	0.418445	-4.02776	5.63E-05	0.000748
Owenia_fusiformis	OFUSG19623.1	255.0006	-3.14752	0.396869	-7.69237	1.44E-14	2.88E-12
Owenia_fusiformis	OFUSG19639.1	11.27511	-1.19164	0.429773	-3.39818	0.000678	0.005578
Owenia_fusiformis	OFUSG19659.1	137.1092	-1.67407	0.444703	-4.26388	2.01E-05	0.000319
Owenia_fusiformis	OFUSG19675.3	109.1139	-2.0079	0.38985	-5.18539	2.16E-07	6.58E-06
Owenia_fusiformis	OFUSG19707.1	735.2171	-1.51146	0.314381	-4.81308	1.49E-06	3.46E-05
Owenia_fusiformis	OFUSG19708.3	55.56561	-2.43849	0.432212	-5.46612	4.60E-08	1.76E-06
Owenia_fusiformis	OFUSG19709.1	101.6458	-1.55475	0.329499	-4.72253	2.33E-06	5.10E-05
Owenia_fusiformis	OFUSG19722.1	62.82838	-1.29974	0.373023	-3.43285	0.000597	0.005025
Owenia_fusiformis	OFUSG19734.2	226.4865	-1.68145	0.441327	-3.59971	0.000319	0.003071
Owenia_fusiformis	OFUSG19737.1	20.36271	-1.48594	0.443635	-3.31705	0.00091	0.007042
Owenia_fusiformis	OFUSG19753.1	140.9312	-1.4971	0.448641	-3.7354	0.000187	0.002004
Owenia_fusiformis	OFUSG19767.2	439.7984	-1.17373	0.317758	-3.74846	0.000178	0.001918
Owenia_fusiformis	OFUSG19770.1	1338.735	-1.15194	0.221268	-5.21368	1.85E-07	5.77E-06
Owenia_fusiformis	OFUSG19784.1	10.89879	-1.07062	0.409973	-3.30743	0.000942	0.007235
Owenia_fusiformis	OFUSG19863.1	159.574	-1.35034	0.361898	-3.8833	0.000103	0.001227
Owenia_fusiformis	OFUSG19954.1	191.9932	-1.12259	0.423784	-2.98368	0.002848	0.017276
Owenia_fusiformis	OFUSG19961.1	41.37949	-1.48202	0.436034	-3.45869	0.000543	0.004671
Owenia_fusiformis	OFUSG20004.1	119.5003	-1.30015	0.275449	-4.71653	2.40E-06	5.23E-05
Owenia_fusiformis	OFUSG20015.1	6.422658	-1.20139	0.383702	-2.55306	0.010678	0.047847
Owenia_fusiformis	OFUSG20018.2	219.9747	-1.81735	0.442644	-5.8231	5.78E-09	2.82E-07
Owenia_fusiformis	OFUSG20067.1	77.45913	-2.27535	0.407553	-5.55346	2.80E-08	1.14E-06
Owenia_fusiformis	OFUSG20069.1	9.862695	-1.55089	0.437114	-3.01696	0.002553	0.015883
Owenia_fusiformis	OFUSG20078.2	470.1671	-1.30898	0.321329	-3.99651	6.43E-05	0.000832
Owenia_fusiformis	OFUSG20079.1	151.1358	-1.46659	0.337648	-4.28334	1.84E-05	0.000295
Owenia_fusiformis	OFUSG20123.1	80.81756	-1.5596	0.393496	-3.93304	8.39E-05	0.001039
Owenia_fusiformis	OFUSG20125.1	162.2942	-1.0846	0.289951	-3.73172	0.00019	0.002025
Owenia_fusiformis	OFUSG20167.1	329.4656	-1.17265	0.376221	-3.28046	0.001036	0.007798
Owenia_fusiformis	OFUSG20236.1	190.9848	-1.11319	0.380194	-2.785	0.005353	0.028179
Owenia_fusiformis	OFUSG20299.1	4099.947	-2.21332	0.375699	-5.91006	3.42E-09	1.78E-07
Owenia_fusiformis	OFUSG20363.1	33.57499	-1.47092	0.44767	-3.35887	0.000783	0.006254
Owenia_fusiformis	OFUSG20368.1	24.37296	-2.01193	0.443271	-4.47603	7.60E-06	0.000141
Owenia_fusiformis	OFUSG20399.1	58.80063	-2.14781	0.380672	-5.42459	5.81E-08	2.14E-06
Owenia_fusiformis	OFUSG20428.1	78.96164	-1.96889	0.335665	-5.69127	1.26E-08	5.65E-07
Owenia_fusiformis	OFUSG20429.1	697.7279	-1.46921	0.193713	-7.58548	3.31E-14	5.95E-12
Owenia_fusiformis	OFUSG20451.1	8.488839	-1.31324	0.392146	-3.30952	0.000935	0.00719
Owenia_fusiformis	OFUSG20456.1	168.0402	-1.32523	0.285409	-4.61157	4.00E-06	8.11E-05
Owenia_fusiformis	OFUSG20483.1	45.25647	-1.15552	0.448451	-3.07736	0.002088	0.013639
Owenia_fusiformis	OFUSG20505.2	141.8824	-1.40932	0.315569	-4.42478	9.65E-06	0.000173
Owenia_fusiformis	OFUSG20508.1	1138.034	-1.25492	0.297868	-4.19652	2.71E-05	0.00041
Owenia_fusiformis	OFUSG20512.1	929.5719	-1.53342	0.362931	-4.14695	3.37E-05	0.000491
Owenia_fusiformis	OFUSG20535.1	303.3252	-1.10954	0.358042	-3.06914	0.002147	0.013909
Owenia_fusiformis	OFUSG20556.1	31.22175	-1.03783	0.441501	-2.55581	0.010594	0.047557
Owenia_fusiformis	OFUSG20557.1	23.15365	-1.77824	0.443927	-3.70286	0.000213	0.002225
Owenia_fusiformis	OFUSG20561.1	1510.166	-1.21475	0.282624	-4.29147	1.77E-05	0.000286
Owenia_fusiformis	OFUSG20588.1	341.883	-1.7336	0.267312	-6.46927	9.85E-11	7.65E-09
Owenia_fusiformis	OFUSG20624.2	278.6603	-1.23613	0.416767	-3.03589	0.002398	0.015175
Owenia_fusiformis	OFUSG20633.1	2767.699	-1.29233	0.228339	-5.64586	1.64E-08	7.08E-07
Owenia_fusiformis	OFUSG20640.1	37.07558	-1.83038	0.438216	-4.02325	5.74E-05	0.000758
Owenia_fusiformis	OFUSG20644.1	20.35486	-1.8989	0.437115	-3.78676	0.000153	0.001693
Owenia_fusiformis	OFUSG20665.1	1519.359	-1.33801	0.288578	-4.63635	3.55E-06	7.30E-05
Owenia_fusiformis	OFUSG20707.1	20.85065	-1.28503	0.444582	-2.99174	0.002774	0.016926
Owenia_fusiformis	OFUSG20712.1	470.2433	-1.03734	0.275983	-3.73574	0.000187	0.002002
Owenia_fusiformis	OFUSG20718.1	938.4095	-2.39498	0.301637	-8.08687	6.12E-16	1.66E-13
Owenia_fusiformis	OFUSG20720.1	39.1907	-1.91432	0.440838	-3.91023	9.22E-05	0.001118
Owenia_fusiformis	OFUSG20751.1	669.4646	-1.13707	0.204271	-5.56527	2.62E-08	1.07E-06

Owenia_fusiformis	OFUSG20764.1	608.7846	-1.09993	0.233294	-4.70389	2.55E-06	5.52E-05
Owenia_fusiformis	OFUSG20770.2	132.3914	-1.65075	0.365521	-4.42422	9.68E-06	0.000173
Owenia_fusiformis	OFUSG20790.1	36.74117	-1.06952	0.381314	-2.76409	0.005708	0.029663
Owenia_fusiformis	OFUSG20821.1	109.8096	-1.36612	0.393839	-3.49841	0.000468	0.004148
Owenia_fusiformis	OFUSG20832.1	524.2792	-1.84521	0.376238	-4.82324	1.41E-06	3.30E-05
Owenia_fusiformis	OFUSG20834.1	815.1875	-1.52803	0.257245	-5.94683	2.73E-09	1.47E-07
Owenia_fusiformis	OFUSG20835.1	38.04559	-1.34909	0.447745	-3.59332	0.000326	0.003129
Owenia_fusiformis	OFUSG20836.1	50.74247	-1.34198	0.432445	-3.11733	0.001825	0.012293
Owenia_fusiformis	OFUSG20844.1	237.5112	-2.35721	0.351238	-6.67966	2.39E-11	2.18E-09
Owenia_fusiformis	OFUSG20856.4	71.10048	-2.33226	0.429959	-5.1551	2.53E-07	7.58E-06
Owenia_fusiformis	OFUSG20901.2	746.9327	-1.10759	0.185934	-5.96052	2.51E-09	1.37E-07
Owenia_fusiformis	OFUSG20914.1	17.00512	-1.15639	0.442184	-2.55881	0.010503	0.047261
Owenia_fusiformis	OFUSG20914.3	17.00512	-1.15639	0.442184	-2.55881	0.010503	0.047261
Owenia_fusiformis	OFUSG21000.3	4372.762	-1.08963	0.300321	-3.60955	0.000307	0.002981
Owenia_fusiformis	OFUSG21020.2	28.24655	-1.12142	0.446172	-2.7056	0.006818	0.034004
Owenia_fusiformis	OFUSG21022.2	294.5709	-1.1752	0.307534	-3.79683	0.000147	0.001635
Owenia_fusiformis	OFUSG21041.1	67.55982	-1.44702	0.396045	-3.66524	0.000247	0.002506
Owenia_fusiformis	OFUSG21042.1	14.43497	-1.09908	0.446613	-2.70395	0.006852	0.03411
Owenia_fusiformis	OFUSG21045.1	4819.518	-1.34234	0.173545	-7.73098	1.07E-14	2.22E-12
Owenia_fusiformis	OFUSG21100.1	3021.826	-1.78125	0.139917	-12.7303	4.01E-37	8.24E-34
Owenia_fusiformis	OFUSG21124.1	12.88699	-1.50198	0.443505	-3.3512	0.000805	0.006401
Owenia_fusiformis	OFUSG21157.1	543.2381	-1.35204	0.370976	-3.81557	0.000136	0.001537
Owenia_fusiformis	OFUSG21210.1	4084.05	-1.22144	0.16379	-7.45519	8.97E-14	1.46E-11
Owenia_fusiformis	OFUSG21217.1	21.71511	-1.12611	0.443482	-2.73018	0.00633	0.032215
Owenia_fusiformis	OFUSG21220.1	54.77726	-1.01921	0.446668	-2.8037	0.005052	0.027024
Owenia_fusiformis	OFUSG21224.1	37.32081	-1.3366	0.391465	-3.36443	0.000767	0.006153
Owenia_fusiformis	OFUSG21229.3	19.3102	-1.6493	0.443358	-3.5225	0.000428	0.003854
Owenia_fusiformis	OFUSG21236.1	40.79227	-1.38553	0.447907	-4.12468	3.71E-05	0.000533
Owenia_fusiformis	OFUSG21279.1	48.72027	-3.07575	0.409271	-5.36081	8.29E-08	2.90E-06
Owenia_fusiformis	OFUSG21282.3	100.0249	-1.52103	0.337521	-4.44516	8.78E-06	0.00016
Owenia_fusiformis	OFUSG21300.1	5879.874	-1.04574	0.200029	-5.22249	1.77E-07	5.57E-06
Owenia_fusiformis	OFUSG21342.1	1833.042	-2.07606	0.223503	-9.28239	1.66E-20	9.72E-18
Owenia_fusiformis	OFUSG21349.1	445.6756	-1.05198	0.188895	-5.55881	2.72E-08	1.11E-06
Owenia_fusiformis	OFUSG21402.1	2878.651	-1.05757	0.091785	-11.5247	9.90E-31	1.43E-27
Owenia_fusiformis	OFUSG21454.1	42.66632	-1.14664	0.41347	-2.91352	0.003574	0.02067
Owenia_fusiformis	OFUSG21473.1	549.2814	-2.03087	0.241584	-8.38721	4.98E-17	1.59E-14
Owenia_fusiformis	OFUSG21473.2	675.3808	-1.09705	0.271507	-4.04075	5.33E-05	0.000716
Owenia_fusiformis	OFUSG21484.1	10372.15	-1.4012	0.198676	-7.05097	1.78E-12	2.18E-10
Owenia_fusiformis	OFUSG21527.1	4008.546	-1.40297	0.2302	-6.09204	1.11E-09	6.62E-08
Owenia_fusiformis	OFUSG21545.1	1466.85	-1.15738	0.230126	-5.03069	4.89E-07	1.31E-05
Owenia_fusiformis	OFUSG21581.2	322.447	-1.094	0.323252	-3.40265	0.000667	0.005504
Owenia_fusiformis	OFUSG21583.1	82.1701	-1.53787	0.397017	-3.79958	0.000145	0.001622
Owenia_fusiformis	OFUSG21584.1	11.13124	-1.01022	0.425569	-3.3565	0.000789	0.006299
Owenia_fusiformis	OFUSG21588.1	16.6183	-1.74079	0.428926	-4.16072	3.17E-05	0.000468
Owenia_fusiformis	OFUSG21589.1	47.49078	-3.02736	0.445426	-5.58617	2.32E-08	9.59E-07
Owenia_fusiformis	OFUSG21596.1	21.04674	-1.3838	0.445076	-3.07296	0.002119	0.013777
Owenia_fusiformis	OFUSG21668.1	61.04783	-1.12181	0.365078	-3.05269	0.002268	0.014507
Owenia_fusiformis	OFUSG21678.1	168.6466	-1.21961	0.236469	-5.1414	2.73E-07	8.08E-06
Owenia_fusiformis	OFUSG21700.1	35.06694	-1.1614	0.428194	-2.84684	0.004416	0.024382
Owenia_fusiformis	OFUSG21730.2	953.6752	-1.01036	0.357643	-2.82043	0.004796	0.025936
Owenia_fusiformis	OFUSG21746.1	28.67871	-1.19227	0.447956	-2.71981	0.006532	0.032936
Owenia_fusiformis	OFUSG21748.3	140.8218	-2.16224	0.279312	-7.60392	2.87E-14	5.28E-12
Owenia_fusiformis	OFUSG21792.1	18.96668	-2.08906	0.442161	-4.38474	1.16E-05	0.000201
Owenia_fusiformis	OFUSG21806.1	487.7784	-1.57853	0.442073	-3.13362	0.001727	0.011802
Owenia_fusiformis	OFUSG21850.1	373.5224	-1.33193	0.231812	-5.74272	9.32E-09	4.31E-07
Owenia_fusiformis	OFUSG21850.2	103.5629	-1.45673	0.441412	-3.91593	9.01E-05	0.001098
Owenia_fusiformis	OFUSG21903.1	18.32263	-1.3192	0.446515	-3.04672	0.002314	0.014747
Owenia_fusiformis	OFUSG21953.1	41.46235	-1.20304	0.413147	-3.14295	0.001673	0.011504
Owenia_fusiformis	OFUSG21984.1	20.73093	-2.14461	0.446171	-4.56094	5.09E-06	0.0001
Owenia_fusiformis	OFUSG22046.1	56.02635	-1.02339	0.386286	-2.59363	0.009497	0.043732
Owenia_fusiformis	OFUSG22068.1	20.75423	-2.07743	0.443685	-4.42642	9.58E-06	0.000172
Owenia_fusiformis	OFUSG22193.1	45.8021	-1.5287	0.444572	-3.5712	0.000355	0.003347
Owenia_fusiformis	OFUSG22210.1	87.69738	-1.1874	0.346431	-3.46356	0.000533	0.004601
Owenia_fusiformis	OFUSG22241.1	8.681945	-1.18233	0.418581	-3.31145	0.000928	0.007147
Owenia_fusiformis	OFUSG22283.1	1882.728	-1.0415	0.221378	-4.70082	2.59E-06	5.57E-05
Owenia_fusiformis	OFUSG22283.2	202.663	-1.45148	0.290301	-4.98814	6.10E-07	1.59E-05
Owenia_fusiformis	OFUSG22295.1	814.7027	-1.03231	0.226597	-4.55196	5.31E-06	0.000104
Owenia_fusiformis	OFUSG22317.2	148.3364	-2.78518	0.412369	-5.77031	7.91E-09	3.73E-07
Owenia_fusiformis	OFUSG22325.1	433.1975	-1.25989	0.285889	-4.46045	8.18E-06	0.00015
Owenia_fusiformis	OFUSG22325.2	247.6685	-1.18427	0.293568	-4.07436	4.61E-05	0.000638
Owenia_fusiformis	OFUSG22344.1	185.6424	-1.06183	0.256781	-4.10101	4.11E-05	0.00058
Owenia_fusiformis	OFUSG22350.1	51.184	-1.37128	0.433616	-3.13116	0.001741	0.011878
Owenia_fusiformis	OFUSG22377.1	23.91575	-1.69959	0.432732	-3.5467	0.00039	0.003601
Owenia_fusiformis	OFUSG22434.1	68.52428	-1.50856	0.424449	-3.49966	0.000466	0.004133
Owenia_fusiformis	OFUSG22460.1	213.9548	-1.16597	0.297272	-3.90259	9.52E-05	0.001146
Owenia_fusiformis	OFUSG22461.1	236.9521	-1.10982	0.267375	-4.13772	3.51E-05	0.000508
Owenia_fusiformis	OFUSG22465.1	44.89523	-1.10838	0.424419	-2.61466	0.008932	0.041628
Owenia_fusiformis	OFUSG22503.1	1517.795	-1.24508	0.152729	-8.15718	3.43E-16	9.60E-14
Owenia_fusiformis	OFUSG22504.1	483.0727	-1.70593	0.43046	-4.22326	2.41E-05	0.000372
Owenia_fusiformis	OFUSG22615.1	92.56045	-1.39104	0.436989	-3.03786	0.002383	0.015096

Owenia_fusiformis	OFUSG22647.1	1253.973	-1.62363	0.176624	-9.17775	4.40E-20	2.31E-17
Owenia_fusiformis	OFUSG22648.1	1108.042	-2.12007	0.162151	-13.0533	6.08E-39	1.36E-35
Owenia_fusiformis	OFUSG22694.1	44.89991	-1.01634	0.405586	-2.79715	0.005156	0.027464
Owenia_fusiformis	OFUSG22703.1	342.9878	-1.25361	0.215933	-5.81233	6.16E-09	2.99E-07
Owenia_fusiformis	OFUSG22710.1	13.78099	-1.39335	0.433229	-3.16636	0.001544	0.010778
Owenia_fusiformis	OFUSG22719.1	64.7046	-2.30195	0.418028	-5.43536	5.47E-08	2.03E-06
Owenia_fusiformis	OFUSG22723.1	230.6009	-2.23783	0.348494	-6.41639	1.40E-10	1.03E-08
Owenia_fusiformis	OFUSG22744.1	20.38372	-1.37824	0.446278	-2.94952	0.003183	0.018896
Owenia_fusiformis	OFUSG22789.1	24.49373	-1.2387	0.420938	-2.90607	0.00366	0.021045
Owenia_fusiformis	OFUSG22815.1	608.7862	-1.82825	0.279865	-6.50672	7.68E-11	6.12E-09
Owenia_fusiformis	OFUSG22816.1	455.0996	-1.75775	0.333729	-5.19738	2.02E-07	6.22E-06
Owenia_fusiformis	OFUSG22857.1	43.55162	-1.55458	0.441087	-3.73267	0.000189	0.002021
Owenia_fusiformis	OFUSG22887.1	64.93471	-1.18688	0.350815	-3.34438	0.000825	0.006528
Owenia_fusiformis	OFUSG22888.1	78.99309	-1.10699	0.308158	-3.5839	0.000338	0.003224
Owenia_fusiformis	OFUSG22943.2	18.87898	-2.18329	0.443583	-4.09235	4.27E-05	0.0006
Owenia_fusiformis	OFUSG23017.1	21.6709	-1.86206	0.399294	-3.58011	0.000343	0.003261
Owenia_fusiformis	OFUSG23061.1	15.86655	-1.46036	0.447393	-3.00425	0.002662	0.0164
Owenia_fusiformis	OFUSG23070.1	683.7097	-1.34986	0.276834	-4.84311	1.28E-06	3.04E-05
Owenia_fusiformis	OFUSG23074.1	104.5428	-1.35413	0.322864	-4.10313	4.08E-05	0.000576
Owenia_fusiformis	OFUSG23121.1	16.04034	-1.74075	0.443657	-3.53763	0.000404	0.003692
Owenia_fusiformis	OFUSG23149.1	76.96242	-1.20764	0.416422	-2.82613	0.004711	0.025636
Owenia_fusiformis	OFUSG23157.1	47.94559	-1.14381	0.393508	-2.85744	0.004271	0.023752
Owenia_fusiformis	OFUSG23159.1	56.82108	-1.24822	0.374391	-3.326	0.000881	0.006864
Owenia_fusiformis	OFUSG23166.1	72.19365	-1.81016	0.41466	-4.3076	1.65E-05	0.00027
Owenia_fusiformis	OFUSG23175.1	195.717	-3.82243	0.307059	-11.3878	4.81E-30	6.58E-27
Owenia_fusiformis	OFUSG23187.1	353.2728	-1.02355	0.332242	-3.08089	0.002064	0.013503
Owenia_fusiformis	OFUSG23191.4	337.513	-1.31186	0.310911	-4.2381	2.25E-05	0.000352
Owenia_fusiformis	OFUSG23217.1	60.88918	-2.2759	0.437452	-5.01834	5.21E-07	1.39E-05
Owenia_fusiformis	OFUSG23256.1	2333.319	-1.18687	0.21742	-5.45424	4.92E-08	1.85E-06
Owenia_fusiformis	OFUSG23286.1	37.27368	-1.50123	0.43607	-3.47544	0.00051	0.004449
Owenia_fusiformis	OFUSG23301.1	50.14303	-1.14271	0.447058	-2.67648	0.00744	0.036405
Owenia_fusiformis	OFUSG23305.1	47.85744	-1.36853	0.410315	-3.20608	0.001346	0.009597
Owenia_fusiformis	OFUSG23308.1	487.2999	-1.50682	0.263431	-5.6957	1.23E-08	5.53E-07
Owenia_fusiformis	OFUSG23324.1	420.4335	-1.31965	0.343025	-3.85623	0.000115	0.001343
Owenia_fusiformis	OFUSG23367.1	72.88021	-1.49782	0.391449	-3.88641	0.000102	0.001215
Owenia_fusiformis	OFUSG23391.3	559.3161	-2.17182	0.328747	-6.59475	4.26E-11	3.64E-09
Owenia_fusiformis	OFUSG23391.5	4311.57	-1.42279	0.219237	-6.50414	7.81E-11	6.21E-09
Owenia_fusiformis	OFUSG23442.2	409.311	-1.48456	0.275213	-5.38747	7.15E-08	2.56E-06
Owenia_fusiformis	OFUSG23457.2	4216.863	-1.01348	0.17456	-5.80183	6.56E-09	3.17E-07
Owenia_fusiformis	OFUSG23459.1	56.50389	-1.15696	0.431559	-2.96386	0.003038	0.018222
Owenia_fusiformis	OFUSG23466.3	3261.113	-1.17438	0.228939	-5.15295	2.56E-07	7.65E-06
Owenia_fusiformis	OFUSG23486.1	10350.09	-1.21385	0.202315	-5.99902	1.99E-09	1.11E-07
Owenia_fusiformis	OFUSG23496.1	486.4618	-1.03068	0.333643	-3.06962	0.002143	0.013904
Owenia_fusiformis	OFUSG23518.1	9.654897	-1.45372	0.438075	-2.89999	0.003732	0.021335
Owenia_fusiformis	OFUSG23536.1	90.5327	-1.3729	0.345241	-4.08402	4.43E-05	0.000617
Owenia_fusiformis	OFUSG23539.2	22.35029	-1.7137	0.446853	-4.23346	2.30E-05	0.000358
Owenia_fusiformis	OFUSG23580.2	153.9827	-1.13741	0.439772	-3.12499	0.001778	0.012068
Owenia_fusiformis	OFUSG23600.1	91.76991	-1.09336	0.364594	-2.9029	0.003697	0.021205
Owenia_fusiformis	OFUSG23634.1	864.4673	-1.24709	0.170943	-7.31055	2.66E-13	4.02E-11
Owenia_fusiformis	OFUSG23656.1	32.71643	-1.21619	0.430243	-3.06329	0.002189	0.014113
Owenia_fusiformis	OFUSG23707.1	711.0147	-1.20566	0.345055	-3.54715	0.000389	0.003596
Owenia_fusiformis	OFUSG23708.1	210.0124	-1.03133	0.443768	-2.76895	0.005624	0.02933
Owenia_fusiformis	OFUSG23730.1	12.15061	-1.39583	0.436081	-3.70882	0.000208	0.002184
Owenia_fusiformis	OFUSG23771.1	484.0052	-1.03939	0.36259	-2.80126	0.00509	0.027199
Owenia_fusiformis	OFUSG23782.1	17.94156	-1.42912	0.43809	-3.20486	0.001351	0.009632
Owenia_fusiformis	OFUSG23836.1	1068.082	-1.46353	0.317415	-4.6164	3.90E-06	7.95E-05
Owenia_fusiformis	OFUSG23856.1	18.72684	-1.81291	0.44564	-4.29313	1.76E-05	0.000285
Owenia_fusiformis	OFUSG23909.1	34.91516	-1.56221	0.438717	-3.41273	0.000643	0.00534
Owenia_fusiformis	OFUSG23918.1	247.7892	-1.35577	0.431685	-3.66212	0.00025	0.002526
Owenia_fusiformis	OFUSG23937.1	93.66097	-1.00657	0.311718	-3.28885	0.001006	0.007611
Owenia_fusiformis	OFUSG23942.1	23.87012	-2.6683	0.440472	-4.21004	2.55E-05	0.000389
Owenia_fusiformis	OFUSG23974.1	91.44604	-1.15253	0.379968	-3.29476	0.000985	0.00749
Owenia_fusiformis	OFUSG23976.1	24.85526	-1.22339	0.443586	-2.84692	0.004414	0.024382
Owenia_fusiformis	OFUSG23995.1	1024.38	-1.14429	0.186916	-6.11717	9.53E-10	5.81E-08
Owenia_fusiformis	OFUSG23998.1	25.78913	-1.3268	0.448242	-2.80488	0.005033	0.026948
Owenia_fusiformis	OFUSG24005.1	43.91586	-1.42482	0.448422	-3.25737	0.001124	0.008319
Owenia_fusiformis	OFUSG24008.1	894.1234	-1.0957	0.159517	-6.8624	6.77E-12	7.22E-10
Owenia_fusiformis	OFUSG24008.2	389.6367	-1.10702	0.212038	-5.21205	1.87E-07	5.81E-06
Owenia_fusiformis	OFUSG24008.3	437.7867	-1.65454	0.285593	-5.83792	5.29E-09	2.62E-07
Owenia_fusiformis	OFUSG24024.2	490.3932	-1.15854	0.294417	-3.98622	6.71E-05	0.000864
Owenia_fusiformis	OFUSG24049.2	55.46718	-1.05619	0.445495	-2.76238	0.005738	0.029788
Owenia_fusiformis	OFUSG24074.1	1501.396	-1.62809	0.252874	-6.46116	1.04E-10	8.02E-09
Owenia_fusiformis	OFUSG24109.1	396.1833	-1.09504	0.317005	-3.4398	0.000582	0.004927
Owenia_fusiformis	OFUSG24111.1	351.9584	-1.35229	0.351084	-3.87585	0.000106	0.00126
Owenia_fusiformis	OFUSG24120.1	9.611969	-1.42496	0.425843	-3.54218	0.000397	0.003641
Owenia_fusiformis	OFUSG24160.1	40.42621	-1.22204	0.403379	-3.03951	0.00237	0.015037
Owenia_fusiformis	OFUSG24161.1	164.2642	-1.06246	0.289155	-3.65986	0.000252	0.002545
Owenia_fusiformis	OFUSG24171.1	55.56718	-1.53158	0.424399	-3.73037	0.000191	0.002031
Owenia_fusiformis	OFUSG24188.1	12.07356	-1.4049	0.443662	-3.6163	0.000299	0.002921
Owenia_fusiformis	OFUSG24201.1	58.99434	-1.3195	0.379337	-3.42311	0.000619	0.005172

Owenia_fusiformis	OFUSG24233.1	42.03707	-1.91763	0.405174	-4.6118	3.99E-06	8.11E-05
Owenia_fusiformis	OFUSG24241.1	146.8246	-1.17025	0.356028	-3.32068	0.000898	0.006965
Owenia_fusiformis	OFUSG24293.4	108.2771	-1.16638	0.328372	-3.44822	0.000564	0.004797
Owenia_fusiformis	OFUSG24338.1	49.50239	-1.60884	0.419924	-3.80335	0.000143	0.001602
Owenia_fusiformis	OFUSG24406.1	64.29218	-1.50634	0.378307	-3.94646	7.93E-05	0.000993
Owenia_fusiformis	OFUSG24456.1	143.7887	-1.17249	0.306715	-3.81351	0.000137	0.001548
Owenia_fusiformis	OFUSG24461.1	11.37716	-1.29428	0.446359	-2.8224	0.004767	0.025831
Owenia_fusiformis	OFUSG24472.1	81.30091	-1.2909	0.40875	-3.19774	0.001385	0.009841
Owenia_fusiformis	OFUSG24475.1	242.6787	-2.09639	0.409535	-5.60147	2.13E-08	8.89E-07
Owenia_fusiformis	OFUSG24477.1	14.12295	-1.05283	0.439211	-2.57446	0.01004	0.045643
Owenia_fusiformis	OFUSG24518.1	652.2017	-2.11656	0.353645	-5.94316	2.80E-09	1.50E-07
Owenia_fusiformis	OFUSG24576.1	4230.683	-1.14688	0.212943	-5.40856	6.35E-08	2.31E-06
Owenia_fusiformis	OFUSG24637.1	330.0671	-1.80454	0.355991	-5.44117	5.29E-08	1.98E-06
Owenia_fusiformis	OFUSG24645.1	20.59787	-1.16601	0.428856	-2.75328	0.0059	0.030431
Owenia_fusiformis	OFUSG24668.1	49.80645	-1.4399	0.423815	-3.40412	0.000664	0.00548
Owenia_fusiformis	OFUSG24749.1	240.0732	-1.10425	0.389688	-2.8863	0.003898	0.022114
Owenia_fusiformis	OFUSG24757.1	250.9561	-1.69443	0.304709	-5.59395	2.22E-08	9.20E-07
Owenia_fusiformis	OFUSG24762.1	73.57409	-1.74789	0.447843	-4.62305	3.78E-06	7.73E-05
Owenia_fusiformis	OFUSG24793.2	124.6089	-1.82997	0.424096	-4.27905	1.88E-05	0.0003
Owenia_fusiformis	OFUSG24794.1	649.1321	-1.15943	0.156745	-7.39527	1.41E-13	2.21E-11
Owenia_fusiformis	OFUSG24817.1	130.0824	-2.52245	0.430865	-6.03137	1.63E-09	9.29E-08
Owenia_fusiformis	OFUSG24817.2	196.8688	-2.03749	0.284082	-7.11351	1.13E-12	1.47E-10
Owenia_fusiformis	OFUSG24822.1	1137.006	-1.48765	0.17836	-8.33246	7.92E-17	2.47E-14
Owenia_fusiformis	OFUSG24831.1	95.97324	-2.18313	0.396181	-5.41793	6.03E-08	2.20E-06
Owenia_fusiformis	OFUSG24844.1	176.0426	-3.07622	0.390889	-7.69192	1.45E-14	2.88E-12
Owenia_fusiformis	OFUSG24928.1	149.3711	-1.04338	0.36892	-2.87751	0.004008	0.02262
Owenia_fusiformis	OFUSG24934.1	636.3905	-1.4326	0.231337	-6.20318	5.53E-10	3.62E-08
Owenia_fusiformis	OFUSG24986.2	247.3208	-1.04804	0.381503	-2.74465	0.006058	0.031008
Owenia_fusiformis	OFUSG25009.1	71.3306	-1.10362	0.406835	-2.89775	0.003759	0.021456
Owenia_fusiformis	OFUSG25014.2	322.2464	-1.25534	0.368324	-3.52356	0.000426	0.003844
Owenia_fusiformis	OFUSG25036.2	501.1171	-1.26737	0.346934	-3.74323	0.000182	0.001952
Owenia_fusiformis	OFUSG25038.5	4554.317	-1.3211	0.294473	-4.52881	5.93E-06	0.000115
Owenia_fusiformis	OFUSG25090.2	98.58323	-2.80822	0.426462	-5.74408	9.24E-09	4.29E-07
Owenia_fusiformis	OFUSG25106.1	487.043	-1.07653	0.300013	-3.56251	0.000367	0.003434
Owenia_fusiformis	OFUSG25120.1	62.27769	-2.04285	0.443017	-3.79019	0.000151	0.001673
Owenia_fusiformis	OFUSG25144.1	20.61494	-1.637	0.446658	-3.61147	0.000304	0.002965
Owenia_fusiformis	OFUSG25153.1	367.8308	-1.21853	0.186512	-6.51823	7.11E-11	5.73E-09
Owenia_fusiformis	OFUSG25158.1	62.8298	-2.57812	0.36711	-6.60537	3.97E-11	3.44E-09
Owenia_fusiformis	OFUSG25176.1	31.46503	-1.23005	0.439502	-2.88875	0.003868	0.021988
Owenia_fusiformis	OFUSG25187.1	119.1236	-1.41483	0.336572	-4.16327	3.14E-05	0.000463
Owenia_fusiformis	OFUSG25241.1	1562.693	-2.14401	0.255592	-8.41385	3.97E-17	1.29E-14
Owenia_fusiformis	OFUSG25262.1	2839.669	-1.34589	0.299931	-4.48415	7.32E-06	0.000137
Owenia_fusiformis	OFUSG25283.1	217.3124	-1.13963	0.328879	-3.45546	0.000549	0.004709
Owenia_fusiformis	OFUSG25292.1	128.6015	-1.70873	0.414558	-4.14218	3.44E-05	0.0005
Owenia_fusiformis	OFUSG25293.1	80.39292	-1.41157	0.361062	-3.85554	0.000115	0.001345
Owenia_fusiformis	OFUSG25312.1	154.126	-1.09353	0.351996	-3.05663	0.002238	0.014381
Owenia_fusiformis	OFUSG25342.1	17.97386	-1.71484	0.444159	-4.14924	3.34E-05	0.000487
Owenia_fusiformis	OFUSG25366.1	223.1004	-1.68861	0.335374	-4.96903	6.73E-07	1.73E-05
Owenia_fusiformis	OFUSG25416.1	612.3282	-1.38967	0.322607	-4.29579	1.74E-05	0.000283
Owenia_fusiformis	OFUSG25434.2	89.1372	-1.57421	0.443182	-4.05644	4.98E-05	0.00068
Owenia_fusiformis	OFUSG25463.1	221.2683	-1.2828	0.410072	-3.10868	0.001879	0.012556
Owenia_fusiformis	OFUSG25521.3	23.28629	-1.16249	0.404229	-3.81174	0.000138	0.001557
Owenia_fusiformis	OFUSG25532.1	23.04817	-1.34257	0.447063	-3.29217	0.000994	0.00754
Owenia_fusiformis	OFUSG25538.1	39.72125	-1.26494	0.448	-2.75908	0.005796	0.030015
Owenia_fusiformis	OFUSG25552.1	58.17333	-1.32998	0.408966	-3.30391	0.000953	0.007297
Owenia_fusiformis	OFUSG25585.1	30.50806	-1.24127	0.431799	-2.69194	0.007104	0.035122
Owenia_fusiformis	OFUSG25586.1	125.5214	-1.21429	0.364194	-3.25133	0.001149	0.008462
Owenia_fusiformis	OFUSG25633.2	491.58	-1.23549	0.31328	-3.94498	7.98E-05	0.000998
Owenia_fusiformis	OFUSG25633.3	230.9591	-1.2151	0.25162	-4.84312	1.28E-06	3.04E-05
Owenia_fusiformis	OFUSG25633.5	28.28715	-1.87307	0.438567	-4.52661	5.99E-06	0.000116
Owenia_fusiformis	OFUSG25670.1	18.70283	-1.18005	0.44635	-3.57212	0.000354	0.003341
Owenia_fusiformis	OFUSG25672.1	40.14156	-1.85047	0.417854	-4.27579	1.90E-05	0.000304
Owenia_fusiformis	OFUSG25674.1	18.70283	-1.18005	0.44635	-3.57212	0.000354	0.003341
Owenia_fusiformis	OFUSG25675.1	83.80831	-3.52558	0.366621	-5.36927	7.91E-08	2.79E-06
Owenia_fusiformis	OFUSG25694.1	256.9841	-1.08012	0.399721	-2.56814	0.010225	0.046321
Owenia_fusiformis	OFUSG25715.1	58.26112	-1.50837	0.423199	-3.65666	0.000256	0.002565
Owenia_fusiformis	OFUSG25739.1	178.7899	-1.17083	0.29352	-3.95227	7.74E-05	0.000971
Owenia_fusiformis	OFUSG25774.1	31.52662	-1.05505	0.432087	-2.82798	0.004684	0.025527
Owenia_fusiformis	OFUSG25825.1	65.64834	-1.80347	0.448479	-3.82763	0.000129	0.001478
Owenia_fusiformis	OFUSG25846.1	18.30018	-1.34618	0.447304	-3.13913	0.001694	0.011631
Owenia_fusiformis	OFUSG25918.1	415.0767	-1.19335	0.39973	-2.89041	0.003847	0.021898
Owenia_fusiformis	OFUSG25967.1	13.07174	-1.19801	0.446415	-2.85653	0.004283	0.023787
Owenia_fusiformis	OFUSG26007.1	92.46925	-2.98788	0.403464	-6.75892	1.39E-11	1.35E-09
Owenia_fusiformis	OFUSG26013.1	137.0676	-1.62517	0.364437	-4.38535	1.16E-05	0.000201
Owenia_fusiformis	OFUSG26017.2	824.2558	-1.49607	0.185458	-8.06605	7.26E-16	1.92E-13
Owenia_fusiformis	OFUSG26036.1	2094.066	-1.25501	0.185182	-6.77734	1.22E-11	1.21E-09
Owenia_fusiformis	OFUSG26060.1	41.99369	-1.52355	0.400219	-3.74703	0.000179	0.001925
Owenia_fusiformis	OFUSG26072.1	948.8124	-1.10696	0.207867	-5.31701	1.05E-07	3.54E-06
Owenia_fusiformis	OFUSG26079.1	26.89491	-1.55035	0.441755	-3.51715	0.000436	0.003915
Owenia_fusiformis	OFUSG26126.1	47.43251	-1.28961	0.421644	-3.01791	0.002545	0.015849

Owenia_fusiformis	OFUSG26232.1	15.60994	-1.19677	0.447005	-2.71913	0.006545	0.032989
Owenia_fusiformis	OFUSG26242.1	244.7215	-1.34568	0.317323	-4.24392	2.20E-05	0.000344
Owenia_fusiformis	OFUSG26257.2	300.6281	-1.03756	0.352235	-3.02958	0.002449	0.015405
Owenia_fusiformis	OFUSG26269.1	410.854	-1.82754	0.425483	-4.12395	3.72E-05	0.000534
Owenia_fusiformis	OFUSG26323.1	24.72411	-1.24121	0.393228	-4.04063	5.33E-05	0.000716
Owenia_fusiformis	OFUSG26371.1	405.0904	-1.89663	0.377598	-5.04327	4.58E-07	1.25E-05
Owenia_fusiformis	OFUSG26379.1	47.83207	-1.12207	0.414092	-2.76836	0.005634	0.029364
Owenia_fusiformis	OFUSG26405.1	24.19854	-1.21068	0.447182	-3.76474	0.000167	0.001819
Owenia_fusiformis	OFUSG26417.1	24.23295	-1.30048	0.446296	-3.09604	0.001961	0.012994
Owenia_fusiformis	OFUSG26437.1	1024.698	-1.74821	0.28376	-6.14323	8.09E-10	5.02E-08
Owenia_fusiformis	OFUSG26438.1	37.68844	-1.75095	0.446589	-4.05084	5.10E-05	0.000691
Owenia_fusiformis	OFUSG26458.1	31.06076	-1.72897	0.43818	-3.79932	0.000145	0.001623
Owenia_fusiformis	OFUSG26482.1	120.9678	-1.13153	0.445802	-2.99418	0.002752	0.016833
Owenia_fusiformis	OFUSG26529.2	184.4441	-2.02922	0.32698	-6.08753	1.15E-09	6.76E-08
Owenia_fusiformis	OFUSG26536.1	15.14404	-1.33555	0.432689	-3.71503	0.000203	0.002137
Owenia_fusiformis	OFUSG26573.1	641.1936	-1.4744	0.408184	-3.50722	0.000453	0.004033
Owenia_fusiformis	OFUSG26573.2	214.9447	-1.40262	0.438891	-3.02894	0.002454	0.01542
Owenia_fusiformis	OFUSG26575.1	322.9504	-1.091	0.248885	-4.38423	1.16E-05	0.000202
Owenia_fusiformis	OFUSG26593.1	103.5662	-1.19078	0.333619	-3.57479	0.000351	0.003316
Owenia_fusiformis	OFUSG26608.2	36.85887	-1.24613	0.446542	-3.12053	0.001805	0.012194
Owenia_fusiformis	OFUSG26637.1	38.33326	-1.92131	0.440173	-4.5952	4.32E-06	8.71E-05
Owenia_fusiformis	OFUSG26638.1	164.0507	-1.27232	0.329001	-3.8324	0.000127	0.001456
Owenia_fusiformis	OFUSG26651.4	89.02992	-1.32952	0.326604	-4.01143	6.04E-05	0.00079
Owenia_fusiformis	OFUSG26659.1	291.63	-1.1716	0.316389	-3.71316	0.000205	0.002151
Owenia_fusiformis	OFUSG26703.1	78.84594	-1.27969	0.438116	-2.96322	0.003044	0.018242
Owenia_fusiformis	OFUSG26714.1	28.40782	-1.58033	0.438421	-3.53129	0.000414	0.003764
Owenia_fusiformis	OFUSG26787.1	403.2538	-1.26867	0.227536	-5.53477	3.12E-08	1.24E-06
Owenia_fusiformis	OFUSG26791.1	27.75933	-1.46663	0.448235	-3.12845	0.001757	0.011962
Owenia_fusiformis	OFUSG26797.1	55.52043	-1.10533	0.353657	-3.0166	0.002556	0.015897
Owenia_fusiformis	OFUSG26829.1	1503.792	-1.02472	0.190923	-5.3607	8.29E-08	2.90E-06
Owenia_fusiformis	OFUSG26830.1	3781.277	-1.89636	0.135417	-13.9969	1.63E-44	4.76E-41
Owenia_fusiformis	OFUSG26835.1	10.80118	-1.08317	0.445592	-2.67364	0.007503	0.036612
Owenia_fusiformis	OFUSG26848.1	10555.62	-1.46041	0.146551	-9.96114	2.25E-23	1.79E-20
Owenia_fusiformis	OFUSG26849.1	44.07448	-1.32089	0.443711	-2.89995	0.003732	0.021335
Owenia_fusiformis	OFUSG26856.2	1844.308	-1.13305	0.448695	-3.26376	0.001099	0.008163
Owenia_fusiformis	OFUSG26886.1	279.043	-1.44619	0.220671	-6.53689	6.28E-11	5.12E-09
Owenia_fusiformis	OFUSG26926.1	13.70196	-1.38421	0.445713	-3.14923	0.001637	0.011321

Supplementary Table 38. Scoring of morphological phenotypes of stage 5 and 7 larvae of C. teleta treated with SB431542.

Species	Treatment	Time_window	Stage	Total_n	Normal	Ball	Curved_trunk	Other_abnormal
Capitella_teleta	0.4 % DMSO	4-cell-to-32-cell	5	19	12	7	NA	NA
Capitella_teleta	40uM SB431542	4-cell-to-32-cell	5	21	3	18	NA	NA
Capitella_teleta	0.4 % DMSO	4-cell-to-32-cell	7	48	35	10	0	3
Capitella_teleta	40uM SB431543	4-cell-to-32-cell	7	64	3	52	9	0
