# Supplementary material for: Developmental system drift in dorsoventral patterning is linked to transitions to autonomous development in Annelida
Source: Nat Commun. 2026 Apr 18;17:5381. doi: 10.1038/s41467-026-71950-7 (PMC13275895; doi:10.1038/s41467-026-71950-7)
Supplement: Supplementary file 3 — Reporting Summary [file 41467_2026_71950_MOESM3_ESM.pdf]

nature portfolio

Corresponding author(s): Stephan Q. Schneider, José M. Martín-Durán

Last updated by author(s): Mar 21, 2026

Reporting Summary

Nature Portfolio wishes to improve the reproducibility of the work that we publish. This form provides structure for consistency and transparency in reporting. For further information on Nature Portfolio policies, see our Editorial Policies and the Editorial Policy Checklist.

Statistics

For all statistical analyses, confirm that the following items are present in the figure legend, table legend, main text, or Methods section.

n/a	Confirmed	
☐	☒	The exact sample size (*n*) for each experimental group/condition, given as a discrete number and unit of measurement
☐	☒	A statement on whether measurements were taken from distinct samples or whether the same sample was measured repeatedly
☐	☒	The statistical test(s) used AND whether they are one- or two-sided<br>*Only common tests should be described solely by name; describe more complex techniques in the Methods section.*
☒	☐	A description of all covariates tested
☐	☒	A description of any assumptions or corrections, such as tests of normality and adjustment for multiple comparisons
☐	☒	A full description of the statistical parameters including central tendency (e.g. means) or other basic estimates (e.g. regression coefficient) AND variation (e.g. standard deviation) or associated estimates of uncertainty (e.g. confidence intervals)
☐	☒	For null hypothesis testing, the test statistic (e.g. *F*, *t*, *r*) with confidence intervals, effect sizes, degrees of freedom and *P* value noted<br>*Give P values as exact values whenever suitable.*
☒	☐	For Bayesian analysis, information on the choice of priors and Markov chain Monte Carlo settings
☒	☐	For hierarchical and complex designs, identification of the appropriate level for tests and full reporting of outcomes
☒	☐	Estimates of effect sizes (e.g. Cohen's *d*, Pearson's *r*), indicating how they were calculated

Our web collection on statistics for biologists contains articles on many of the points above.

Software and code

Policy information about availability of computer code

Data collection	No custom code was utilised, we followed standard DESeq2 differential expression analyses.
Data analysis	For Owenia fusiformis, Adapter and low-quality bases were removed using FastP v.0.20.1. Cleaned reads were mapped to the O. fusiformis genome annotation using Kallisto v.0.46.2. Read counts normalisation and differential gene expression analyses for each pairwise comparison between control and treatment conditions were performed with the R package DESeq2 v.1.26.0. The enrichment of Gene Ontology terms in the differentially expressed genes was calculated with the TopGO R package. For Platynereis dumerilii, Raw reads were quality-trimmed to remove adapters and low-quality bases using Trimmomatic v.0.39. Trimmed reads were mapped to the Platynereis reference genome v.0.2.1 using STAR v.2.7.11b, and gene abundance was estimated with featureCounts v.2.0.1. Read counts normalisation and differential gene expression analyses for each pairwise comparison between control and treatment conditions were performed with the R package DESeq2 v.1.26.0, adjusting log2 fold changes with the shrinkage approach included in DESeq2.

For manuscripts utilizing custom algorithms or software that are central to the research but not yet described in published literature, software must be made available to editors and reviewers. We strongly encourage code deposition in a community repository (e.g. GitHub). See the Nature Portfolio guidelines for submitting code & software for further information.

Data

Policy information about availability of data

All manuscripts must include a data availability statement. This statement should provide the following information, where applicable:

- Accession codes, unique identifiers, or web links for publicly available datasets
- A description of any restrictions on data availability
- For clinical datasets or third party data, please ensure that the statement adheres to our policy

All expression data generated in this manuscript has been uploaded to SRA (project ID PRJNA1267570) and Gene Expression Omnibus (study ID GSE297936).

Research involving human participants, their data, or biological material

Policy information about studies with human participants or human data. See also policy information about sex, gender (identity/presentation), and sexual orientation and race, ethnicity and racism.

Reporting on sex and gender	*Use the terms sex (biological attribute) and gender (shaped by social and cultural circumstances) carefully in order to avoid confusing both terms. Indicate if findings apply to only one sex or gender; describe whether sex and gender were considered in study design; whether sex and/or gender was determined based on self-reporting or assigned and methods used. Provide in the source data disaggregated sex and gender data, where this information has been collected, and if consent has been obtained for sharing of individual-level data; provide overall numbers in this Reporting Summary. Please state if this information has not been collected. Report sex- and gender-based analyses where performed, justify reasons for lack of sex- and gender-based analysis.*
Reporting on race, ethnicity, or other socially relevant groupings	*Please specify the socially constructed or socially relevant categorization variable(s) used in your manuscript and explain why they were used. Please note that such variables should not be used as proxies for other socially constructed/relevant variables (for example, race or ethnicity should not be used as a proxy for socioeconomic status). Provide clear definitions of the relevant terms used, how they were provided (by the participants/respondents, the researchers, or third parties), and the method(s) used to classify people into the different categories (e.g. self-report, census or administrative data, social media data, etc.) Please provide details about how you controlled for confounding variables in your analyses.*
Population characteristics	*Describe the covariate-relevant population characteristics of the human research participants (e.g. age, genotypic information, past and current diagnosis and treatment categories). If you filled out the behavioural & social sciences study design questions and have nothing to add here, write "See above."*
Recruitment	*Describe how participants were recruited. Outline any potential self-selection bias or other biases that may be present and how these are likely to impact results.*
Ethics oversight	*Identify the organization(s) that approved the study protocol.*

Note that full information on the approval of the study protocol must also be provided in the manuscript.

Field-specific reporting

Please select the one below that is the best fit for your research. If you are not sure, read the appropriate sections before making your selection.

[x] Life sciences [ ] Behavioural & social sciences [ ] Ecological, evolutionary & environmental sciences

For a reference copy of the document with all sections, see nature.com/documents/nr-reporting-summary-flat.pdf

Life sciences study design

All studies must disclose on these points even when the disclosure is negative.

Sample size	For bulk RNA-seq a minimum of 300 individuals were snap-frozen. For statistical analyses, most treatments were done in replicates (n=3), with each replicate having >100 individuals.
Data exclusions	No data exclusions
Replication	Two and three replicates were used for bulk RNA-seq of Owenia fusiformis and Platynereis dumerilii embryos, respectively. Statistical analyses were done with three replicates per treatment.
Randomization	Embryos from a mix females were pooled and randomly assigned to the different treatments.
Blinding	Blinding was not necessary, most scoring was done as present/absence.

Reporting for specific materials, systems and methods

We require information from authors about some types of materials, experimental systems and methods used in many studies. Here, indicate whether each material, system or method listed is relevant to your study. If you are not sure if a list item applies to your research, read the appropriate section before selecting a response.

Materials & experimental systems

n/a	Involved in the study
☐	☒ Antibodies
☒	☐ Eukaryotic cell lines
☒	☐ Palaeontology and archaeology
☐	☒ Animals and other organisms
☒	☐ Clinical data
☒	☐ Dual use research of concern
☒	☐ Plants

Methods

n/a	Involved in the study
☒	☐ ChIP-seq
☒	☐ Flow cytometry
☒	☐ MRI-based neuroimaging

Antibodies

Antibodies used

mouse anti-acetylated α-tubulin (clone 6-11B-1, Merck-Sigma; Cat No.: #MABT868, 1:500), mouse anti-β-tubulin (E7, Developmental Studies Hybridoma Bank), rabbit anti-Phospho-Smad1/5 (Cell Signaling Technology; Cat No.: 9516),mouse anti-dp-ERK1/2 (Merck-Sigma; Cat No.: #M9692),P. dumerilii-derived rabbit anti-RYamide, goat anti-mouse AlexaFluor 488 (ThermoFisher Scientific; Cat No.: A32731), goat anti-mouse AlexaFluor 647 (ThermoFisher Scientific; Cat No.: A-21235), and goat anti-rabbit AlexaFluor 555 (ThermoFisher Scientific; Cat No.: A32731).

Validation

Antibodies were only validated with immunofluorescence or colourimetric detection in whole organisms.

Animals and other research organisms

Policy information about studies involving animals; ARRIVE guidelines recommended for reporting animal research, and Sex and Gender in Research

Laboratory animals

Capitella teleta
Platynereis dumerilii

Wild animals

Owenia fusiformis
Spirobranchus lamarcki

Reporting on sex

n/a

Field-collected samples

Owenia fusiformis and Spirobranchus lamarcki were collected and transported to sea tables or CT incubators with artificial sea water, and kept there for the duration of the experiments.

Ethics oversight

No ethical approval was needed.

Note that full information on the approval of the study protocol must also be provided in the manuscript.

Plants

Seed stocks

Report on the source of all seed stocks or other plant material used. If applicable, state the seed stock centre and catalogue number. If plant specimens were collected from the field, describe the collection location, date and sampling procedures.

Novel plant genotypes

Describe the methods by which all novel plant genotypes were produced. This includes those generated by transgenic approaches, gene editing, chemical/radiation-based mutagenesis and hybridization. For transgenic lines, describe the transformation method, the number of independent lines analyzed and the generation upon which experiments were performed. For gene-edited lines, describe the editor used, the endogenous sequence targeted for editing, the targeting guide RNA sequence (if applicable) and how the editor was applied.

Authentication

Describe any authentication procedures for each seed stock used or novel genotype generated. Describe any experiments used to assess the effect of a mutation and, where applicable, how potential secondary effects (e.g. second site T-DNA insertions, mosiacism, off-target gene editing) were examined.
