# Supplementary material for: Developmental system drift in dorsoventral patterning is linked to transitions to autonomous development in Annelida
Source: Nat Commun. 2026 Apr 18;17:5381. doi: 10.1038/s41467-026-71950-7 (PMC13275895; doi:10.1038/s41467-026-71950-7)
Supplement: Supplementary file 2 — Peer Review file [file 41467_2026_71950_MOESM2_ESM.pdf]

nature portfolio

Peer Review File

Developmental system drift in dorsoventral patterning is linked to transitions to autonomous development in Annelida

Corresponding Author: Dr José Martín-Durán

This file contains all reviewer reports in order by version, followed by all author rebuttals in order by version.

Version 0:

Reviewer comments:

Reviewer #1

(Remarks to the Author)
Key results & significance
The paper compares mechanisms of DV axis specification in three species of annelids, two with equal spiral cleavage (Owenia fusiformis, Spirobranchus lamarcki) and one with unequal spiral cleavage (Platynereis dumerilii), in an attempt to reconcile the broad range of molecular mechanisms previously found to affect DV axis formation in different spiralian species. The authors focus on the function of MAPK activation (pERK1/2) in establishing the DV organizer in equally-cleaving embryos and subsequent activation of BMP versus Activin/Nodal signaling to pattern the DV axis. The main results are that in both equally-cleaving animals, activation of ERK1/2 in one cell (4d) breaks symmetry and establishes this cell as the DV organizer. Shortly after ERK1/2 activation, BMP signaling, and to a lesser extent Nodal signaling in O. fusiformis, then establishes dorsal/dorsoposterior tissues. In the unequally-cleaving P. dumerilii, 2d is likely autonomously established and acts as the DV organizer for the head/episphere using BMP signaling to form dorsal tissues; no function for Activin/Nodal signaling in DV axis formation was found. The authors also compare differential gene expression after up- and down-regulation of BMP signaling during cleavage stages in O. fusiformis, P. dumerilii, and another unequally-cleaving annelid, C. teleta, and find very little overlap in BMP responsive genes across the three species. Overall, I think this paper is very interesting and the comparative focus on the molecular mechanism of DV axis formation is relevant for a broad audience of developmental biologists. The authors also ask fundamental questions about how developmental systems can shift over evolutionary time, which is broadly relevant for evolutionary and evo devo biologists.

Validity of results re: conclusions
One question is whether the mechanisms being tested in this paper are involved in DV axis formation versus specification of blastomere fate and/or quadrant identity (e.g., D versus B quadrants) during early cleavage stages in spiralian embryos. The blastomeres ultimately generate the body plan and specific tissues, but the tissues formed by each quadrant aren't partitioned along the DV axis of the eventual larva or the adult of every spiralian species. The data in this paper support a function for MAPK and BMP signaling in breaking symmetry in radially-symmetric, equally-cleaving embryo. MAPK establishes the D-quadrant organizer, which then uses BMP and/or Nodal/Activin signaling to specify the fate of different blastomeres along a D to B axis. How this relates to the ancestral DV axis is still unclear to me (further comments below). I don't think there is a fate map for O. fusiformis, and perturbation of MAPK and BMP signaling in P. lamarcki seem to affect tissues between the episphere and hyposphere rather than along the DV axis. Furthermore, in P. dumerilii, 1c and 1d micromeres generate dorsal ectoderm in the episphere while 1a and 1b generate the ventral ectoderm. However, for the trunk, I don't think there is a similar division of blastomere fate and ectoderm tissue along the DV axis; 2d and 4d form most of the trunk ectoderm and mesoderm, respectively. I wonder if finding a function for DV axis formation in the head but not trunk in this animal is related to the division of blastomere fate along the DV axis in the head but not trunk. I think a more nuanced discussion of fate maps in different species versus how BMP signaling functions in each animal would be helpful for understanding evolution of the molecular control of DV axis formation. The ancestral spiralian DV axis could have been established by the D (dorsal) to B (ventral) quadrant identities; however, there are many taxa (especially annelids) that do not have this division in the trunk.

For the comparison of DEGs between rBMP4 and rActivinA treatments in O. fusiformis, the results are difficult to interpret since different concentrations of the two proteins (1000 versus 75 ng/mL) were used. Both proteins are known to activate different sets of genes at different concentrations in other animals; the amount of rBMP4 could have been maximally activating while the amount of rActivinA could have been a low concentration. Similarly, for the two drugs (DMH1 and

SB431542), you could have been maximally inhibiting a pathway with one drug and only partially inhibiting with the other drug. Without dose response curves for the proteins and drugs and quantification of pSMAD2/3 and pSMAD1/5/8 levels, I think caution is needed when directly comparing the DEGs for the different treatments within the same animal. This same problem holds for the comparisons between species. At the very least, I think assessment of one DV phenotype at different concentrations of rBMP4 is needed for O. fusiformis and P. dumerilii.

The results and conclusions presented for O. fusiformis are the most convincing. The descriptions of the data from S. lamarcki and P. dumerilii are less clearly explained, making these data sets more difficult to interpret (see comments below). I think this can be fixed with text edits that clarify how the experiments were done and explain the figures in more depth. Adding labels and diagrams to the figures could also improve accessibility for readers not familiar with spiralian development.

Suggested edits/minor questions
Would be helpful to add text about the results in Owenia relative to the quadrants after 4d specification. This would help with comparisons to other spiralian taxa.

Line 34 “implies”

Line 51 “of known animals”

Line 119 The region of overlap between bmp2/4 and chordin is difficult to see in the lateral blastula stage images. The two patterns look non-overlapping in the images shown.

Line 135 Based on the diagram in Fig. 1g, it appears that pSMAD1/5/8 is present in ectoderm, mesoderm, and endoderm precursor cells at 6 hpf. Do these pSMAD1/5/8+ cells go on to form dorsoposterior tissues in each germ layer, or do only the ectodermal precursor cells form dorsoposterior tissue in the larva? (And how does this relate to the juvenile axes?)

Line 151 Given that the SMAD1/5/8 homolog in Owenia does not have the ERK phosphorylation sites present in deuterostomes, how do you think the organizer regulates SMAD activity? How do you think U0126 blocks phosphorylation of SMAD1/5/8 in Owenia?

Line 154 In the U0126 treatments from 4–6 hpf in Ofu, was 4d specified? Does treatment during this time window block pERK in 4d? There are arrowheads indicating 4d in Fig. 1i, j, so it looks like 4d is morphologically visible.

Line 155 Did U0126 treatment affect bmp5-8 expression in O. fusiformis?

In L. goshimai (Tan et al. 2021), U0126 treatment causes expression of chd in all 3Q macromeres; chd is normally not expressed in the organizer 3D. In O. fusiformis, U0126 treatment appears to affect chd expression in the ectoderm but not the endomesoderm. Is this correct? Do all 4Q macromeres and 4q micromeres express chd after U0126 treatment in Owenia (Fig. 1)? Is MAPK signaling from the organizer inhibiting bmp2/4 expression on the future dorsal side of the embryo in Owenia?

Line 164 For these experiments, you used 1000 and 2000 ng/mL recombinant BMP4 protein, which is a very high concentration compared to other animals. Did you try lower concentrations and did those affect pSMAD 1/5/8 levels? Why do you think so much rBMP4 protein was needed to see an effect?

Line 166 Is the chaetal sac posterior or ventroposterior tissue? Based on the morphological descriptions, it seems like blocking BMP signaling does not cause a radialized phenotype while ectopic BMP signaling does. Wouldn’t you expect radialized phenotypes for both blocking and upregulating BMP signaling? Is it possible that DMH1 is only partially blocking BMP signaling while the amount of rBMP4 protein added is maximally activating? Even though there is a loss of pSMAD1/5/8 antibody labeling with DMH1 treatment, there could still be non-detectable but biologically-relevant levels of pSMAD.

Line 185 Similar to the comment above, why do you think there were so many more DEGs detected in the BMP-treated embryos versus the DMH1-treated embryos?

Line 228 Spell out genus when start with same letter and in same sentence.

Line 234 In S. lamarcki, one cell has pERK at 6 hpf, which is assumed to be 4d. Does this cell divide later than the other 4q micromeres (as in Owenia)? Is this cell larger than the other 4q micromeres? Was pERK detected in other blastomeres before 6 hpf? What are the first morphological asymmetries visible during spiral cleavage in this animal?

Line 238 Use past tense verbs for treatments

Line 238 What was the treatment window for U0126? Were any other treatment windows tested? Same question for DMH1 and rBMP4 treatments.

Line 239 DMH1 treatment is stated to ‘not affect di-pERK1/2 enrichment in 4d’; however, in Suppl. Table 17, 71% of the DMH1-treated embryos were reported to have pERK while 88% of the controls had pERK. I think a statistical analysis is

needed to test if these two treatments are different.

In P. lamarcki, which blastomeres have pSMAD1/5/8? Is pSMAD1/5/8 detected before 6 hpf? Does expression in the D quadrant (or future dorsal?) persist past 6 hpf? Does pERK1/2 proceed pSMAD1/5/8 as in O. fusiformis?

Line 244 What was the pSMAD1/5/8 pattern after rBMP4 treatment in P. lamarcki?

Line 244 In Fig. 3e, both the episphere and hyposphere appear reduced relative to the controls in P. lamarcki, not just the episphere. Was this quantified? Is the reduction for the episphere along the AP and/or DV axis? Were there specific tissues missing in the episphere? In Fig. 3e, the apical tuft is labeled in the rBMP4-treated animal suggesting that this tissue is still present. Was this quantified? Were there any DV phenotypes noted for the rBMP4 treatment (or the DMH1 treatment)?

Line 246 It's stated that BMP signaling establishes the DV axis in S. lamarcki; however, the reported phenotypes after DMH1 and rBMP4 treatment appear to be along the AP axis or between the episphere (anterior?) and hyposphere (posterior?). If there are clear affects on tissues localized along the DV axis, there needs to be an explanation and demonstration of these. As presented, the data do not support a function of BMP signaling in DV axis formation. Addition of a diagram of the larva with labeled tissues and axes after the different treatments (cont, U0126, DMH1, rBMP4) could be helpful.

Is there a chordin and/or chordin-like homolog in S. lamarcki? If so, where is this gene(s) expressed during cleavage stages? I think this information would be an interesting addition to the paper since the regulation of chordin expression is an important part of DV axis formation in L. goshimai (and vertebrates) but apparently not in O. fusiformis.

Line 258 Not all unequally-cleaving spiralian embryos have the same contributions to dorsal versus ventral ectoderm in the episphere. For example, in Ilyanassa obsoleta most of the dorsal-left and dorsal-right tissue in the episphere is generated by 1a and 1c, respectively, while 1b forms most of the ventral tissue in the episphere. Also, the contributions to dorsal versus ventral tissue are for the ectoderm but not mesoderm in the head.

Line 261 I'm not sure I understand this as written. Is this statement for P. dumerilii, unequally-cleaving annelids, or unequally-cleaving spiralians? In annelids, 2d and 4d generally form all or most of the ectoderm and mesoderm of the hyposphere, respectively. I am not aware of fate maps for annelids that show that 2d and 4d generate dorsal tissue rather than ventral tissue in the trunk.

Line 262 I don't see how early cell divisions define the DV axis in the trunk of P. dumerilii. This needs to be explained. Also, in the Pfeifer et al. 2014 paper, they used 10, 25 and 50 uM U0126 from 13.5 to 16.5 hpf, which I think is after 2d and 4d are born, making these treatments not comparable with the current study. Have earlier U0126 treatments been carried out in Pdu? Pfeifer et al. did not report seeing pERK in 2d, 4d or 3D, but they could have missed low levels of pERK at early cleavage stages, so the statement that DV patterning "arises independently of ERK1/2 activity" doesn't seem to be strongly supported.

Line 263 "P. dumerilii"

Line 270 Is bmp2/4 expressed in 1a and 1b or only in 1c and 1d at 6 hpf? Which cell(s) express dlx at 6 hpf? I think it's necessary to state which cells express each gene at least at the 6 and 8 hpf stages rather than saying that the genes are expressed "dorsally" or "ventrally". In the animal views it's difficult to tell where the quadrant boundaries are. Are the C and D quadrants at the top of the figure panel? Are the "dorsal" views lateral images with the D quadrant centered?

Line 271 Similar to the comment above, it's not clear to me which blastomeres are being considered "ventral trunk" precursors. Are bmp 3 and noggin expressed in A and B quadrant micromeres? Is the B quadrant centered in the lateral views in Fig. 4c?

Does P. dumerilii have chordin-like and gremlin homologs? What are their expression patterns during cleavage? Where is bmp5-8 expressed during cleavage stages?

Line 281 Why were the DMH1 and rBMP4 treatments done for such a long time window (i.e., from 2 hpf to 72 hpf)? I think treatments only spanning birth of 1c and 1d, birth of 2d, and birth of 4d are needed for comparison with the other two species. Later, it's mentioned that shorter treatments were used, but the time length relative to the developmental stages is not explained for each of the treatments.

Line 282 I don't understand how the described morphological phenotypes after DMH1 treatment are "ventralized". Please explain. In the diagram in Fig. 4a, the eyes are depicted as dorsal. Are the larval and adult eyes generated by 1a and 1c in P. dumerilii? If so, that doesn't match the 1c/1d as dorsal head scenario. Also, what is the dorsoanterior ciliary band? Is this the same as the prototroch?

It's difficult to understand what rBMP4 and DMH1 treatment windows were tested and the resulting phenotypes based on the main figure and the manuscript text. The data in Extended Fig. 5 for the difference in phenotypes for 6 hpf and 8 hpf DMH1 treatments and hnf6 expression are convincing. I think the shorter treatments are more relevant for this paper rather than the continuous treatments, so I might swap some of the extended figure data for the shorter treatments with the data for the continuous treatments in the main figure.

Line 304 It's stated that the dorsal trunk expression of bmp2/4, dlx, and smad 6 are reduced, but then later, Line 310, you state that BMP signaling primarily affects dorsal cell lineages in the head. Was the extent of dorsal and ventral expression domains in the head versus trunk for any of the genes after DMH1 or rBMP4 treatment quantified? I think this is necessary to be able to make this statement. Also, are the "animal" views in Fig. 4f of the anterior side of the embryo? Is the focal plan in the trunk or the head? From the lateral images, it does look like there is larger DV shift in gene expression in the episphere versus the trunk, but this is difficult to tell. I would include the lateral diagram from Extended Fig. 4g in Fig. 4 since this was necessary for interpreting the expression patterns.

After 2d ablation, do the resulting embryos/larvae have episphere tissue confined to one side of the AP axis? If so, where is the prototroch in these animals and what tissue is on the other side (i.e., the non-episphere tissue)? Do the animals undergo gastrulation, and where does the trunk mesoderm and endoderm end up? In Fig. 5e, what tissue is in the bottom half of the partial embryos shown from a lateral view? Is this mesodermal tissue? noggin appears to be expressed in ventral episphere but not in the hyposphere/trunk region in the control animal. However, in the animal with 2d ablated, noggin is only expressed in one half of the animal. What is the tissue that does not express noggin (in the bottom half of the lateral image)? If possible, include expression of hyposphere/trunk mesodermal markers and a ciliary marker to show their localization after 2d deletion.

For P. dumerilii, how much rBMP4 protein was used? Is it possible that the rBMP4 treatments were able to cross-activate the Nodal/Activin pathway? Was specificity of perturbation of BMP signaling tested? Has function of Activin/Nodal signaling at the 16-cell stage been tested, and how does this compare with perturbation of BMP signaling?

Line 379 Did SB431542 treatment affect DV axis formation in O. fusiformis?

Line 470 In C. teleta, BMP signaling also promotes formation of 1d micromere identity at the expense of the 1b micromeres (Webster et al. 2021 and 2024), which seems very similar to P. dumerilii. The difference seems to be the function of Activin/Nodal signaling in formation of the episphere tissues.

Methods: Add details of the drug/protein treatments for each species. I could not find the treatment windows or amounts for the different treatments outside of the Suppl Table info, and some of the information was not included there either (e.g., amount or timing of rBMP4 on S. lamarcki). Also, an explanation of how the concentration of drug/protein and the treatment window was selected was not explained. Why was 75 uM U0126 used on S. lamarcki while 20 uM was used for O. fusiformis? Were lower amounts of U0126 not effective at blocking pERK enrichment in S. lamarcki? Were different time windows tried for S. lamarcki?

Fig. 1e–l Add directional arrows or text in the legend to indicate the orientation for the lat and veg views in 1e and other panels so that the position of the future anterior pole is clear. I see the arrowhead for 4d, but it's not clear to me where anterior is. In Fig. 1l, is this a lat view? How do the axes change from 5 hpf through gastrulation? Does gastrulation change where the future anterior tissue is between 5 and 9 hpf? For Fig. 1l, a diagram at 5 hpf and one after gastrulation could be helpful. Also, please add the hpf for the in situs. Are the gastrula-stage animals shown 9 hpf?

Fig. 3 Label the DV axis for the larval images.

Fig. 4 Label the orientation of the images (quadrant for cleavage stages and AP and DV axes for larval stages). Add a diagram or text explaining what developmental stage (e.g., birth of 2d and birth of 4d) occurs at each hpf, e.g., 6, 8, 10, and 12 hpf. Possibly add summary diagrams for 6 and 8 hpf to show which cells express each gene. Add a summary diagram showing the treatment windows and resulting phenotypes for each treatment window.

In Fig. 5, are the animals to the same scale? Are the partial larvae that form after 2d is ablated the same size as the controls? Please add scale bars to the images and add "n's" for how many animals had the same phenotype after 2d ablation. Add an arrowhead or line to indicate where the prototroch cells are.

Suppl Table 2 Add percentages for the phenotypes (same for some of the later Suppl data tables)

Suppl Tables 19–26 are empty

Reviewer #2

(Remarks to the Author)
This is a complex and interesting manuscript that addresses the evolution of axial patterning in Spiralia, a long-standing question in the evo-devo field, for which a lot of laboratories have already provided many puzzle pieces with partially contradicting observations in many species – leading to a so far confusing overall picture. To solve this issue and explain the diverse findings across spiralians, the authors have now undertaken an embryological tour de force involving four different annelid species and a lot of smart choices regarding experimentation, constrained by what is possible in the different non-conventional systems. In their multispecies approach combining Wholemount In Situ Hybridisation (WMISH), pharmacological inhibition of the ERK, BMP and Activin/Nodal signalling pathways, bulk transcriptomics, and blastomere deletions, the authors show conservation of the synergistic ERK1/2 and BMP pathways in DV axis specification in two distant annelids with conditional spiral cleavage, the basal annelid Owenia and the Sedentarian Spriorbis. Given the similar observations in the equally conditional specifier Lottia, this is strong evidence that this situation is ancestral for Spiralia. Their findings also imply that annelids with autonomous axis specification have convergently acquired the use of

Activin/Nodal signaling and restricted BMP patterning via determinants.
In Owenia fusiformis, active BMP4 signaling occurs on the dorsoposterior side, i.e. opposite to the site of expression, which is convincingly demonstrated by clear WMISH and pSMAD1/5/8 antibody staining. They show that SMAD1/5/8 signaling requires FGFR-ERK1/2 signaling, since in the presence of the U0126 inhibitor activation is abolished. In an elegant experiment, they also reveal the downstream effect of BMP signaling regarding specification, in that DMH1 treatment expands foregut and the expression of the oral marker gsc, yet represses the dorsal markers notch-like and BAMBI (leaving the hindgut marker cdx unaffected. This goes in concert with differential analysis of bulk RNA sequencing after DMH1 versus BMP treatment. Furthermore, DMH1 treatment downregulates expression of syt1, elav1 and six3/6 in the apical organ (while expression of the neuropeptide RYamide is upregulated. Similar experiments are conducted in the sedentarian annelid Spirobranchus lamarcki, with similar results, showing that these mechanisms are remarkably conserved.
Next, the authors investigate the role of BMP signaling in Platynereis, which has so far only been marginally addressed, by extensive WMISH analysis of dorsal and ventral markers, and by bulk RNAseq. Here, inhibition with DMH1 leads to circumferential expression of Onecut/Hnf6, which is a neural marker at later stages, and strong upregulation in the RNAseq data. This goes in concert with significant upregulation of neural genes at later larval stages. In an elegant ablation experiment of the 1D versus 2d cell in the Platyereis embryo the authors further localize the dorsalising signal to the 2d cell. This is partially rescued with BMP4 incubation.
Finally, the authors focus on the putative ancestral role of Nodal signaling in annelids, by treating Owenia embryos with the Nodal inhibitor SB431542 and recombinant Activin-A at the time of axial patterning. Interestingly, treatment of Platyereis larvae with Nodal inhibitor does not lead to DV patterning defects but only absence of the developing adult eyes.
The authors document their findings with a rich and clear collection of figures and provide instructive summary schemes to explain their data. In addition, they provide an impressive 10 extended data figures with equal accuracy and elaborate documentation.
Most important, with their broad approach the authors indeed come a new comprehensive characterization, interpretation, and understanding of axis formation in Spiralia. They explain the overall situation as an ancestral pattern undergoing what they call “developmental system drift”, in the context of the (long-known and -debated) repeated transition towards autonomous specification in Spiralia. This contributes a great deal to understand an old basic question of comparative embryology – the phenomenon of conditional versus autonomous specification. The authors are to be congratulated for an impressive developmental opus, which should attract the interest of a broad readership interested in developmental biology.
I have some points and suggestions to improve the manuscript.
1) The authors interprete their U0126 data in that FGFR-ERK1/2 establishes the bilateral symmetry in O. fusiformis embryos by regulating BMP signaling. While it is true that it is required for SMAD1/5/8 activation, it appears that DV patterning is still accomplished by BMP signalling, and FGFR-ERK1/2 signaling rather acts as a gate for this mechanism to be active. Why do you read the data that FRGR-ERK1/2 is doing the actual patterning job? It’s a precondition but does not necessarily convey the spatial information. In a similar direction, why is the BMP specification activity leading to gsc foregut expansion not considered AP specification (or a mix of both)? In a way, co-specification of both axes is included in the authors’ own summary, stating that BMP signaling activates dorsoposterior genes (and sustain Notch/Delta activity) and represses anteroventral fates.
2) Similarly questioning their interpretation, I am not sure whether I would not speak of antineural activity if syt1 and elav expression are downregulated upon DMH1 treatment in Owenia; the continued expression of RYamide is a weak argument in comparison to the synapse and axonal marker gene that disappear. My suggestion is to diagnose antineural activity yet with the exception of a neuropeptide. (Remember, Trichoplax has no nervous system but neuropeptides).
3) Can the authors comment how the adult eyes are missing in Nodal inhibitor SB431542 – treated Platynereis larvae? How about the larval eyes? How does the left-right asymmetry in expression possibly relates to this? Do they observe any left-right effects?

Reviewer #3

(Remarks to the Author)
This is a technically excellent and important study, showing that BMP signaling is patterning the dorsal-ventral axis in Owenia and Spirobranchus, two annelids that have the presumed ancestral mode of spiralian development (“equal cleavage/ conditional specification of the D quadrant”) This is important and clarifying for the field because it shows that these embryos are similar to what is known from molluscs, and thus strongly implies that the ancestor of molluscs and annelids (and thus maybe the spiralian common ancestor) used BMP signaling to pattern the DV axis, along with ERK to specify the axis. Until now, the situation in annelids has been very confusing, with marked differences between groups that have been examined, all of which have the presumed derived mode of spiralian development (“unequal cleavage/ autonomous specification of the D quadrant”). The final part of the paper, which is equally very important, clarifies the role of BMP signaling in one of these latter embryos, Platynereis, and make the results comparable to studies of BMP signaling in other spiralian groups. (There was a paper some time ago that studied the role of BMP signaling in later Platynereis development, but we have not known what the pathways was doing during the earlier phase of spiralian organizer signaling.) This section is further strengthened by 2d organizer cell ablation, which is a key line of evidence for the role of the pathway at these stages. This could easily be 3 very solid papers in good specialist journals—taken together it is a major advance that is definitely appropriate for Nature Communications.

I have some concerns about terminology and making it clear how this work fits in with the existing literature. I am aware that some of this is nuance and likely caused by writing a paper in a non-native language, (I would be incapable of that!). Please take my comments as constructive thoughts on a paper that I strongly support.

Major comments:

First, the ms is not clear, and needlessly confusing, about what is known about the relationship between BMP signaling and the ERK1/2 signaling pathway in spiralians.
line 61-: [After they describe the similarities between dueterostomes and ecdysozoans in terms of BMP patterning of the DV axis]" However, in Spiralia (e.g. molluscs and annelids), the third major lineage of bilaterian animals characterised by an ancestral stereotypical early embryonic program named spiral cleavage, the initial specification of the DV axis ancestrally involved the Fibroblast Growth Factor receptor (FGFR) and ERK1/2 pathways. Notably, an opposing gradient along the secondary body axis of BMP antagonists and pSMAD1/5/8 also occurs in Cnidaria, the sister group to Bilateria. Therefore, an upstream role of the BMP pathway in defining the body symmetry and DV axis is likely ancestral to Bilateria (Fig. 1a), rendering the condition in Spiralia a pronounced and unique yet still poorly understood transition in the 69 mechanisms controlling animal body patterning.".

Similarly, line 86: "Therefore, the ancestral role of the BMP signalling pathway remains contentious in Spiralia, preventing a fundamental understanding of how the shift to an FGFR-ERK1/2-mediated DV axial cue originated during animal evolution."

These statements seem to be making the argument that BMP patterning of the secondary axis is ancestral for Cnidaria+Bilateria, but that in Spiralians it has been lost. More specifically, they are saying that the available evidence suggests that there was a switch between BMP and ERK1/2. However, if BMP was ancestral for bilaterians, and it is known to be patterning the DV axis in two gastropod molluscs (Tritia and Lottia), which they indicate elsewhere, then the obvious interpretation is that BMP patterning of the DV axis was present in the common ancestor of spiralians, and retained in molluscs at least, but lost in the annelids that had been looked at before this work. In addition, since in these two gastropods (Tritia and Lottia) both ERK signaling and BMP signaling are found to be involved in specification and patterning of the DV axis, it does not make sense to hypothesize a "switch" from BMP to ERK. They can both be there. One way they could say this more clearly is simply to say that the role of ERK signaling in the common ancestor of molluscs and annelids was well-supported (thanks to the great 2021 paper from the M-D group, and previous mollusc work), but the role of BMP signaling in that common ancestor is much less clear.

[A minor point here: for the statement above they cite 7-9, the senior author's very nice paper on ERK signaling in Owenia, which makes sense, but also the Tritia BMP paper, and an organizer review, neither of which claim that the ancestral spiralian had ERK signaling instead of BMP, as implied here.]

A related issue, and one that could help resolve and clarify these ideas, is that they are comparing two different kinds of events. BMP signaling seems to have a conserved role in patterning the secondary axis across animals. But it does not have a conserved role in specifying the secondary axis. In at least two molluscs, (and to a reduced extent in Platynereis) BMP signaling does pattern the secondary axis, but it does not specify it. That is true in flies and frogs as well. (In fact in flies, the specification of the axis is also mediated by ERK signaling (!) downstream of gurken and torpedo. And as the authors know well, the specification of the secondary axis in frogs is mediated by the WNT pathway, but then patterned by BMP signaling.) The preceding specification of the secondary axis, or D quadrant, in molluscs usually requires ERK signaling.

I think that making the distinction between the specification of the axis and the patterning of the axis (and being consistent about it) would clarify the paper a great deal.
Minor point related to this: line 55, "In Deuterostomia (i.e., chordates, echinoderms, and hemichordates) and Ecdysozoa (e.g., arthropods), the Bone Morphogenetic Protein (BMP) pathway specifies the DV axis…" The pathway doesn't specify the DV axis in flies or frogs, it patterns it.

Overall, it seems to me that the clearest way to describe the significance of the results in this paper is to say that it was unclear what the ancestral roles of these two pathways were in spiralians, because their known roles in Platynereis and Capitella were so different from molluscs, but examination of Owenia and Spirobranchus has clarified that the ancestral situation in annelids is similar to molluscs.
Once again, it is very clarifying for the field.

Another important semantic issue that I have with this paper is the use of conditional vs autonomous to categorize different spiralian embryos. What I think they are referring to when they use this terminology is the distinction between embryos that use conditional versus autonomous mechanisms to specify the D quadrant. Importantly, even in embryos that seem to use autonomous specification of the D quadrant, most of lineages of the embryo are subsequently patterned by conditional mechanisms. This is probably best understood in Tritia based on cell ablation experiments by Clement, and subsequent molecular work, especially on BMP and ERK signaling. So it is confusing and misleading to call this embryo "autonomous". Further confusing things is the fact that the classic Wilson experiment that defined autonomous specification was done in an embryo that they would call "conditional" (Patella).

The authors could address this by specifically referring to embryos that specify their D quadrants autonomously vs conditionally. Alternatively, the standard in the field is the use of "unequal" to refer to embryos that specify the D quadrant by presumed autonomous mechanism, and "equal" to refer to those that specify by presumed conditional mechanisms. (Equal vs unequal referring to the first two divisions and the subsequent size of the cells at the four cell stage.) The authors do use this terminology in their figures, and I think it is preferable, for two reasons. First, as far as I know, we don't know the mechanism of autonomous specification of the D quadrant in any spiralian—meaning, what is the determinant that is inherited by the presumptive D lineage? And we don't really know the molecular mechanism of conditional specification in any equal cleaving spiralian. Finally, Gary Freeman's experiment, where he mechanically increases the size of one of the macromeres at the 4 cell stage in Lymnaea and it becomes the D cell, indicates that the size alone of the cell is sufficient,

strengthening the case for thinking of the inequality of the sizes could possibly be the key point.
The authors should either switch to “unequal/cleaving” or describe what they mean by conditional vs autonomous embryos more clearly, including consistently using “conditional/autonomous specification of the D quadrant”

Minor comments:
Overall, it feels like there is an unusually large proportion of data in the supplemental/extended data section, and the paper would benefit by moving more to the main text. If they can fit more in the main text under the journal rules, I would urge them to do it. I would also urge the editors to allow more if that is at their discretion.

The authors should be careful about claims about what is ancestral for spiralians based on evidence from molluscs and annelids, because they are more closely related to each other than many other spiralian clades.

Line 95: “This is like what has been described in a mollusc with conditional spiral cleavage12,24”
It is also like what has been described in Tritia, with ERK being activated in 3D, then BMP signaling creating a DV gradient of Smad1/5/8 and being required for normal DV fates. They include the Tritia BMP paper in a later similar statement (line 159).

Lines 267 to 276: I thought smad6 was an inhibitory smad, so it’s not clear why they are including it with “putative targets” vs “antagonists”. It is totally plausible that an inhibitor would also be a target, it just needs to be clarified.

Lines 342-343: Confusing, I had to read a few times to be sure they weren’t describing an experiment where all of these cells were ablated at the same time. Maybe describe phenotype of 2d ablation first and then say you did the other cells and it did not look the same?

377-384: At the end of this section they write: “Therefore, Activin/Nodal is unnecessary to establish the DV polarity in O. fusiformis. Yet, the inhibition of this pathway is reminiscent of the effects of inhibiting the BMP signalling”. Don’t they mean “Yet the ectopic activation of this pathway is reminiscent of the effects of ectopic activation of BMP signaling.”

Fig. 1: The symbol for unequal cleaver is confusing, maybe make one of the cells clearly bigger in this one?

Fig. 2d: This panel is very confusing. Why does control have a phenotype and why is “mock” different from control? And is this figure reporting percentages side by side for different phenotypes for different treatments, as indicated in the text, lines 165 to 169? If so, very confusing. If the only point of this panel is to report the percentages of treated embryos with described phenotypes, I think it is fine to just report percentages in the text.

The discussion on DSD is interesting. It made me think that since these crucial pathways are evolving more quickly than we might have predicted, we need even more sampling to confirm what is conserved vs. derived.

Version 1:

Reviewer comments:

Reviewer #1

(Remarks to the Author)
Overall, the resubmitted manuscript is much clearer, and describing the function of BMP signaling in terms of DV axis patterning vs specification makes comparisons with other taxa easier. The addition of the dose response curves and methodological details of treatments for each drug/protein in each species also satisfies the questions I had about this. The edits to the figures make them easier to understand and more applicable for a broader audience, and the extended descriptions of data from P. dumerilii and S. lamarcki improve the manuscript.

All of my other questions and suggestions have been met through either new data (e.g., dose response curves and additional time points for labeling with pSMAD 1/5/8 or pSMAD and pERK1/2 in Owenia and Spirobranchus, respectively), changes to the manuscript (e.g., adding treatment details to the Methods), or replies in the rebuttal.

This manuscript clarifies the function of signaling systems including BMP that control dorsoventral patterning in annelids, thus illuminating the ancestral role of these pathways across Bilateria. The data are of high quality and approach the question from multiple angles, and the comparisons across three taxa with different modes of organizer specification enable a better understanding of developmental systems drift. Overall, this is a seminal manuscript. I highly recommend publication in Nature Communications and congratulate the authors on their work.

Minor edits

line 39 implies

line 59 symmetry-breaking

line 60 axes; involved

lines 249-250 Fig. 4b

line 252 Fig. 4a

Reviewer #2

(Remarks to the Author)
The authors have carried a thorough revision of the manuscipt, having changed large part of the text and figures and also revising/amending some of their interpretations.
My main concerns / suggestions had been to clarify the role of BMP in the simultaneous co-patterning of both axes, which has been addressed and is now adequately revised by the authors.
Also, they have reconsidered a neurogenic role of BMP in Owenia, based on their observation that inhibition of the pathway leads to a lack of neurogenic markers, in line with a potential proneural role. I am fully satisfied with these revisions and do not raise other points.

Open Access This Peer Review File is licensed under a Creative Commons Attribution 4.0 International License, which permits use, sharing, adaptation, distribution and reproduction in any medium or format, as long as you give appropriate credit to the original author(s) and the source, provide a link to the Creative Commons license, and indicate if changes were made.
In cases where reviewers are anonymous, credit should be given to 'Anonymous Referee' and the source.
The images or other third party material in this Peer Review File are included in the article's Creative Commons license, unless indicated otherwise in a credit line to the material. If material is not included in the article's Creative Commons license and your intended use is not permitted by statutory regulation or exceeds the permitted use, you will need to obtain permission directly from the copyright holder.
To view a copy of this license, visit https://creativecommons.org/licenses/by/4.0/

Response to Reviewers

We want to thank the three reviewers for their insightful and constructive comments on our manuscript, which we believe have significantly improved our work. Following the points raised by the three reviewers, we have extensively reframed the manuscript to contextualise our findings on the role of BMP in patterning rather than specifying the dorsoventral axis and the relationship of this signalling pathway with spiralian cell lineages. We have also performed dose-response analyses for the treatments across all species (reviewer #1 point), added new data on the dynamics of pERK1/2 and pSMAD1/5/8 in *Owenia* and *Spirobranchus* (reviewer #1), and clarified the text regarding *Platynereis* and *Spirobranchus* (reviewer #1), as well as the neural role of BMP in *Owenia* (reviewer #2). In addition, we have moved a significant portion of Extended Data to the main displays and reorganised all figures accordingly. Finally, we addressed all other minor points, text and figure suggestions, and reformatted the manuscript in accordance with the journal's requirements.

Below, we provide a detailed point-by-point response to each reviewer's concern.

Reviewer Comments

Reviewer #1 (Remarks to the Author):

Key results & significance

The paper compares mechanisms of DV axis specification in three species of annelids, two with equal spiral cleavage (Owenia fusiformis, Spirobranchus lamarcki) and one with unequal spiral cleavage (Platynereis dumerilii), in an attempt to reconcile the broad range of molecular mechanisms previously found to affect DV axis formation in different spiralian species. The authors focus on the function of MAPK activation (pERK1/2) in establishing the DV organizer in equally-cleaving embryos and subsequent activation of BMP versus Activin/Nodal signaling to pattern the DV axis. The main results are that in both equally-cleaving animals, activation of ERK1/2 in one cell (4d) breaks symmetry and establishes this cell as the DV organizer. Shortly after ERK1/2 activation, BMP signaling, and to a lesser extent Nodal signaling in O. fusiformis, then establishes dorsal/dorsoposterior tissues. In the unequally-cleaving P. dumerilii, 2d is likely autonomously established and acts as the DV organizer for the head/episphere using BMP signaling to form dorsal tissues; no function for Activin/Nodal signaling in DV axis formation was found. The authors also compare differential gene expression after up- and down-regulation of BMP signaling during cleavage stages in O. fusiformis, P. dumerilii, and another unequally-cleaving annelid, C. teleta, and find very little overlap in BMP responsive genes across the three species. Overall, I think this paper is very interesting and the comparative focus on the molecular mechanism of DV axis formation is relevant for a broad audience of developmental biologists. The authors also ask fundamental questions about how developmental systems can shift over evolutionary time, which is broadly relevant for evolutionary and evo devo biologists.

RESPONSE: Many thanks for the positive assessment of our work.

Validity of results re: conclusions

One question is whether the mechanisms being tested in this paper are involved in DV axis formation versus specification of blastomere fate and/or quadrant identity (e.g., D versus B quadrants) during early cleavage stages in spiralian embryos. The blastomeres ultimately generate the body plan and specific tissues, but the tissues formed by each quadrant aren't partitioned along the DV axis of the eventual larva or the adult of every spiralian species. The data in this paper support a function for MAPK and BMP signaling in breaking symmetry in radially-symmetric, equally-cleaving embryo. MAPK establishes the D-quadrant organizer,

which then uses BMP and/or Nodal/Activin signaling to specify the fate of different blastomeres along a D to B axis. How this relates to the ancestral DV axis is still unclear to me (further comments below). I don't think there is a fate map for O. fusiformis, and perturbation of MAPK and BMP signaling in P. lamarcki seem to affect tissues between the episphere and hyposphere rather than along the DV axis. Furthermore, in P. dumerilii, 1c and 1d micromeres generate dorsal ectoderm in the episphere while 1a and 1b generate the ventral ectoderm. However, for the trunk, I don't think there is a similar division of blastomere fate and ectoderm tissue along the DV axis; 2d and 4d form most of the trunk ectoderm and mesoderm, respectively. I wonder if finding a function for DV axis formation in the head but not trunk in this animal is related to the division of blastomere fate along the DV axis in the head but not trunk. I think a more nuanced discussion of fate maps in different species versus how BMP signaling functions in each animal would be helpful for understanding evolution of the molecular control of DV axis formation. The ancestral spiralian DV axis could have been established by the D (dorsal) to B (ventral) quadrant identities; however, there are many taxa (especially annelids) that do not have this division in the trunk.

RESPONSE: We agree with all these essential observations that align well with reviewers #2 and #3's suggestions to qualify the role of BMP in axial patterning rather than axial specification (see points below). We have now added a paragraph to the discussion on spiralian fate maps, the formation of the dorsoventral axis, and the head and trunk divisions, and how these correlate with BMP activity (lines 531–557). As the reviewer indicates, there is much more variability in fate maps and cell lineages across Spiralia than is often recognised. We highlight the differences in trunk cell lineages between *Capitella* and *Platynereis* and how these might explain the distinct phenotypes observed in these annelids upon BMP inhibition. We argue that dorsoventral patterning might not be strictly determined by cell lineages but rather by the activity of signalling pathways on certain embryonic regions, whose cell lineage composition might change between species.

For the comparison of DEGs between rBMP4 and rActivinA treatments in O. fusiformis, the results are difficult to interpret since different concentrations of the two proteins (1000 versus 75 ng/mL) were used. Both proteins are known to activate different sets of genes at different concentrations in other animals; the amount of rBMP4 could have been maximally activating while the amount of rActivinA could have been a low concentration. Similarly, for the two drugs (DMH1 and SB431542), you could have been maximally inhibiting a pathway with one drug and only partially inhibiting with the other drug. Without dose response curves for the proteins and drugs and quantification of pSMAD2/3 and pSMAD1/5/8 levels, I think caution is needed when directly comparing the DEGs for the different treatments within the same animal. This same problem holds for the comparisons between species. At the very least, I think assessment of one DV phenotype at different concentrations of rBMP4 is needed for O. fusiformis and P. dumerilii.

RESPONSE: Thanks for pointing this out. All drug treatments were performed with the lowest drug concentrations that maximise phenotypic penetrance, with the shortest time windows that pSMAD1/5/8 dynamics and/or our understanding of axial patterning in the three systems suggested (e.g., treating between 4 and 6 hpf in *Owenia*, when the axial organiser becomes specified and SMAD1/5/8 becomes active). Following the reviewer's comment, we have now generated dose-response curves for all treatments to support our original approach, and, as suggested below, we have included three paragraphs in the Methods section clarifying the concentrations and time windows used in the manuscript (lines 623–650). The new data is included in Extended Data Figures 2, 4, and 6.

The results and conclusions presented for O. fusiformis are the most convincing. The descriptions of the data from S. lamarcki and P. dumerilii are less clearly explained, making

these data sets more difficult to interpret (see comments below). I think this can be fixed with text edits that clarify how the experiments were done and explain the figures in more depth. Adding labels and diagrams to the figures could also improve accessibility for readers not familiar with spiralian development.

RESPONSE: We have clarified and extended the interpretation of the data from *S. lamarcki* and *P. dumerilii* in response to the edits and minor questions suggested below (see below for specific details). In addition, we have moved two Extended Data figures with *Platynereis* data to the main text (per reviewer #3's suggestion) and reorganised all this data into three new main displays (Figures 5–7), which we believe provide more context for all the work and conclusions on this species.

Suggested edits/minor questions

Would be helpful to add text about the results in Owenia relative to the quadrants after 4d specification. This would help with comparisons to other spiralian taxa.

RESPONSE: As indicated above, we have included a paragraph in the Discussion putting our data in the context of the quadrant and blastomere fates (lines 531–557).

Line 34 "implies"

Line 51 "of known animals"

RESPONSE: These two points have been changed as suggested.

Line 119 The region of overlap between bmp2/4 and chordin is difficult to see in the lateral blastula stage images. The two patterns look non-overlapping in the images shown.

RESPONSE: We have updated the images in Figure 1 with clearer gene expression patterns, indicating the region of expression of *bmp2/4* at 6 hpf with a dotted line, and toned down the text (line 119: "At that stage (9 hpf), *bmp2/4*, *bmp5-8*, and *chordin* seem to be restricted to non-overlapping, staggered expression domains…"). We also tried FISH and HCR approaches for these genes to simultaneously colocalise their expression, but the attempts were unsuccessful because they are low-expressed and have weak probes.

Line 135 Based on the diagram in Fig. 1g, it appears that pSMAD1/5/8 is present in ectoderm, mesoderm, and endoderm precursor cells at 6 hpf. Do these pSMAD1/5/8+ cells go on to form dorsoposterior tissues in each germ layer, or do only the ectodermal precursor cells form dorsoposterior tissue in the larva? (And how does this relate to the juvenile axes?)

RESPONSE: This is a great question. We have now performed pSMAD1/5/8 immunostaining in elongating embryos (13 hpf), early larvae (18 hpf), and fully developed mitraria (24 hpf). We detected active SMAD1/5/8 in all stages on the dorsal ectoderm and endoderm, as well as in the posterior foregut (in the connection between the foregut and midgut). Whether it is also active in mesodermal derivatives is difficult to assess without co-labelling (unfortunately, the pSMAD1/5/8 antibody staining is not compatible with phalloidin). Nonetheless, this new data indicates broad activity of pSMAD1/5/8 across germ layers and in what would, at least for the gut, correspond to the juvenile dorsal side (the larval gut is preserved during metamorphosis, but the dorsal larval epithelium is reabsorbed). This is now in Figure 1h and mentioned in the main text (lines 134–137: "This pattern remains as gastrulation proceeds, with pSMAD1/5/8 remaining enriched in the progeny of the embryonic organiser, the dorsoposterior half of the embryo, as well as the larval dorsal gut and ectoderm, the posterior foregut and oesophagus (Fig. 1h).").

Line 151 Given that the SMAD1/5/8 homolog in Owenia does not have the ERK phosphorylation sites present in deuterostomes, how do you think the organizer regulates SMAD activity? How do you think U0126 blocks phosphorylation of SMAD1/5/8 in Owenia?

RESPONSE: This is an excellent point. At 5 hpf, pERK1/2 is only active in the 4d cell, but pSMAD1/5/8 is active in the 4d and other D-quadrant blastomeres. This indicates that there must be pERK-independent mechanisms to activate SMAD1/5/8 in *Owenia*. We do not know what these are, and we are excited to investigate further. We envisage two possibilities: (i) this is *chordin*-mediated and the symmetry-break triggered by pERK1/2 leads to an asymmetry in *chordin* and, thereby, in BMP signalling; (ii) Notch/Delta contributes to this asymmetry. Together with the activation of pERK1/2 in 4d, *delta* is also upregulated in the 4d cell, and Notch signalling is essential for normal DV patterning (Seudre, Carrillo-Baltodano *et al* 2022). We now discuss this point more extensively in the text (lines 160–164).

Line 154 In the U0126 treatments from 4–6 hpf in Ofu, was 4d specified? Does treatment during this time window block pERK in 4d? There are arrowheads indicating 4d in Fig. 1i, j, so it looks like 4d is morphologically visible.

RESPONSE: Yes, U0126 treatment from 4 to 6 hpf blocks pERK1/2 and 4d specification, as published in Seudre, Carrillo-Baltodano *et al* (2022). The arrowheads in Figure 1i, j indicate the 4d in the control samples (top rows) to highlight the loss of that identity in U0126-treated embryos (bottom rows).

Line 155 Did U0126 treatment affect bmp5-8 expression in O. fusiformis?

RESPONSE: It does not, according to our RNA-seq expression data after pERK1/2 inhibition at 5.5 hpf (Seudre, Carrillo-Baltodano et al 2022). We now mention this in the text (lines 156–157: "Interestingly, *bmp5-8* is not downregulated at the blastula stages after pERK1/2 inhibition[8].").

In L. goshimai (Tan et al. 2021), U0126 treatment causes expression of chd in all 3Q macromeres; chd is normally not expressed in the organizer 3D. In O. fusiformis, U0126 treatment appears to affect chd expression in the ectoderm but not the endomesoderm. Is this correct? Do all 4Q macromeres and 4q micromeres express chd after U0126 treatment in Owenia (Fig. 1)? Is MAPK signaling from the organizer inhibiting bmp2/4 expression on the future dorsal side of the embryo in Owenia?

RESPONSE: As shown in Figure 1j, *chordin* expression is lost in the blastula ectoderm, but not in the presumptive gastral plate (at that time consisting of 5Q and 4q progeny; Figure 1g and see Seudre and Carillo-Baltodano et al 2022 blastomere-fate annotation of the vegetal pole), after U0126 treatment. However, this gastral plate expression is transitory, as it disappears with gastrulation (Figure 1e). The ectodermal loss of expression in the blastula is consistent with the loss of expression in the posteroventral mouth ectoderm in the U0126-treated larva (Figure 1k). As shown in those two figure panels, U0126 also completely inhibits *bmp2/4*'s expression in both the blastula and larva. This would also be consistent with the two possible scenarios of how pERK1/2 regulates BMP signalling (see above) because the inhibition of 4d specification blocks Notch-delta signalling, and the loss of ectodermal expression of chordin might interfere with potential ligand-inhibitor interactions that may create the pSMAD1/5/8 dorsoventral gradient at this stage.

Line 164 For these experiments, you used 1000 and 2000 ng/mL recombinant BMP4 protein, which is a very high concentration compared to other animals. Did you try lower concentrations and did those affect pSMAD 1/5/8 levels? Why do you think so much rBMP4 protein was needed to see an effect?

RESPONSE: We have now performed a broader dose-response curve for this treatment, demonstrating that rBMP4 affects dorsoventral patterning at lower concentrations (Extended Data Figure 2b). However, a penetrant phenotype with a more pronounced effect on axial

patterning is observed at 1,000 ng/mL, the concentration we used in downstream analyses. This is now clarified in the Methods section (lines 623–650) and main text (line 170).

Line 166 Is the chaetal sac posterior or ventroposterior tissue? Based on the morphological descriptions, it seems like blocking BMP signaling does not cause a radialized phenotype while ectopic BMP signaling does. Wouldn't you expect radialized phenotypes for both blocking and upregulating BMP signaling? Is it possible that DMH1 is only partially blocking BMP signaling while the amount of rBMP4 protein added is maximally activating? Even though there is a loss of pSMAD1/5/8 antibody labeling with DMH1 treatment, there could still be non-detectable but biologically-relevant levels of pSMAD.

RESPONSE: If we consider the anus as the terminal posterior end of the larva, as it is in the adult (and the larval gut is the adult gut after metamorphosis), the chaetal sac is part of the posterodorsal tissue. This is consistent with the new pSMAD1/5/8 immunostainings in post-gastrula embryos and larvae (Figure 1h) and the expression of dorsal (e.g., *Notch-like*; Figure 2g) and posterior markers (e.g., *wnt1* and *evx*; Martin-Duran *et al* 2017; Seudre, Carrillo-Baltodano *et al* 2022). Because pSMAD1/5/8 is downstream of pERK1/2 (hierarchical and temporally), the DMH1 treatment does not affect the initial symmetry breaking event, and thereby, the larvae develop with anteroposterior symmetry but lacking the dorsal territories (no chaetal sacs and dorsal ectoderm as revealed by *Notch-like* and *BAMBI* in situ hybridisation; Figure 2g). In other words, the DMH1 treatment does not affect the initial specification of the quadrants and anterior vs posterior, but the subsequent patterning/development of a certain part of the D quadrant. Because we start the rBMP4 treatment slightly before the pERK1/2-mediated symmetry-break, pSMAD1/5/8 becomes active in all quadrants, even when only one gets pERK1/2, thereby acquiring a D-quadrant fate and the larvae developing more radially. However, there are slight asymmetries (much more evident at lower rBMP4 doses; see new Extended Data Figure 2b). Not all chaetal lobes are equal, and *cdx* is more asymmetrically expressed (e.g., Figure 2f), as expected. Nonetheless, we now indicate that we cannot exclude complete inhibition of pSMAD1/5/8 with our treatments (line 178–179: "…DMH1 exposure leading to a complete inhibition of SMAD1/5/8 (although we cannot exclude remaining non-detectable but biologically-relevant levels of pSMAD1/5/8)…"), and indicate the rBMP4 phenotype is not radialise (lines 185–186: "(but not of *cdx*, which tends to localise slightly asymmetrically on one side)").

Line 185 Similar to the comment above, why do you think there were so many more DEGs detected in the BMP-treated embryos versus the DMH1-treated embryos?

RESPONSE: As explained in the comment above, the rBMP4 treatment is more radical than DMH1 inhibition of BMP signalling, as the former leads to a transformation of all quadrants to a common fate, while the other only impacts the normal development of a portion of the D-quadrant (mostly the ectoderm, since the 4d, which is the endomesodermal precursor, is specified). Therefore, the differences in differentially expressed genes (DEGs) between the two treatments are consistent with their expected roles. In addition, we collected samples for RNA-seq analyses near the timing of signalling activation (6 hpf) and speculate that we would have seen more DEGs in DMH1 had we allowed more developmental time. We now clarify this (line 198–201: "This difference in the number of differentially expressed genes is consistent with the broader impact of rBMP4 on the embryo, which enriches pSMAD1/5/8 across all quadrants (Fig. 2e), and with the fact that samples were collected at 6 hpf, when BMP signalling starts and, therefore, only the earliest response genes might be active.").

Line 228 Spell out genus when start with same letter and in same sentence.

RESPONSE: Corrected.

Line 234 In S. lamarcki, one cell has pERK at 6 hpf, which is assumed to be 4d. Does this cell divide later than the other 4q micromeres (as in Owenia)? Is this cell larger than the other 4q micromeres? Was pERK detected in other blastomeres before 6 hpf? What are the first morphological asymmetries visible during spiral cleavage in this animal?

RESPONSE: These are excellent points. We do not detect pERK1/2 before 6 hpf, but we now include data on pERK1/2 labelling from 6 to 9 hpf (Figure 4c). At those later stages, we detect enrichment in only one cell, suggesting that the specification of the 4d cell might create a cell-division asymmetry, as in *Owenia*. However, it is difficult to identify other asymmetries given the small size of *Spirobranchus* embryos. Nonetheless, our data warrant future in-depth investigations of spiral cleavage in these species. We mention these new findings in lines 252–254: "Enrichment in a single cell continues as the embryo undergoes gastrulation, suggesting that, as in *O. fusiformis*, the specification of the 4d cell creates a cell division asymmetry[8] (Fig. 4c).".

Line 238 Use past tense verbs for treatments

RESPONSE: We have amended the manuscript accordingly.

Line 238 What was the treatment window for U0126? Were any other treatment windows tested? Same question for DMH1 and rBMP4 treatments.

RESPONSE: As mentioned above, we now include three paragraphs in the Methods section (lines 623–650) that provide all the information on treatment windows and concentrations, supported by dose-response assays.

Line 239 DMH1 treatment is stated to 'not affect di-pERK1/2 enrichment in 4d'; however, in Suppl. Table 17, 71% of the DMH1-treated embryos were reported to have pERK while 88% of the controls had pERK. I think a statistical analysis is needed to test if these two treatments are different.

RESPONSE: Following the reviewer's suggestion, we triplicated the DMH1 and control treatments, assessed pERK1/2 enrichment, and counted embryos with and without enrichment in the 4d cell. We confirmed that there is no significant change. This is now added in Figure 4f and in the text (line 266–267: "…the inhibition of the BMP signalling with DMH1 did not statistically affect the dpERK1/2 activation and the specification of the 4d…").

In P. lamarcki, which blastomeres have pSMAD1/5/8? Is pSMAD1/5/8 detected before 6 hpf? Does expression in the D quadrant (or future dorsal?) persist past 6 hpf? Does pERK1/2 proceed pSMAD1/5/8 as in O. fusiformis?

RESPONSE: As with pERK1/2, we did not detect pSMAD1/5/8 before 6 hpf (we tested 4 and 5 hpf), and the asymmetric activation persists after 6 hpf in what seems to be the D quadrant, at least at 7 hpf. In later stages, asymmetric expression also occurs, but given the small embryo size, orienting the embryo and discerning the exact identities of the blastomeres without markers (see below) are challenging. We have added this new data in Figure 4c and in the main text (lines 254–257: "Likewise, as in *O. fusiformis*, pSMAD1/5/8 is asymmetrically enriched in the 4d cell, and one side of the embryo (the putative dorsoposterior side) at 6 hpf in the embryos of *S. lamarcki*, and this asymmetric localisation continues during the early stages of gastrulation (Fig. 4c).").

Line 244 What was the pSMAD1/5/8 pattern after rBMP4 treatment in P. lamarcki?

RESPONSE: Thanks for the question. pSMAD1/5/8 was not affected after rBMP4 treatment in *S. lamarcki*, which is consistent with the reported phenotype displaying only minimal defects (Figure 4e; e.g. the neurotroch is still present in the hyposphere). This is intriguing, as it might indicate species-specific differential sensitivity of the BMP receptor to its ligand, and is worth exploring in the future. We have now included the new data in Extended Data Figure 4c and in

the main text (lines 277–280: "Accordingly, rBMP4 treatment did not result in ectopic pSMAD1/5/8 activation, even at high concentrations (Extended Data Fig. 4c; Supplementary Table 22), indicating species-specific sensitivities of the BMP receptor to its ligand in annelids.").

Line 244 In Fig. 3e, both the episphere and hyposphere appear reduced relative to the controls in P. lamarcki, not just the episphere. Was this quantified? Is the reduction for the episphere along the AP and/or DV axis? Were there specific tissues missing in the episphere? In Fig. 3e, the apical tuft is labeled in the rBMP4-treated animal suggesting that this tissue is still present. Was this quantified? Were there any DV phenotypes noted for the rBMP4 treatment (or the DMH1 treatment)?

RESPONSE: To assess this point, we cloned and generated riboprobes for known axial and neuronal markers in other annelids, including those we use in *Owenia* (i.e., *cdx*, *gsc*, *six3/6*). Unfortunately, repeated trials produced unreliable staining in the tiny *Spirobranchus* larvae, mainly due to what we suspect is background staining in the mouth (see Figure below). The only two dorsoventral anatomical asymmetries we could identify are a dorsal FMRFamide+ ganglion in the episphere and the ventral neurotroch in the hyposphere. Interestingly, DMH1 treatment removes the dorsal FMRFamide+ ring around the gut in the episphere, supporting that dorsoventral patterning is also affected in the anterior region and that the phenotype is probably not a defect in epi- versus hyposphere patterning (see point below). This is now in Figure 4g, h and in lines 271–275.

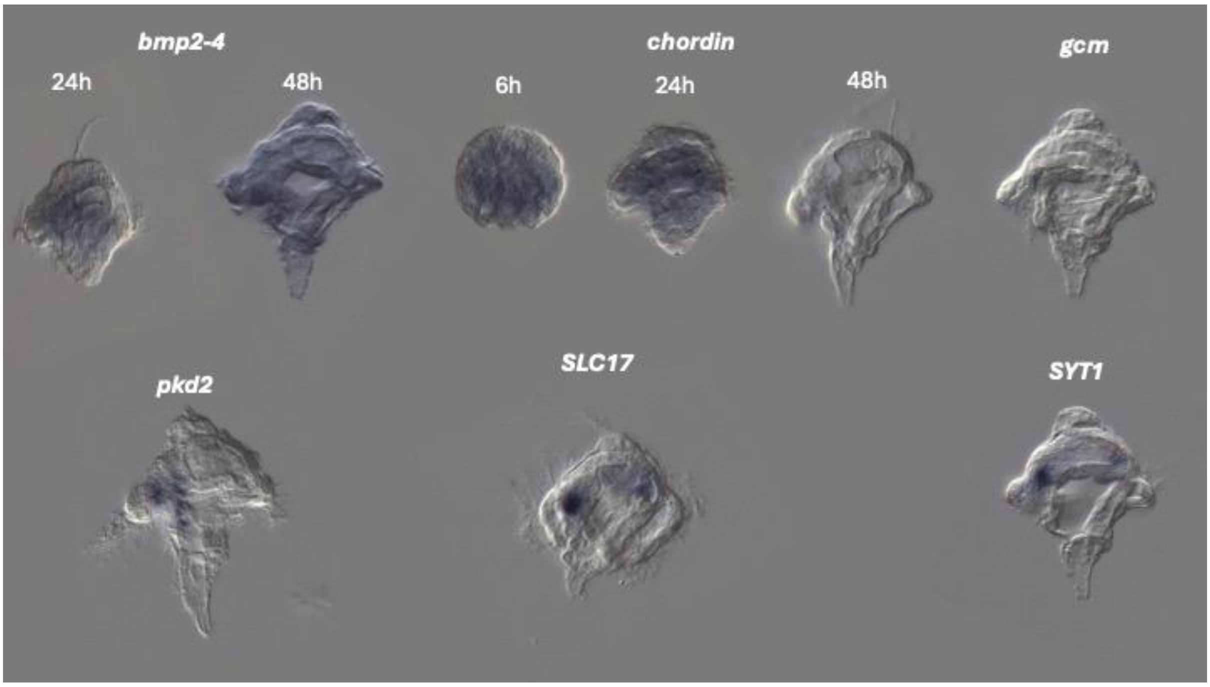

Figure 1 – Gene expression data for Spirobranchus. Whole-mount *in situ* hybridisation of selected marker genes with obvious signal in embryonic and larval stages. In most cases, the signal localises to the mouth, suggesting this might be a probe-trapping area.

Line 246 It's stated that BMP signaling establishes the DV axis in S. lamarcki; however, the reported phenotypes after DMH1 and rBMP4 treatment appear to be along the AP axis or between the episphere (anterior?) and hyposphere (posterior?). If there are clear affects on tissues localized along the DV axis, there needs to be an explanation and demonstration of these. As presented, the data do not support a function of BMP signaling in DV axis formation. Addition of a diagram of the larva with labeled tissues and axes after the different treatments (cont, U0126, DMH1, rBMP4) could be helpful.

RESPONSE: We agree with the reviewer that an anteroposterior role for BMP cannot be completely ruled out in *Spirobranchus*. However, given the known role of BMP in other systems (particularly in other equal spiral-cleaving annelids and molluscs), the loss of the dorsal FRMFamide+ ring in the episphere (see point above), and the fact that pSMAD1/5/8 is downstream of pERK1/2 and that there is a more obvious anteroposterior polarity after DMH1 treatment, all as observed in *Owenia*, we believe it is more parsimonious to hypothesise that the axial role of this signalling pathway is in dorsoventral patterning and not a newly-acquired, species-specific, divergent role in anteroposterior development. Moreover, as in *Lottia goshimai* and likely *O. fusiformis*, asymmetric BMP signalling in the dorsal D quadrant is probably essential for axial elongation, which, at least in some embryos, seems to be driven by the displacement of dorsal D quadrant cells to the ventral midline (e.g., Damen and Dictus, 1997; van den Biggelaar *et al*, 2002) and could explain the anteroposterior defects the reviewer points out. At the end, as reviewer #2 highlights (see below), anteroposterior and dorsoventral development are tightly linked in these embryos. Nonetheless, we now tone down our interpretations and indicate that further analyses (hopefully involving the troubleshooting of gene expression analyses in this annelid) are required to fully validate our hypothesis (line 280–285: "Therefore, ERK1/2 signalling establishes the bilateral polarity in *S. lamarcki*. Although a role in patterning the epi- and hyposphere cannot be completely ruled out, our findings are consistent with a role of the BMP pathway in patterning the DV axis in *S. lamarcki*, as in *O. fusiformis*, thereby indicating an ancestral role of this pathway in dorsoventral patterning in Annelida. Future analyses of gene expression of axial polarity markers will further clarify the precise axial functions of these signalling pathways.").

Is there a chordin and/or chordin-like homolog in S. lamarcki? If so, where is this gene(s) expressed during cleavage stages? I think this information would be an interesting addition to the paper since the regulation of chordin expression is an important part of DV axis formation in L. goshimai (and vertebrates) but apparently not in O. fusiformis.

RESPONSE: We agree with the reviewer that this would be an interesting addition. *Spirobranchus* has a chordin orthologue, suggesting greater conservation of DV patterning mechanisms across these annelids and many equal cleavers. We now comment on this (line 257–259: "Interestingly, *S. lamarcki*, as *O. fusiformis,* but unlike other unequal spiral cleaving annelids, has retained a *chordin* ortholog in its genome."). We could not identify a *chordin-like* orthologue, but available transcriptomic resources are limited, and therefore, we cannot exclude that it is still present in the genome. Unfortunately, we were unable to establish standard *in situ* hybridisation methods for this species within the time given to complete these revisions.

Line 258 Not all unequally-cleaving spiralian embryos have the same contributions to dorsal versus ventral ectoderm in the episphere. For example, in Ilyanassa obsoleta most of the dorsal-left and dorsal-right tissue in the episphere is generated by 1a and 1c, respectively, while 1b forms most of the ventral tissue in the episphere. Also, the contributions to dorsal versus ventral tissue are for the ectoderm but not mesoderm in the head.

RESPONSE: Thanks for pointing this out. We have amended the start of the sentence accordingly (line 295: "As in other annelids with unequal cleavage,…").

Line 261 I'm not sure I understand this as written. Is this statement for P. dumerilii, unequally-cleaving annelids, or unequally-cleaving spiralians? In annelids, 2d and 4d generally form all or most of the ectoderm and mesoderm of the hyposphere, respectively. I am not aware of fate maps for annelids that show that 2d and 4d generate dorsal tissue rather than ventral tissue in the trunk.

RESPONSE: We have clarified this statement in line with the reviewer's suggestion: "In subsequent cell divisions, the largest macromere 1D generates two uniquely large blastomeres —2d and 4d— which demarcate the dorsal side of the trunk at an early developmental stage before eventually forming most of the larval trunk ecto- and mesoderm, respectively (Fig. 5a)." (line 298–301).

Line 262 I don't see how early cell divisions define the DV axis in the trunk of P. dumerilii. This needs to be explained. Also, in the Pfeifer et al. 2014 paper, they used 10, 25 and 50 uM U0126 from 13.5 to 16.5 hpf, which I think is after 2d and 4d are born, making these treatments not comparable with the current study. Have earlier U0126 treatments been carried out in Pdu? Pfeifer et al. did not report seeing pERK in 2d, 4d or 3D, but they could have missed low levels of pERK at early cleavage stages, so the statement that DV patterning "arises independently of ERK1/2 activity" doesn't seem to be strongly supported.

RESPONSE: We agree with the reviewer and have removed this statement. Likewise, our study warrants (and lays the foundation for) a detailed investigation into how pERK activity might interact with and regulate BMP signalling during early cleavage in *Platynereis*.

Line 263 "P. dumerilii"

RESPONSE: Amended.

Line 270 Is bmp2/4 expressed in 1a and 1b or only in 1c and 1d at 6 hpf? Which cell(s) express dlx at 6 hpf? I think it's necessary to state which cells express each gene at least at the 6 and 8 hpf stages rather than saying that the genes are expressed "dorsally" or "ventrally". In the animal views it's difficult to tell where the quadrant boundaries are. Are the C and D quadrants at the top of the figure panel? Are the "dorsal" views lateral images with the D quadrant centered?

Line 271 Similar to the comment above, it's not clear to me which blastomeres are being considered "ventral trunk" precursors. Are bmp 3 and noggin expressed in A and B quadrant micromeres? Is the B quadrant centered in the lateral views in Fig. 4c?

RESPONSE: As suggested in these two points, we now specify which cells express the dorsoventral genes (*bmp2/4*, *smad6*, *dlx, noggin and bmp3*) in early stages (lines 309–317) and provide more information on the orientation of each photo (see figure captions).

Does P. dumerilii have chordin-like and gremlin homologs? What are their expression patterns during cleavage? Where is bmp5-8 expressed during cleavage stages?

RESPONSE: We identified a *chordin-like* ortholog in *Platynereis,* but, unfortunately, it seems to be expressed at low levels, and we were unable to clone it and analyse its expression. We could not identify a *bona fide gremlin*. *Bmp5-8* is expressed rather ubiquitously at early stages and thereafter more concentrated on the dorsal side (but also expressed ventrally and in a segmental pattern). We include this expression in Extended Data Figure 5g and mentioned it in the text (lines 319–321: "As in *O. fusiformis*, *bmp5-8* shows an early ubiquitous expression that concentrates more on the dorsal side of the larva as development progresses (Extended Data Fig. 5g).").

Line 281 Why were the DMH1 and rBMP4 treatments done for such a long time window (i.e., from 2 hpf to 72 hpf)? I think treatments only spanning birth of 1c and 1d, birth of 2d, and birth of 4d are needed for comparison with the other two species. Later, it's mentioned that shorter treatments were used, but the time length relative to the developmental stages is not explained for each of the treatments.

RESPONSE: At the start of this study, we did not know when the organising activity during early development occurred in *Platynereis*. Therefore, we took a conservative approach and performed continued treatments. We do not think this significantly affects the comparison with

the other species because, as our ablation data demonstrates, axial patterning occurs early (2d cell-mediated). Thus, the phenotype we observed even at 72 hpf is influenced by that initial event, as we confirmed with the shorter time windows. Nonetheless, we agree with the reviewer that, in hindsight, such long treatments were probably unnecessary and could have been shortened. Following the reviewer's suggestion below, we now show the shorter windows in the main Figure and have also written an extra paragraph in the Methods section detailing all treatment information (lines 623–650).

Line 282 I don't understand how the described morphological phenotypes after DMH1 treatment are "ventralized". Please explain. In the diagram in Fig. 4a, the eyes are depicted as dorsal. Are the larval and adult eyes generated by 1a and 1c in P. dumerilii? If so, that doesn't match the 1c/1d as dorsal head scenario. Also, what is the dorsoanterior ciliary band? Is this the same as the prototroch?

RESPONSE: The clearest morphological landmark supporting a ventralisation of the head after DMH1 treatment is the expansion of the ventral gland cells, as revealed by *hnf6* expression (as well as by the expansion of the expression of ventral marker genes and the loss of expression of dorsal markers). Furthermore, the dorsal cephaloblasts also give rise to the two nephroblasts (1c11221 and 1d11221), and akrotroch cells (dorsoanterior band), both of which are lost in DMH1-treated 24 hpf and 72 hpf embryos (Extended Data Figure 7a, b). These observations supporting a ventralised phenotype are explained in lines 340–343.

Regarding the eyes, the situation is a bit more complex. The adult eyes form from the dorsal cephaloblasts (1c112 and 1d112), and thus their loss is also indicative of a ventralisation. Interestingly, the larval eyes, which are also lost in their original position, form from the most anterior lateral progeny of two intermediate girdle cells (1a12 and 1c12). The left larval eye pigment cell (1a1211211), and the left larval eye photoreceptor cell (1a1211212); and the right larval eye pigment cell (1c121121b), and the right larval eye photoreceptor cell (1c121121a) according to Vopalensky et al, 2019. However, the larval eyes form in cells in close proximity between the progeny of ventral and dorsal cephaloblasts, and thus they might require exposure to two opposing BMP signalling levels to become specified (which is lost after DMH1 treatment). Interestingly, after DMH1 treatment, a single eye (we assume a larval eye) is ectopically formed on the dorsal midline at the most posterior position of the episphere. As indicated to reviewer #2 (see below), more work is needed to precisely determine the signalling interactions between BMP and Activin/Nodal that define the position of the larval and adult eyes.

It's difficult to understand what rBMP4 and DMH1 treatment windows were tested and the resulting phenotypes based on the main figure and the manuscript text. The data in Extended Fig. 5 for the difference in phenotypes for 6 hpf and 8 hpf DMH1 treatments and hnf6 expression are convincing. I think the shorter treatments are more relevant for this paper rather than the continuous treatments, so I might swap some of the extended figure data for the shorter treatments with the data for the continuous treatments in the main figure.

RESPONSE: We have amended the main Figure to include the shorter treatments. Information on the treatment windows tested is now in an extra paragraph in the Methods section (see above).

Line 304 It's stated that the dorsal trunk expression of bmp2/4, dlx, and smad 6 are reduced, but then later, Line 310, you state that BMP signaling primarily affects dorsal cell lineages in the head. Was the extent of dorsal and ventral expression domains in the head versus trunk for any of the genes after DMH1 or rBMP4 treatment quantified? I think this is necessary to be able to make this statement. Also, are the "animal" views in Fig. 4f of the anterior side of the embryo? Is the focal plan in the trunk or the head? From the lateral images, it does look like

there is larger DV shift in gene expression in the episphere versus the trunk, but this is difficult to tell. I would include the lateral diagram from Extended Fig. 4g in Fig. 4 since this was necessary for interpreting the expression patterns.

RESPONSE: As suggested, we now include the lateral diagram in the former Extended Data Figure 4g in the main display (new Figure 5d). Regarding the expression of dorsal and ventral markers in the trunk, as shown in Figure 6f, dorsal genes are still expressed in the hyposphere/trunk after DMH1 treatment, in some cases (e.g., *smad6*) with a reduced pattern, but not in others (e.g., *bmp2/4* and *dlx*). This indicates that, unlike in the episphere, the dorsoventral patterning is not completely distorted in the hyposphere. However, as the reviewer suggests, without an exhaustive quantification, we cannot completely exclude an effect in the trunk. We now indicate this in the text (line 355–359: "Altogether, our findings demonstrate that BMP signalling is essential for DV patterning during early spiral cleavage in *P. dumerilii*. However, the head and trunk respond differently to this morphogen, with BMP signalling required to specify dorsal cell lineages in the head, but signs of dorsoventral patterning remain visible in the trunk after inhibiting this pathway."). We now also indicate that the animal views in Figure 6f, g are at the level of the progeny of the first quartet micromeres, with the 1c/1d to the top (lines 1102–1103).

After 2d ablation, do the resulting embryos/larvae have episphere tissue confined to one side of the AP axis? If so, where is the prototroch in these animals and what tissue is on the other side (i.e., the non-episphere tissue)? Do the animals undergo gastrulation, and where does the trunk mesoderm and endoderm end up? In Fig. 5e, what tissue is in the bottom half of the partial embryos shown from a lateral view? Is this mesodermal tissue? noggin appears to be expressed in ventral episphere but not in the hyposphere/trunk region in the control animal. However, in the animal with 2d ablated, noggin is only expressed in one half of the animal. What is the tissue that does not express noggin (in the bottom half of the lateral image)? If possible, include expression of hyposphere/trunk mesodermal markers and a ciliary marker to show their localization after 2d deletion.

RESPONSE: Yes, 2d-ablated embryos have an episphere that is separated from the rest of the embryo (2a, 2b, 2c, 3n, 4n and 4N progeny) by the forming prototroch. These embryos gastrulate and have trunk mesoderm (developed from 4d) and endoderm (from 4N) (see new Figure 7e, f), although 2d ablation seems to affect axial elongation (unlike with the ablation of 2D, 3d and 4d) (Figure 7b). We now clarify this (lines 398–400: "2d-ablated embryos exhibit an epi- and hyposphere separated by a prototroch. However, 2d ablation impaired axial elongation, unlike after 2D, 3d, or 4d cell ablation (Fig. 7b)."). We also indicate the position of the prototroch in Figure 7.

Regarding *noggin* and the expression of trunk markers, the tissue that does not express *noggin* is the hyposphere/trunk, which has not elongated. This is more obvious in *nk2.1* (Figure 7e), which, in control embryos, is expressed in the ventral episphere and hyposphere, with the two domains clearly separated by the prototroch. After 2d ablation, the ventral hyposphere/trunk expression is as in the control, but the episphere expression is ventralised, with both domains still separated by the developing prototroch.

For P. dumerilii, how much rBMP4 protein was used? Is it possible that the rBMP4 treatments were able to cross-activate the Nodal/Activin pathway? Was specificity of perturbation of BMP signaling tested? Has function of Activin/Nodal signaling at the 16-cell stage been tested, and how does this compare with perturbation of BMP signaling?

RESPONSE: We have now done dose-response assays for rBMP4 and DMH1 treatments, as suggested in a point above. The function of Activin/Nodal was tested through continued treatment during early development, as shown in Figure 8a, b and described in the text (lines

471–472), without affecting dorsoventral polarity. Unfortunately, as indicated in our original manuscript, pSMAD1/5/8 immunostainings do not work well in *Platynereis* and pSMAD2/3 (the readout of the Activin/Nodal pathway) antibodies do not seem to work in the spiralians tested so far, including *Owenia*, making it difficult to explore how these two signalling pathways might influence each other upon disruption. Nonetheless, the fact that inhibition of Nodal/Activin does not cause an apparent dorsoventral defect, whereas inhibition/overactivation of BMP does, suggests that both DMH1 and rBMP4 are likely acting through the same receptor.

Line 379 Did SB431542 treatment affect DV axis formation in O. fusiformis?

RESPONSE: We now include the expression of the dorsal marker *Notch-like* in control and SB431542-treated embryos, demonstrating that it is reduced but still present in the treated samples. This suggests that Activin/Nodal does not interfere with dorsoventral specification or patterning but contributes to the normal axial development (in line with what can be inferred in *Platynereis*; see response below to reviewer #2's comment). We now include this in the main text (lines 433–434) and in Figure 8h.

Line 470 In C. teleta, BMP signaling also promotes formation of 1d micromere identity at the expense of the 1b micromeres (Webster et al. 2021 and 2024), which seems very similar to P. dumerilii. The difference seems to be the function of Activin/Nodal signaling in formation of the episphere tissues.

RESPONSE: We now include this observation and references (lines 565–566: "Yet, BMP signalling seems to promote the establishment of the 1d micromere identity at the expense of the 1b micromeres in both species[56].").

Methods: Add details of the drug/protein treatments for each species. I could not find the treatment windows or amounts for the different treatments outside of the Suppl Table info, and some of the information was not included there either (e.g., amount or timing of rBMP4 on S. lamarcki). Also, an explanation of how the concentration of drug/protein and the treatment window was selected was not explained. Why was 75 uM U0126 used on S. lamarcki while 20 uM was used for O. fusiformis? Were lower amounts of U0126 not effective at blocking pERK enrichment in S. lamarcki? Were different time windows tried for S. lamarcki?

RESPONSE: We have now included three specific paragraphs in the Methods section with this information (lines 623–650). As noted above, all working concentrations match those inferred from dose-response assays (now included in Extended Data Figures 2, 4, and 6). Accordingly, 75 µM U0126 is the minimum concentration resulting in the strongest phenotype (loss of AP patterning).

Fig. 1e–l Add directional arrows or text in the legend to indicate the orientation for the lat and veg views in 1e and other panels so that the position of the future anterior pole is clear. I see the arrowhead for 4d, but it's not clear to me where anterior is. In Fig. 1l, is this a lat view? How do the axes change from 5 hpf through gastrulation? Does gastrulation change where the future anterior tissue is between 5 and 9 hpf? For Fig. 1l, a diagram at 5 hpf and one after gastrulation could be helpful. Also, please add the hpf for the in situs. Are the gastrula-stage animals shown 9 hpf?

RESPONSE: We now clarify the orientation in the figure legend (lines 1004–1005: "In **e**–**l**, animal/vegetal views are with the 4d to the bottom, and lateral views are with anteroventral to the left.").

Regarding Fig. 1l, we have kept the panel as it is because it aims to show (i) the relative approximate position of the signalling events in the late blastula at the time of axial patterning, and (ii) the inferred signalling interactions involved in axial patterning. Including the

gastrulation stage could make the panel more speculative, as we do not precisely know how the pattern we see at 5–6 hpf relates to that after gastrulation at 9 hpf. For that, we would need more detailed analyses with higher temporal resolution, as well as cell lineages and morphogenetic movements. Nonetheless, the available expression data (e.g., of apical and oral markers) suggest that the spatial organisation of embryonic territories does not change much during gastrulation (e.g., oral markers like *gsc* are already expressed at ~6 hpf in the vegetal micromeres that will eventually define the antero-ventral blastoporal lip and larval mouth [Seudre, Carrillo-Baltodano *et al* 2022]).

Fig. 3 Label the DV axis for the larval images.

RESPONSE: Done.

Fig. 4 Label the orientation of the images (quadrant for cleavage stages and AP and DV axes for larval stages). Add a diagram or text explaining what developmental stage (e.g., birth of 2d and birth of 4d) occurs at each hpf, e.g., 6, 8, 10, and 12 hpf. Possibly add summary diagrams for 6 and 8 hpf to show which cells express each gene. Add a summary diagram showing the treatment windows and resulting phenotypes for each treatment window.

RESPONSE: We now indicate the D quadrant and DV axis. Following a previous recommendation, we have moved the summary diagram showing the expression of BMP components and DV markers from the Extended Data Figure to the main display. We have also clarified the treatment windows in Methods (lines 623–650) and explained the main developmental events occurring at 6, 8, 10 and 12 hpf in the text (lines 301–305) and figure.

In Fig. 5, are the animals to the same scale? Are the partial larvae that form after 2d is ablated the same size as the controls? Please add scale bars to the images and add "n's" for how many animals had the same phenotype after 2d ablation. Add an arrowhead or line to indicate where the prototroch cells are.

RESPONSE: The episphere is slightly decreased, but roughly the same size between control and after 2d ablation, because 2d contributes only 13% of the total volume of the embryo, compared to, e.g., 3% by 4d and 62% by the four macromeres 4N (Dorresteijn, 1990). We have now added scale bars and small arrowheads to indicate where the ring of trochoblasts has formed (the protrotroch is not yet present at 12 hpf).

Suppl Table 2 Add percentages for the phenotypes (same for some of the later Suppl data tables)

RESPONSE: Done.

Suppl Tables 19–26 are empty.

RESPONSE: We are sorry for the oversight. This is now solved (new Supplementary Tables 24–31).

Reviewer #2 (Remarks to the Author):

This is a complex and interesting manuscript that addresses the evolution of axial patterning in Spiralia, a long-standing question in the evo-devo field, for which a lot of laboratories have already provided many puzzle pieces with partially contradicting observations in many species – leading to a so far confusing overall picture. To solve this issue and explain the diverse findings across spiralians, the authors have now undertaken an embryological tour de force involving four different annelid species and a lot of smart choices regarding experimentation, constrained by what is possible in the different non-conventional systems. In their multispecies approach combining Wholemount In Situ Hybridisation (WMISH), pharmacological inhibition of the ERK, BMP and Activin/Nodal signalling pathways, bulk transcriptomics, and blastomere deletions, the authors show conservation of the synergistic ERK1/2 and BMP pathways in DV axis specification in two distant annelids with conditional spiral cleavage, the basal annelid

Owenia and the Sedentarian Spriorbis. Given the similar observations in the equally conditional specifier Lottia, this is strong evidence that this situation is ancestral for Spiralia. Their findings also imply that annelids with autonomous axis specification have convergently acquired the use of Activin/Nodal signaling and restricted BMP patterning via determinants.

In Owenia fusiformis, active BMP4 signaling occurs on the dorsoposterior side, i.e. opposite to the site of expression, which is convincingly demonstrated by clear WMISH and pSMAD1/5/8 antibody staining. They show that SMAD1/5/8 signaling requires FGFR-ERK1/2 signaling, since in the presence of the U0126 inhibitor activation is abolished. In an elegant experiment, they also reveal the downstream effect of BMP signaling regarding specification, in that DMH1 treatment expands foregut and the expression of the oral marker gsc, yet represses the dorsal markers notch-like and BAMBI (leaving the hindgut marker cdx unaffected. This goes in concert with differential analysis of bulk RNA sequencing after DMH1 versus BMP treatment. Furthermore, DMH1 treatment downregulates expression of syt1, elav1 and six3/6 in the apical organ (while expression of the neuropeptide RYamide is upregulated. Similar experiments are conducted in the sedentarian annelid Spirobranchus lamarcki, with similar results, showing that these mechanisms are remarkably conserved.

Next, the authors investigate the role of BMP signaling in Platynereis, which has so far only been marginally addressed, by extensive WMISH analysis of dorsal and ventral markers, and by bulk RNAseq. Here, inhibition with DMH1 leads to circumferential expression of Onecut/Hnf6, which is a neural marker at later stages, and strong upregulation in the RNAseq data. This goes in concert with significant upregulation of neural genes at later larval stages. In an elegant ablation experiment of the 1D versus 2d cell in the Platyereis embryo the authors further localize the dorsalising signal to the 2d cell. This is partially rescued with BMP4 incubation.

Finally, the authors focus on the putative ancestral role of Nodal signaling in annelids, by treating Owenia embryos with the Nodal inhibitor SB431542 and recombinant Activin-A at the time of axial patterning. Interestingly, treatment of Platyereis larvae with Nodal inhibitor does not lead to DV patterning defects but only absence of the developing adult eyes. The authors document their findings with a rich and clear collection of figures and provide instructive summary schemes to explain their data. In addition, they provide an impressive 10 extended data figures with equal accuracy and elaborate documentation.

Most important, with their broad approach the authors indeed come a new comprehensive characterization, interpretation, and understanding of axis formation in Spiralia. They explain the overall situation as an ancestral pattern undergoing what they call "developmental system drift", in the context of the (long-known and -debated) repeated transition towards autonomous specification in Spiralia. This contributes a great deal to understand an old basic question of comparative embryology – the phenomenon of conditional versus autonomous specification. The authors are to be congratulated for an impressive developmental opus, which should attract the interest of a broad readership interested in developmental biology.

RESPONSE: Many thanks for the positive comments on our work.

I have some points and suggestions to improve the manuscript.

1) The authors interprete their U0126 data in that FGFR-ERK1/2 establishes the bilateral symmetry in O. fusiformis embryos by regulating BMP signaling. While it is true that it is required for SMAD1/5/8 activation, it appears that DV patterning is still accomplished by BMP signalling, and FGFR-ERK1/2 signaling rather acts as a gate for this mechanism to be active. Why do you read the data that FRGR-ERK1/2 is doing the actual patterning job? It's a precondition but does not necessarily convey the spatial information. In a similar direction, why is the BMP specification activity leading to gsc foregut expansion not considered AP

specification (or a mix of both)? In a way, co-specification of both axes is included in the authors' own summary, stating that BMP signaling activates dorsoposterior genes (and sustain Notch/Delta activity) and represses anteroventral fates.

RESPONSE: We agree with the reviewer's interpretation. Following this and reviewer #3's suggestions, we have now thoroughly amended the manuscript (including abstract, introduction, and discussion) to separate dorsoventral development into a specification (FGF-ERK1/2-dependent) and a patterning (BMP-dependent) process. As the reviewer points out, however, anteroposterior and dorsoventral development is strongly intertwined and likely involves sequential and mutually inductive/repressive interactions, which would explain why *gsc*, a ventro-anterior marker, expands in the absence of dorsal tissue, as also observed when blocking ERK1/2 signal [Seudre and Carrillo-Baltodano et al 2022]. This could be because differential proliferation of dorsal cells towards the ventral midline is required for axial elongation along the anteroposterior axis, as described in other spiralians (e.g., Damen and Dictus, 1997; van den Biggelaar *et al*, 2002). This is something to explore in the future, especially regarding the interplay between BMP signalling and Notch/delta activity. We now indicate, however, that BMP activity is also broadly required to ensure the correct development of the bilateral symmetry (both AP and DV; lines 187–191: "Therefore, BMP signalling establishes a gradient of pSMAD1/5/8 activity that is necessary and sufficient to pattern the dorsoventral axis in *O. fusiformis* and to ensure a coordinated development of bilateral symmetry in these embryos, perhaps by determining the cells in the D quadrant that will contribute to axial elongation along the primary axis[30].").

2) Similarly questioning their interpretation, I am not sure whether I would not speak of antineural activity if syt1 and elav expression are downregulated upon DMH1 treatment in Owenia; the continued expression of RYamide is a weak argument in comparison to the synapse and axonal marker gene that disappear. My suggestion is to diagnose antineural activity yet with the exception of a neuropeptide. (Remember, Trichoplax has no nervous system but neuropeptides).

RESPONSE: We thank the reviewer for suggesting that we reconsider the implications of the data on the neurogenic role of BMP in *Owenia*. In our original submission, we preferred to be cautious and discard a neurogenic role given the seemingly conflicting results between the lack of expression of neural-related genes after BMP inhibition, the limited overexpression of neurogenic markers with rBMP (although *elav1* indeed seems somewhat overexpressed; see new Figure 3c), and the presence of RY immunoreactivity. However, we agree with the reviewer that greater weight should be placed on changes in the expression of neurogenic markers, as immunostaining may reflect the presence of neurosecretory cells rather than neurons (as in *Trichoplax*). We have now amended the section accordingly (lines 215–237), proposing a potential proneural role (because inhibition of the pathway leads to a lack of neurogenic markers), as in other molluscs. We have also changed the Discussion accordingly (lines 520–524) and moved the former Extended Data Figure 3 to the main text (new Figure 3), as suggested by reviewer #3.

3) Can the authors comment how the adult eyes are missing in Nodal inhibitor SB431542 – treated Platynereis larvae? How about the larval eyes? How does the left-right asymmetry in expression possibly relates to this? Do they observe any left-right effects?

RESPONSE: Thanks for highlighting this interesting point. SB431542-treated larvae lack adult eyes but still form larval eyes in what appears to be a symmetrical phenotype (new Figure 9a). This suggests that Nodal might contribute to and have a downstream role in dorsoventral development in *Platynereis*, as also observed in *Owenia*. However, whether this is directly related to its asymmetric expression and its potential involvement in left-right development remains unclear. Investigating it further goes beyond the scope of the manuscript, as it would

require a detailed analysis of the cell lineage pattern of the episphere, which, at the stage of analysis, contains over 200 cells. We now discuss this in the text (lines 474–476: "However, we noted the absence of adult eyes at 20 µM in the dorsal head region, although these larvae still formed larval eyes in what appeared a left-right symmetrical phenotype (Fig. 9a).").

Reviewer #3 (Remarks to the Author):

This is a technically excellent and important study, showing that BMP signaling is patterning the dorsal-ventral axis in Owenia and Spirobranchus, two annelids that have the presumed ancestral mode of spiralian development ("equal cleavage/ conditional specification of the D quadrant") This is important and clarifying for the field because it shows that these embryos are similar to what is known from molluscs, and thus strongly implies that the ancestor of molluscs and annelids (and thus maybe the spiralian common ancestor) used BMP signaling to pattern the DV axis, along with ERK to specify the axis. Until now, the situation in annelids has been very confusing, with marked differences between groups that have been examined, all of which have the presumed derived mode of spiralian development ("unequal cleavage/ autonomous specification of the D quadrant"). The final part of the paper, which is equally very important, clarifies the role of BMP signaling in one of these latter embryos, Platynereis, and make the results comparable to studies of BMP signaling in other spiralian groups. (There was a paper some time ago that studied the role of BMP signaling in later Platynereis development, but we have not known what the pathways was doing during the earlier phase of spiralian organizer signaling.) This section is further strengthened by 2d organizer cell ablation, which is a key line of evidence for the role of the pathway at these stages. This could easily be 3 very solid papers in good specialist journals—taken together it is a major advance that is definitely appropriate for Nature Communications.

RESPONSE: Many thanks for the positive appraisal of our work.

I have some concerns about terminology and making it clear how this work fits in with the existing literature. I am aware that some of this is nuance and likely caused by writing a paper in a non-native language, (I would be incapable of that!). Please take my comments as constructive thoughts on a paper that I strongly support.

Major comments:

First, the ms is not clear, and needlessly confusing, about what is known about the relationship between BMP signaling and the ERK1/2 signaling pathway in spiralians.

line 61-: [After they describe the similarities between dueterostomes and ecdysozoans in terms of BMP patterning of the DV axis] " However, in Spiralia (e.g. molluscs and annelids), the third major lineage of bilaterian animals characterised by an ancestral stereotypical early embryonic program named spiral cleavage, the initial specification of the DV axis ancestrally involved the Fibroblast Growth Factor receptor (FGFR) and ERK1/2 pathways. Notably, an opposing gradient along the secondary body axis of BMP antagonists and pSMAD1/5/8 also occurs in Cnidaria, the sister group to Bilateria. Therefore, an upstream role of the BMP pathway in defining the body symmetry and DV axis is likely ancestral to Bilateria (Fig. 1a), rendering the condition in Spiralia a pronounced and unique yet still poorly understood transition in the 69 mechanisms controlling animal body patterning.".

Similarly, line 86: "Therefore, the ancestral role of the BMP signalling pathway remains contentious in Spiralia, preventing a fundamental understanding of how the shift to an FGFR-ERK1/2-mediated DV axial cue originated during animal evolution."

These statements seem to be making the argument that BMP patterning of the secondary axis is ancestral for Cnidaria+Bilateria, but that in Spiralians it has been lost. More specifically, they are saying that the available evidence suggests that there was a switch between BMP and

ERK1/2. However, if BMP was ancestral for bilaterians, and it is known to be patterning the DV axis in two gastropod molluscs (Tritia and Lottia), which they indicate elsewhere, then the obvious interpretation is that BMP patterning of the DV axis was present in the common ancestor of spiralians, and retained in molluscs at least, but lost in the annelids that had been looked at before this work. In addition, since in these two gastropods (Tritia and Lottia) both ERK signaling and BMP signaling are found to be involved in specification and patterning of the DV axis, it does not make sense to hypothesize a "switch" from BMP to ERK. They can both be there. One way they could say this more clearly is simply to say that the role of ERK signaling in the common ancestor of molluscs and annelids was well-supported (thanks to the great 2021 paper from the M-D group, and previous mollusc work), but the role of BMP signaling in that common ancestor is much less clear.

RESPONSE: This is an excellent suggestion. We have rewritten the abstract, introduction, and discussion in accordance with this recommendation and adjusted other parts of the manuscript.

[A minor point here: for the statement above they cite 7-9, the senior author's very nice paper on ERK signaling in Owenia, which makes sense, but also the Tritia BMP paper, and an organizer review, neither of which claim that the ancestral spiralian had ERK signaling instead of BMP, as implied here.]

RESPONSE: In the updated introduction, we removed the references to the *Tritia* BMP paper and the organiser review in that sentence.

A related issue, and one that could help resolve and clarify these ideas, is that they are comparing two different kinds of events. BMP signaling seems to have a conserved role in patterning the secondary axis across animals. But it does not have a conserved role in specifying the secondary axis. In at least two molluscs, (and to a reduced extent in Platynereis) BMP signaling does pattern the secondary axis, but it does not specify it. That is true in flies and frogs as well. (In fact in flies, the specification of the axis is also mediated by ERK signaling (!) downstream of gurken and torpedo. And as the authors know well, the specification of the secondary axis in frogs is mediated by the WNT pathway, but then patterned by BMP signaling.) The preceding specification of the secondary axis, or D quadrant, in molluscs usually requires ERK signaling.

I think that making the distinction between the specification of the axis and the patterning of the axis (and being consistent about it) would clarify the paper a great deal.
RESPONSE: We have amended the manuscript as suggested (see point above).

Minor point related to this: line 55, "In Deuterostomia (i.e., chordates, echinoderms, and hemichordates) and Ecdysozoa (e.g., arthropods), the Bone Morphogenetic Protein (BMP) pathway specifies the DV axis…" The pathway doesn't specify the DV axis in flies or frogs, it patterns it.

RESPONSE: Changed.

Overall, it seems to me that the clearest way to describe the significance of the results in this paper is to say that it was unclear what the ancestral roles of these two pathways were in spiralians, because their known roles in Platynereis and Capitella were so different from molluscs, but examination of Owenia and Spirobranchus has clarified that the ancestral situation in annelids is similar to molluscs.

Once again, it is very clarifying for the field.

RESPONSE: Many thanks; as stated above, we have amended the manuscript (abstract, introduction and discussion) as proposed.

Another important semantic issue that I have with this paper is the use of conditional vs autonomous to categorize different spiralian embryos. What I think they are referring to when

they use this terminology is the distinction between embryos that use conditional versus autonomous mechanisms to specify the D quadrant. Importantly, even in embryos that seem to use autonomous specification of the D quadrant, most of lineages of the embryo are subsequently patterned by conditional mechanisms. This is probably best understood in Tritia based on cell ablation experiments by Clement, and subsequent molecular work, especially on BMP and ERK signaling. So it is confusing and misleading to call this embryo "autonomous". Further confusing things is the fact that the classic Wilson experiment that defined autonomous specification was done in an embryo that they would call "conditional" (Patella).

The authors could address this by specifically referring to embryos that specify their D quadrants autonomously vs conditionally. Alternatively, the standard in the field is the use of "unequal" to refer to embryos that specify the D quadrant by presumed autonomous mechanism, and "equal" to refer to those that specify by presumed conditional mechanisms. (Equal vs unequal referring to the first two divisions and the subsequent size of the cells at the four cell stage.) The authors do use this terminology in their figures, and I think it is preferable, for two reasons. First, as far as I know, we don't know the mechanism of autonomous specification of the D quadrant in any spiralian—meaning, what is the determinant that is inherited by the presumptive D lineage? And we don't really know the molecular mechanism of conditional specification in any equal cleaving spiralian. Finally, Gary Freeman's experiment, where he mechanically increases the size of one of the macromeres at the 4 cell stage in Lymnaea and it becomes the D cell, indicates that the size alone of the cell is sufficient, strengthening the case for thinking of the inequality of the sizes could possibly be the key point. The authors should either switch to "unequal/cleaving" or describe what they mean by conditional vs autonomous embryos more clearly, including consistently using "conditional/autonomous specification of the D quadrant"

RESPONSE: Thanks for the suggestion. We have switched to using "equal/unequal" to align with field standards and "conditional/autonomous" only when referring to the D quadrant specification.

Minor comments:

Overall, it feels like there is an unusually large proportion of data in the supplemental/extended data section, and the paper would benefit by moving more to the main text. If they can fit more in the main text under the journal rules, I would urge them to do it. I would also urge the editors to allow more if that is at their discretion.

RESPONSE: Given the editorial limit of 10 display items, we have moved the former Extended Data Figures 3, 6, 7, and 10 into the main text, now becoming Figures 3, 6, 8, and 9. This also addresses the reviewer #1's suggestion to explain the *Platynereis* data in more detail and reviewer #2's comment about the neural role of BMP signalling in *Owenia*. We have amended the manuscript accordingly.

The authors should be careful about claims about what is ancestral for spiralians based on evidence from molluscs and annelids, because they are more closely related to each other than many other spiralian clades.

RESPONSE: We have toned down claims of what is ancestral to Spiralia based on molluscs and annelids (e.g., lines 56–58: "Therefore, an early role of the BMP signalling pathway in setting up the DV axis is likely ancestral to Spiralia…") and included a statement and reference at the end of the discussion indicating current uncertainties around the internal tree topology of Spiralia, which complicates the reconstruction of ancestral characters to this large group (lines 592–595).

Line 95: "This is like what has been described in a mollusc with conditional spiral cleavage12,24". It is also like what has been described in Tritia, with ERK being activated in

3D, then BMP signaling creating a DV gradient of Smad1/5/8 and being required for normal DV fates. They include the Tritia BMP paper in a later similar statement (line 159).

RESPONSE: We have amended the statement as suggested (lines 91–92: "This is similar to what has been described in molluscs with equal and unequal cleavage[12,13,24]…")

Lines 267 to 276: I thought smad6 was an inhibitory smad, so it's not clear why they are including it with "putative targets" vs "antagonists". It is totally plausible that an inhibitor would also be a target, it just needs to be clarified.

RESPONSE: This is likely the case in *Platynereis*. *Smad6* is a downstream target of BMP signalling (as observed via RNA-seq following DMH1 treatment; Figure 6i), and, given its presumed inhibitory function, it might generate a negative feedback loop to limit BMP signalling in dorsal head progenitors. We now clarify the text (lines 305–306: "Our expression profile analysis reveals that the genes *bmp2/4*, *smad6* (a putative BMP signalling antagonist[47],…"; lines 374–377: "The fact that the antagonist *smad6* is a downstream target of BMP signalling suggests the existence of autoregulatory feedback loops that might help restrict the pathway activity to the dorsal head progenitors.").

Lines 342-343: Confusing, I had to read a few times to be sure they weren't describing an experiment where all of these cells were ablated at the same time. Maybe describe phenotype of 2d ablation first and then say you did the other cells and it did not look the same?

RESPONSE: We have clarified this sentence (lines 392–395: "To test this, we obtained batches of embryos at various cleavage stages and ablated a single D quadrant blastomere from each. In particular, we targeted the 2d cell, as well as its progenitor, the 1D blastomere, its sister cell, the 2D blastomere, and its early progeny, the 3d and 4d cells (Fig. 7a).").

377-384: At the end of this section they write: "Therefore, Activin/Nodal is unnecessary to establish the DV polarity in O. fusiformis. Yet, the inhibition of this pathway is reminiscent of the effects of inhibiting the BMP signalling". Don't they mean "Yet the ectopic activation of this pathway is reminiscent of the effects of ectopic activation of BMP signaling."

RESPONSE: We have clarified this section of the text. We now indicate that the effect of SB431542, with a smaller posterodorsal side, is "mildly reminiscent of the effects of BMP signalling inhibition" (line 430) and end the paragraph as suggested (lines 437–440: "Therefore, Activin/Nodal is unnecessary to establish the initial DV pattern in *O. fusiformis*. Still, it seems to contribute to its subsequent development, as evidenced by phenotypic similarities following ectopic activation of Activin/Nodal and BMP signalling.").

Fig. 1: The symbol for unequal cleaver is confusing, maybe make one of the cells clearly bigger in this one?

RESPONSE: Changed as suggested.

Fig. 2d: This panel is very confusing. Why does control have a phenotype and why is "mock" different from control? And is this figure reporting percentages side by side for different phenotypes for different treatments, as indicated in the text, lines 165 to 169? If so, very confusing. If the only point of this panel is to report the percentages of treated embryos with described phenotypes, I think it is fine to just report percentages in the text.

RESPONSE: We have clarified panel d in Figure 2. We now report the percentage of larvae with the predominant phenotype in each condition: the control samples (DMSO for DMH1 and "mock" [BSA in 4 mM HCl] for rBMP4) and in the treatments. We define the phenotype on the top (wild-type, no chaetae, all chaetae). We amended a similar plot for Activin/Nodal treatments in Figure 8f.

The discussion on DSD is interesting. It made me think that since these crucial pathways are evolving more quickly than we might have predicted, we need even more sampling to confirm what is conserved vs. derived.

RESPONSE: We agree with the reviewer. We have added a sentence in the discussion to reflect this (lines 592–595: "Therefore, crucial pathways involved in axial polarity can evolve more quickly than predicted, which, given the current uncertainties in the interrelationships of Spiralia[67], calls for expanding the taxon sampling in developmental studies across and within spiralian phyla to better identify conserved and derived traits in body patterning.")

Response to Reviewers

Reviewer Comments

Reviewer #1 (Remarks to the Author):

Overall, the resubmitted manuscript is much clearer, and describing the function of BMP signaling in terms of DV axis patterning vs specification makes comparisons with other taxa easier. The addition of the dose response curves and methodological details of treatments for each drug/protein in each species also satisfies the questions I had about this. The edits to the figures make them easier to understand and more applicable for a broader audience, and the extended descriptions of data from P. dumerilii and S. lamarcki improve the manuscript.

All of my other questions and suggestions have been met through either new data (e.g., dose response curves and additional time points for labeling with pSMAD 1/5/8 or pSMAD and pERK1/2 in Owenia and Spirobranchus, respectively), changes to the manuscript (e.g., adding treatment details to the Methods), or replies in the rebuttal.

This manuscript clarifies the function of signaling systems including BMP that control dorsoventral patterning in annelids, thus illuminating the ancestral role of these pathways across Bilateria. The data are of high quality and approach the question from multiple angles, and the comparisons across three taxa with different modes of organizer specification enable a better understanding of developmental systems drift. Overall, this is a seminal manuscript. I highly recommend publication in Nature Communications and congratulate the authors on their work.

Minor edits

line 39 implies

line 59 symmetry-breaking

line 60 axes; involved

lines 249-250 Fig. 4b

line 252 Fig. 4a

RESPONSE: We thank the reviewer for the positive appraisal of our reviewed manuscript. We have included those five text changes in the revised submission.

Reviewer #2 (Remarks to the Author):

The authors have carried a thorough revision of the manuscipt, having changed large part of the text and figures and also revising/amending some of their interpretations. My main concerns / suggestions had been to clarify the role of BMP in the simultaneous co-patterning of both axes, which has been addressed and is now adequately revised by the authors.

Also, they have reconsidered a neurogenic role of BMP in Owenia, based on their observation that inhibition of the pathway leads to a lack of neurogenic markers, in line with a potential proneural role. I am fully satisfied with these revisions and do not raise other points.

RESPONSE: We thank the reviewer for the positive evaluation of our work and constructive criticism.
